# Supplementary material for: Protein mimetic 2D FAST rescues alpha synuclein aggregation mediated early and post disease Parkinson’s phenotypes
Source: Nat Commun. 2024 Apr 30;15:3658. doi: 10.1038/s41467-024-47980-4 (PMC11061149; doi:10.1038/s41467-024-47980-4)
Supplement: Supplementary file 1 — Supplementary Information [file 41467_2024_47980_MOESM1_ESM.pdf]

Supplementary Information

Protein Mimetic 2D FAST Rescues Alpha Synuclein Aggregation Mediated Early and Post Disease Parkinson's Phenotypes

Nicholas H. Stillman[1,2], Johnson A. Joseph[1,2], Jemil Ahmed[2,3], Charles Zuwu Baysah[1,2], Ryan A. Dohoney[1,2], Tyler D. Ball[1,2], Alexandra G. Thomas[1,2], Tessa C. Fitch[2], Courtney M. Donnelly[1,2], Sunil Kumar[1,2,3#]

[1]Department of Chemistry and Biochemistry, F.W. Olin Hall, 2190 E Iliff Ave, University of Denver, Denver, CO 80210, USA.
[2]The Knoebel Institute for Healthy Aging, 2155 E. Wesley Ave, Suite 579, University of Denver, Denver, CO 80208, USA.
[3]Molecular and Cellular Biophysics Program, Boettcher West, Room 228, 2050 E. Iliff Ave, University of Denver, Denver, CO 80210, USA.

[#]Correspondence: sunil.kumar97@du.edu

SUPPLEMENTARY METHODS

Synthetic Methods and Materials:

All synthetic starting materials, reagents, and solvents were purchased from commercial suppliers. Silica XG TLC plates (with UV254, aluminum backed, 200 μm thickness) and silica gel (standard grade, particle size = 40–63 μm, 230–400 mesh) for flash column chromatography were purchased from Sorbent Technologies, Inc. (Norcross, GA). 6-chloro-5-nitro-2-picoline (CAS: 56057-19-3) was obtained from Chem Scene (Monmouth Junction, NJ). All other general solvents, reagents, and chemicals (not synthesized) were purchased from Oakwood Chemical (Estill, SC), Sigma Aldrich (St. Louis, MO), or Fisher Scientific (Allentown, PA). Reactions conducted in anhydrous solvent were carried out in flame-dried round bottom flasks, under the atmosphere of argon gas. Anhydrous solvents were obtained through an on-site Innovative Technology PURESOLV solvent purification system (University of Denver). A Bruker (Billerica, MA) 500 MHz Ultrashield Plus was used to collect all Nuclear Magnetic Resonance (NMR) spectra. Chemical shifts are measured relative to the residual solvent peaks as an internal standard set to δ 7.26 ppm and δ 2.50 ppm for $CDCl_3$ and DMSO-d_6, respectively, for ^{1}H NMR. All ^{13}C NMR spectra were referenced to 77.16 ppm for $CDCl_3$ and δ 39.52 ppm for DMSO-d_6. The following abbreviations are used to describe observed peak splitting patterns: s = singlet, d = doublet, dd = doublet of doublets, t = triplet, q = quartet, and m = multiplet. HRMS spectra of the synthesized were obtained by the Mass Spectrometry Lab at the University of Illinois (Urbana, IL) or at the University of Denver (Denver, CO). The following reaction conditions for amide coupling and aromatic substitution (SnAr) were developed specific to each reaction for the purpose of reducing the number chromatography purification steps. The reported yields refer to spectroscopically (^{1}H NMR) and/or chromatographically homogeneous materials, unless otherwise stated.

Synthesis of Starting Monomers (1)

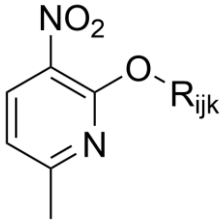

A solution of 6-chloro-5-nitro-2-picoline (2.00 g, 11.6 mmol) in toluene (30 mL) under argon (g) was stirred at 0 °C for 15 min before adding a primary alcohol [tert-butyl 2-hydroxyacetate for NS41] (18.5 mmol, 1.6 eq.). After stirring the solution for an additional 15 min at 0 °C, NaH (60% dispersion in mineral oil) or Na metal (18.5 mmol, 1.6 eq.) was added incrementally over 20 min. The reaction was then stirred at 0 °C for 50 min and then at r.t. for 5 h. After the disappearance of the starting material was observed via TLC, the reaction mixture was partitioned between EtOAc and brine. The organic layers were combined, dried over anhydrous Na_2SO_4, and concentrated under vacuum (yellow to brown solid, 78.1 - 97.2%).

General method to reduce arylamides (2,5)

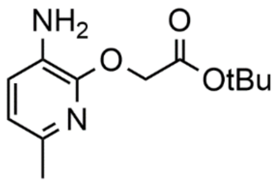

Pd/C (20% by wt., 0.298 g) was added to a solution of nitro arylamide (1.49 g, 5.55 mmol) in EtOAc (15 mL) and continuously stirred while bubbling H_2 (g) for 3 h at r.t. After confirming the disappearance of the starting material via TLC, the reaction was filtered and concentrated. The use of a rotary evaporator in this reaction is ill-advised due to the risk of product degradation. The product of this reaction was used in the next step without being further characterized (red/brown oil, 95.6%).

Synthesis of 6-chloro-5-nitropicolinic acid

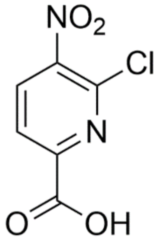

To a stirring solution of 6-chloro-5-nitro-2-picoline (5 g, 29.0 mmol) in conc. H_2SO_4 (45 mL), potassium dichromate (11.4 g, 38.6 mmol, 1.3 eq.) was added portionwise over 20 min and refluxed at 60 °C overnight. After confirming the absence of starting material via TLC, the reaction was placed on ice and quenched with water (25 mL). The reaction was then extracted with EtOAc (6×125 mL), dried over Na_2SO_4, and concentrated under vacuum to yield the pure product (pale yellow solid, 5.63 g, 96.0%).

One-pot Amide Coupling (3)

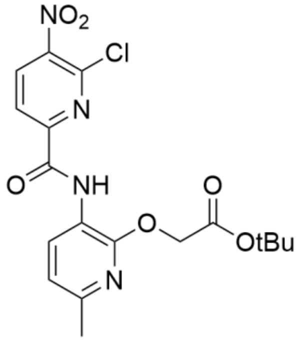

A solution of 6-chloro-5-nitropicolinic acid (0.335 g, 1.65 mmol, 1 eq.) and compound **(2)** (0.394 g, 1.7 mmol) in DCM (anhydrous, 10mL) was equilibrated for 15 min at 0 °C under argon (g). To the stirring solution was added TEA (0.690 mL, 4.96 mmol, 3 eq.) and equilibrated for an additional 60 sec at 0 °C, followed by the addition of thionyl chloride (0.360 mL, 4.96 mmol, 3 eq.). The reaction mixture was stirred for 45 min at r.t. and the disappearance of starting material was confirmed by TLC. The volatiles were removed on a rotovap, and the resulting product was partitioned between EtOAc and 1M HCl (1×15 mL). The organic layer was then washed

with 1M NaOH (3×15 mL), followed by brine (1×15 mL), dried over anhydrous Na_2SO_4, and concentrated under vacuum (yellow solid, 0.699 g, 94.3%).

General Method for Aromatic Substitution (4,7)

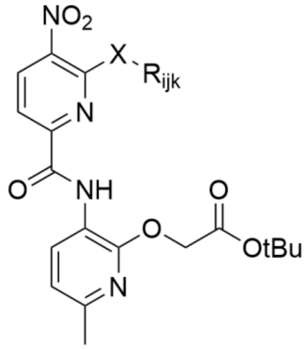

To a solution of compound **(3)** or **(6)** (0.015 g, 0.036 mmol) in DCM (3 mL), a primary amine or thiol [cyclohexylmethanamine for NS55, tryptamine for NS132] (0.355 mmol, 10 eq.) and N,N-Diisopropylethylamine (DIPEA) (0.071 mmol, 20 eq.) were added and stirred at r.t. for 3 h. For reactions involving reagents that exhibit low solubility in DCM, e.g., tryptamine and C-(1-H_Pyrazol-3-yl)-methylamine, DMF was substituted as the solvent. The reaction mixture was dried following confirmation that the starting material had disappeared via TLC. For the removal of non-volatile reagents, flash chromatography is required (10 to 80% EtOAc in hexanes, v/v, over 8 min). The products appear as yellow solids with a yield ranging from 48.0% to 94.3%.

Synthesis of 6-chloro-5-nitropicolinoyl chloride

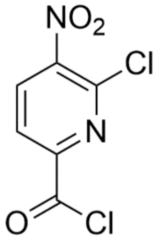

A solution of 6-chloro-5-nitropicolinic acid (0.500 g, 2.47 mmol) in $SOCl_2$ (10 mL) was refluxed overnight at 70 °C. After confirming the reaction was complete via ^1H NMR, the volatiles were removed under vacuum and the final product was preserved under argon (g) at -20 °C (pale brown solid, 0.516 g, 94.8%).

Method for Tripyridyl Amide Coupling (6)

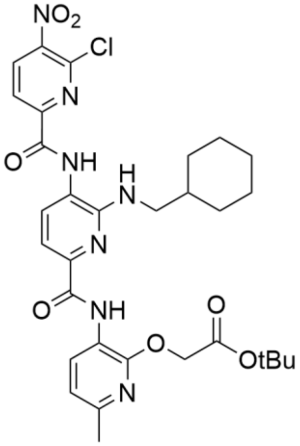

A solution of 6-chloro-5-nitropicolinoyl chloride (0.076g, 0.342 mmol, 1.3 eq) in DCE (10 mL) was stirred at 0 °C for 10 min. After equilibration, saturated $NaHCO_3$ (aq., 10 mL) was added and the solution stirred vigorously for 1 min. A solution of compound **(5)** (0.124 g, 0.263 mmol) in DCE (3 mL) was then added dropwise over 60 sec and the solution was stirred at 0 °C. TLC confirmed the disappearance of the starting material after 10 min and the resulting solution was partitioned between DCM and saturated NaOH. The combined organic layers were washed with brine, dried over Na_2SO_4, and concentrated under vacuum (orange solid, 0.168 g, 97.4 %).

General Method for Deprotection (8)

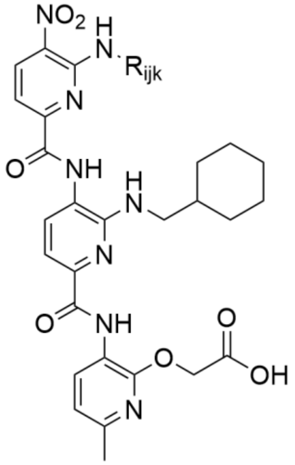

Compound (7) (0.004 - 0.010 g) was dissolved in 3 mL of DCM followed by the addition of 1:1 TES:TFA (0.5 mL each), stirring 60 sec prior to the addition of TFA. The solution was allowed to stir at r.t. for 3 h, confirming the disappearance of starting material by TLC. Upon completion of the reaction, volatiles were removed under vacuum and the resulting product was washed with diethyl ether (3×2 mL, 0 °C) to yield the pure product; Yellow-Orange solids with a yield ranging from 50.8 to 97.1%.

Due to the sensitivity of indoles to acidic conditions, alternative methods of deprotection were required for molecules containing this functional group[1,2]. To a stirring solution of NS72 (0.012 g, 0.022 mmol) in acetonitrile (6 mL), water (40 µL) and elemental iodine (0.002g, 30% mol) were added, and the solution refluxed at 75 °C for 5.5 h. After equilibrium was reached, the solution was extracted with $Na_2S_2O_3$ (aq., 4 mL) and DCM (2×10 mL),

and the combined organic layers were dried over Na_2SO_4. Flash chromatography was used to recover the starting material (0 to 40% EtOAc in hexanes, v/v, over 6 min, 0.002 g, 16.4%) and to isolate the pure product (0 to 20% methanol in DCM, v/v over 6 min, 0.005g 42.9%).

To a stirring solution of NS132 (0.023g, 0.030 mmol) in DCM (6 mL), zinc bromide ($ZnBr_2$, 0.033 g, 0.147 mmol, 5 eq) was added and stirred 72 h at r.t. Additional solvent was added as needed to replace evaporated solvent. Subsequently, deionized water (30 mL) was added, and the solution stirred vigorously for an additional 2 h. The solution was then extracted with DCM (3×10 mL) and the combined organic layers were washed with brine, dried over Na_2SO_4, and concentrated under vacuum (orange solid, 0.014 g, 65.2%).

Synthesis of NS163

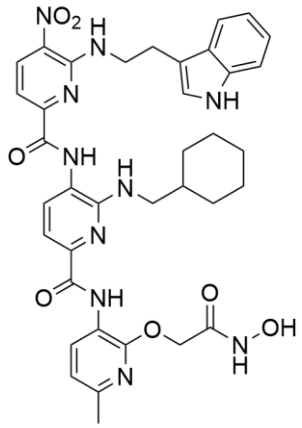

A solution of NS132 (0.009g, 0.011 mmol) and CDI (0.003g, 0.016 mmol, 1.5 eq.) in 2 mL of THF (anhydrous) was stirred at r.t. for 1 h. After adding hydroxylamine hydrochloride (0.002g, 0.022 mmol, 2 eq.), the reaction was stirred at r.t. for 20 hours. The volatiles were removed on a rotovap, and the resulting product was redissolved in EtOAc and washed with brine (3×5 mL). The organic layer was dried over Na_2SO_4 and concentrated under vacuum. Flash chromatography (0 to 15 % EtOAc in Hexanes, v/v, over 15 min) yielded the final product (yellow solid, 0.002g, 23.7%).

SUPPLEMENTARY FIGURES

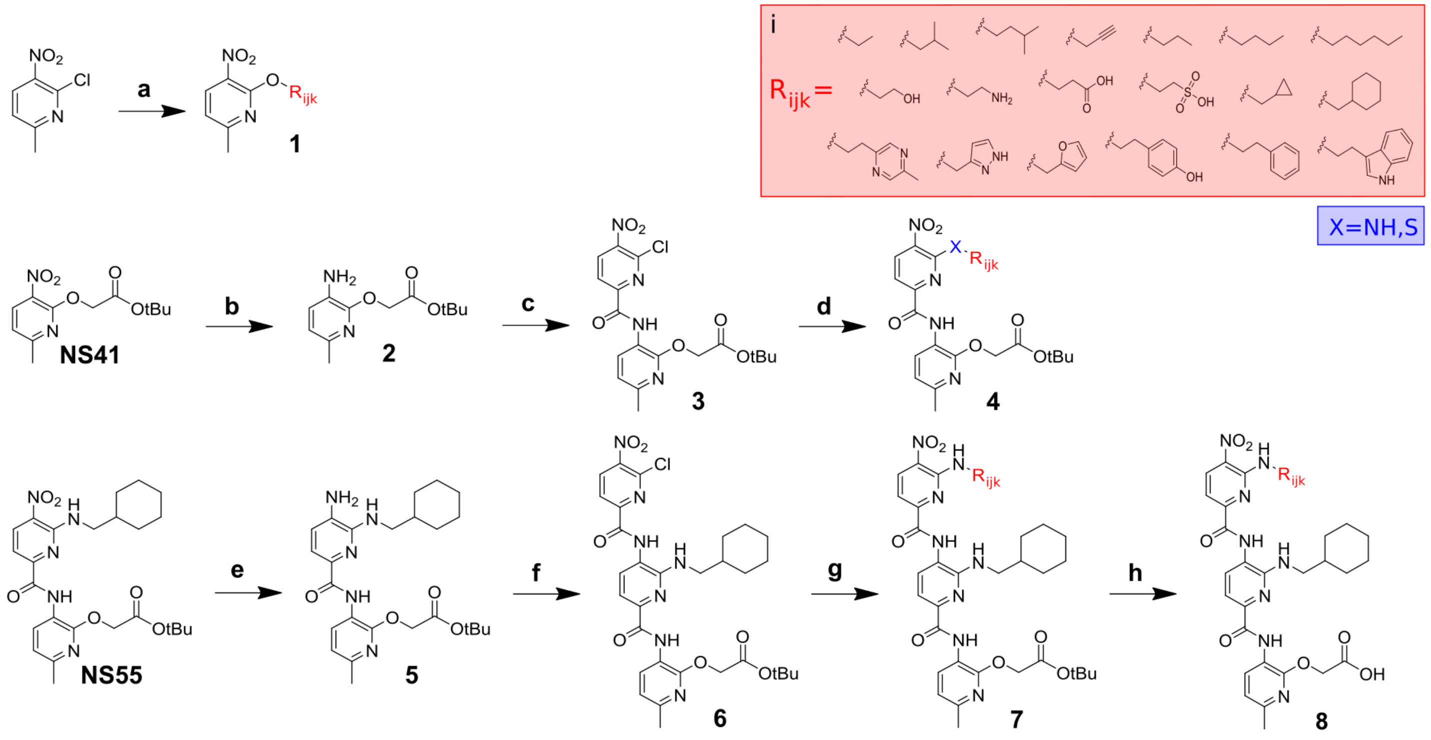

Supplementary Fig. 1. General Synthesis of OPs using 2D-FAST. **a**, primary alcohol, NaH (60% dispersion in mineral oil) or Na metal, toluene, 50 min at 0 °C, then 5 h at room temperature (r.t.) **b,e**, Palladium on activated carbon (Pd/C), H_2 (g), ethyl acetate (EtOAc), 3 h at r.t. **c**, 6-chloro-5-nitropicolinic acid, dichloromethane (DCM, anhydrous), triethylamine (TEA), thionyl chloride 0 °C to r.t., 45 min. **d,g**, Primary amine/thiol, N,N-Diisopropylethylamine (DIPEA), DCM 3 h at r.t. **f**, 6-chloro-5-nitropicolinoyl chloride, dichloroethane (DCE), saturated sodium bicarbonate ($NaHCO_3$), 10 min at 0 °C **h**, Triethylsilane (TES):trifluoroacetic acid (TFA) (1:1), DCM, 3-6 h at r.t. **i**, Chemical structures of various side chains appended on the dipyridyls and tripyridyls.

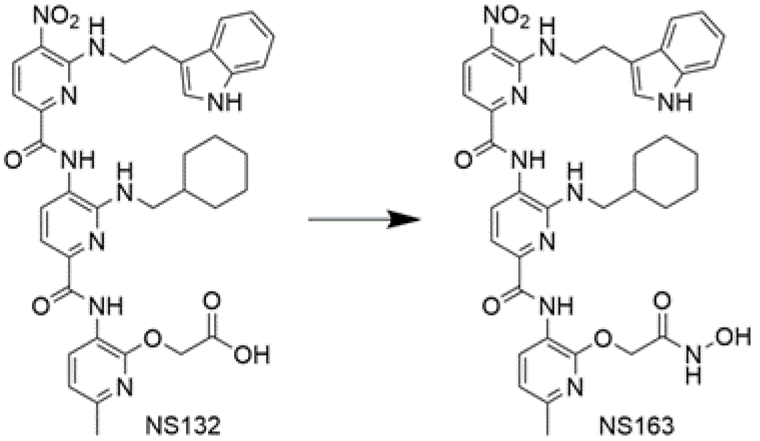

Supplementary Fig. 2. Synthesis of NS163. Reagents and conditions: Carbonyldiimidazole (CDI), tetrahydrofuran (THF, anhydrous), stir 1 h at r.t., hydroxylamine hydrochloride, 20 h, r.t.

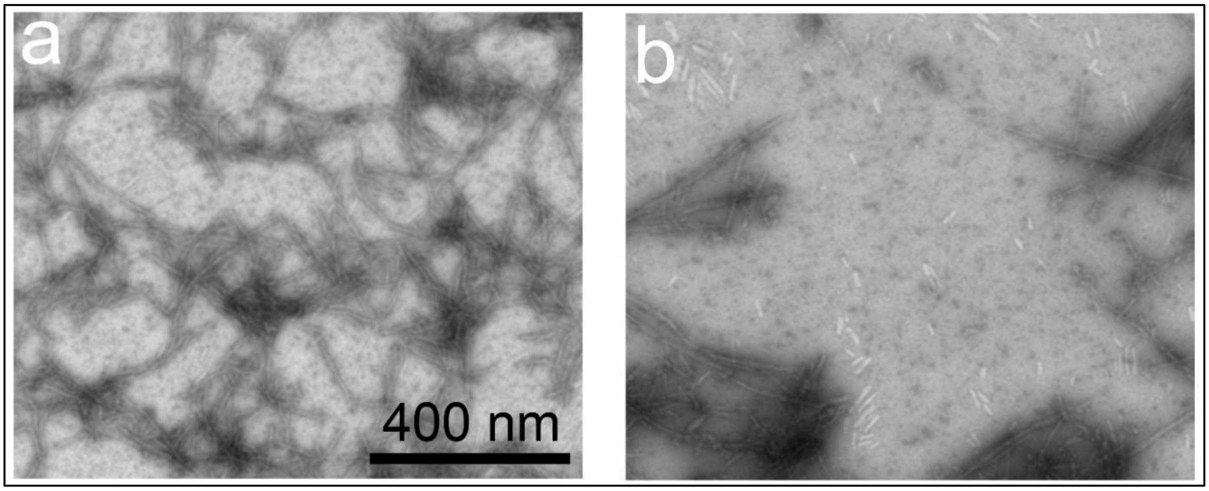

Supplementary Fig. 3. The TEM images of the aggregated solution of 100 μM αS for four days in the absence (a) and presence (b) of NS41 at an equimolar ratio.

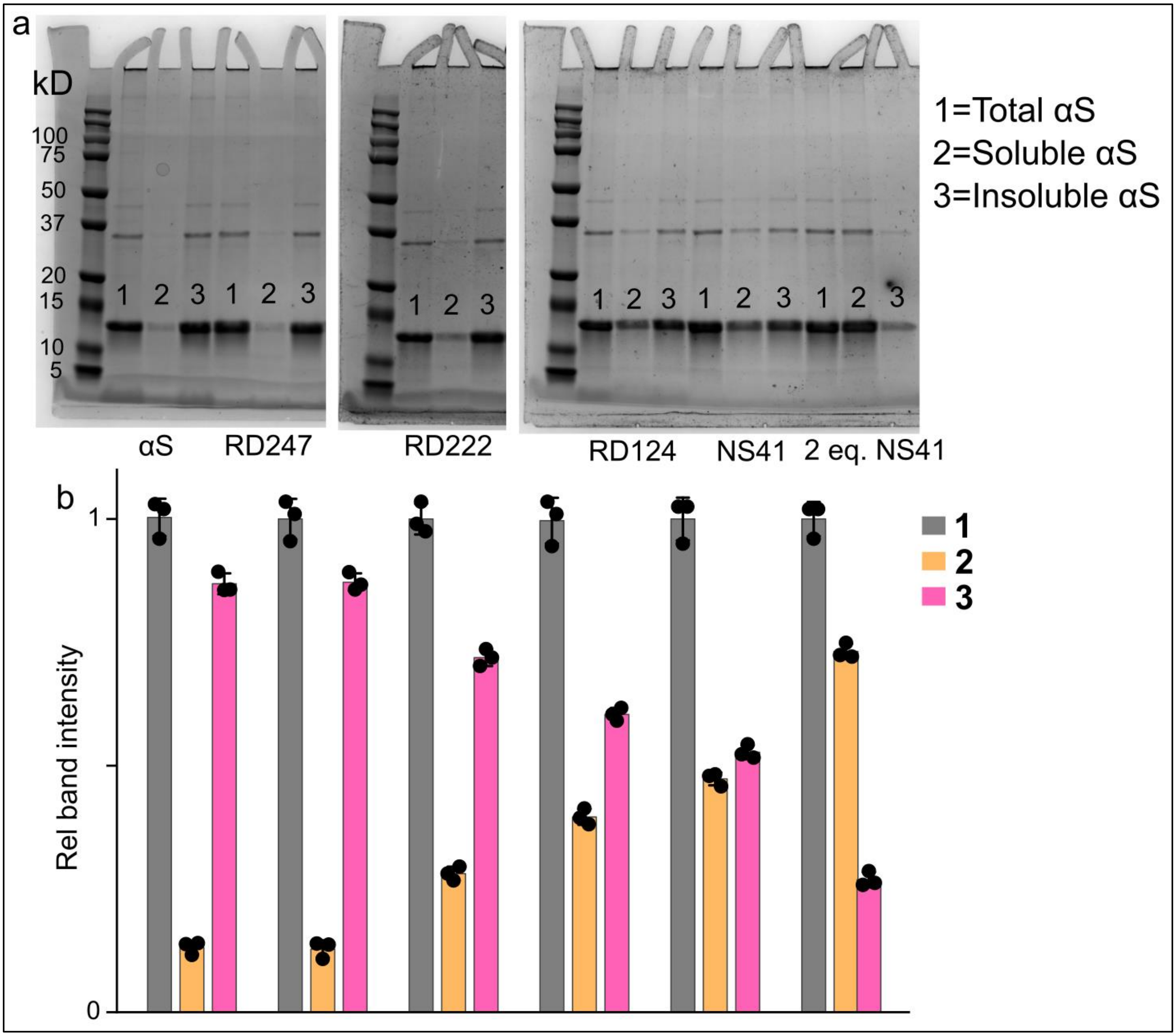

Supplementary Fig. 4. a, The representative SDS-PAGE gels of 100 μM αS aggregation for four days in the absence and presence of the indicated ligands at an equimolar ratio. **b**, The graphical representation of the SDS-PAGE gel analysis of 100 μM αS aggregation for four days in the absence and presence of the indicated ligands in 1 × PBS at an equimolar ratio. For NS41, the experiments were carried out at an equimolar ratio (NS41) and at a moral ratio of 2 (αS:NS41, 1:2), represented as 2 eq. of NS41. The data were expressed as mean and the error bars report the S.D. (n = 3 independent experiments).

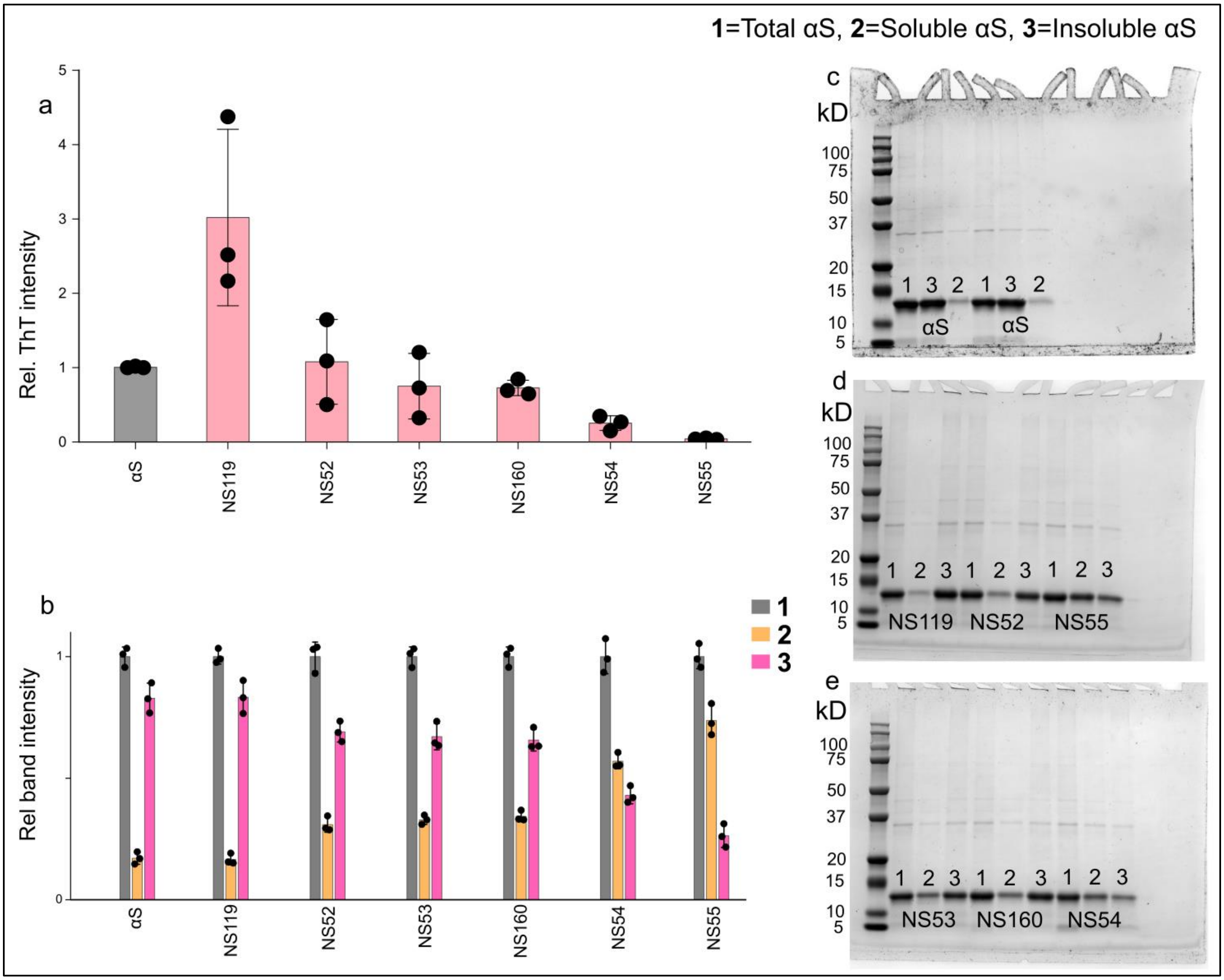

Supplementary Fig. 5. The graphical representation of the ThT intensity (**a**), SDS-PAGE gel analysis (**b**), and SDS-PAGE gels (**c-e**) of 100 μM αS aggregation for four days in the absence and presence of dipyridyls at an equimolar ratio. The data were expressed as mean and the error bars report the S.D. (n = 3 independent experiments).

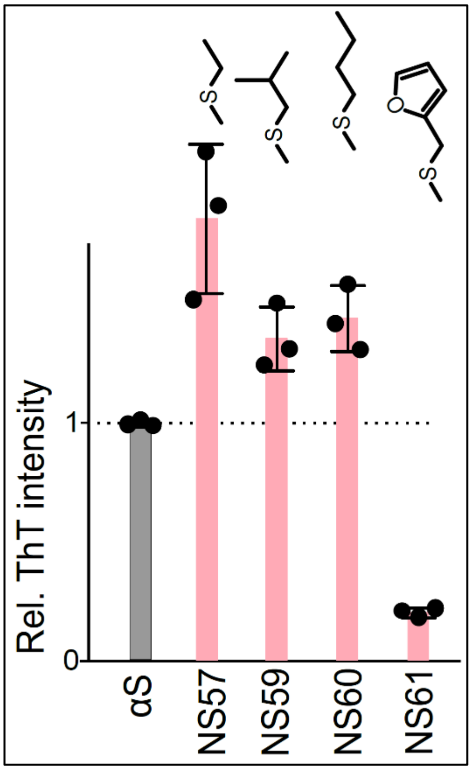

Supplementary Fig. 6. The graphical representation of the ThT intensity of 100 µM αS aggregation for four days in the absence and presence of the indicated dipyridyls at an equimolar ratio. The data were expressed as mean and the error bars report the S.D. (n = 3 independent experiments).

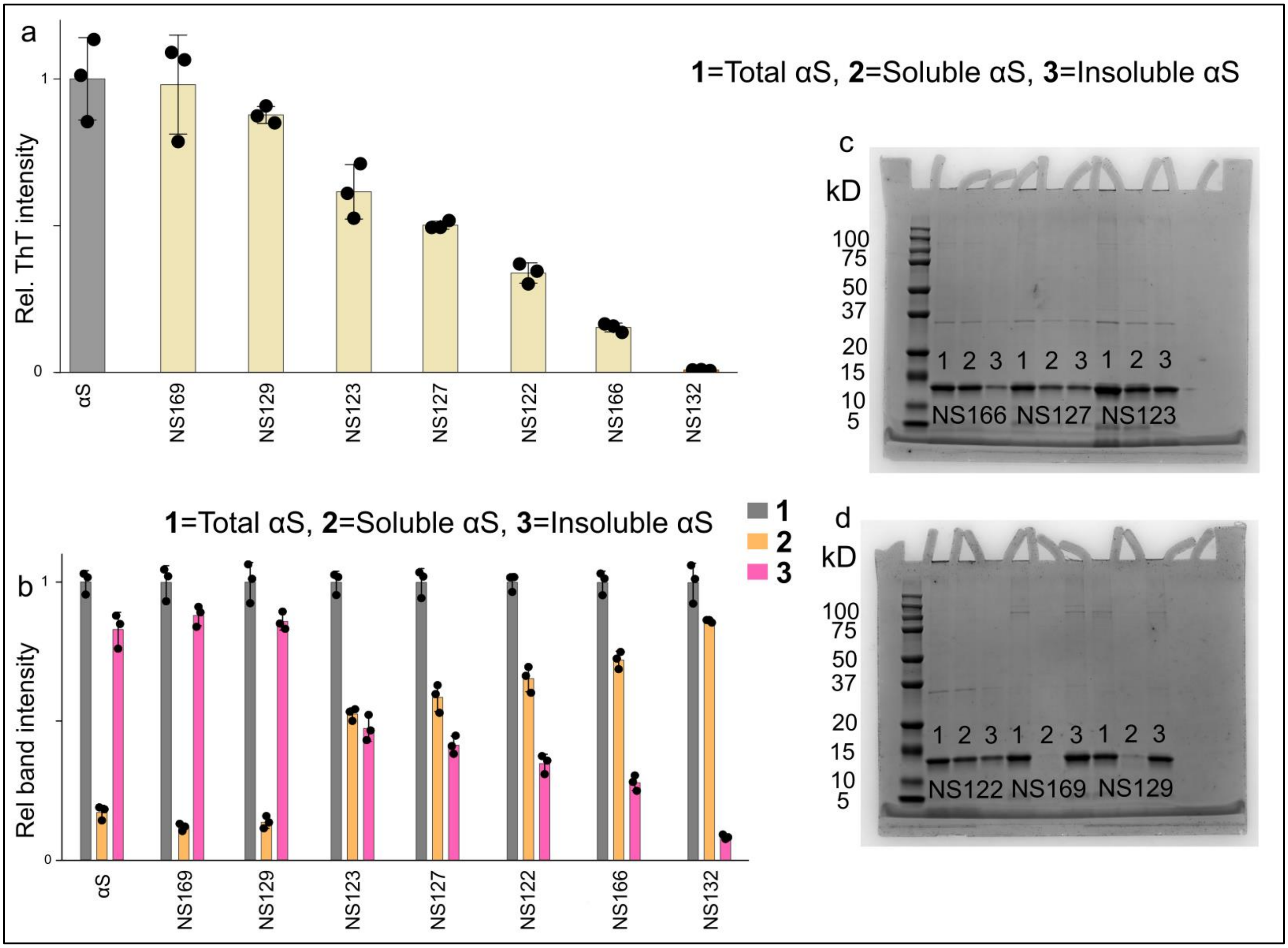

Supplementary Fig. 7. The graphical representation of the ThT intensity (**a**), SDS-PAGE gel analysis (**b**), and SDS-PAGE gels (**c,d**) of 100 μM αS aggregation for four days in the absence and presence of tripyridyls at an equimolar ratio. The data were expressed as mean and the error bars report the S.D. (n = 3 independent experiments).

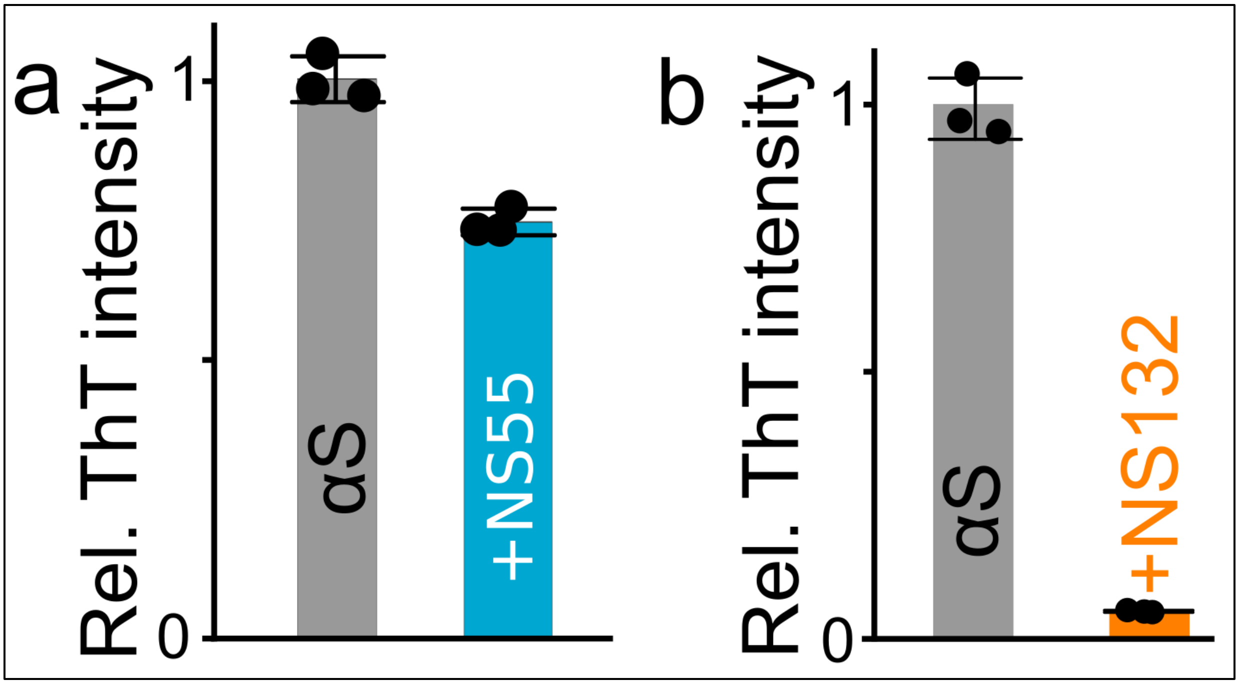

Supplementary Fig. 8. The graphical representation of the ThT intensity of 100 µM αS aggregation for four days in the absence and presence of NS55 (**a**) and NS132 (**b**) in 1 × PBS (pH 6.5) at substoichiometric ratio (for NS55, αS:ligand, 1:0.5, and for NS132, αS:ligand, 1:0.1,). **c**, Representative SDS-PAGE gel images and band intensities of 100 µM αS aggregation for four days in the absence and presence of NS132 (**c,d**) and NS132-P (**e,f**) at the indicated molar ratios. The ThT experiments were conducted three times and the reported change in the ThT intensity was an average of three separate experiments. The data were expressed as mean and the error bars report the S.D. (n = 3 independent experiments).

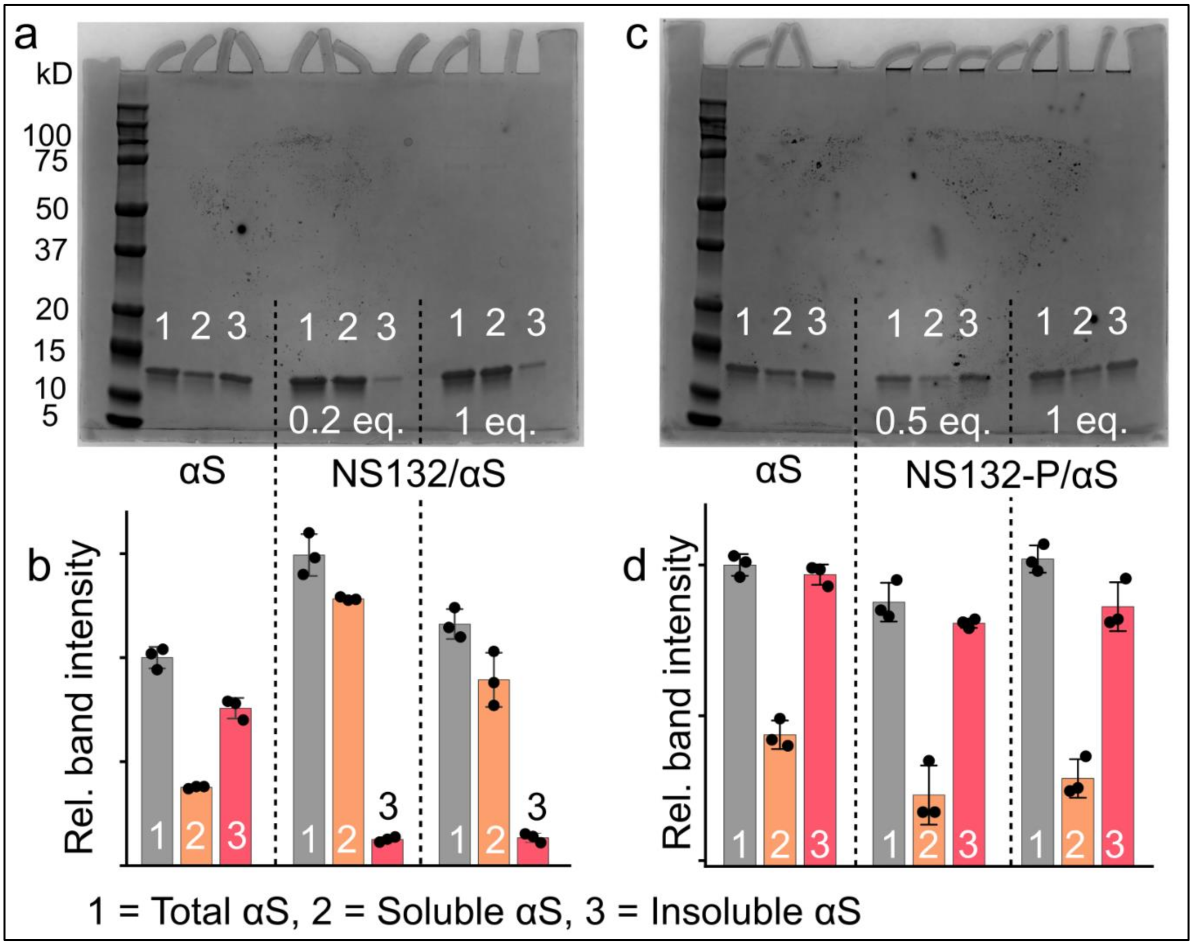

Supplementary Fig. 9. Representative SDS-PAGE gel images and band intensities of 100 μM αS aggregation for four days in the absence and presence of NS132 (**a,b**) and NS132-P (**c,d**) at the indicated molar ratios. The gel shift assay experiments were conducted three times and the reported intensity changes were an average of three separate experiments. The data were expressed as mean and the error bars report the S.D. (n = 3 independent experiments).

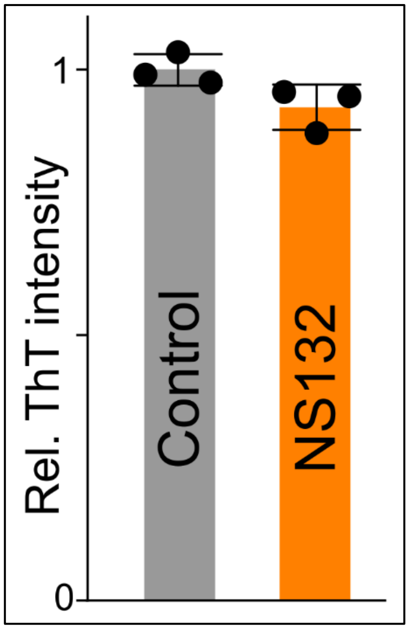

Supplementary Fig. 10. The comparison of the fluorescence intensity of the ThT dye (50 μM) in the absence and presence of NS132 (100 μM) in 1 × PBS buffer (pH 6.5). The ThT experiments were conducted three times and the reported change in the ThT intensity was an average of three separate experiments. The data were expressed as mean and the error bars report the s.d. (n = 3 independent experiments and each n consisted of three technical replicates).

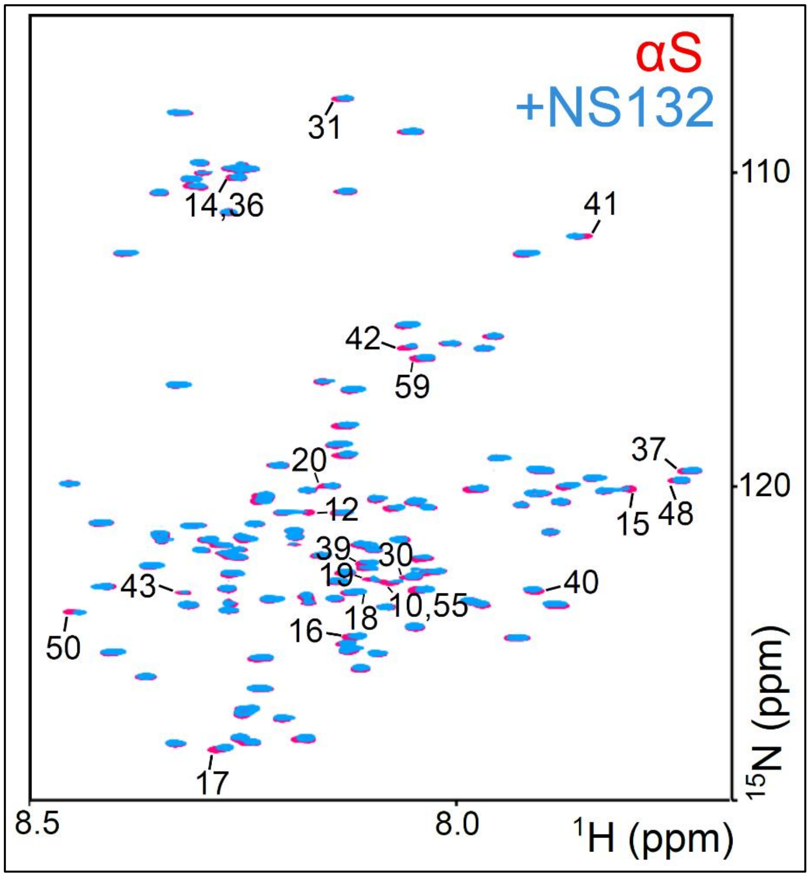

Supplementary Fig. 11. Overlay of 2D HSQC (^{1}H, ^{15}N) NMR spectra of 70 µM uniformly ^{15}N-labelled αS in the absence (red) and presence (blue) of NS132 at an equimolar ratio in 1 × PBS buffer (pH 6.5).

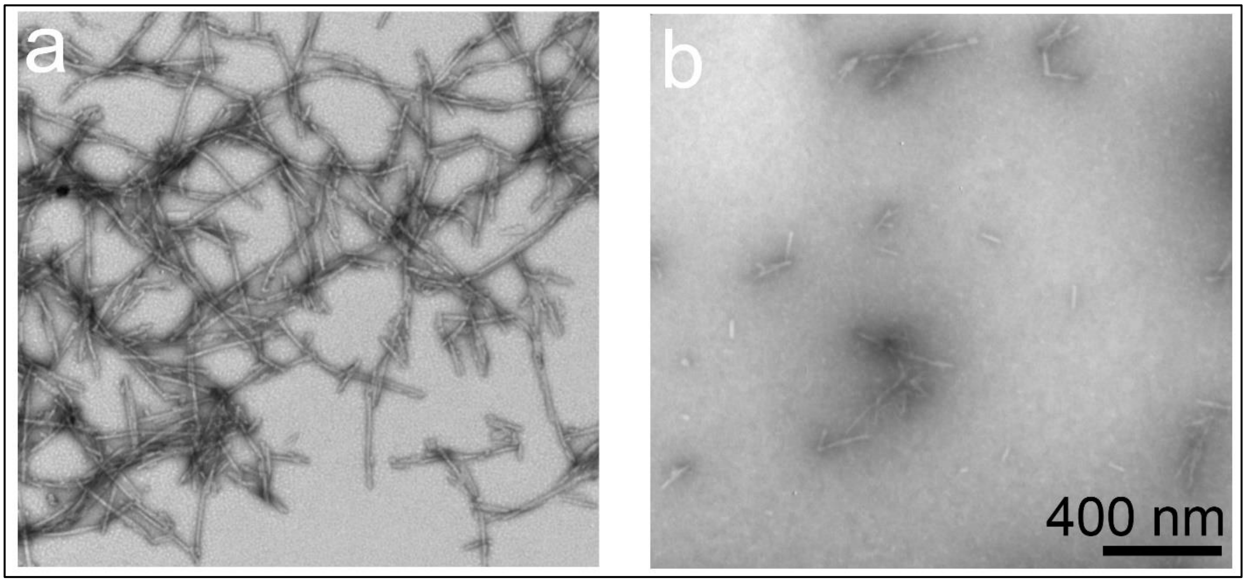

Supplementary Fig. 12. The TEM images of the preformed αS fibers (20%, monomer concentration) catalyzed aggregation of 100 μM αS in the absence (**a**) and presence (**b**) of NS132 at an equimolar ratio in 1 × PBS buffer (pH 6.5).

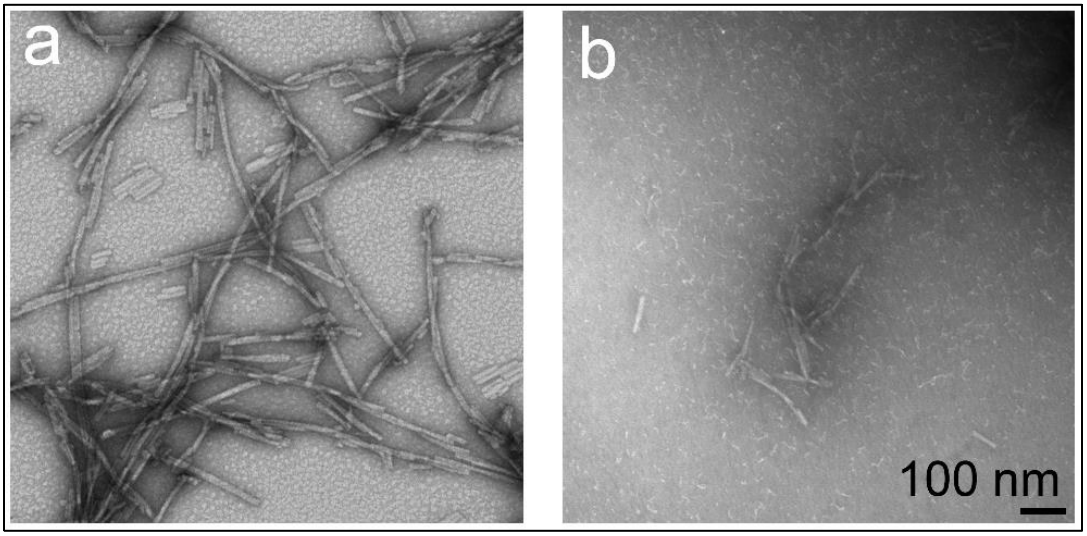

Supplementary Fig 13. The TEM images of the solution from cycle 5th of the PMCA experiment of αS in the absence (**a**) and presence (**b**) of NS132 at an equimolar ratio in 1 × PBS buffer (pH 6.5).

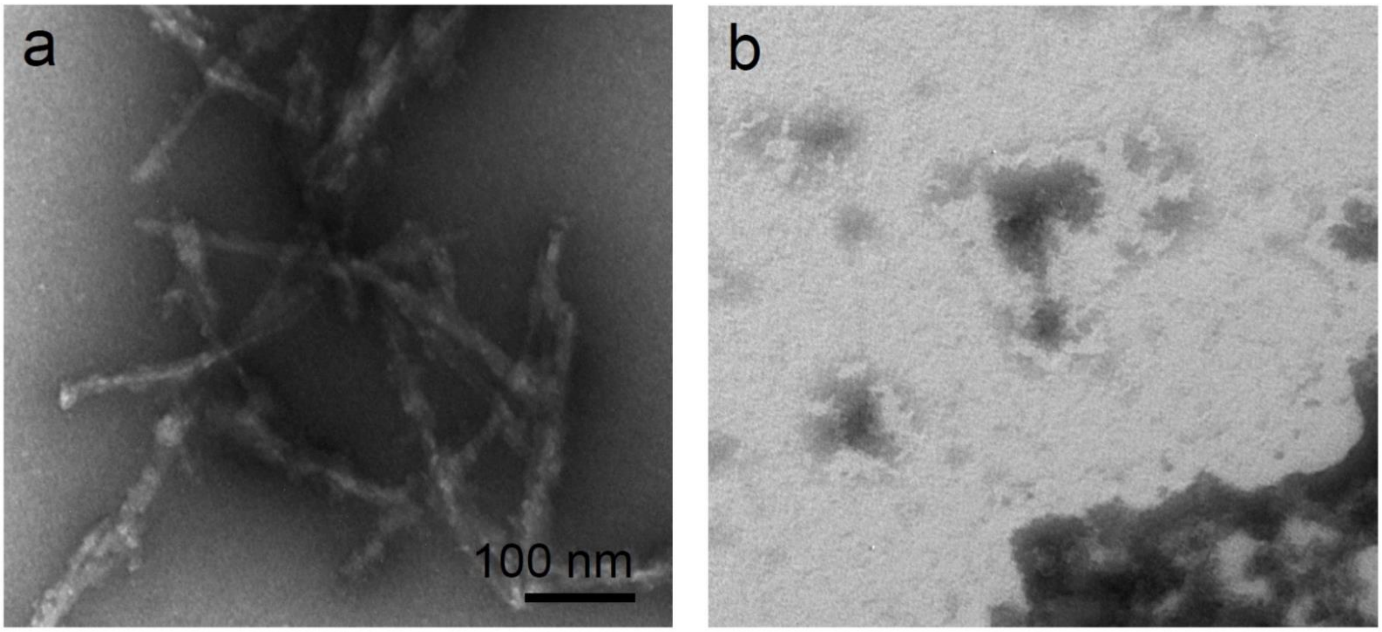

Supplementary Fig. 14. The TEM images of 100 μM αS solution aggregated for four days under aggregation conditions (1 × PBS buffer, pH 6.5) in the absence (a) and presence (b) of NS163 at an equimolar ratio.

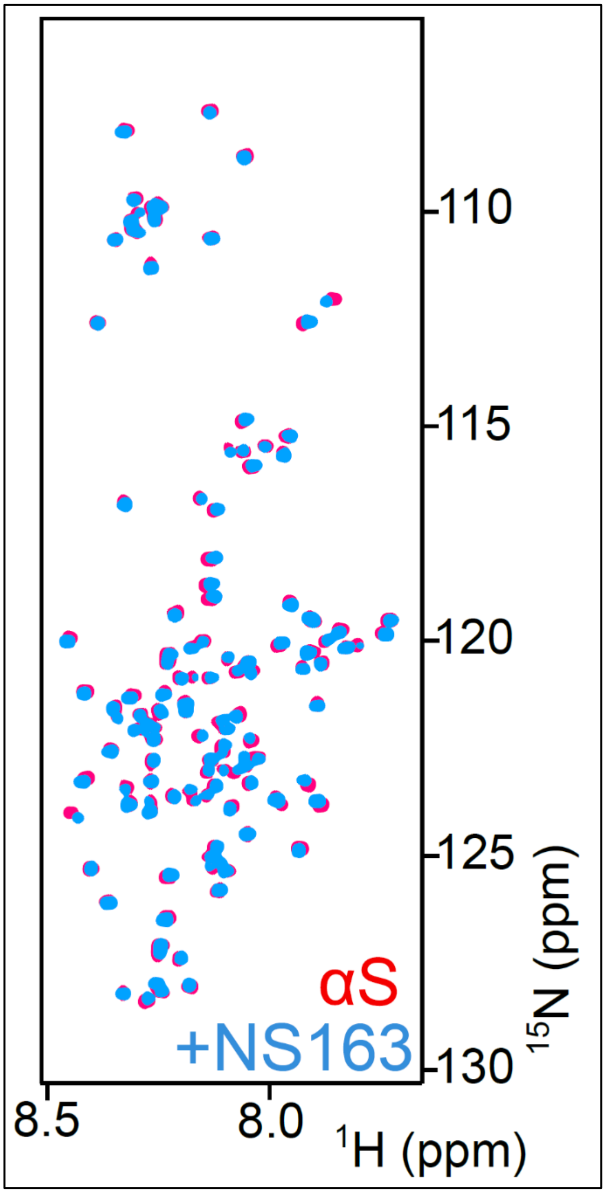

Supplementary Fig. 15. Overlay of 2D HSQC (^{1}H, ^{15}N) NMR spectra of 70 µM uniformly ^{15}N-labelled αS in the absence (red) and presence (blue) of NS163 at an equimolar ratio in 1 × PBS buffer (pH 6.5).

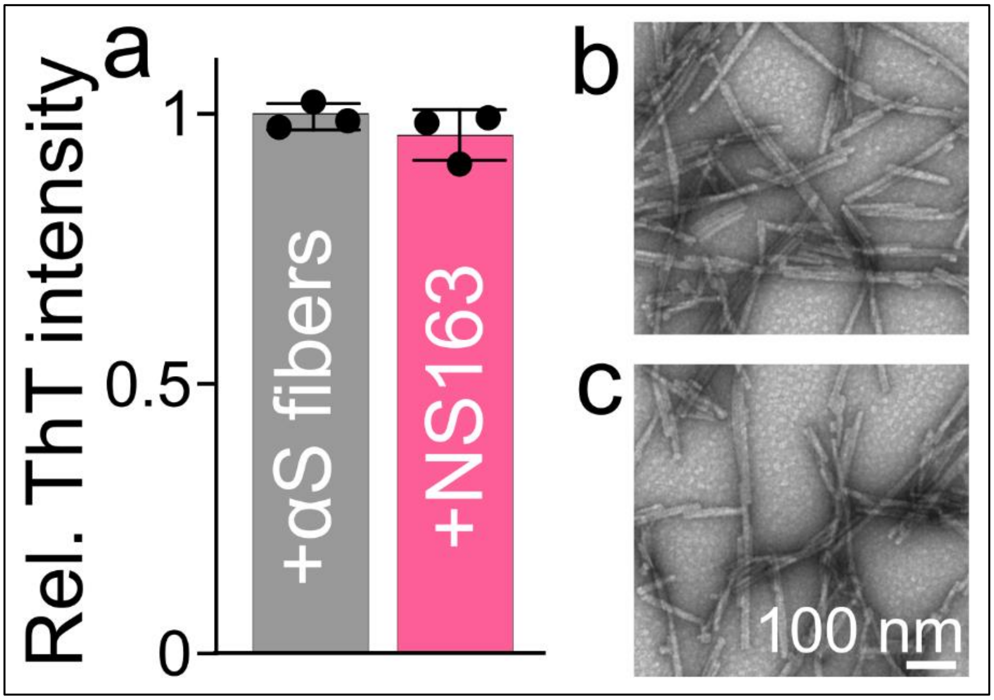

Supplementary Fig. 16. a, The comparison of the fluorescence intensity of ThT dye (50 μM) of preformed 100 μM αS fibers (1 × PBS buffer, pH 6.5) in the absence and presence of NS163 at an equimolar ratio. The representative TEM images of preformed 100 μM αS fibers (**b**) treated with NS163 for 24 h (**c**) at an equimolar ratio. The ThT and TEM experiments were conducted three times and the reported change in the ThT intensity was an average of three separate experiments. The data were expressed as mean and the error bars report the s.d. (n = 3 independent experiments).

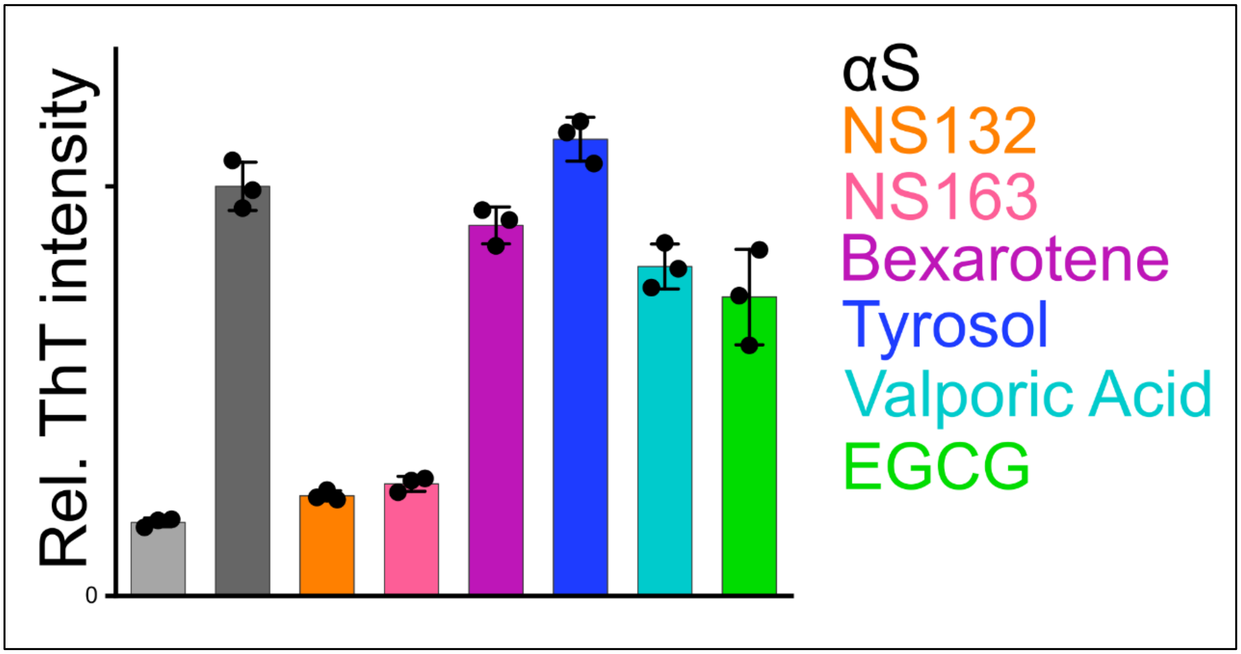

Supplementary Fig. 17. The graphical representation of the ThT intensity of 100 µM αS aggregation for four days in the absence and presence of the indicated ligands at an equimolar ratio. The aggregation assays were conducted three times and the reported change in the ThT intensity was an average of three independent experiments. The data were expressed as mean and the error bars report the s.d. (n = 3 independent experiments).

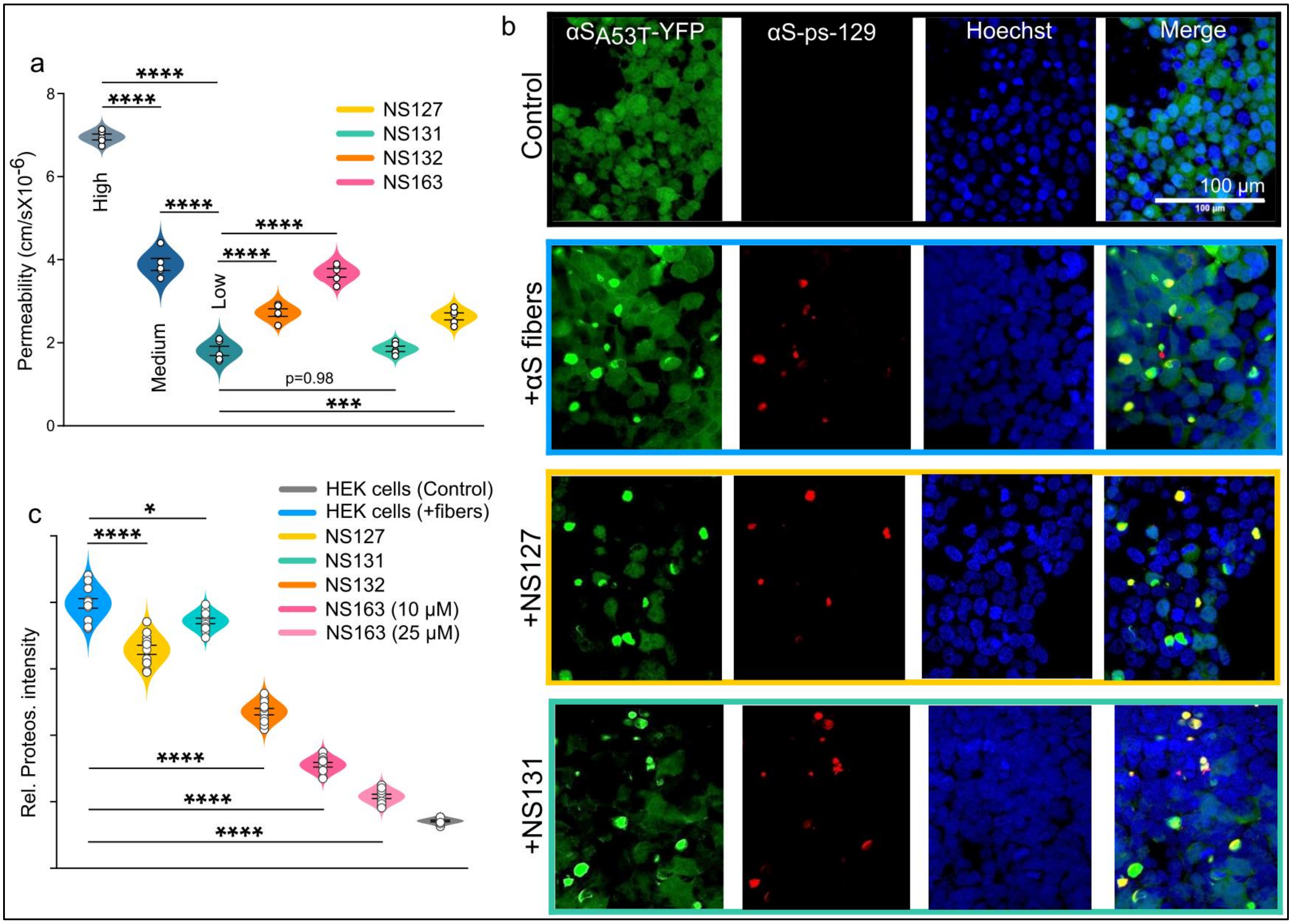

Supplementary Fig. 18. a, Assessment of cell permeability of the indicated ligands using the PAMPA. Confocal images (**b**) and statistical analysis (**c**) of HEK cells treated with the aggregated solution of 5 μM αS, followed by the treatment with the indicated ligands (10 μM). Inclusions of αS_{A53T}-YFP = white arrows, Hoechst = blue, αS-ps-129 = red, merge = Hoechst, αS-ps-129, and αS_{A53T}-YFP. The PAMPA assays were conducted three independent times with three technical replicates. The Proteostat assays were conducted with at least four biological replicates and three technical replicates for each biological replicate. The data were expressed as mean and the error bars report the s.e.m. (n = 3-4 independent experiments and each n consisted of three technical replicates). The statistical analysis was performed using ANOVA with Tukey's multiple comparison test. *$p < 0.05$, **$p < 0.01$, ***$p < 0.001$, ****$p < 0.0001$.

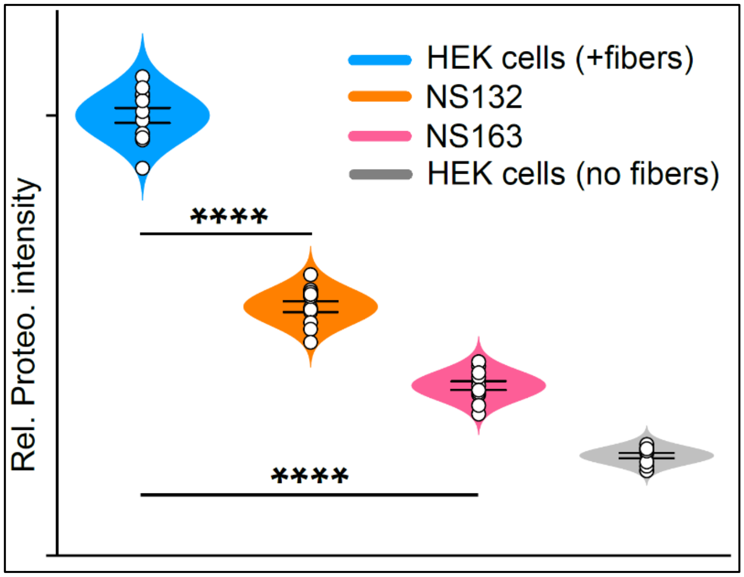

Supplementary Fig. 19. The relative intensity of Proteostat dye-stained intracellular inclusions of αS_{A53T}-YFP after treatment with the indicated ligands (10 µM) followed by the treatment with αS fibers (5 µM monomer conc.) for 24 h. The Proteostat assays were conducted with at least four biological replicates and three technical replicates for each biological replicate. The data were expressed as mean and the error bars report the s.e.m. (n = 4 independent experiments and each n consisted of three technical replicates). The statistical analysis was performed using ANOVA with Tukey's multiple comparison test. *$p < 0.05$, **$p < 0.01$, ***$p < 0.001$, ****$p < 0.0001$.

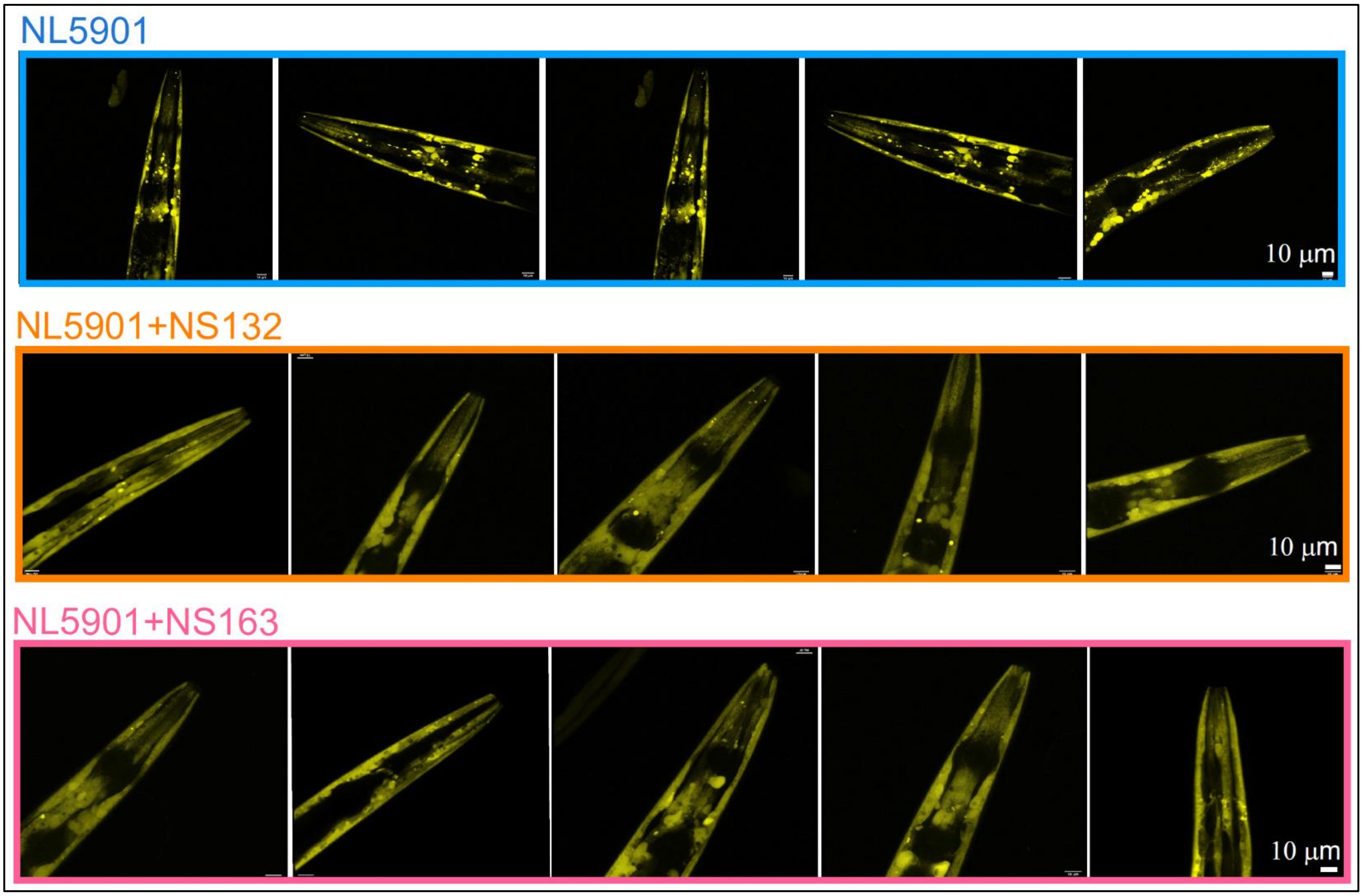

Supplementary Fig. 20. The representative confocal images of αS-YFP inclusions in the body wall muscle cells of NL5901 (Days = 8) in the absence and presence of 50 μM NS163 and 50 μM NS132 (treated on day two and four). These confocal images were collected from at least three independent experiments with random selection of the worms from each independent experiment.

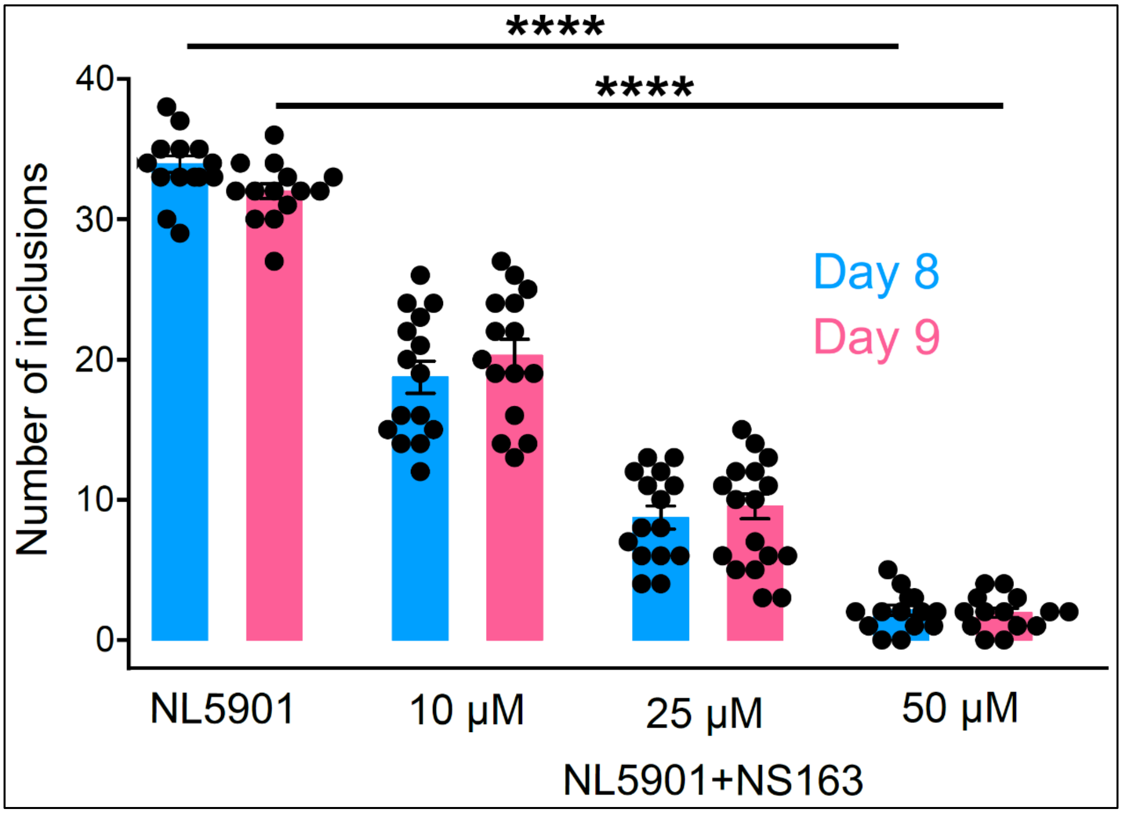

Supplementary Fig. 21. The comparison of the number of intracellular αS inclusions in NL5901 worms in the absence and presence of the indicated doses of NS163 on day eight (blue bar) and nine (red bar). For each confocal imaging experiment, at least 5 worms were used and the inclusions were counted manually, and each condition (each day) consisted of three independent experiments. The data were expressed as mean and the error bars report the s.e.m. (n = 3 independent experiments and each n consisted of a minimum of 5 technical replicates). The statistical analysis was performed using ANOVA with Tukey's multiple comparison test. *$p < 0.05$, **$p < 0.01$, ***$p < 0.001$, ****$p < 0.0001$.

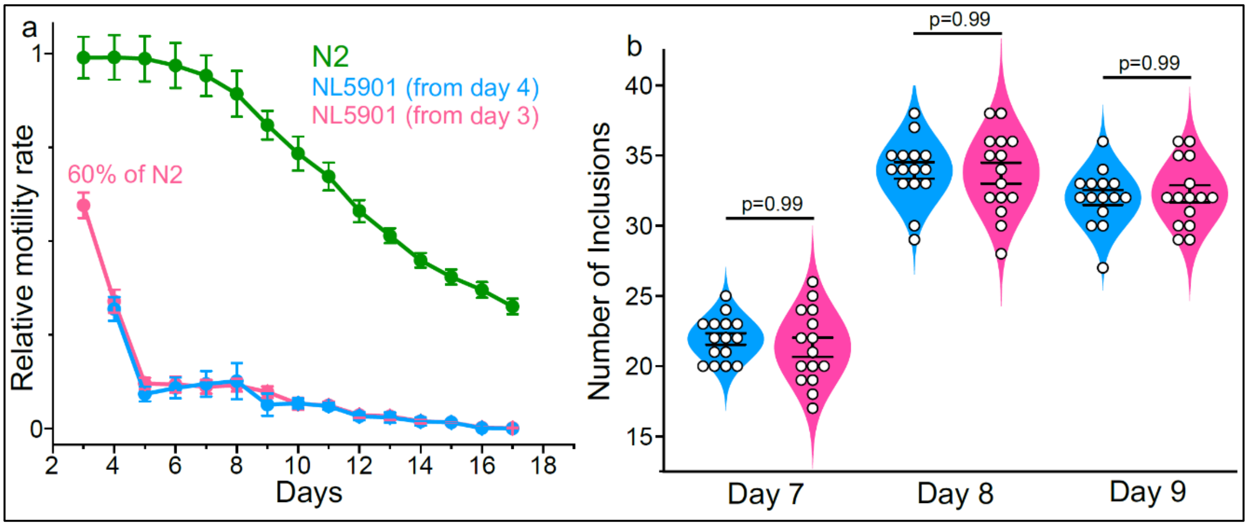

Supplementary Fig. 22. a, The comparison of the motility of N2 and NL5901 worms from day three (blue line) and day four (red line). **b**, The number of αS inclusions in NL5901 worms from day seven to day nine for NL5901 worms from experiments in **a**. For each confocal imaging experiment, at least 5 worms were used, and the inclusions were counted manually. Each condition (Each day) consisted of at least three independent experiments. For motility experiments, a total of 50 worms were used in duplicate for each experiment and each condition consisted of four independent experiments. The data were expressed as mean and the error bars report the s.e.m. (n = 3 or 4 independent experiments and each n consisted of at least three technical replicates). The statistical analysis was performed using ANOVA with Tukey's multiple comparison test.

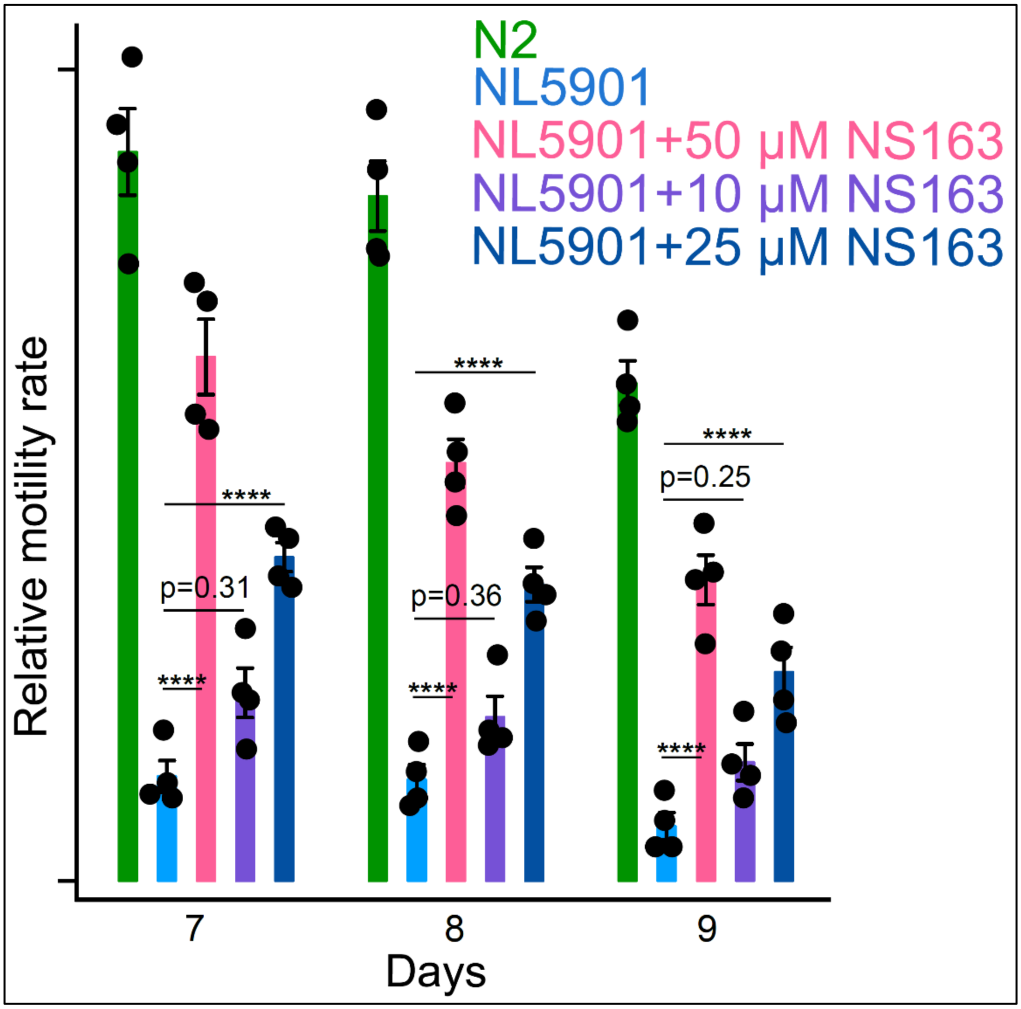

Supplementary Fig. 23. The statistics for the relative motility rate of N2 and NL5901 in the absence and presence of various doses of NS163 on the indicated days. For motility experiments, a total of 50 worms were used in duplicate for each experiment and each condition consisted of at least four independent experiments. The data were expressed as mean and the error bars report the s.e.m. (n = 4 independent experiments and each n consisted of a minimum of two technical replicates). The statistical analysis was performed using ANOVA with Tukey's multiple comparison test. *p < 0.05, **p < 0.01, ***p < 0.001, ****p < 0.0001.

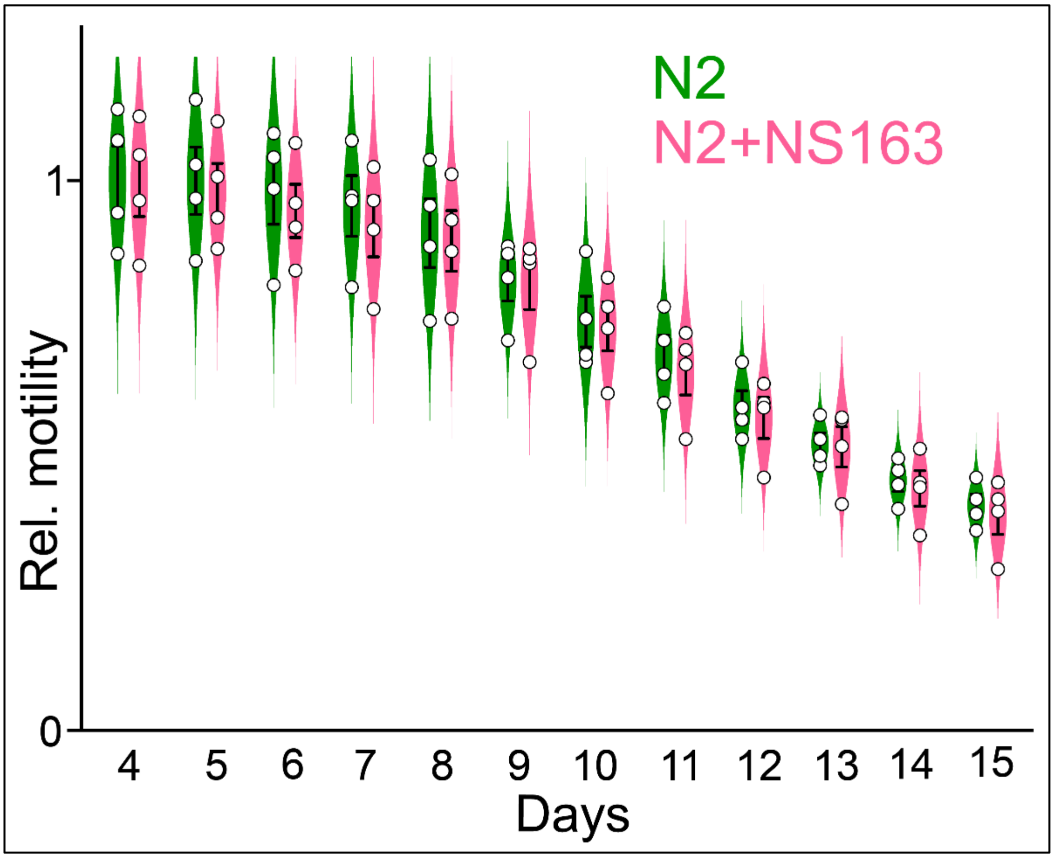

Supplementary Fig. 24. The effect of NS163 on the motility of N2 worms. The comparison of the motility rate of N2 in the absence and presence of 50 µM NS163 (treatment on day two and four). For motility rate experiment, a total of 50 worms were used in duplicate for each experiment and each condition consisted of four independent experiments. The data were expressed as mean and the error bars report the s.e.m. ($n = 4$ independent experiments and each n consisted of two technical replicates). The statistical analysis was performed using ANOVA with Tukey's multiple comparison test. *$p < 0.05$, **$p < 0.01$, ***$p < 0.001$.

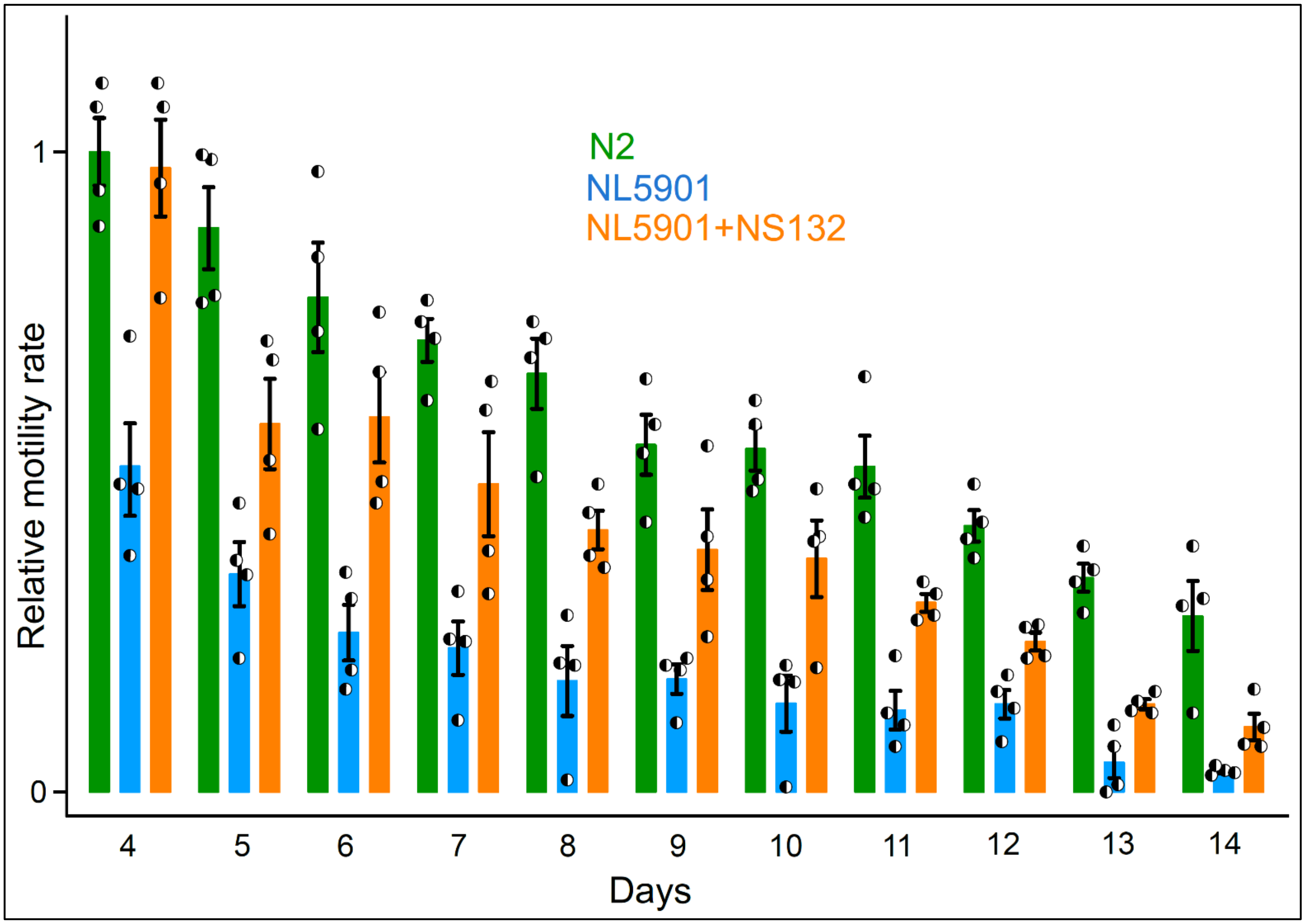

Supplementary Fig. 25. The intracellular inhibition of αS aggregation by NS132 in an *in vivo* PD model. The comparison of the motility rate of N2 and NL5901 and statistics in the absence and presence of 50 µM NS132 (treatment on day two and four). For motility rate experiment, a total of 50 worms were used in duplicate for each experiment and each condition consisted of four independent experiments. The data were expressed as mean and the error bars report the s.e.m. (n = 4 independent experiments and each n consisted of two technical replicates). The statistical analysis was performed using ANOVA with Tukey's multiple comparison test. $*p < 0.05$, $**p < 0.01$, $***p < 0.001$.

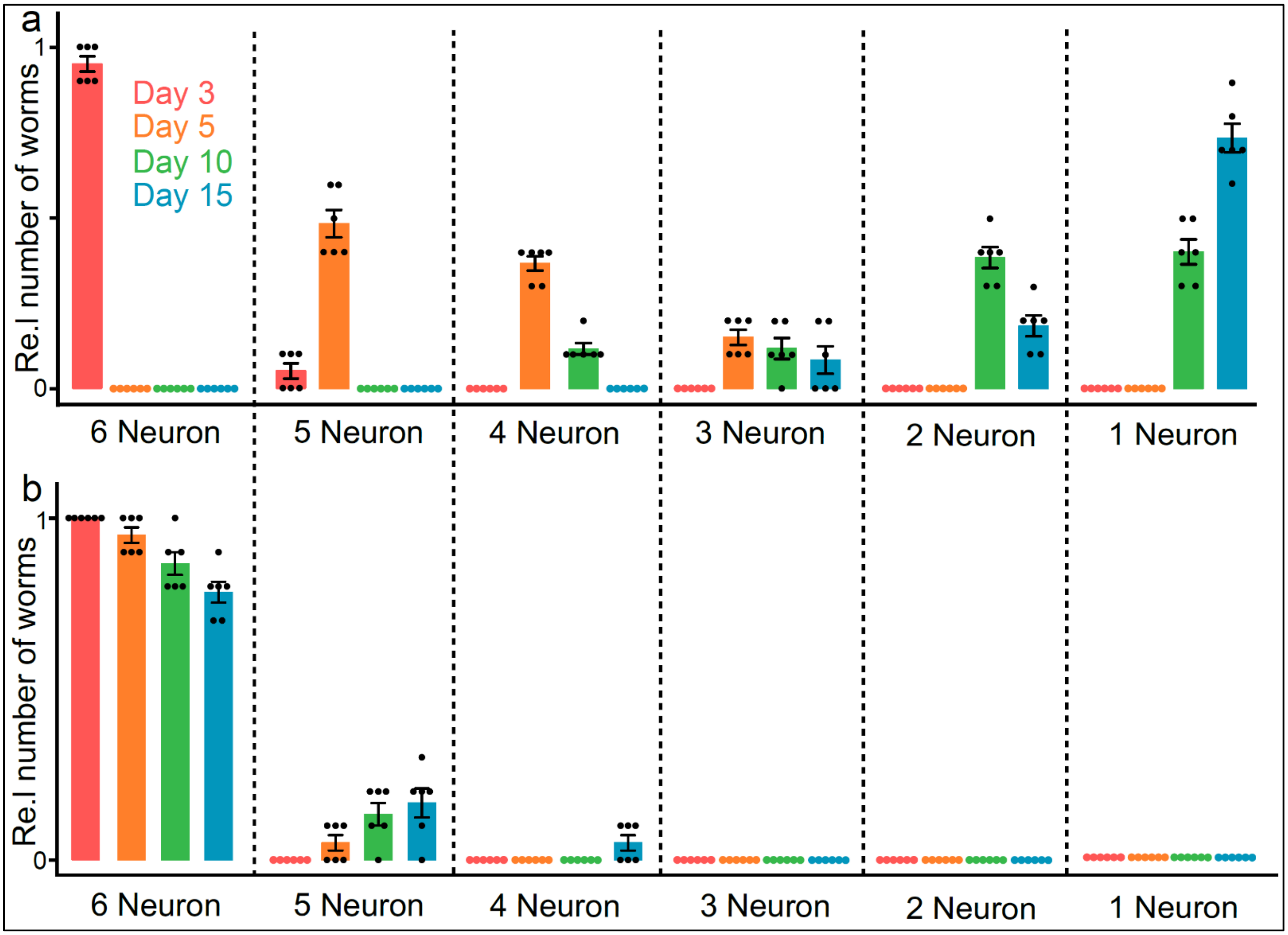

Supplementary Fig. 26. The relative number of healthy neurons in UA196 worms during the aging process in the absence (**a**) and presence (**b**) of 50 µM NS163 (treated on day two and four) on the indicated days. The number of DA neurons were determined using the confocal imaging. For each confocal imaging experiment, 10 worms were used, and the healthy neurons were counted manually, and each condition (day) consisted of six independent experiments with freshly bleached worms. The data were expressed as mean and the error bars report the s.e.m. (n = 6 independent experiments and each experiment consisted of 10 technical replicates).

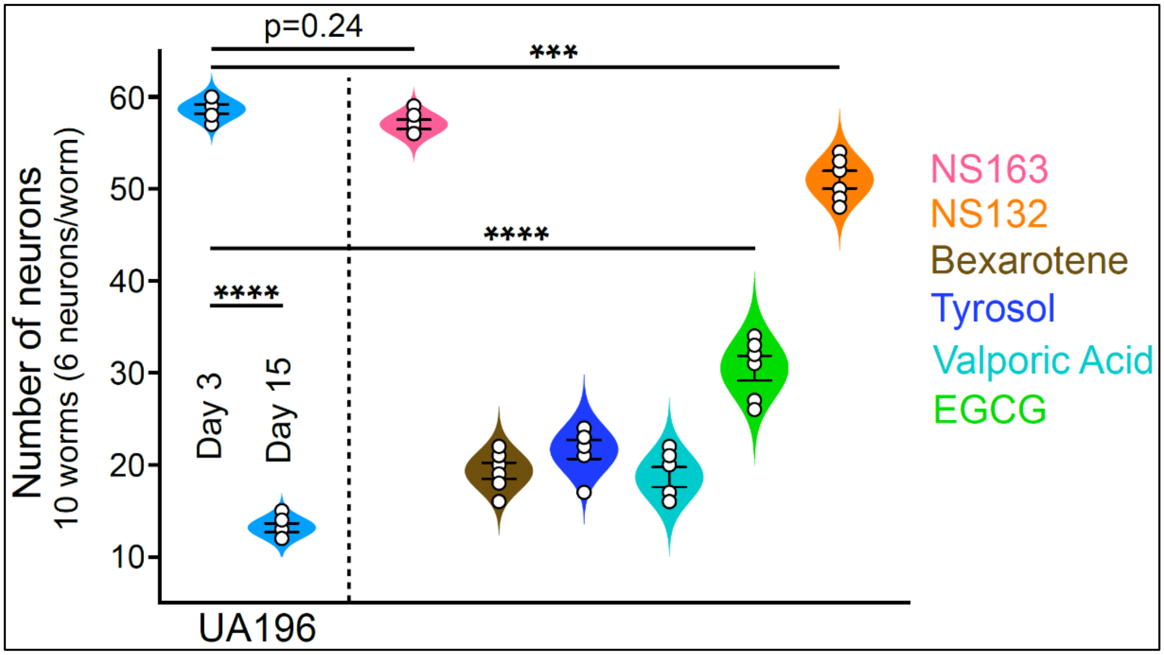

Supplementary Fig. 27. Neuroprotective effect of various ligands on the degeneration of DA neurons. Statistics for the total number of neurons in UA196 worms on the indicated days and the effect of the indicated ligands (50 µM, treatment on day two and four) on the DA neurons on day 15. The number of DA neurons were determined using the confocal imaging. For each confocal imaging experiment, 10 worms were used, and the healthy neurons were counted manually, and each condition (day) consisted of six independent experiments with freshly bleached worms. The data were expressed as mean and the error bars report the s.e.m. ($n = 6$ independent experiments and each experiment consisted of 10 technical replicates). The statistical analysis was performed using ANOVA with Tukey's multiple comparison test. *$p < 0.05$, **$p < 0.01$, ***$p < 0.001$, ****$p < 0.0001$.

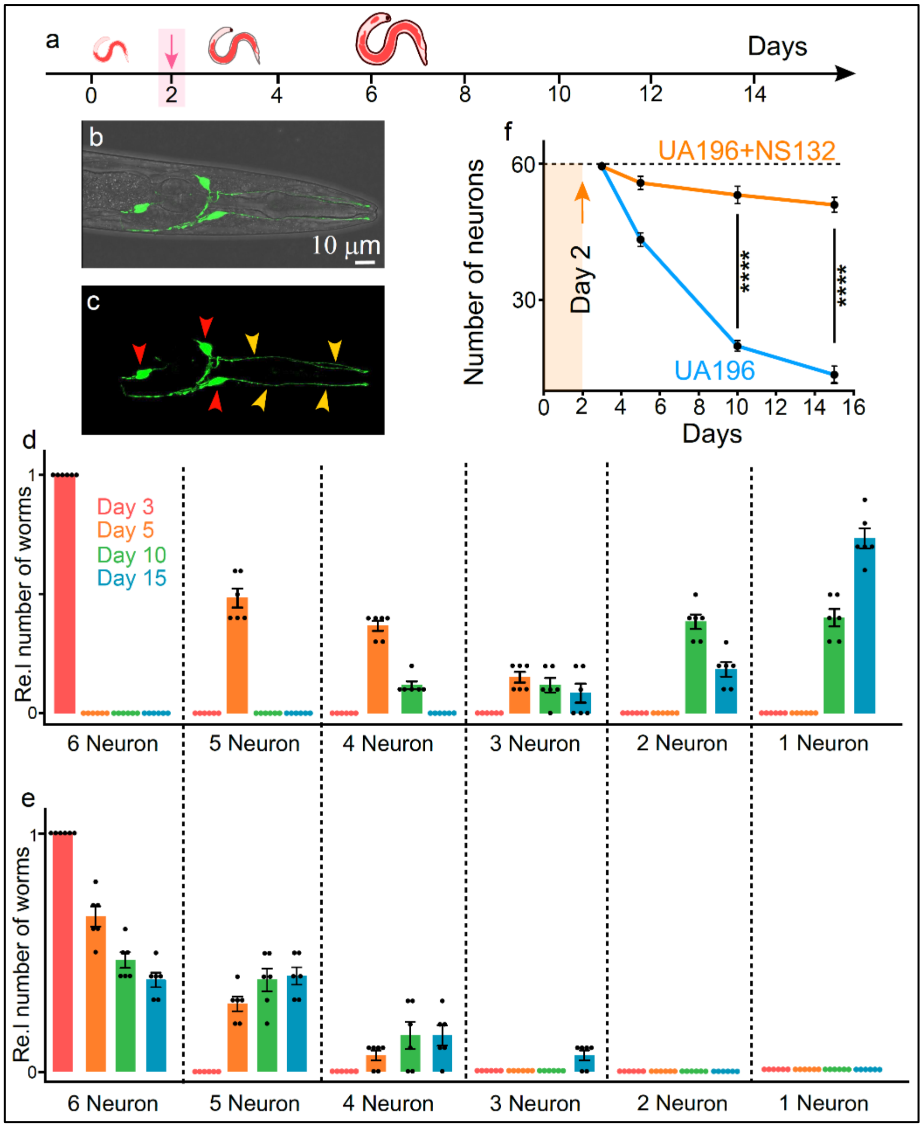

Supplementary Fig. 28. Neuroprotective effect of NS132 on the degeneration of DA neurons. a, Schematic of the aging process of UA196 worms and their treatment with the ligands. Representative confocal images of UA196 worms in the presence of 50 µM NS132 (treatment on day two and four) on day 15 (**b,c**). The healthy DA neurons (red arrow) and neurites (yellow arrow) in UA196 worms on day 15. The relative number of neurons in UA196 worms during the aging process in the absence (**d**) and presence of 50 µM NS132 (**e**). **f**, Statistics for the total number of neurons in UA196 worms during the aging process in the absence (blue) and presence (orange) of 50 µM NS132. For each confocal imaging experiment (**b,c**), at least 10 worms were used, and the healthy neurons were counted manually, and each condition (day) consisted of six independent experiments with freshly bleached worms. The data were expressed as mean and the error bars report the s.e.m. (n = 6 independent experiments and each experiment consisted of 10 technical replicates). The statistical analysis was performed using ANOVA with Tukey's multiple comparison test. $*p < 0.05$, $**p < 0.01$, $***p < 0.001$, $****p < 0.0001$.

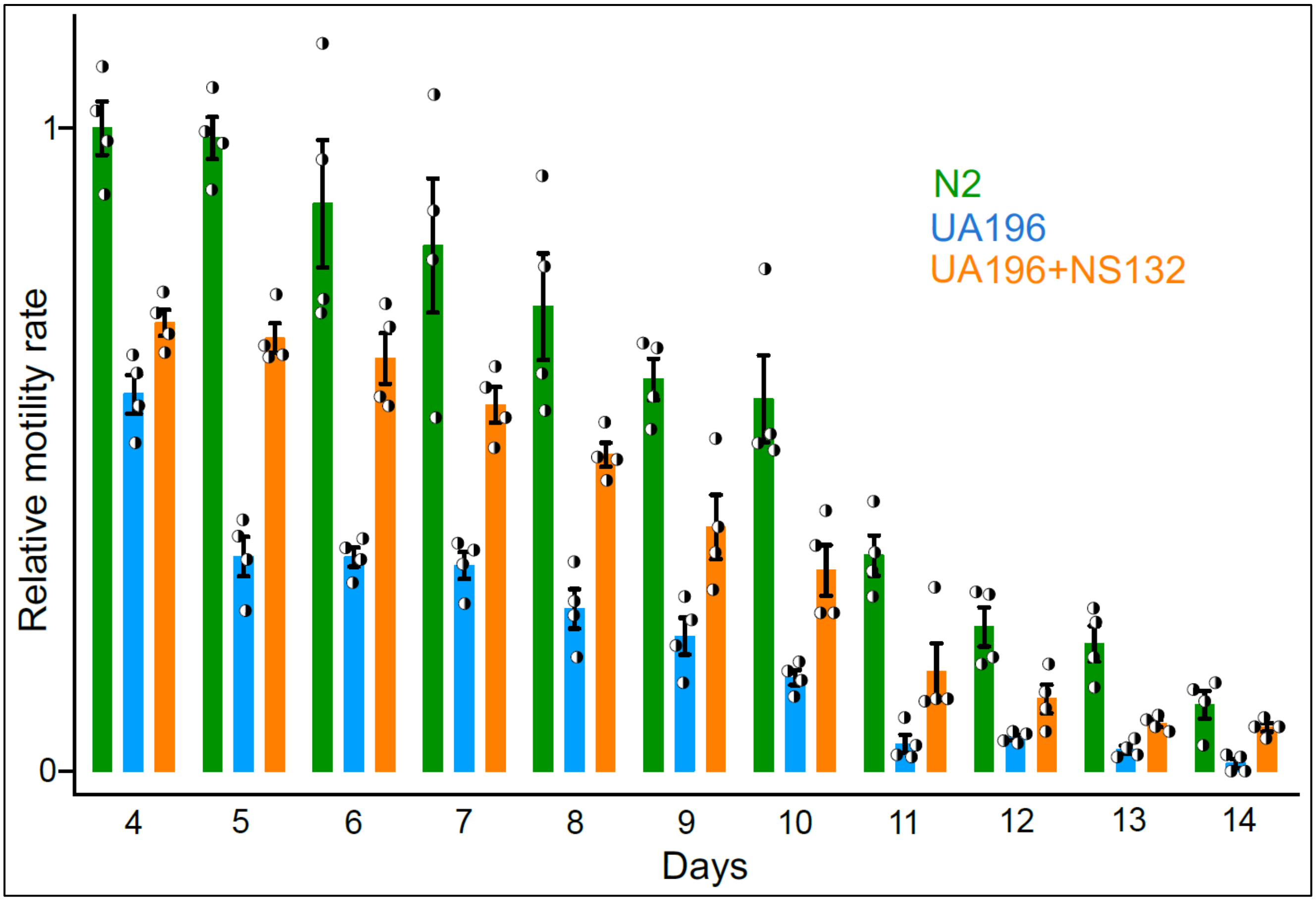

Supplementary Fig. 29. The rescue of motility rate in UA196 worms by NS132. The comparison of the motility rate of N2 (green bar) and UA196 in the absence (blue bar) and presence (orange bar) of 50 µM NS132 (treatment on day two and four). For motility rate experiment, a total of 50 worms were used in duplicate for each experiment and each condition consisted of four independent experiments. The data were expressed as mean and the error bars report the s.e.m. (n = 4 independent experiments and each n consisted of two technical replicates). The statistical analysis was performed using ANOVA with Tukey's multiple comparison test. $*p < 0.05$, $**p < 0.01$, $***p < 0.001$.

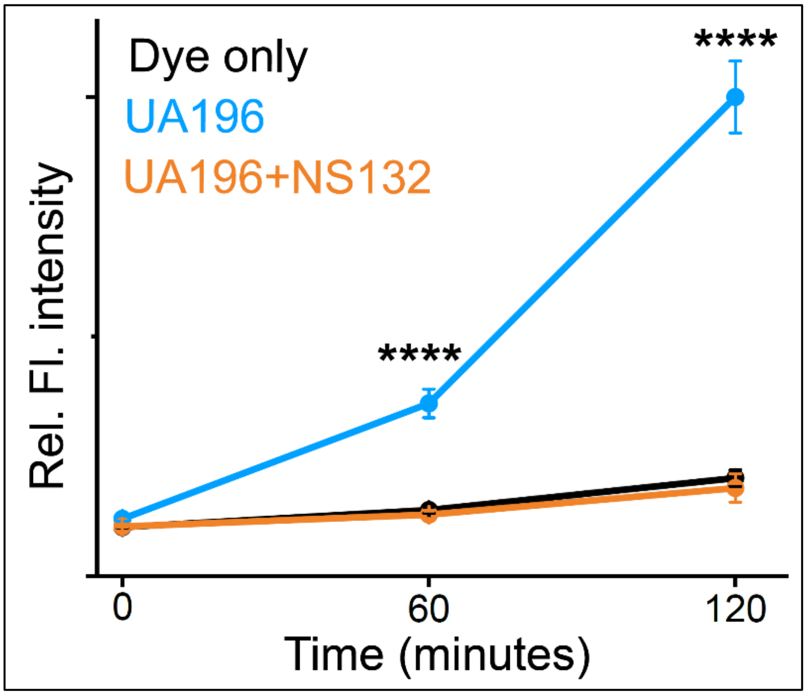

Supplementary Fig. 30. The effect of NS132 on the ROS level in UA196 worms. The comparison of the ROS level in UA196 worms in the absence and presence of 50 µM NS132 (treatment on day two and four)) at the indicated time points. The UA196 worms were treated with NS132 on day two and four and the ROS level was measured on day eight. For ROS level quantification, at least 50 worms were used and each condition consisted of three independent experiments with freshly bleached worms. The data were expressed as mean and the error bars report the s.d. (n = 3 independent experiments and each n consisted of three technical replicates). The statistical analysis was performed using ANOVA with Tukey's multiple comparison test. *$p < 0.05$, **$p < 0.01$, ***$p < 0.001$, ****$p < 0.0001$.

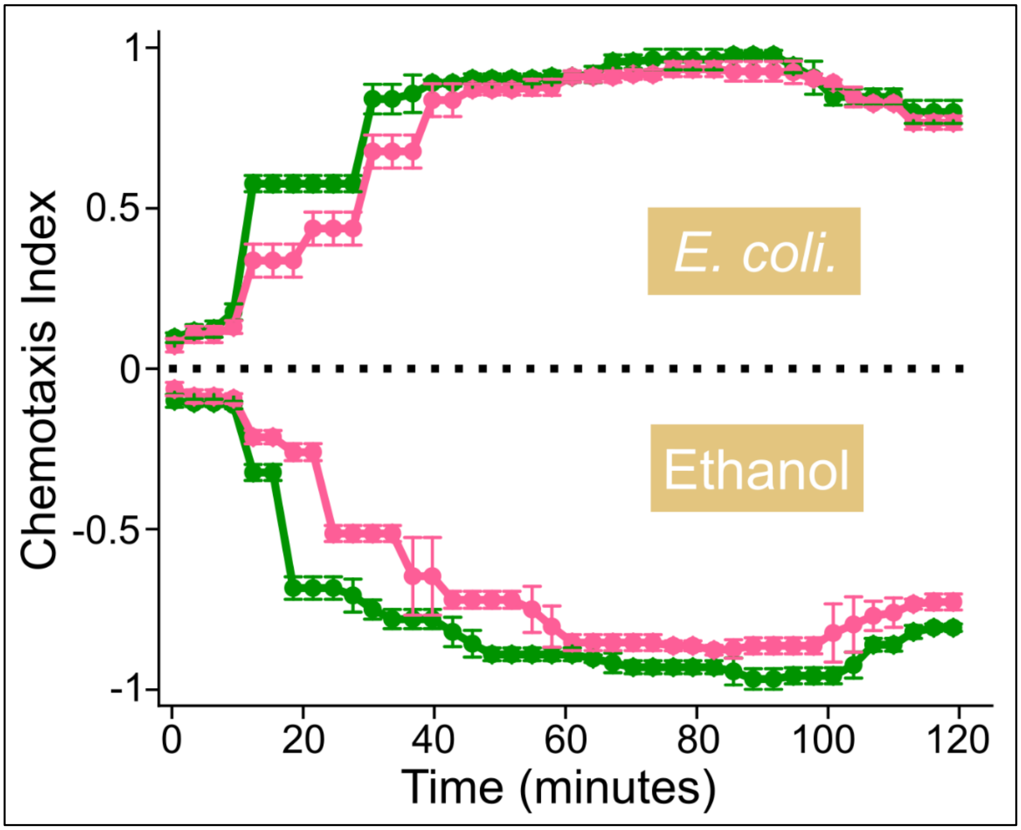

Supplementary Fig. 31. The CI graph for N2 worms in the absence (green bar) and presence (red bar) of 50 µM NS163 (treatment on day two and four) under the indicated conditions on day 10. For chemotaxis assays, a total of 50 worms were used in duplicate for each experiment and each condition consisted of three independent experiments with freshly bleached worms. The data were expressed as mean and the error bars report the s.e.m. (n = 3 independent experiments and each n consisted of two technical replicates).

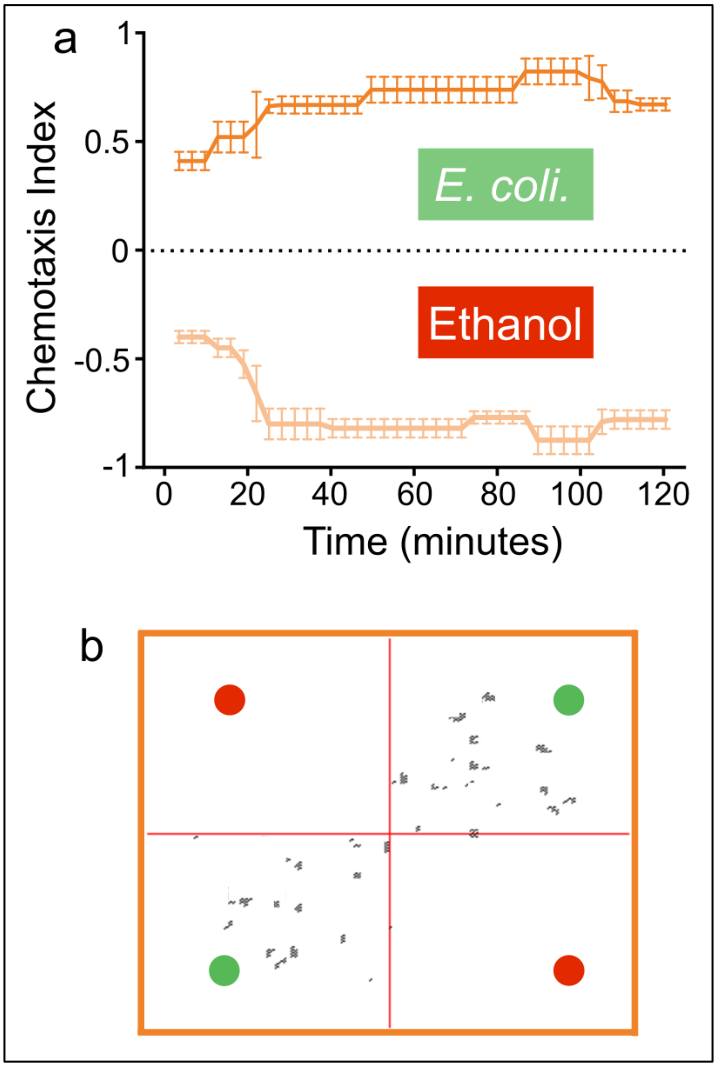

Supplementary Fig. 32. The effect of NS132 on the behavioral deficits mediated by αS aggregation in DA neurons in UA196 worms. a, The CI graph for UA196 worms treated with 50 µM NS132 (treatment on day two and four) under the indicated conditions on day 10. **b**, The snapshots at 60 min. of the animated videos (Supplementary Movie 13, 14) collected for the CI for UA196+NS132 under the indicated conditions. For chemotaxis assays, a total of 50 worms were used in duplicate for each experiment and each condition consisted of three independent experiments with freshly bleached worms. The data were expressed as mean and the error bars report the s.e.m. (n = 3 independent experiments and each n consisted of two technical replicates).

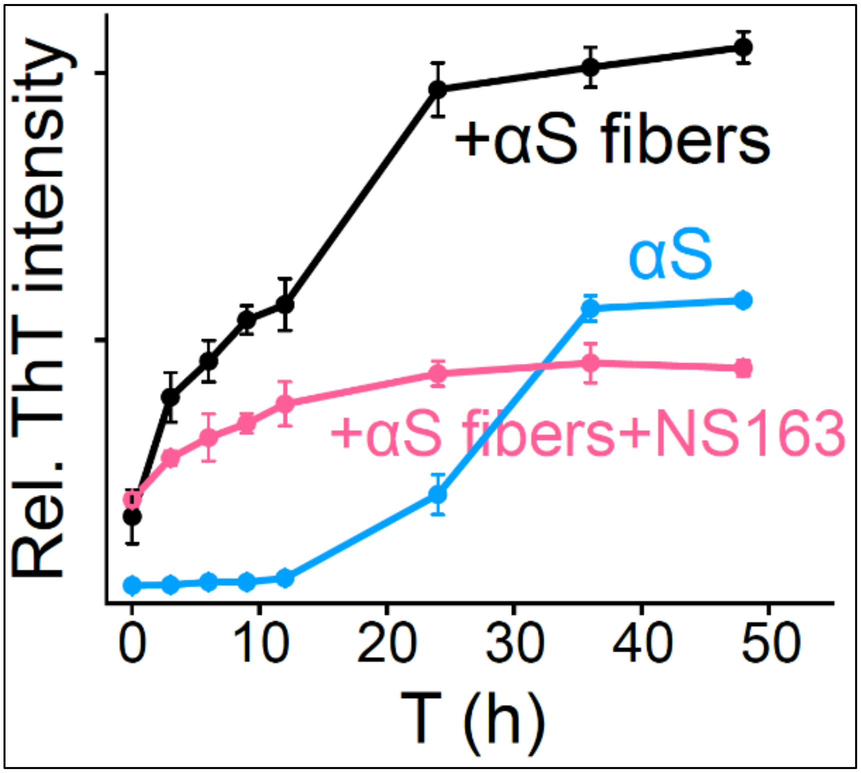

Supplementary Fig. 33. Effect of NS163 on the seed catalyzed aggregation of αS. The aggregation kinetic profile of the aggregation of αS monomer (100 μM, blue line), αS monomer (100 μM) + αS fibers (20% monomer, black line), and αS monomer (100 μM) + αS fibers (20% monomer) + NS163 (100 μM, red line) in the aggregation buffer conditions. The aggregation experiments were conducted three times and the reported change in the ThT intensity was an average of three independent experiments. The data were expressed as mean and the error bars report the s.d. (n = 3 independent experiments).

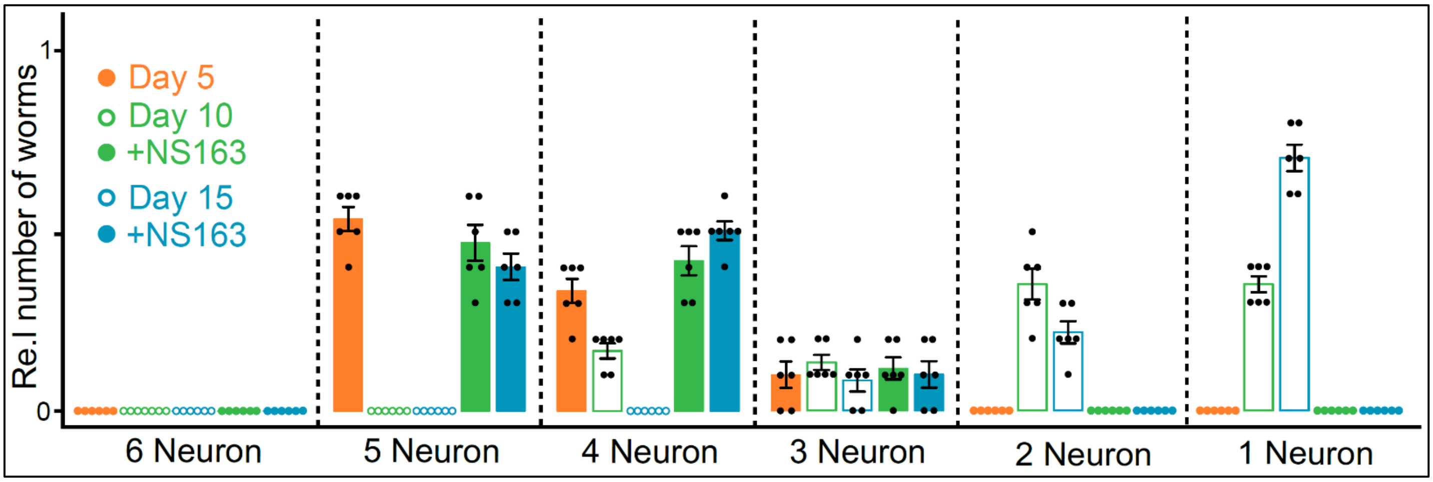

Supplementary Fig. 34. Neuroprotective effect of NS163 on the preexiting PD *C. elegans* model. The relative number of healthy DA neurons in UA196 worms during the aging process in the absence (Open bar and open circle) and when treated with 50 µM NS163 (Closed bar and closed circle) on day 5. The relative number of healthy DA neurons in UA196 worms on day 5 were presented with orange bar. The data were expressed as mean and the error bars report the s.e.m. (n = 6 independent experiments and each n consisted of 10 technical replicates). The statistical analysis was performed using ANOVA with Tukey's multiple comparison test. *p < 0.05, **p < 0.01, ***p < 0.001, ****p < 0.0001.

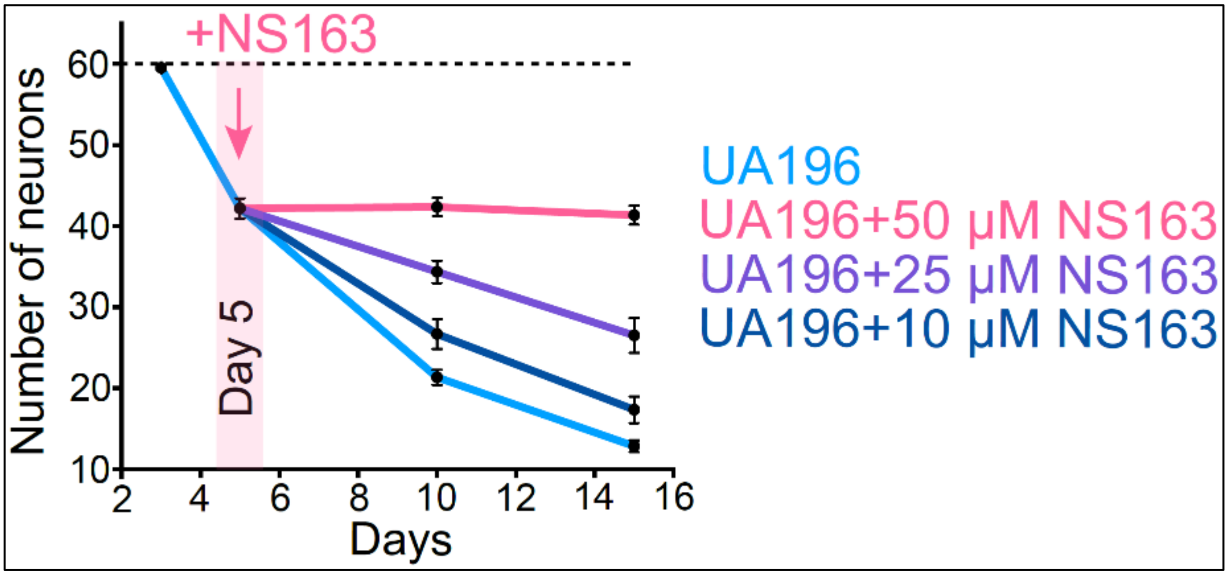

Supplementary Fig. 35. The relative number of healthy DA neurons in UA196 worms during the aging process when treated on day five with the indicated doses of NS163. For each confocal imaging experiment, at least 10 worms were used, and the healthy neurons (GFP signal) were counted manually, and each condition (day) consisted of six independent experiments (total of 60 DA neurons). The data were expressed as mean and the error bars report the s.e.m. (n = 6 independent experiments and each n consisted of 10 technical replicates).

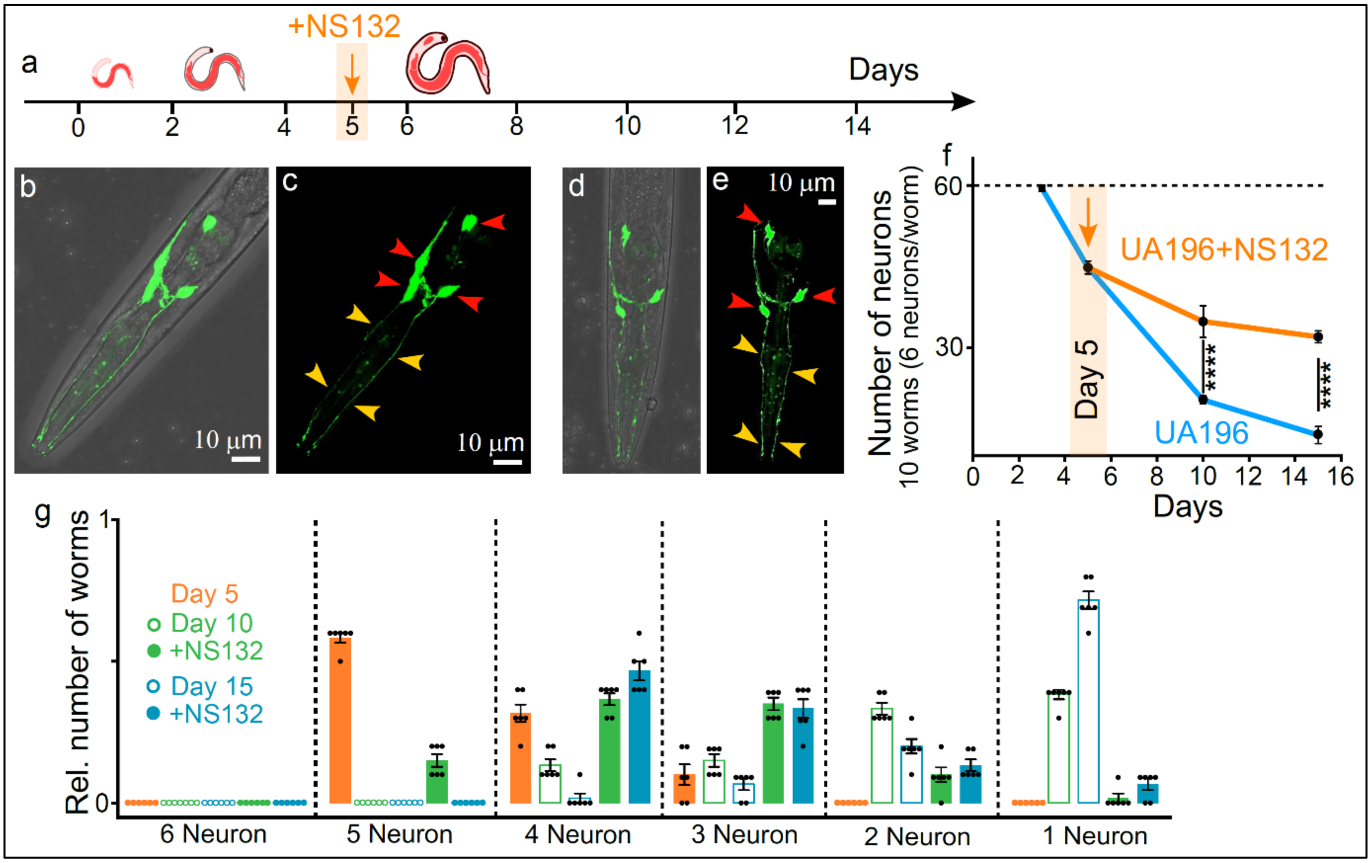

Supplementary Fig. 36. Neuroprotective effect of NS132 on the preexiting PD *C. elegans* model. a, Schematic of the aging process of UA196 worms and their treatment with NS-132 in the late-stage disease onset PD model(day 5). Representative confocal images of UA196 worms on day day 15 in the absence (**b,c**) and presence (**d,e**) of 50 µM NS132 (treated on day 5). Filled red and yellow arrows = healthy neurons and neurites, empty red and yellow arrows = degenerated neurons and neurites. **f**, Statistics for the total number of healthy neurons in UA196 worms during the aging process in the absence and presence of 50 µM NS132. **g**, The relative number of healthy DA neurons in UA196 worms during the aging process in the absence (Open bar and open circle) and when treated with 50 µM NS132 (Closed bar and closed circle) on day 5. The relative number of healthy DA neurons in UA196 worms on day 5 were presented with orange bar. For each confocal imaging experiment at least 10 worms were used, and the healthy neurons were counted manually, and each condition (day) consisted of six independent experiments. The data were expressed as mean and the error bars report the s.e.m. (n = 6 independent experiments and each n consisted of 10 technical replicates). The statistical analysis was performed using ANOVA with Tukey's multiple comparison test. *$p < 0.05$, **$p < 0.01$, ***$p < 0.001$, ****$p < 0.0001$.

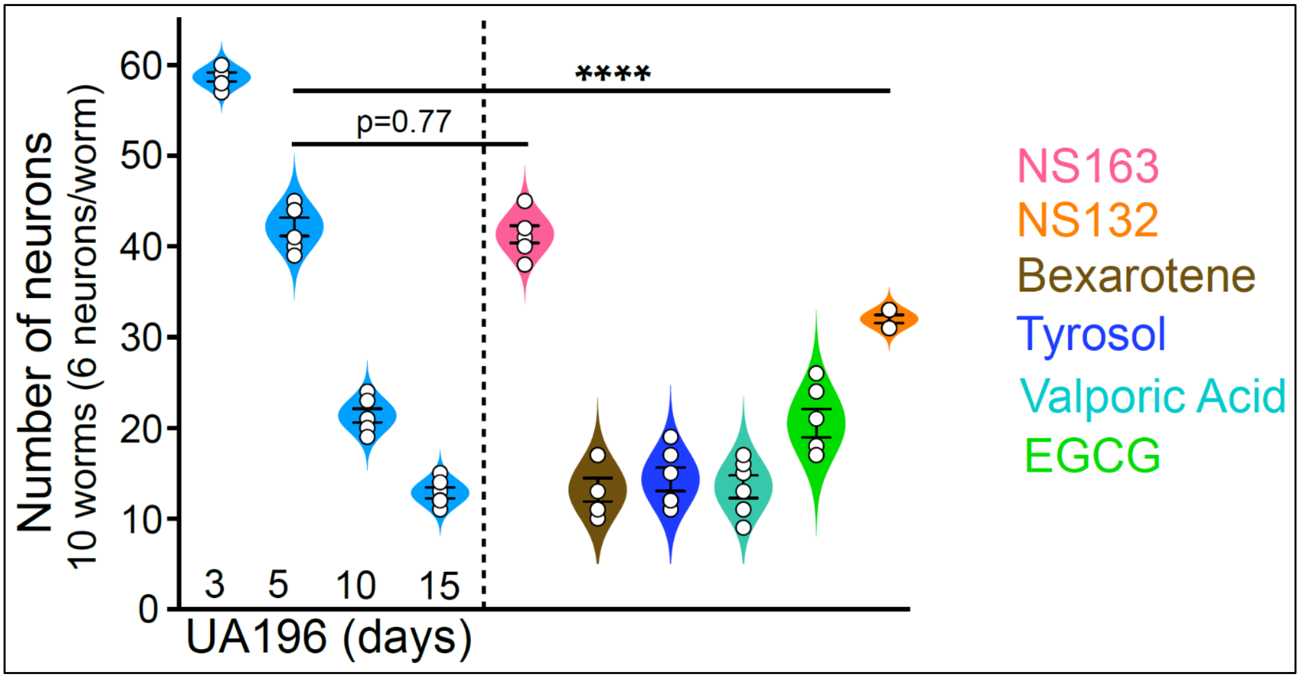

Supplementary Fig. 37. Neuroprotective effect of various ligands on the degeneration of DA neurons in a post-disease PD model of UA196 worms. Statistics for the total number of intact DA neurons in UA196 worms in the absence and presence of the indicated ligands (at 50 µM, day 5) on day 15. For each confocal imaging experiment at least 10 worms were used, and the healthy neurons were counted manually, and each condition (day) consisted of six independent experiments. The data were expressed as mean and the error bars report the s.e.m. (n = 6 independent experiments and each n consisted of 10 technical replicates). The statistical analysis was performed using ANOVA with Tukey's multiple comparison test. $*p < 0.05$, $**p < 0.01$, $***p < 0.001$, $****p < 0.0001$.

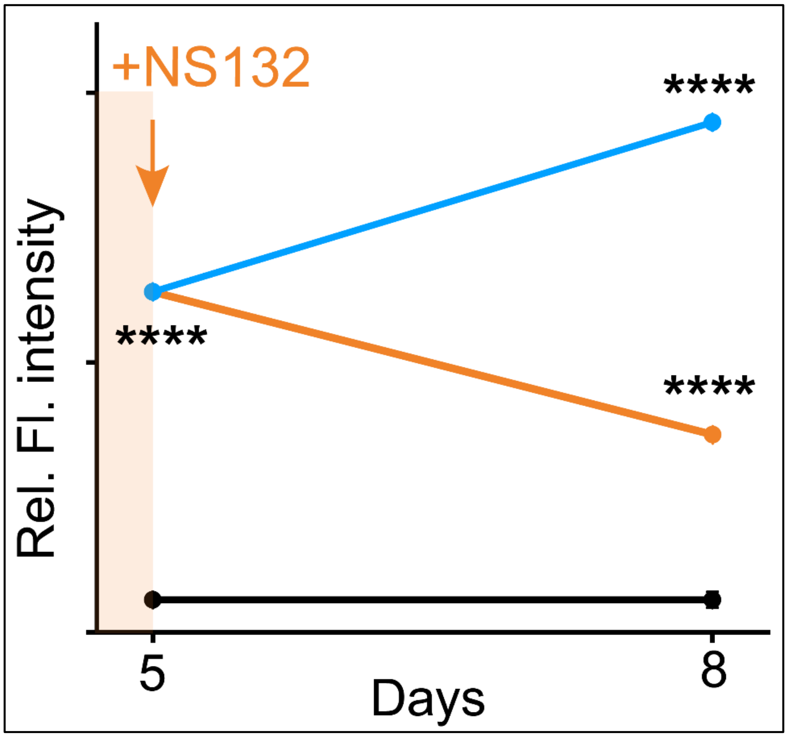

Supplementary Fig. 38. The effect of NS132 on the ROS level in UA196 worms. The comparison of the ROS level in UA196 worms (blue) on day five and day eight when treated (orange) with 50 µM NS132 on day five. For ROS level quantification, at least 50 worms were used and each condition consisted of three independent experiments with freshly bleached worms. The data were expressed as mean and the error bars report the s.d. (n = 3 independent experiments and each n consisted of three technical replicates). The statistical analysis was performed using ANOVA with Tukey's multiple comparison test. *p < 0.05, **p < 0.01, ***p < 0.001, ****p < 0.0001.

Supplementary Fig. 39. ^{1}H-NMR of 6-chloro-5-nitro-2-picoline

^{1}H NMR (500 MHz, $CDCl_3$) δ 2.63 – 2.66 (s, 3H), 7.26 – 7.29 (d, *J* = 8.0 Hz, 1H), 8.13 – 8.18 (d, *J* = 8.1 Hz, 1H). HRMS (m/z): [M]+ calcd. for $C_6H_5ClN_2O_2$, 173.0112; found, 173.0114.

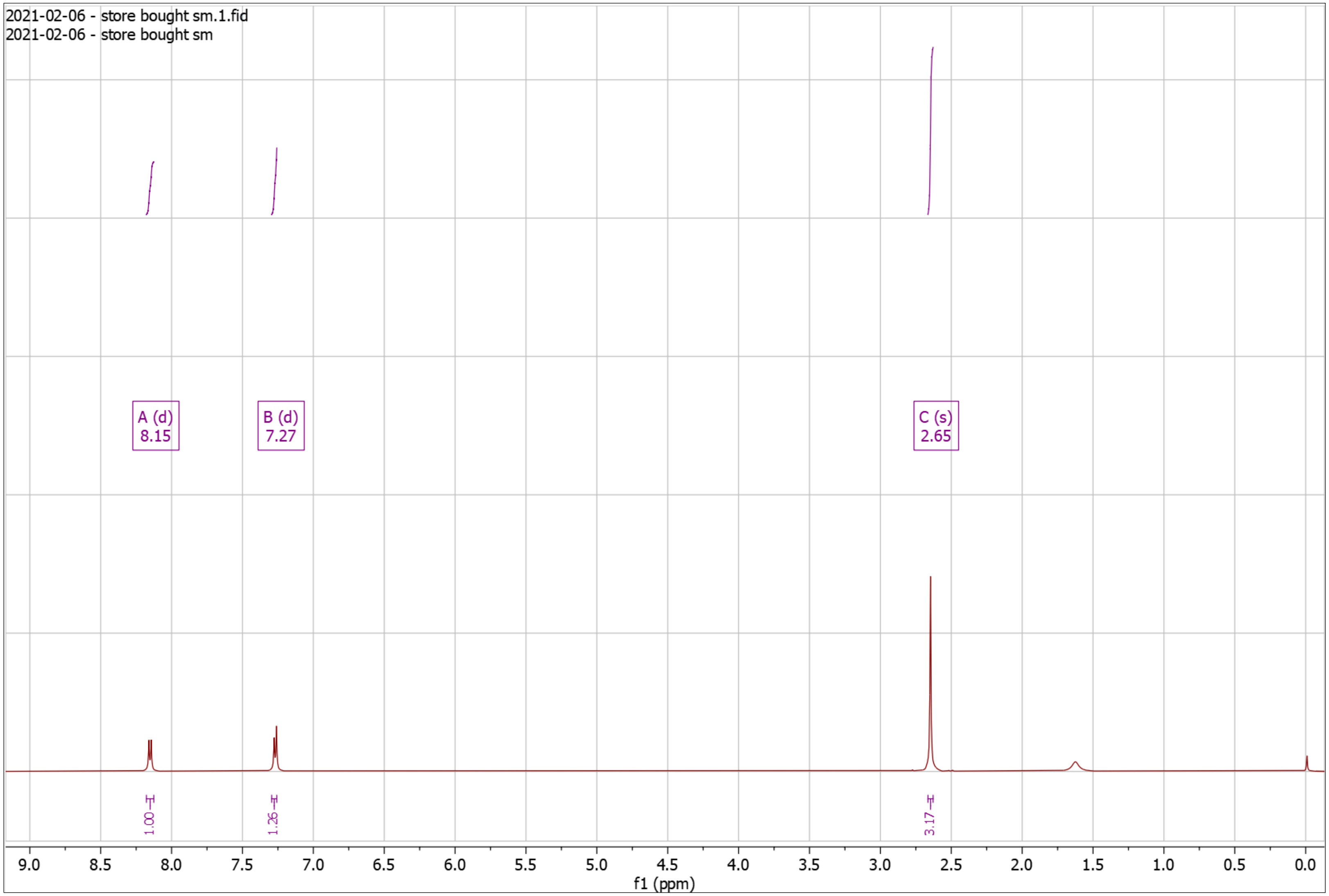

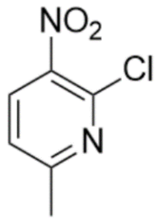

Supplementary Fig. 40. ^{1}H-NMR of NS41 Pro

^{1}H NMR (500 MHz, $CDCl_3$) δ 1.42 – 1.45 (s, 9H), 2.46 – 2.48 (s, 3H), 4.88 – 4.92 (s, 2H), 6.87 – 6.91 (d, *J* = 8.1 Hz, 1H), 8.22 – 8.25 (d, *J* = 8.1 Hz, 1H). [M+Na]+ calcd. for $C_{12}H_{16}N_2NaO_5$, 291.0951; found, 291.0955.

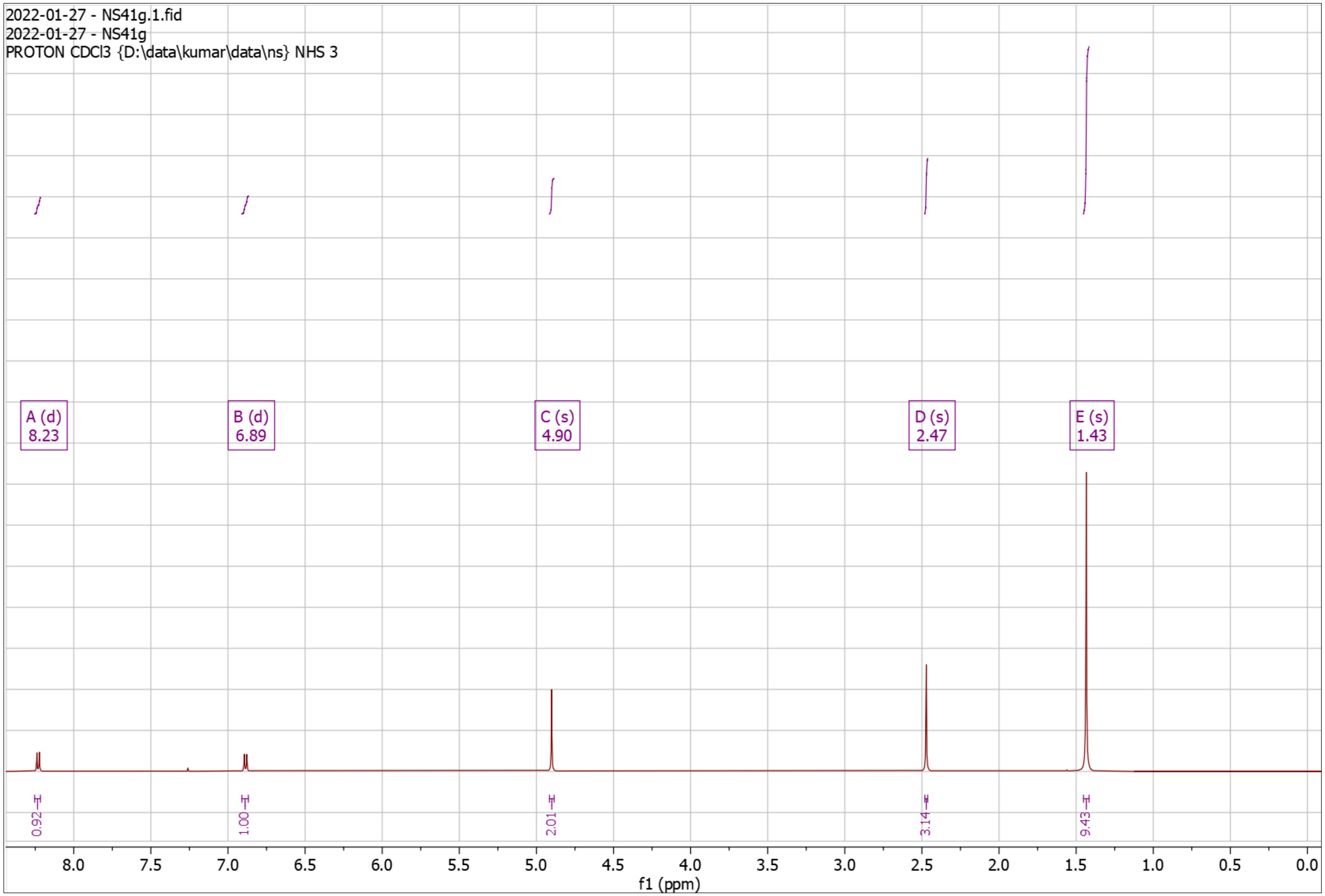

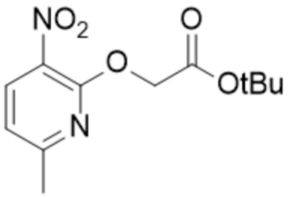

Supplementary Fig. 41. ^{1}H-NMR of NS41 Dep

^{1}H NMR (500 MHz, DMSO) δ 2.44 – 2.47 (s, 3H), 4.98 – 5.01 (s, 2H), 7.10 – 7.15 (d, *J* = 8.1 Hz, 1H), 8.35 – 8.40 (d, *J* = 8.1 Hz, 1H), 13.02 – 13.05 (s, 1H). [M]- calcd. for $C_8H_8N_2O_5$, 211.03604; found, 211.03332.

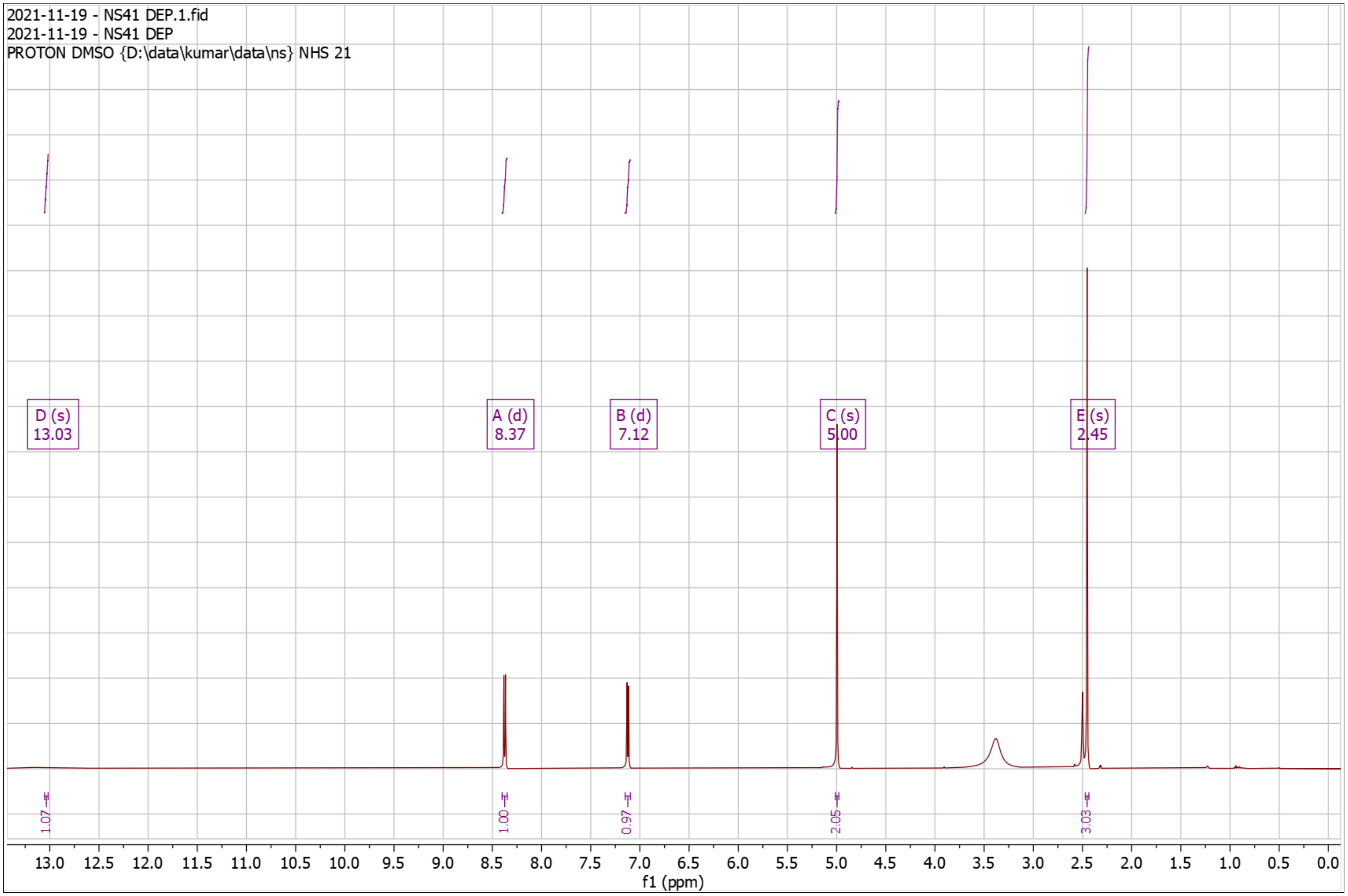

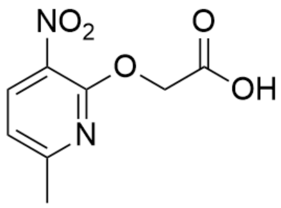

Supplementary Fig. 42. ^{1}H-NMR of RD124 Pro

^{1}H NMR (500 MHz, $CDCl_3$) δ 1.40 – 1.45 (s, 9H), 1.98 – 2.06 (m, 2H), 2.50 – 2.54 (s, 3H), 3.31 – 3.37 (t, J = 6.3 Hz, 2H), 4.53 – 4.59 (t, J = 5.9 Hz, 2H), 5.08 – 5.26 (s, 1H), 6.82 – 6.88 (d, J = 8.1 Hz, 1H), 8.17 – 8.23 (d, J = 8.1 Hz, 1H). [M+Na]+ calcd. For $C_{14}H_{21}N_3NaO_5$, 334.1373; found, 334.1367.

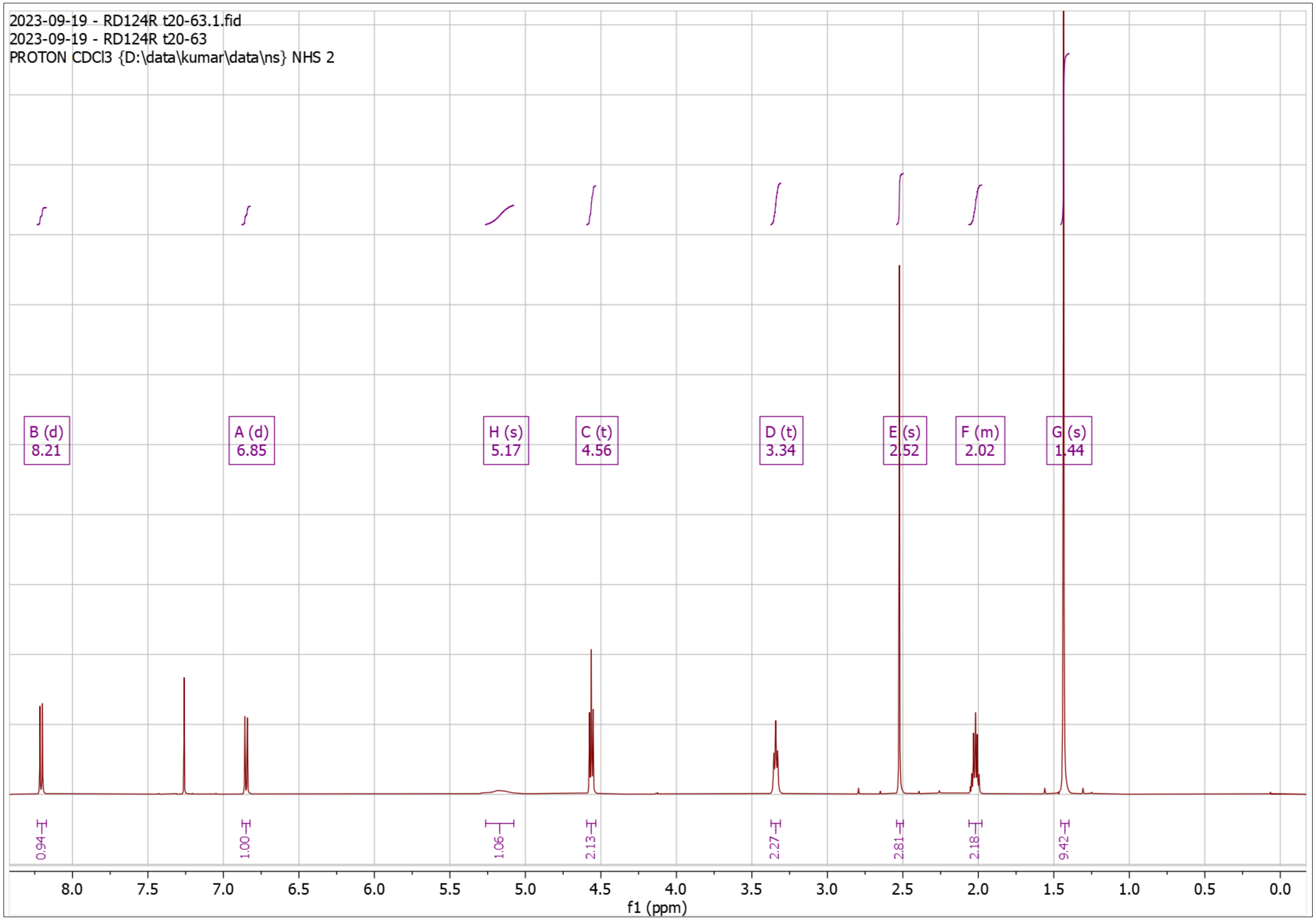

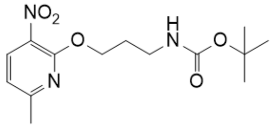

Supplementary Fig. 43. ^{1}H-NMR of RD124 Dep

^{1}H NMR (500 MHz, $CDCl_3$) δ 2.27 – 2.34 (p, *J* = 5.3 Hz, 2H), 2.52 – 2.55 (s, 3H), 3.27 – 3.30 (s, 2H), 3.34 – 3.38 (t, 2H), 4.63 – 4.69 (t, *J* = 5.5 Hz, 2H), 6.88 – 6.93 (d, *J* = 8.1 Hz, 1H), 8.24 – 8.29 (d, *J* = 8.1 Hz, 1H). [M]+ calcd. for C9H13N3O3, 212.1030; found, 212.1096.alcd. for $C_9H_{13}N_3O_3$, 212.1030; found, 212.1096.

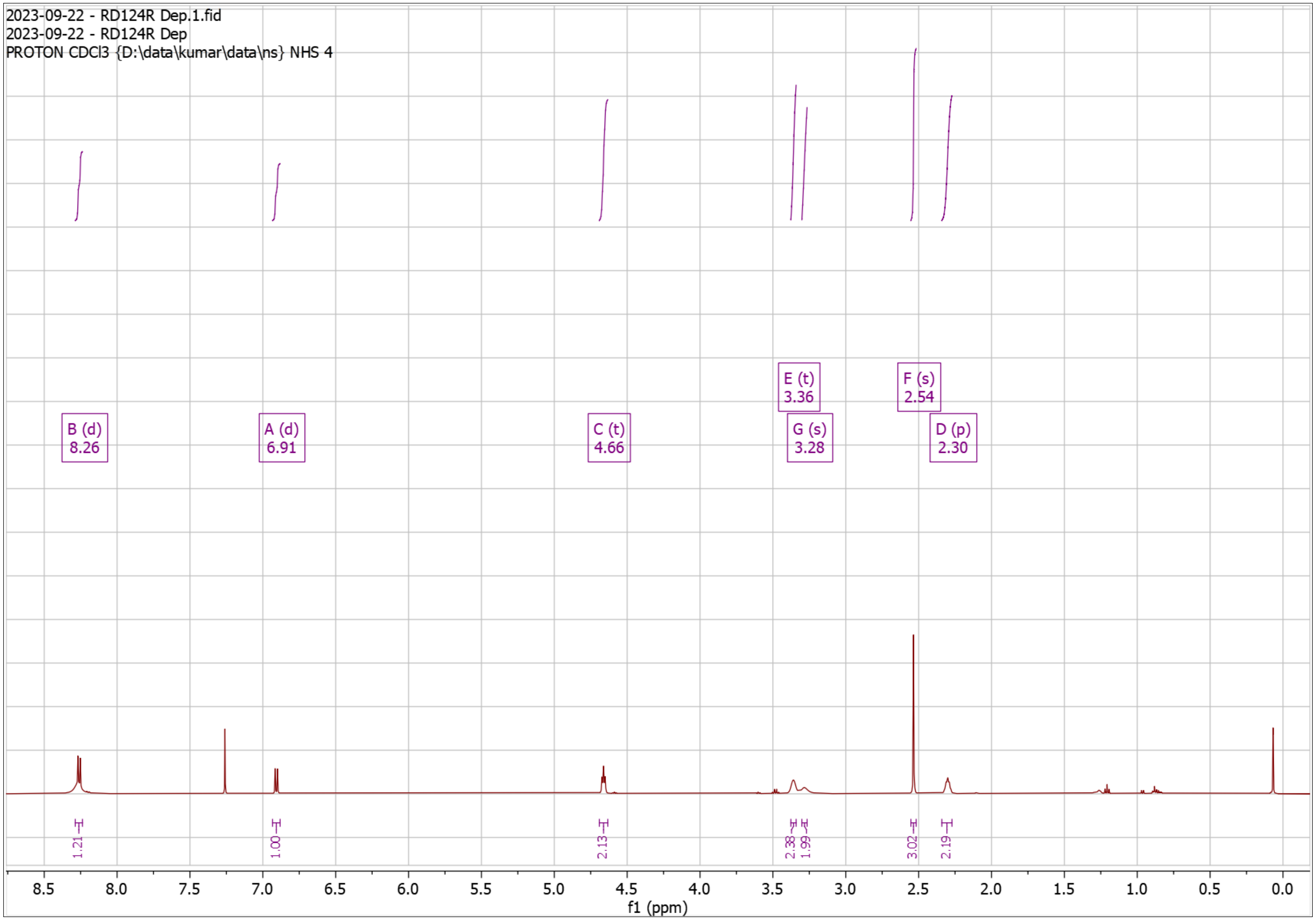

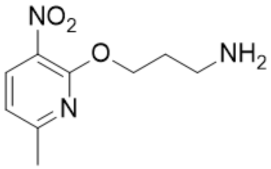

Supplementary Fig. 44. ^{1}H-NMR of NS224

^{1}H NMR (500 MHz, $CDCl_3$) δ 2.52 – 2.56 (s, 3H), 5.56 – 5.60 (s, 2H), 6.83 – 6.88 (d, *J* = 8.1 Hz, 1H), 7.28 – 7.55 (m, 5H), 8.18 – 8.25 (d, *J* = 8.1 Hz, 1H). [M]+ calcd. for $C_{13}H_{12}N_2O_3$, 245.09207; found, 245.09267.

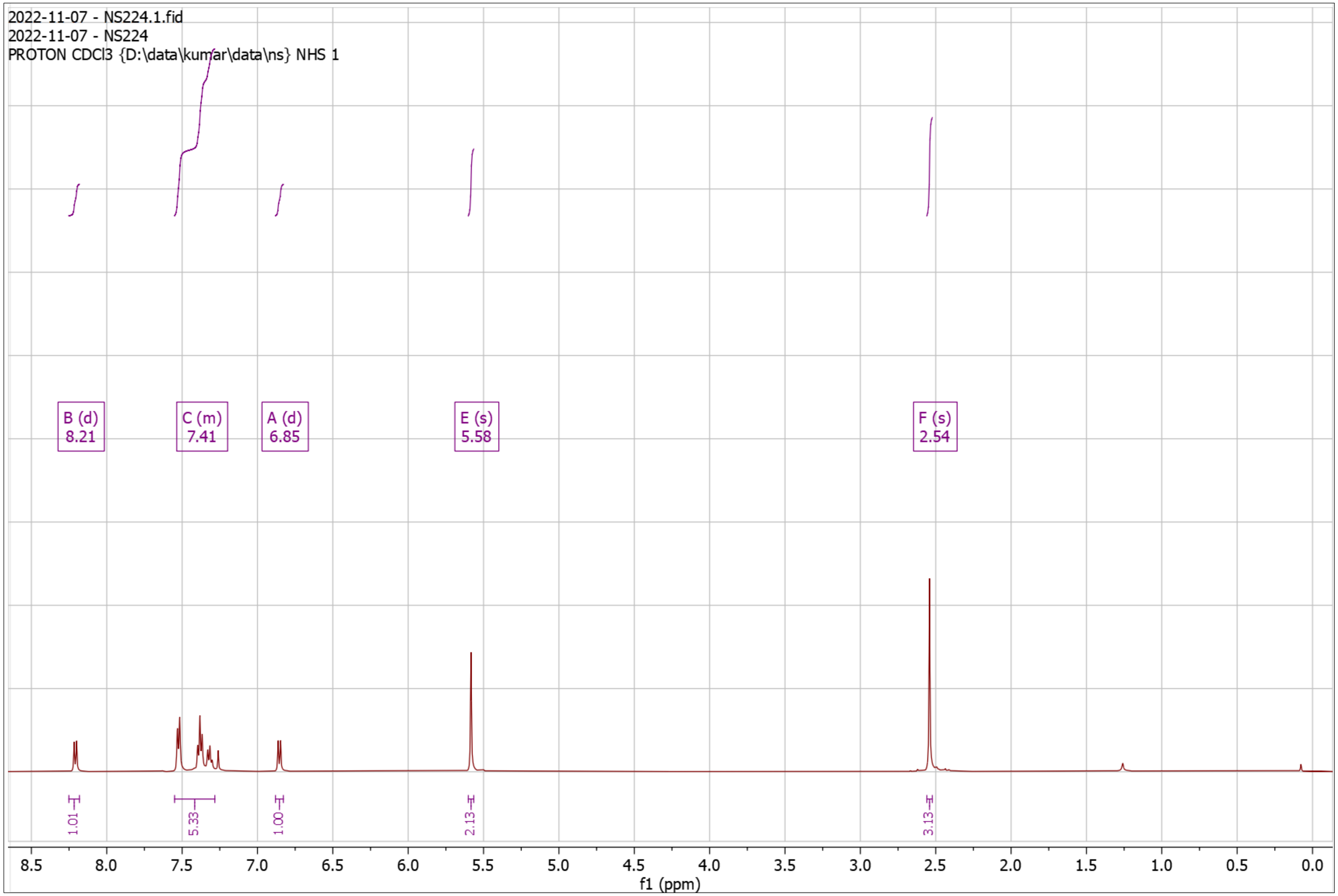

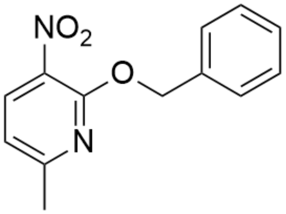

Supplementary Fig. 45. ^{1}H-NMR of RD196

^{1}H NMR (500 MHz, $CDCl_3$) δ 2.46 – 2.50 (t, J = 2.4 Hz, 1H), 2.53 – 2.57 (s, 3H), 5.13 – 5.17 (d, J = 2.4 Hz, 2H), 6.88 – 6.93 (d, J = 8.0 Hz, 1H), 8.20 – 8.26 (d, J = 8.1 Hz, 1H). [M]+ calcd. for $C_9H_8N_2O_3$, 193.0608; found, 193.0607.

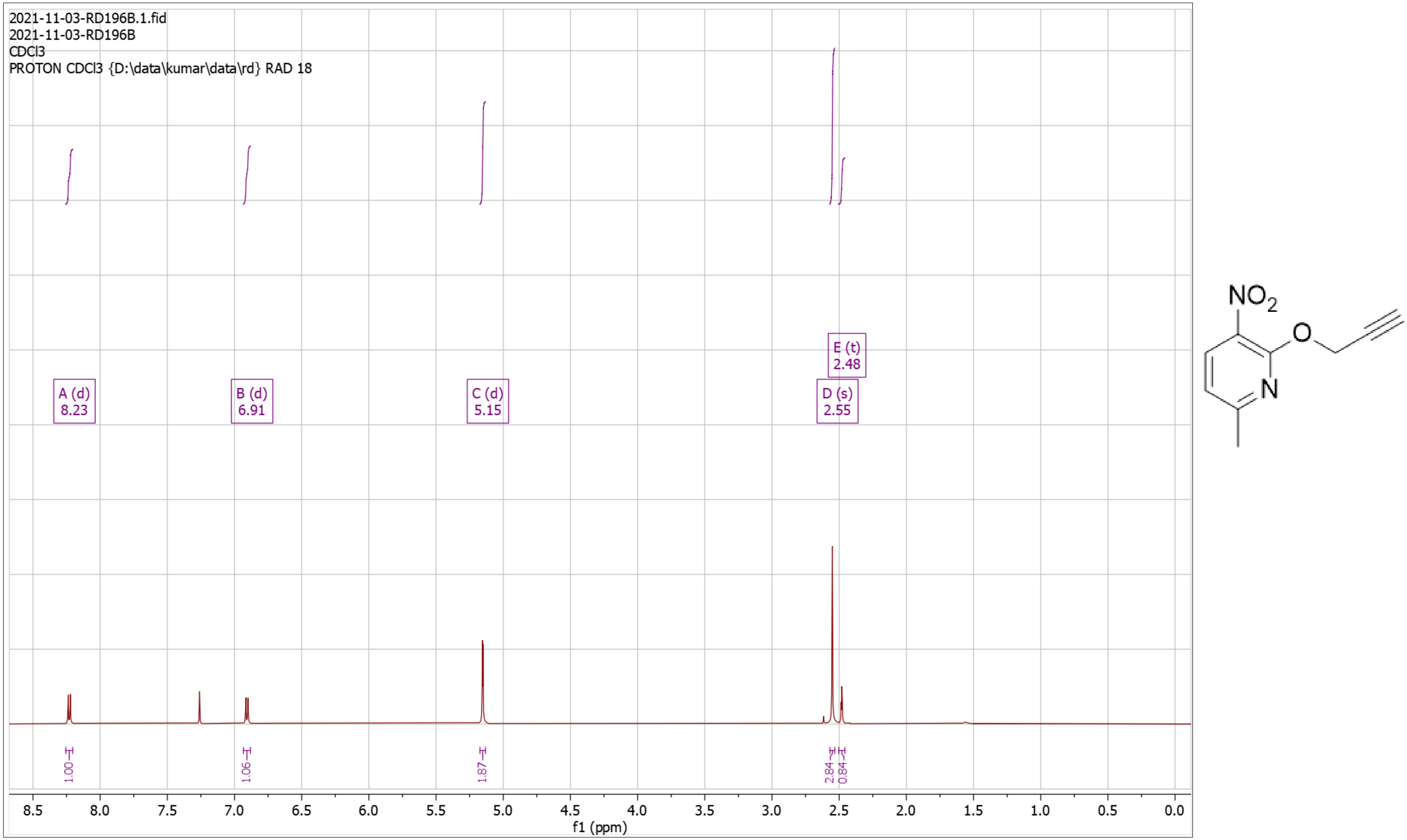

Supplementary Fig. 46. ^{1}H-NMR of NS197

^{1}H NMR (500 MHz, $CDCl_3$) δ 0.93 – 1.01 (t, *J* = 7.4 Hz, 3H), 1.44 – 1.55 (h, *J* = 7.4 Hz, 2H), 1.74 – 1.83 (p, *J* = 6.8 Hz, 2H), 2.48 – 2.52 (s, 3H), 4.43 – 4.50 (t, *J* = 6.6 Hz, 2H), 6.77 – 6.83 (d, *J* = 8.1 Hz, 1H), 8.12 – 8.17 (d, *J* = 8.1 Hz, 1H). [M]+ calcd. for $C_{10}H_{14}N_2O_3$, 210.1004; found, 210.1.

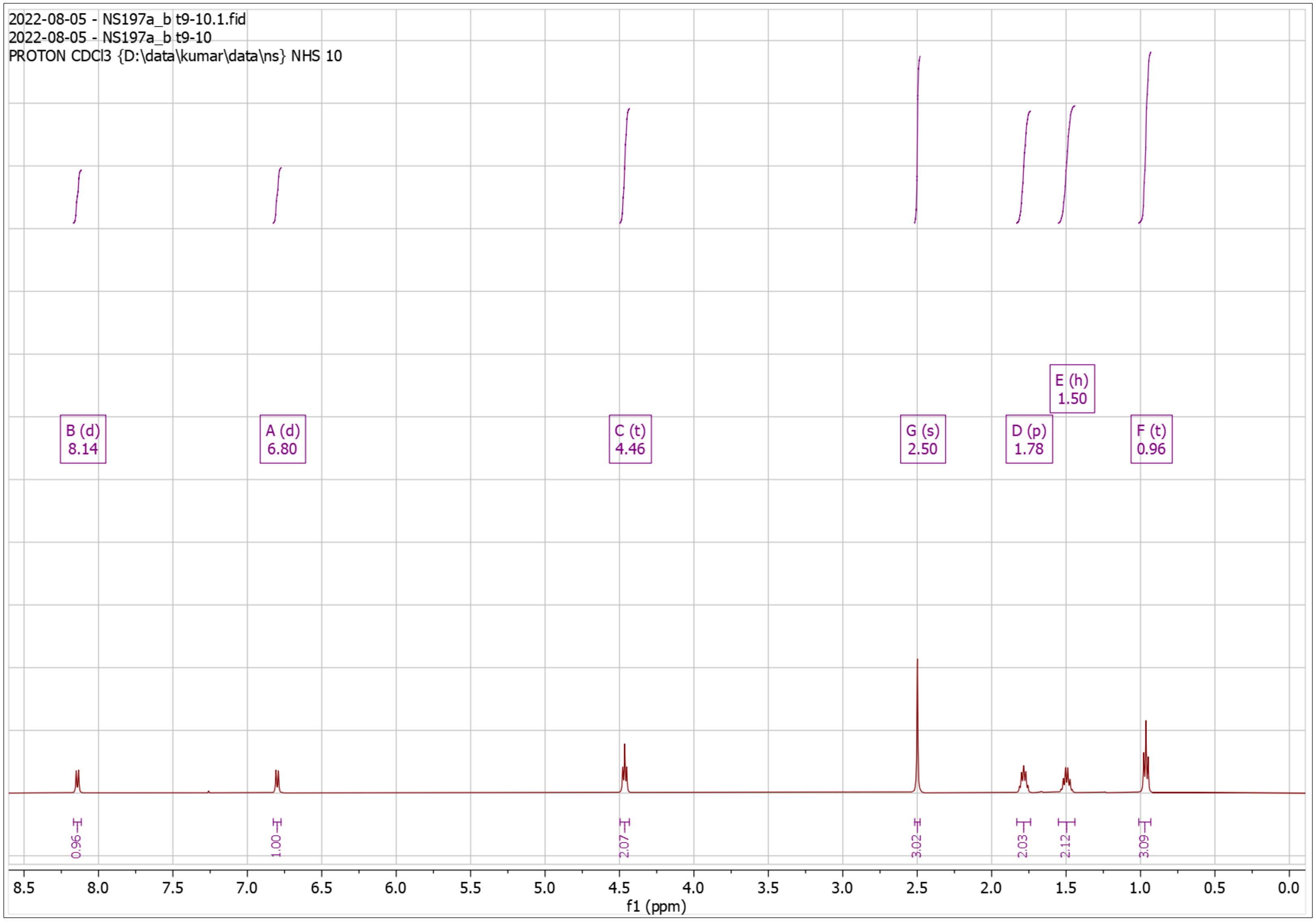

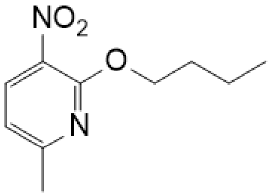

Supplementary Fig. 47. ^{1}H-NMR of RD222

^{1}H NMR (500 MHz, $CDCl_3$) δ 1.02 – 1.35 (m, 5H), 1.64 – 1.92 (m, 6H), 2.47 – 2.52 (s, 3H), 4.23 – 4.29 (d, J = 6.3 Hz, 2H), 6.76 – 6.83 (d, J = 8.1 Hz, 1H), 8.13 – 8.17 (d, J = 8.0 Hz, 1H). [M]+ calcd. for $C_{13}H_{18}N_2O_3$, 250.1317; found, 250.1548.

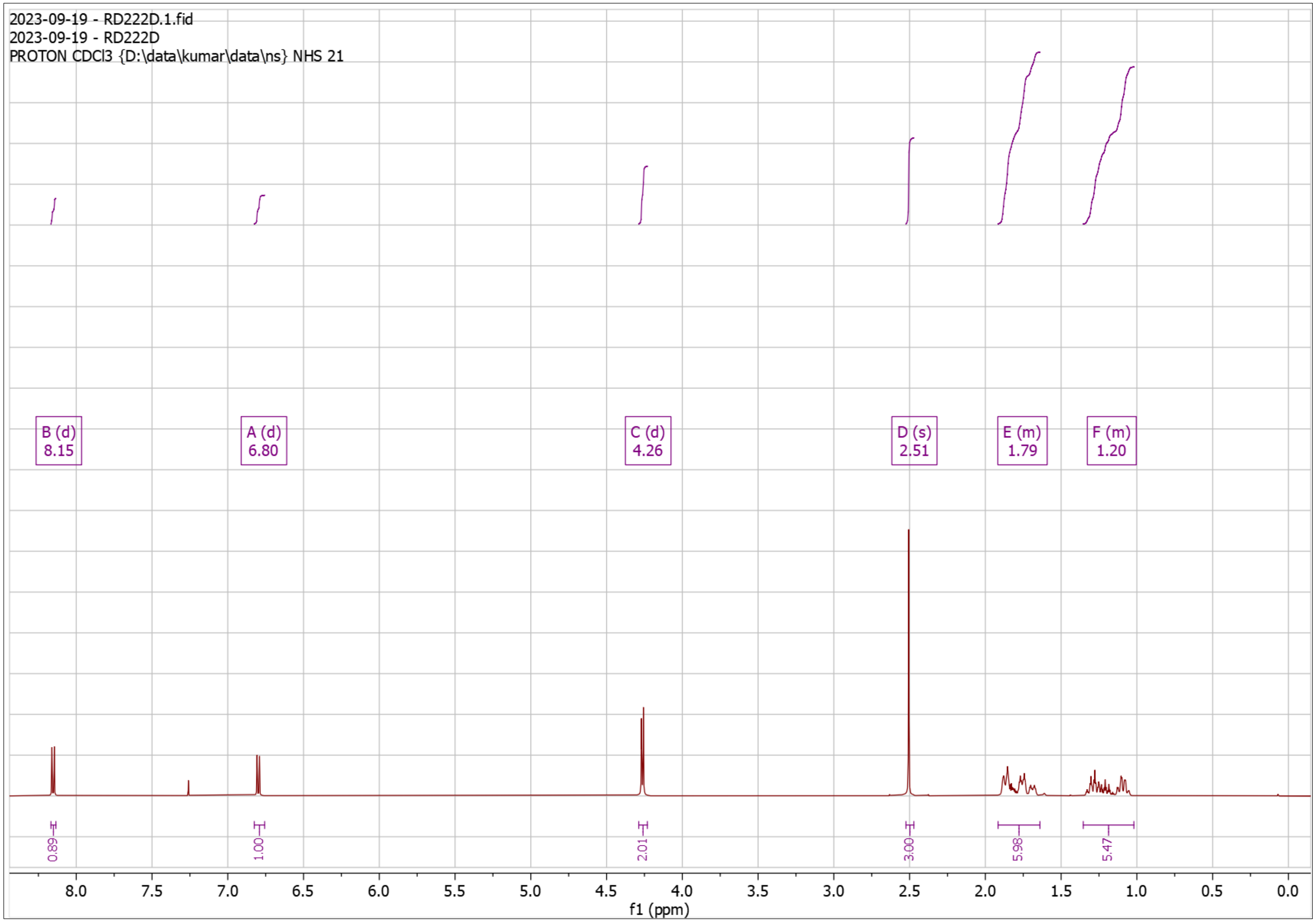

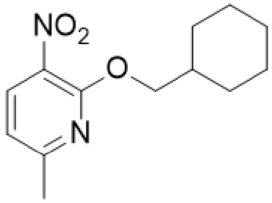

Supplementary Fig. 48. ^{1}H-NMR of RD247

^{1}H NMR (500 MHz, $CDCl_3$) δ 0.87 – 0.93 (t, *J* = 7.1 Hz, 3H), 1.26 – 1.41 (dt, *J* = 7.6, 3.7 Hz, 4H), 1.42 – 1.52 (p, *J* = 6.8 Hz, 2H), 1.78 – 1.86 (p, *J* = 6.9 Hz, 2H), 2.49 – 2.53 (s, 3H), 4.44 – 4.50 (t, *J* = 6.7 Hz, 2H), 6.78 – 6.83 (d, *J* = 8.1 Hz, 1H), 8.14 – 8.19 (d, *J* = 8.0 Hz, 1H).. [M]+ calcd. for $C_{12}H_{18}N_2O_3$, 239.1390; found, 239.1394.

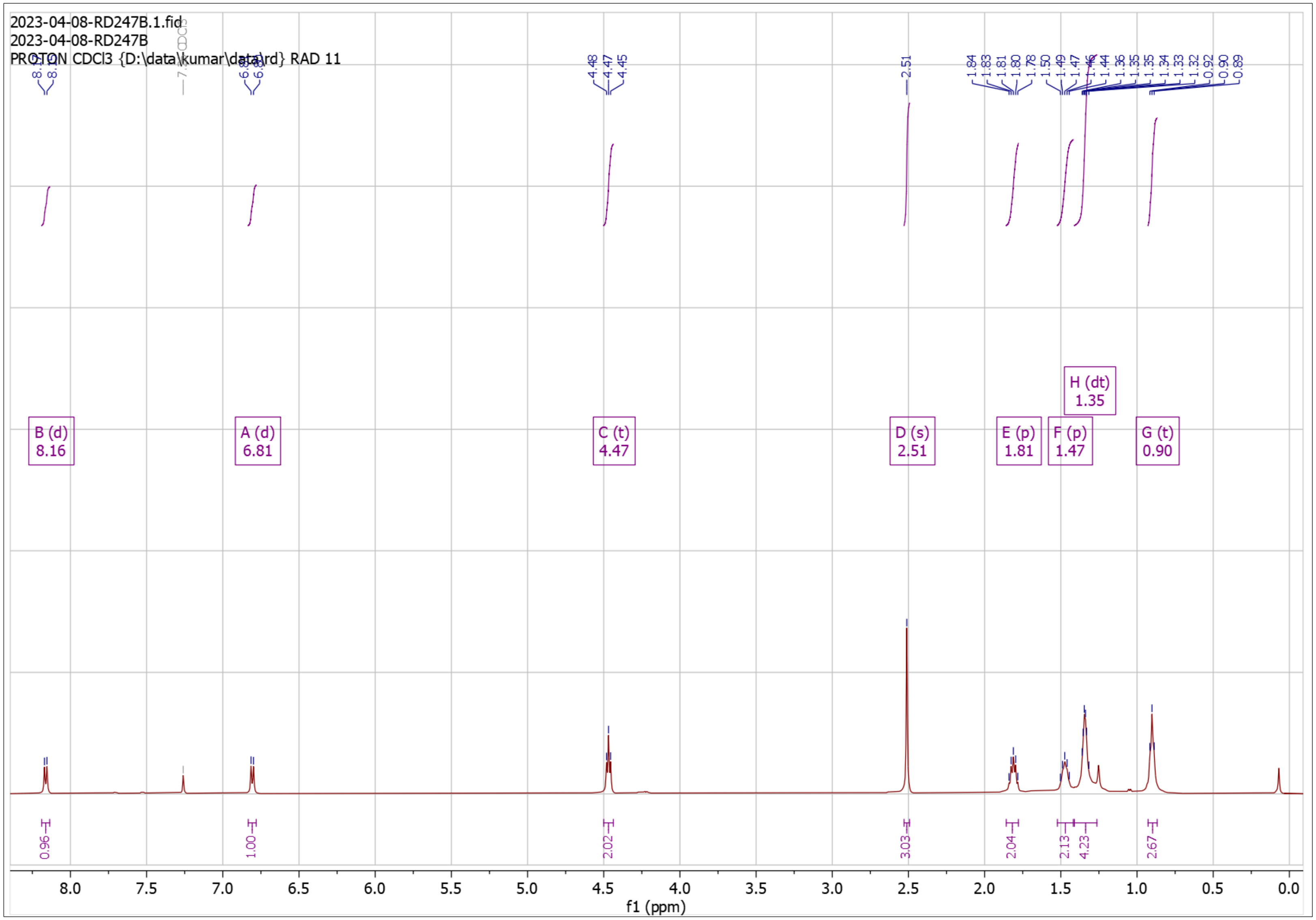

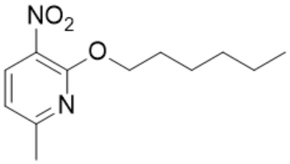

Supplementary Fig. 49. ^{1}H-NMR of NS101

^{1}H NMR (500 MHz, DMSO) δ 8.19 – 8.29 (d, *J* = 8.2 Hz, 1H), 8.65 – 8.73 (d, *J* = 8.2 Hz, 1H). HRMS (m/z): [M]+ calcd. for $C_6H_3ClN_2O_4$, 202.9854; found, 202.9859.

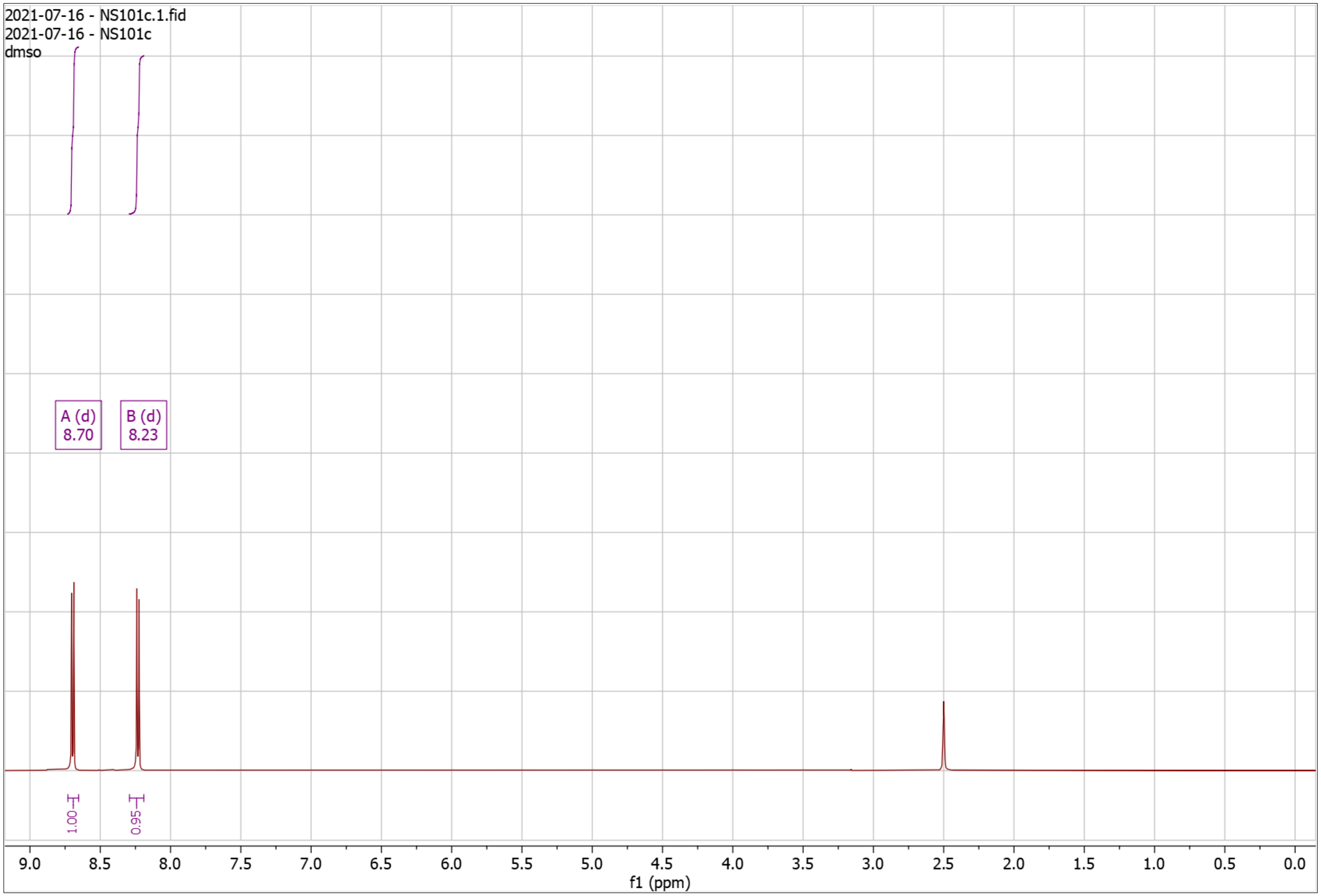

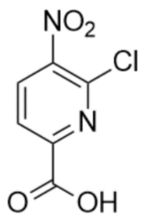

Supplementary Fig. 50. ^{1}H-NMR of RD121

^{1}H NMR (500 MHz, $CDCl_3$) δ 8.15 – 8.25 (d, *J* = 8.1 Hz, 1H), 8.33 – 8.38 (d, *J* = 8.2 Hz, 1H). HRMS (m/z): [M]+ calcd. for $C_6H_2Cl_2N_2O_3$, 220.9515; found, 220.9516.

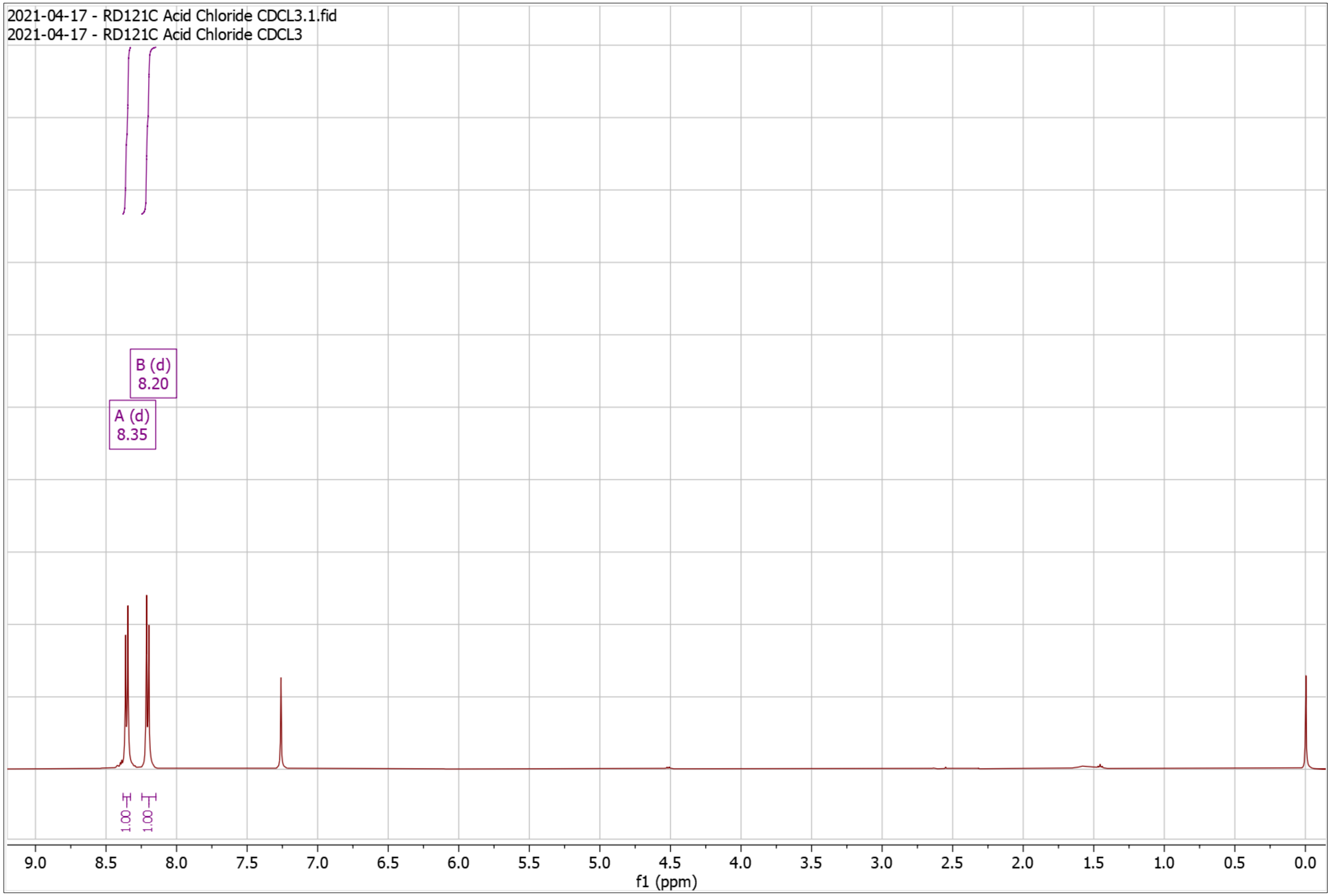

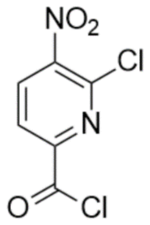

Supplementary Fig. 51. ^{1}H-NMR of RD127 Pro

^{1}H NMR (500 MHz, $CDCl_3$) δ 1.47 – 1.50 (s, 9H), 2.38 – 2.42 (s, 3H), 4.87 – 4.91 (s, 2H), 6.80 – 6.86 (d, *J* = 7.9 Hz, 1H), 8.34 – 8.42 (m, 2H), 8.60 – 8.66 (d, *J* = 7.9 Hz, 1H), 10.02 – 10.06 (s, 1H); HRMS (m/z): [M]+ calcd. for $C_{18}H_{19}ClN_4O_6$, 423.1066; found, 423.1055.

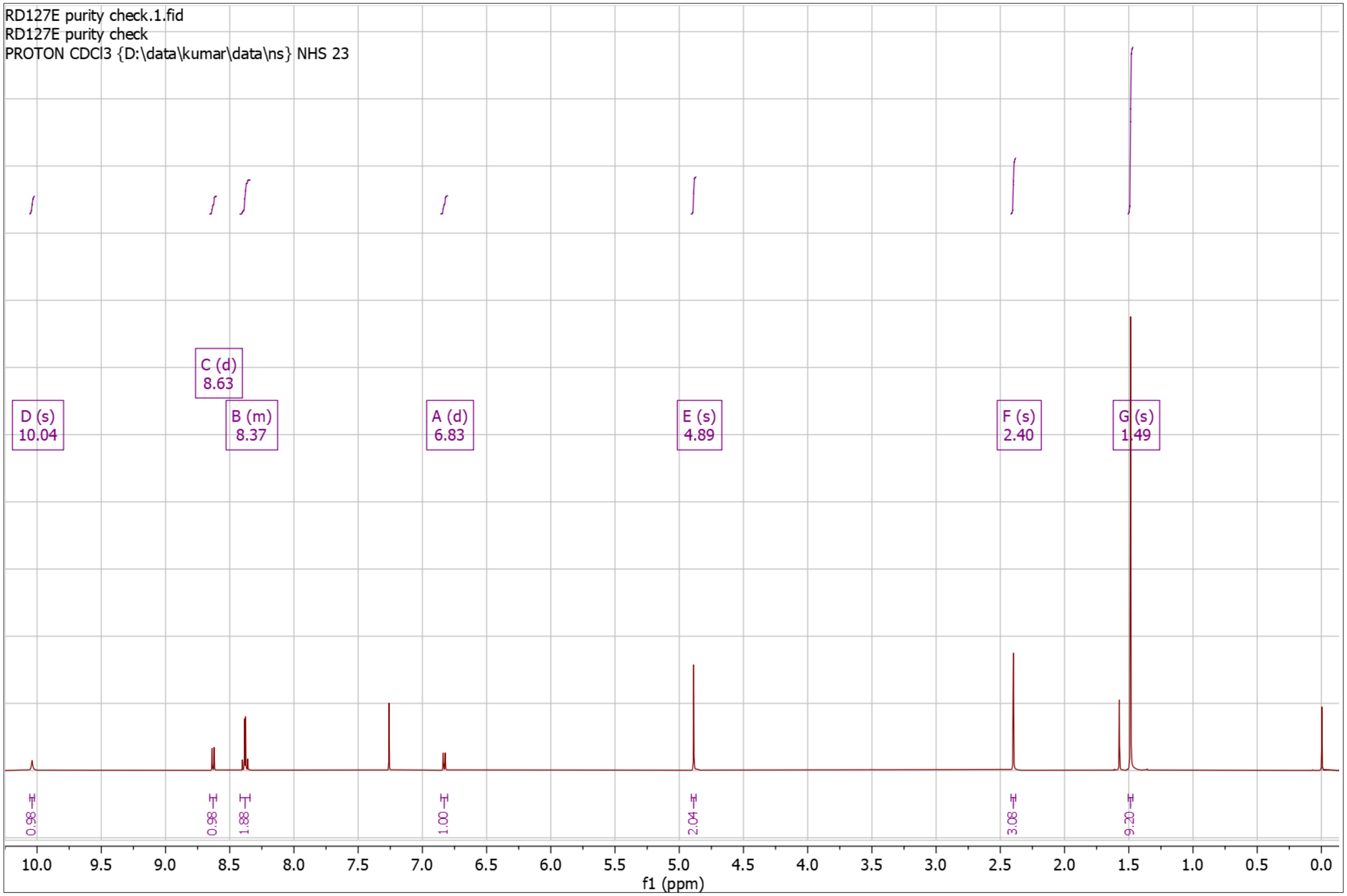

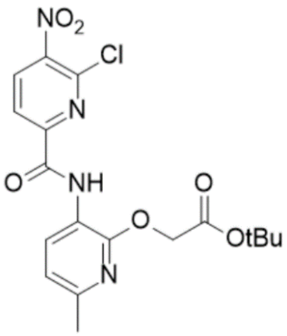

Supplementary Fig. 52. ^{1}H-NMR of RD127 Dep

^{1}H NMR (500 MHz, DMSO) δ 2.31 – 2.40 (s, 3H), 4.92 – 5.00 (s, 2H), 6.93 – 7.00 (d, J = 7.9 Hz, 1H), 8.33 – 8.42 (d, J = 8.3 Hz, 1H), 8.42 – 8.53 (d, J = 7.9 Hz, 1H), 8.74 – 8.86 (d, J = 8.3 Hz, 1H), 9.96 – 10.01 (s, 1H), 12.84 – 13.06 (s, 1H); HRMS (m/z): [M]+ calcd. for $C_{14}H_{11}ClN_4O_6$, 367.0440; found, 367.0423.

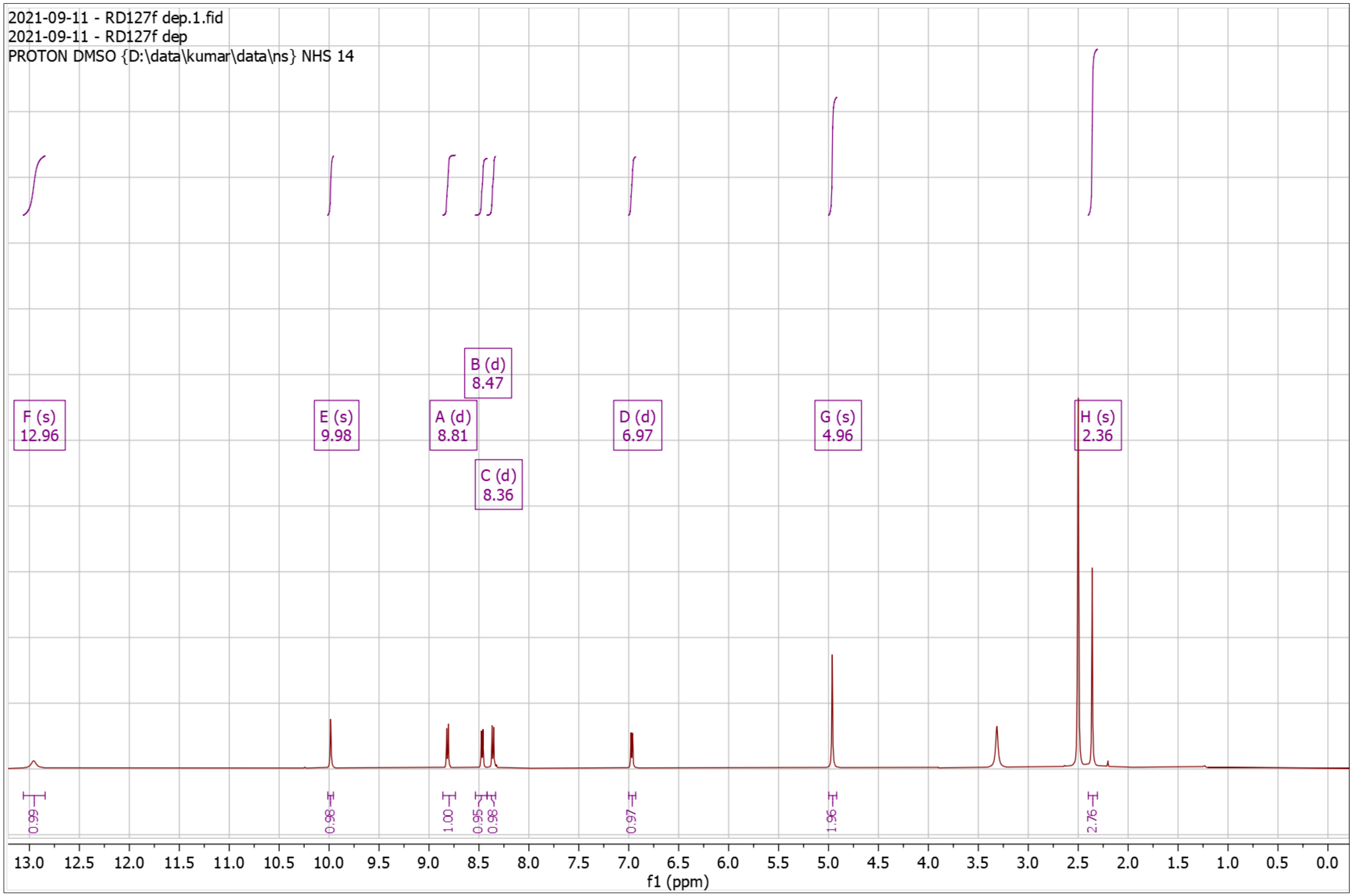

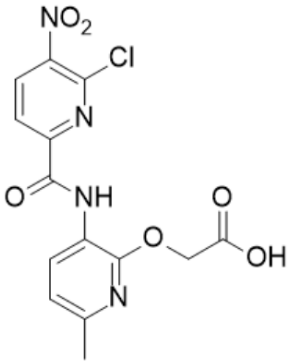

Supplementary Fig. 53. ^{1}H-NMR of NS48 Pro

^{1}H NMR (500 MHz, $CDCl_3$) δ 0.96 – 1.03 (d, *J* = 6.6 Hz, 6H), 1.42 – 1.48 (s, 9H), 1.63 – 1.69 (q, *J* = 7.0 Hz, 2H), 1.71 – 1.83 (dh, *J* = 13.3, 6.6 Hz, 1H), 2.36 – 2.44 (s, 3H), 3.72 – 3.83 (td, *J* = 7.3, 5.4 Hz, 2H), 4.80 – 4.88 (s, 2H), 6.77 – 6.88 (d, *J* = 8.0 Hz, 1H), 7.51 – 7.59 (d, *J* = 8.5 Hz, 1H), 8.15 – 8.27 (t, *J* = 5.4 Hz, 1H), 8.56 – 8.63 (d, *J* = 8.4 Hz, 1H), 8.65 – 8.71 (d, *J* = 7.9 Hz, 1H), 10.16 – 10.28 (s, 1H); HRMS (m/z): [M]+ calcd. for $C_{23}H_{31}N_5O_6$, 474.2347; found, 474.2349.

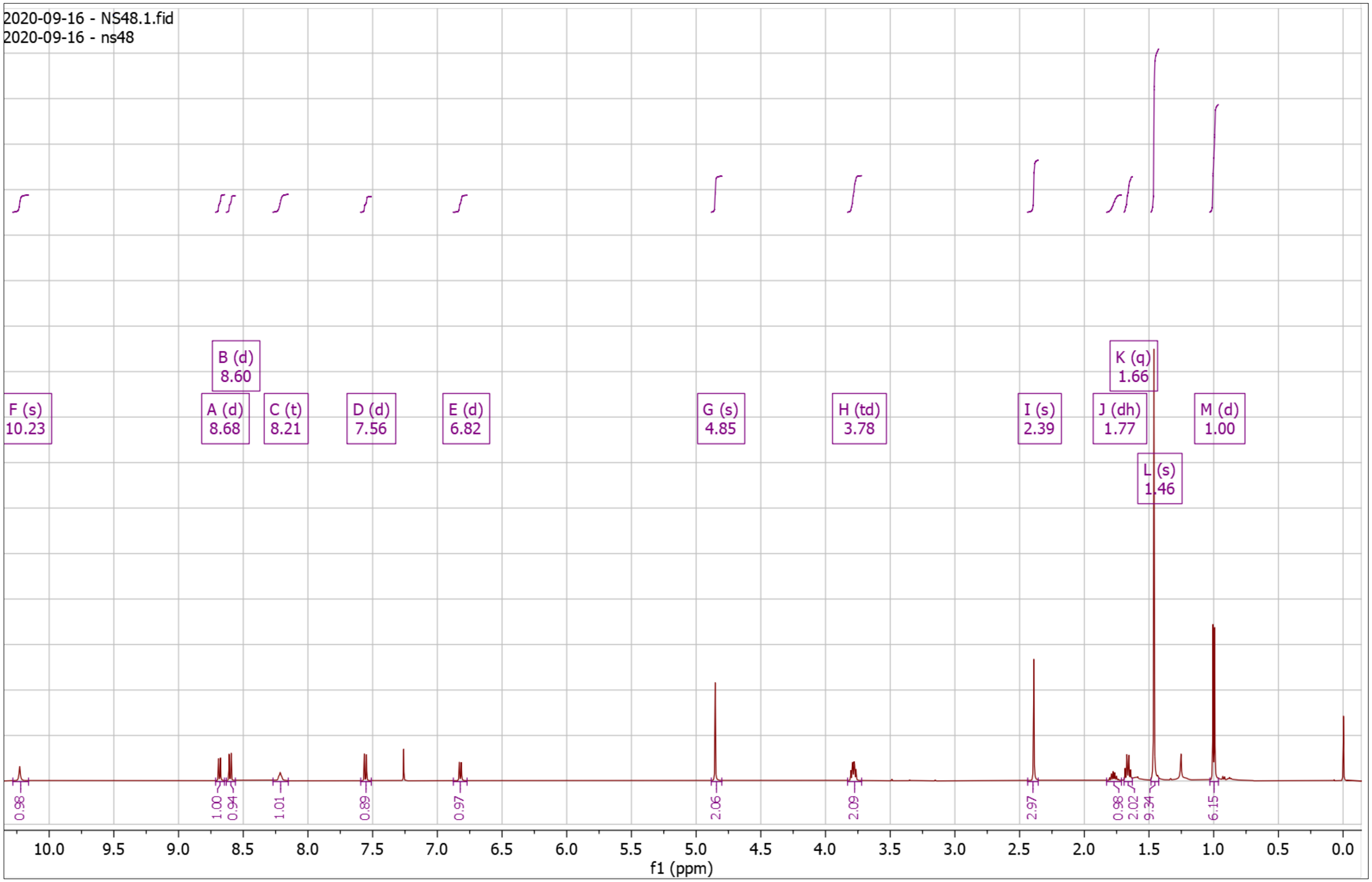

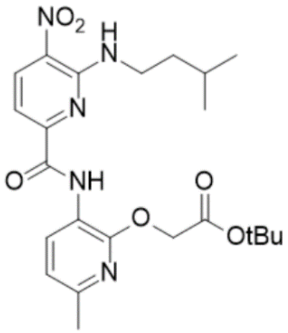

Supplementary Fig. 54. ^{1}H-NMR of NS48 Dep

^{1}H NMR (500 MHz, DMSO) δ 0.90 – 0.93 (d, *J* = 6.5 Hz, 6H), 1.54 – 1.60 (q, *J* = 7.1 Hz, 2H), 1.62 – 1.74 (hept, *J* = 6.6 Hz, 1H), 2.31 – 2.35 (s, 3H), 3.56 – 3.73 (q, *J* = 6.6 Hz, 2H), 4.91 – 4.95 (s, 2H), 6.87 – 6.97 (d, *J* = 8.0 Hz, 1H), 7.33 – 7.44 (d, *J* = 8.4 Hz, 1H), 8.47 – 8.52 (t, *J* = 5.7 Hz, 1H), 8.52 – 8.56 (d, *J* = 7.9 Hz, 1H), 8.56 – 8.64 (d, *J* = 8.4 Hz, 1H), 10.09 – 10.13 (s, 1H), 12.66 – 13.23 (s, 1H); HRMS (m/z): [M]+ calcd. for $C_{19}H_{23}N_5O_6$, 418.1721; found, 418.1704.

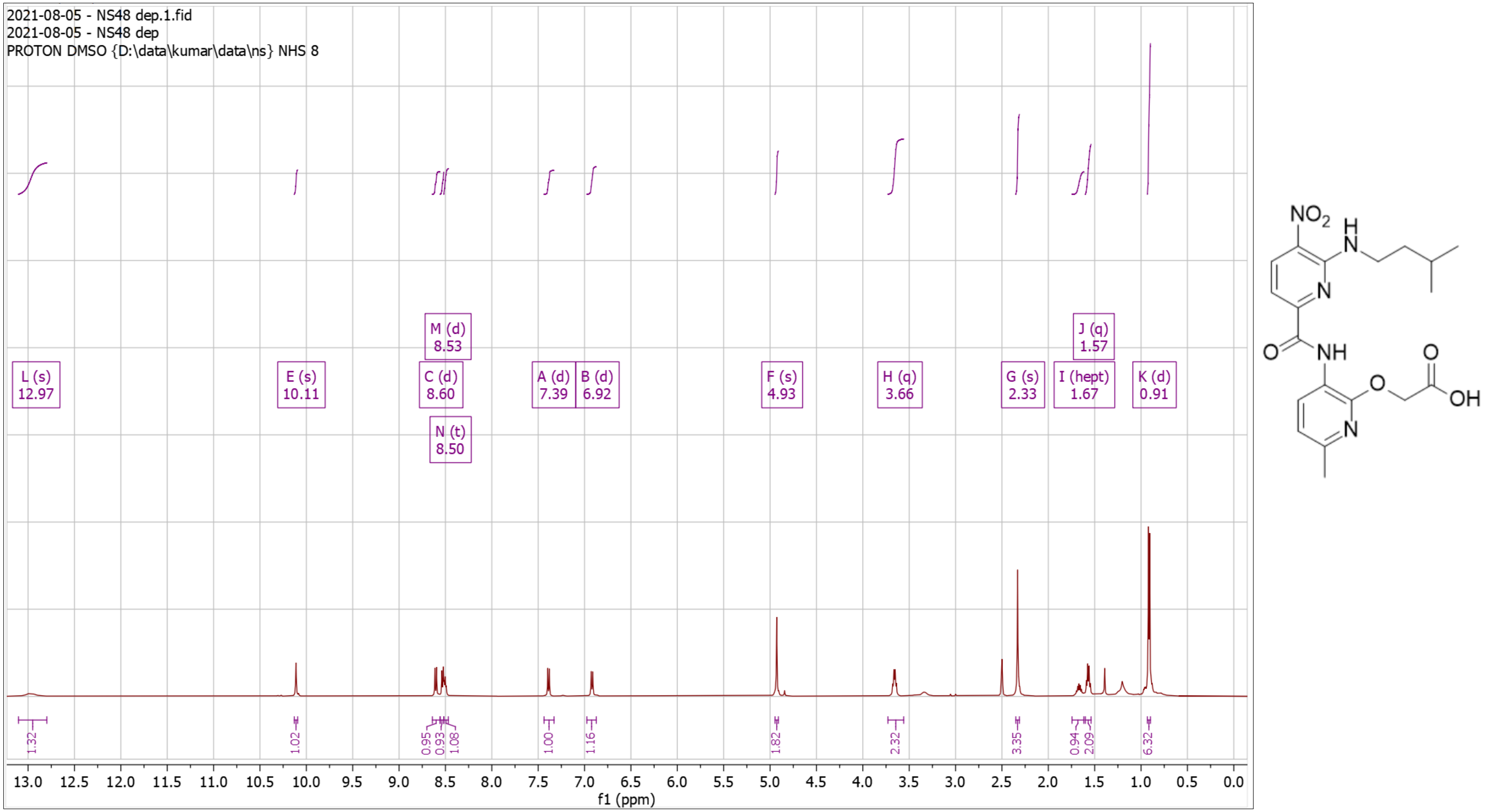

Supplementary Fig. 55. ^{1}H-NMR of NS50 Pro

^{1}H NMR (500 MHz, $CDCl_3$) δ 1.47 – 1.48 (s, 9H), 2.06 – 2.11 (t, *J* = 2.7 Hz, 1H), 2.38 – 2.41 (s, 3H), 2.65 – 2.72 (td, *J* = 6.8, 2.7 Hz, 2H), 3.93 – 4.00 (q, *J* = 6.6 Hz, 2H), 4.90 – 4.94 (s, 2H), 6.80 – 6.85 (d, *J* = 7.9 Hz, 1H), 7.58 – 7.63 (d, *J* = 8.4 Hz, 1H), 8.45 – 8.51 (t, *J* = 6.0 Hz, 1H), 8.60 – 8.65 (d, *J* = 8.4 Hz, 1H), 8.65 – 8.70 (d, *J* = 7.9 Hz, 1H), 10.22 – 10.26 (s, 1H); HRMS (m/z): [M]+ calcd. for $C_{22}H_{25}N_5O_6$, 456.1878; found, 456.1867.

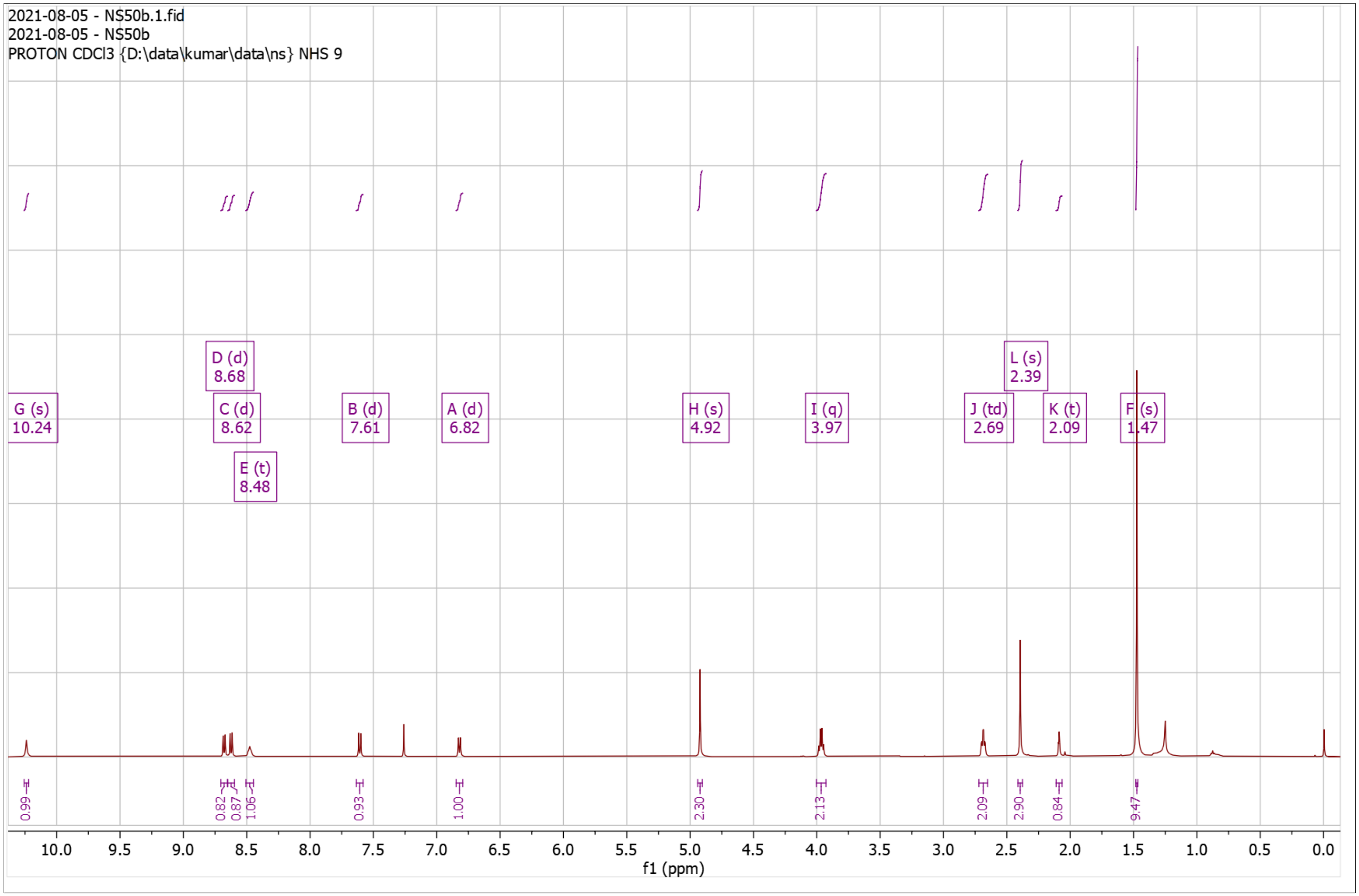

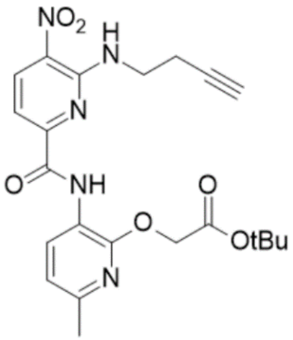

Supplementary Fig. 56. ^{1}H-NMR of NS50 Dep

^{1}H NMR (500 MHz, DMSO) δ 2.32 – 2.36 (s, 3H), 2.55 – 2.67 (td, *J* = 7.2, 2.7 Hz, 2H), 2.85 – 2.90 (d, *J* = 2.7 Hz, 1H), 3.75 – 3.87 (q, *J* = 6.8 Hz, 2H), 4.95 – 5.03 (s, 2H), 6.89 – 6.98 (d, *J* = 7.8 Hz, 1H), 7.40 – 7.50 (d, *J* = 8.1 Hz, 1H), 8.49 – 8.57 (d, *J* = 7.8 Hz, 1H), 8.59 – 8.68 (d, *J* = 8.1 Hz, 1H), 8.68 – 8.78 (t, *J* = 6.0 Hz, 1H), 10.16 – 10.23 (s, 1H); HRMS (m/z): [M]+ calcd. for $C_{18}H_{17}N_5O_6$, 400.1252; found, 400.1251.

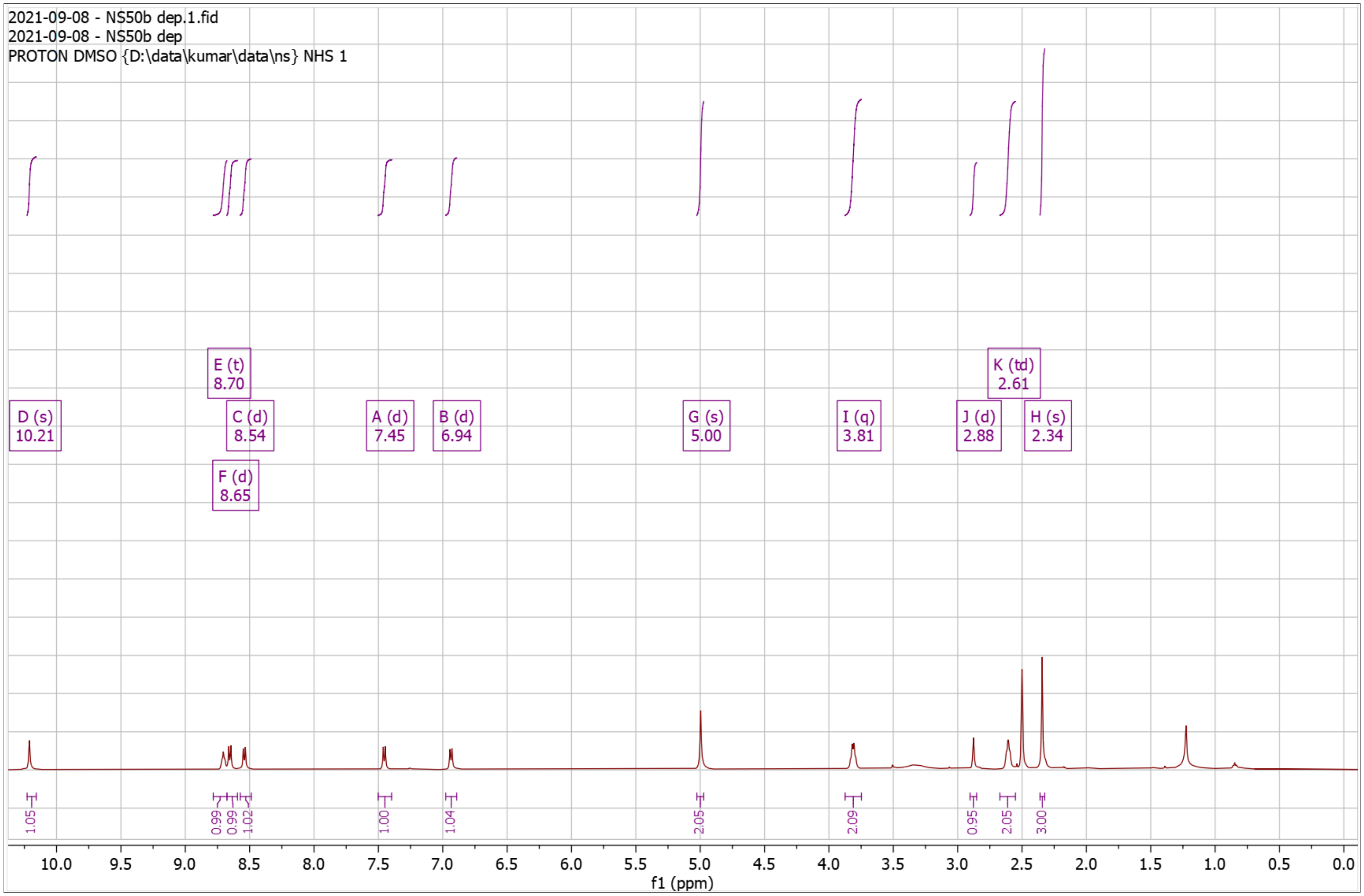

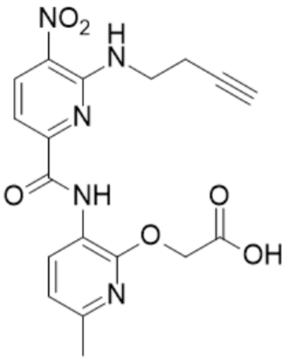

Supplementary Fig. 57. ^{1}H-NMR of NS52 Pro

^{1}H NMR (500 MHz, $CDCl_3$) δ 1.03 – 1.12 (t, *J* = 7.4 Hz, 3H), 1.44 – 1.49 (s, 9H), 1.73 – 1.87 (h, *J* = 7.3 Hz, 2H), 2.37 – 2.41 (s, 3H), 3.68 – 3.79 (td, *J* = 7.0, 5.6 Hz, 2H), 4.84 – 4.93 (s, 2H), 6.78 – 6.89 (d, *J* = 7.9 Hz, 1H), 7.50 – 7.59 (d, *J* = 8.4 Hz, 1H), 8.23 – 8.32 (t, 1H), 8.57 – 8.64 (d, *J* = 8.4 Hz, 1H), 8.65 – 8.72 (d, *J* = 7.9 Hz, 1H), 10.18 – 10.36 (s, 1H); HRMS (m/z): [M]+ calcd. for $C_{21}H_{27}N_5O_6$, 446.2034; found, 446.2037.

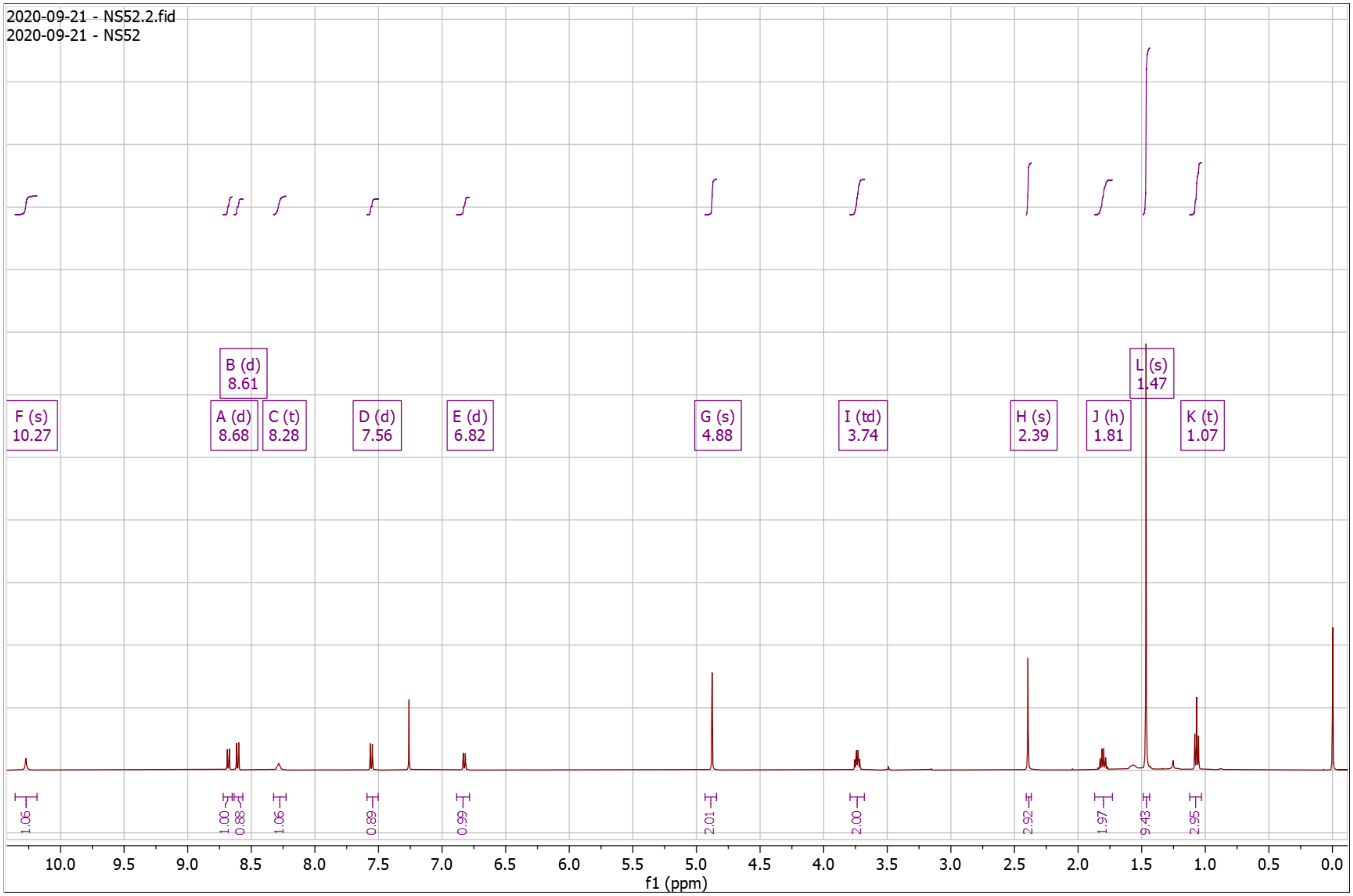

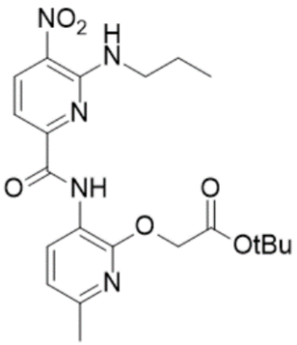

Supplementary Fig. 58. ^{1}H-NMR of NS52 Dep

^{1}H NMR (500 MHz, DMSO) δ 0.89 – 0.98 (t, *J* = 7.4 Hz, 3H), 1.63 – 1.77 (h, *J* = 7.4 Hz, 2H), 2.28 – 2.40 (s, 3H), 3.55 – 3.68 (q, *J* = 6.6 Hz, 2H), 4.89 – 5.05 (s, 2H), 6.90 – 7.04 (d, *J* = 7.9 Hz, 1H), 7.35 – 7.50 (d, *J* = 8.2 Hz, 1H), 8.52 – 8.59 (d, *J* = 7.9 Hz, 1H), 8.59 – 8.64 (t, 1H), 8.64 – 8.69 (d, *J* = 8.3 Hz, 1H), 10.12 – 10.29 (s, 1H), 12.74 – 13.08 (s, 1H); HRMS (m/z): [M]+ calcd. for $C_{17}H_{19}N_5O_6$, 390.1408; found, 390.1403.

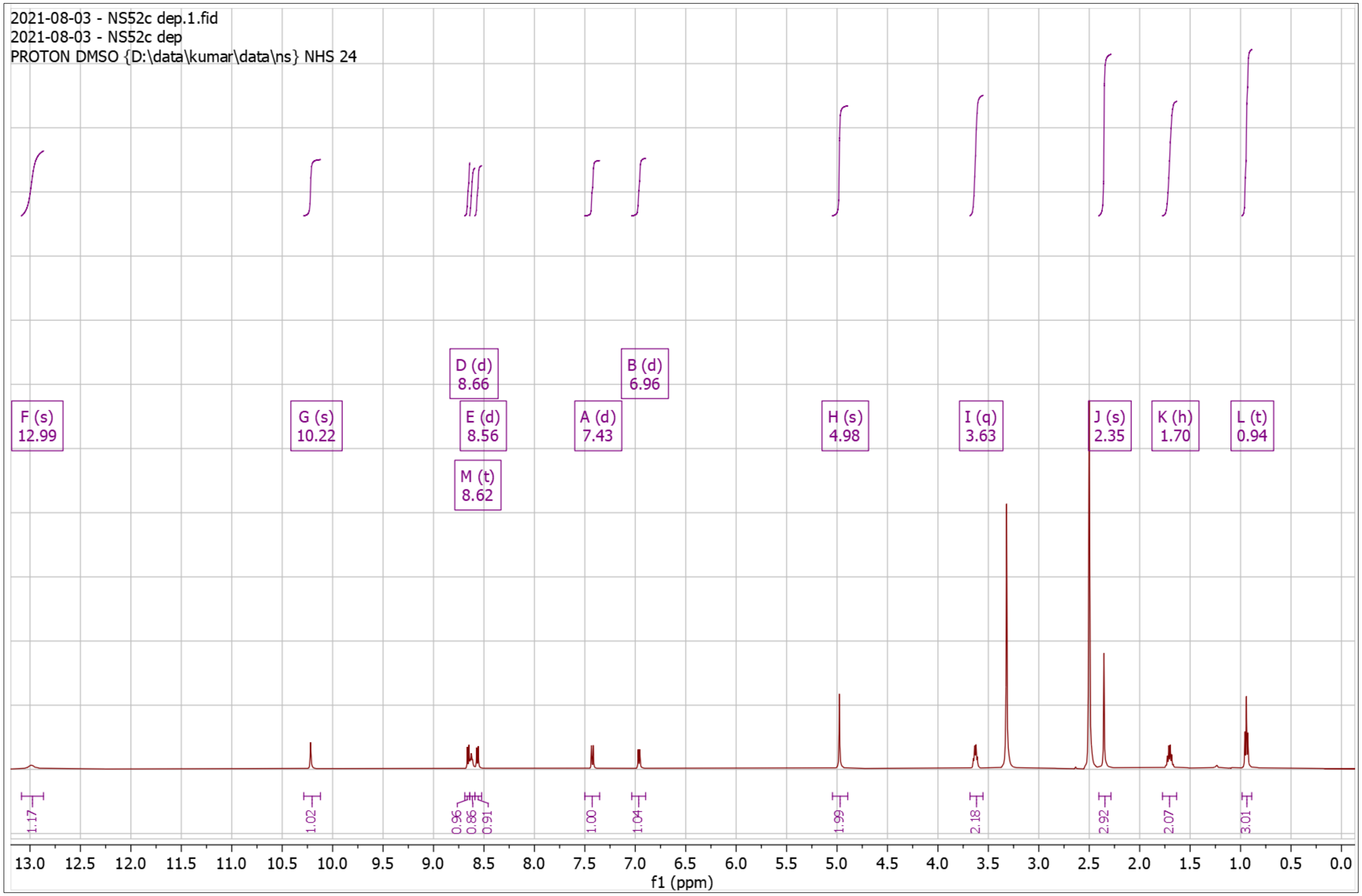

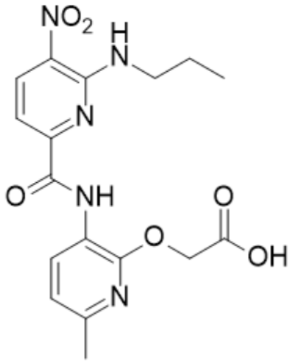

Supplementary Fig. 59. ^{1}H-NMR of NS53 Pro

^{1}H NMR (500 MHz, $CDCl_3$) δ 1.46 – 1.50 (s, 9H), 2.38 – 2.42 (s, 3H), 4.81 – 4.85 (s, 2H), 4.93 – 4.98 (d, *J* = 5.5 Hz, 2H), 6.30 – 6.34 (d, *J* = 2.2 Hz, 1H), 6.80 – 6.85 (d, *J* = 7.9 Hz, 1H), 7.47 – 7.51 (d, *J* = 2.2 Hz, 1H), 7.56 – 7.61 (d, *J* = 8.4 Hz, 1H), 8.47 – 8.52 (d, *J* = 7.9 Hz, 1H), 8.57 – 8.62 (d, *J* = 8.5 Hz, 1H), 8.62 – 8.67 (t, *J* = 5.4 Hz, 1H), 10.31 – 10.35 (s, 1H); HRMS (m/z): [M]+ calcd. for $C_{22}H_{25}N_7O_6$, 484.1939; found, 484.1934.

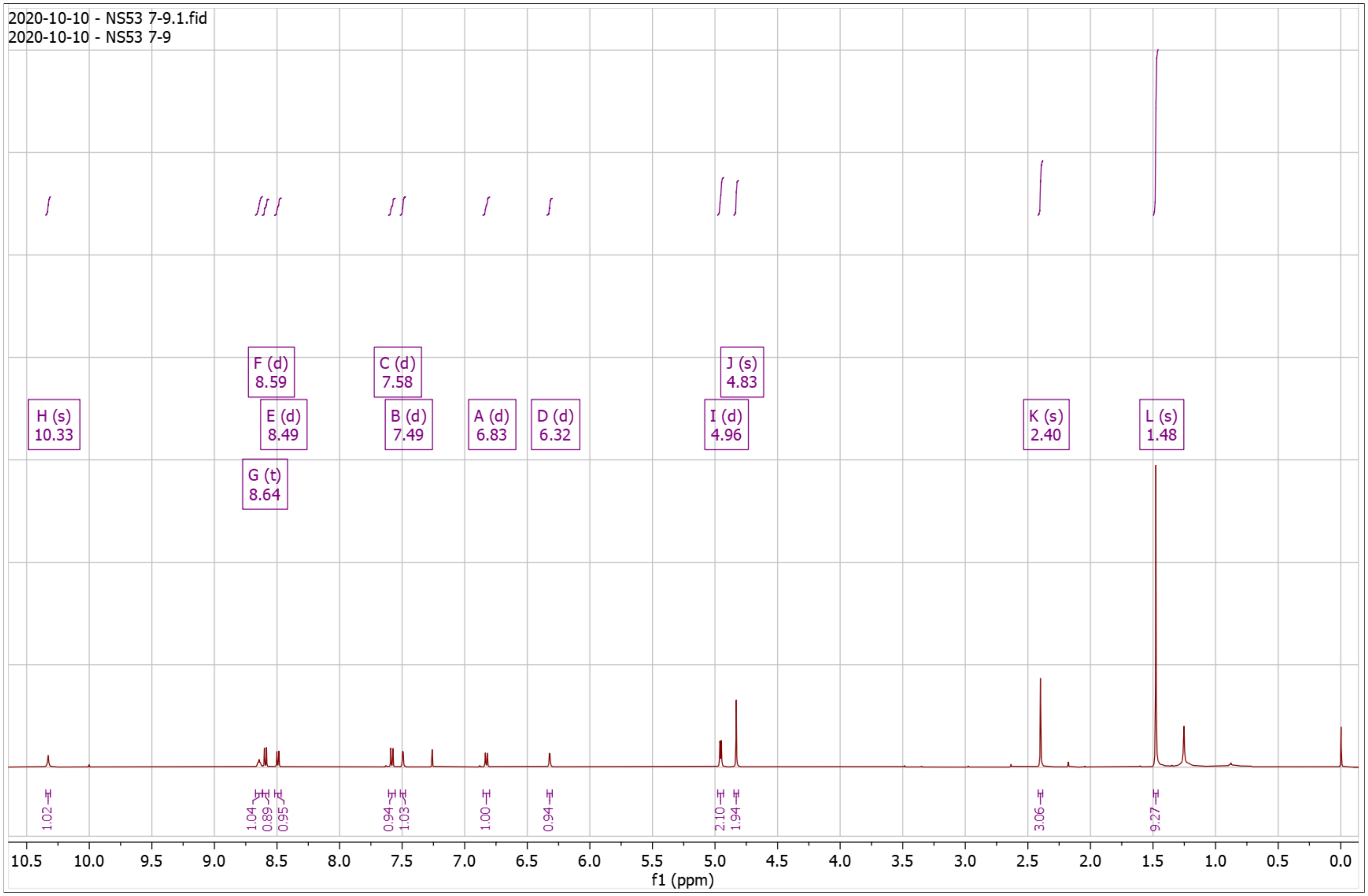

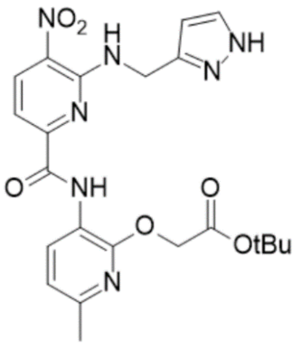

Supplementary Fig. 60. ^{1}H-NMR of NS53 Dep

^{1}H NMR (500 MHz, DMSO) δ 2.34 – 2.37 (s, 3H), 4.87 – 4.90 (s, 3H), 6.22 – 6.31 (s, 1H), 6.89 – 7.00 (d, *J* = 7.9 Hz, 1H), 7.42 – 7.51 (d, *J* = 8.4 Hz, 1H), 7.54 – 7.63 (s, 1H), 8.41 – 8.54 (d, *J* = 7.9 Hz, 1H), 8.64 – 8.73 (d, *J* = 8.4 Hz, 1H), 8.88 – 9.06 (t, *J* = 5.8 Hz, 1H), 10.15 – 10.39 (s, 1H); HRMS (m/z): [M]+ calcd. for $C_{18}H_{17}N_7O_6$, 428.1313; found, 428.1313.

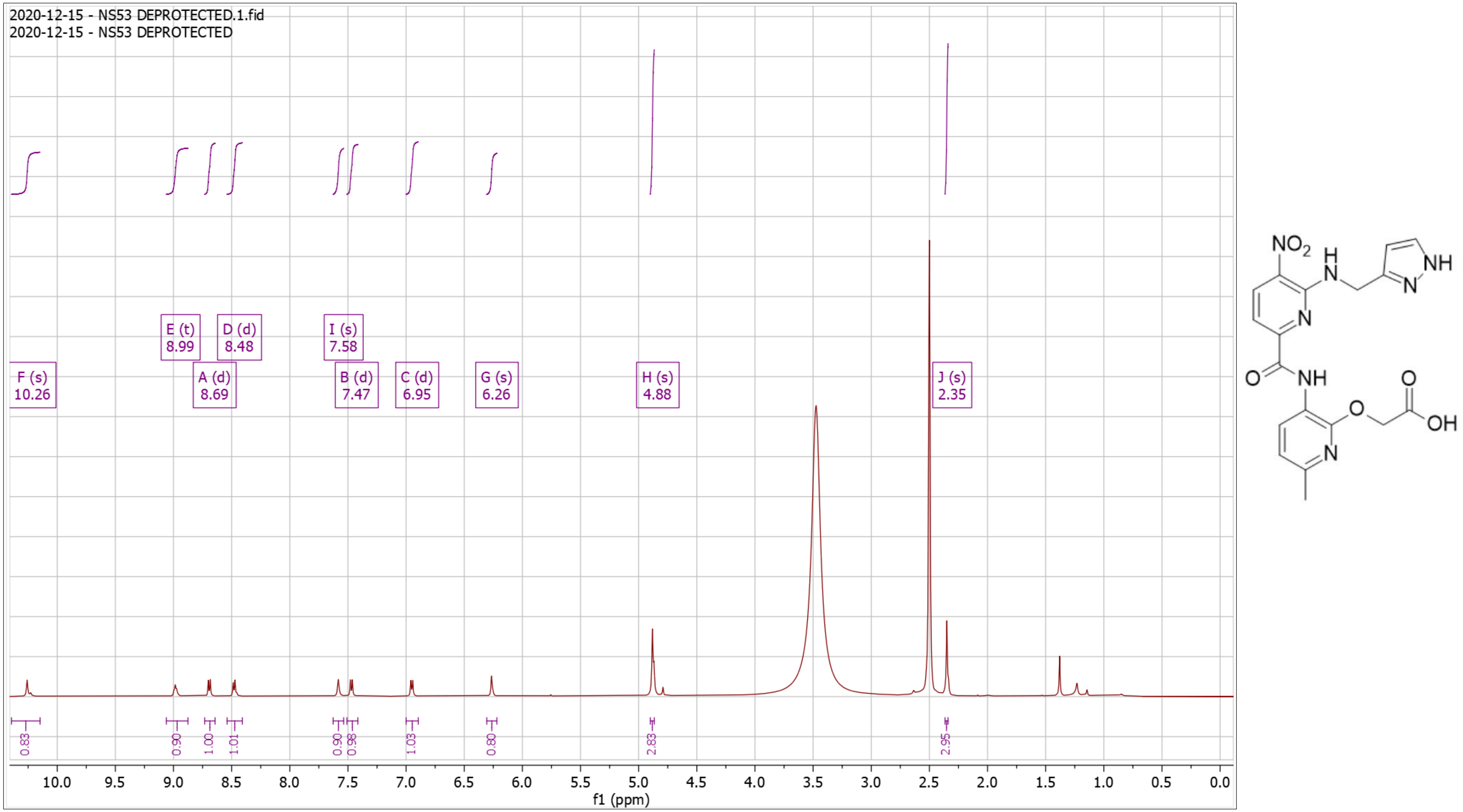

Supplementary Fig. 61. ^{1}H-NMR of NS54 Pro

^{1}H NMR (500 MHz, $CDCl_3$) δ 0.35 – 0.40 (m, 2H), 0.60 – 0.66 (m, 2H), 1.26 – 1.32 (tdd, *J* = 8.0, 5.2, 1.9 Hz, 1H), 1.45 – 1.49 (s, 9H), 2.37 – 2.42 (s, 3H), 3.58 – 3.65 (dd, *J* = 7.2, 5.2 Hz, 2H), 4.87 – 4.91 (s, 2H), 6.80 – 6.85 (d, *J* = 7.9 Hz, 1H), 7.54 – 7.60 (d, *J* = 8.4 Hz, 1H), 8.29 – 8.38 (t, 1H), 8.58 – 8.64 (d, *J* = 8.4 Hz, 1H), 8.64 – 8.70 (d, *J* = 7.9 Hz, 1H), 10.25 – 10.34 (s, 1H); HRMS (m/z): [M]+ calcd. for $C_{22}H_{27}N_5O_6$, 458.2034; found, 458.2034.

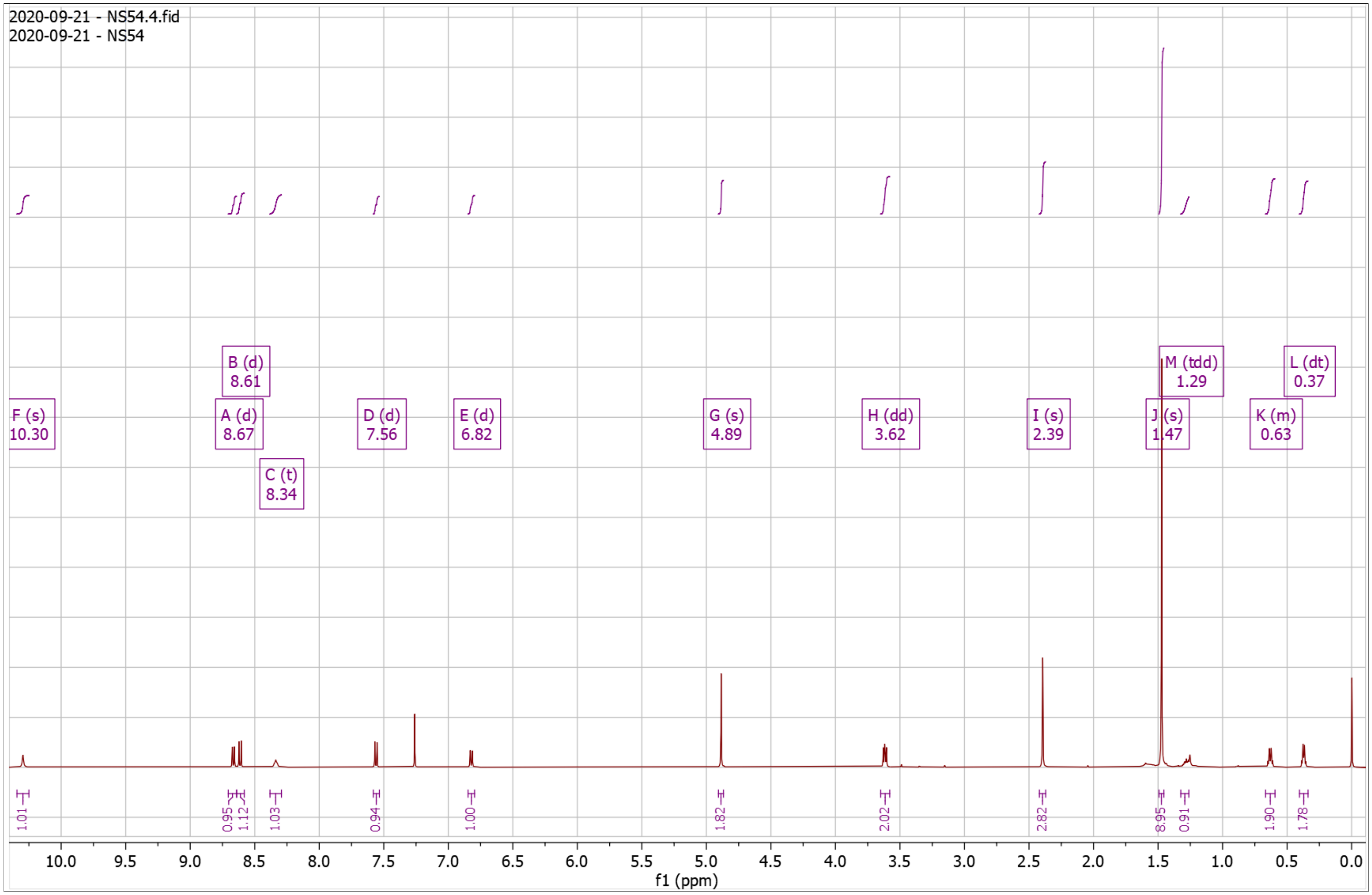

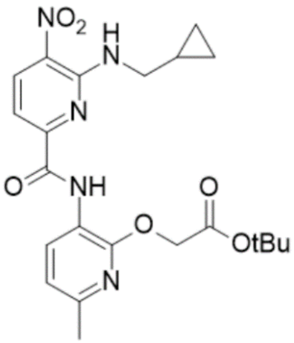

Supplementary Fig. 62. ^{1}H-NMR of NS54 Dep

^{1}H NMR (500 MHz, DMSO) δ 0.32 – 0.38 (q, *J* = 4.8 Hz, 2H), 0.42 – 0.50 (d, *J* = 7.6 Hz, 2H), 1.26 – 1.34 (m, 1H), 2.33 – 2.38 (s, 3H), 3.48 – 3.56 (t, *J* = 6.1 Hz, 2H), 4.96 – 5.01 (s, 2H), 6.91 – 7.01 (d, *J* = 7.9 Hz, 1H), 7.38 – 7.49 (d, *J* = 8.3 Hz, 1H), 8.50 – 8.59 (d, *J* = 7.8 Hz, 1H), 8.63 – 8.69 (d, *J* = 8.4 Hz, 1H), 8.69 – 8.77 (t, *J* = 6.0 Hz, 1H), 10.29 – 10.34 (s, 1H); HRMS (m/z): [M]+ calcd. for $C_{18}H_{19}N_5O_6$, 402.1408; found, 402.1402.

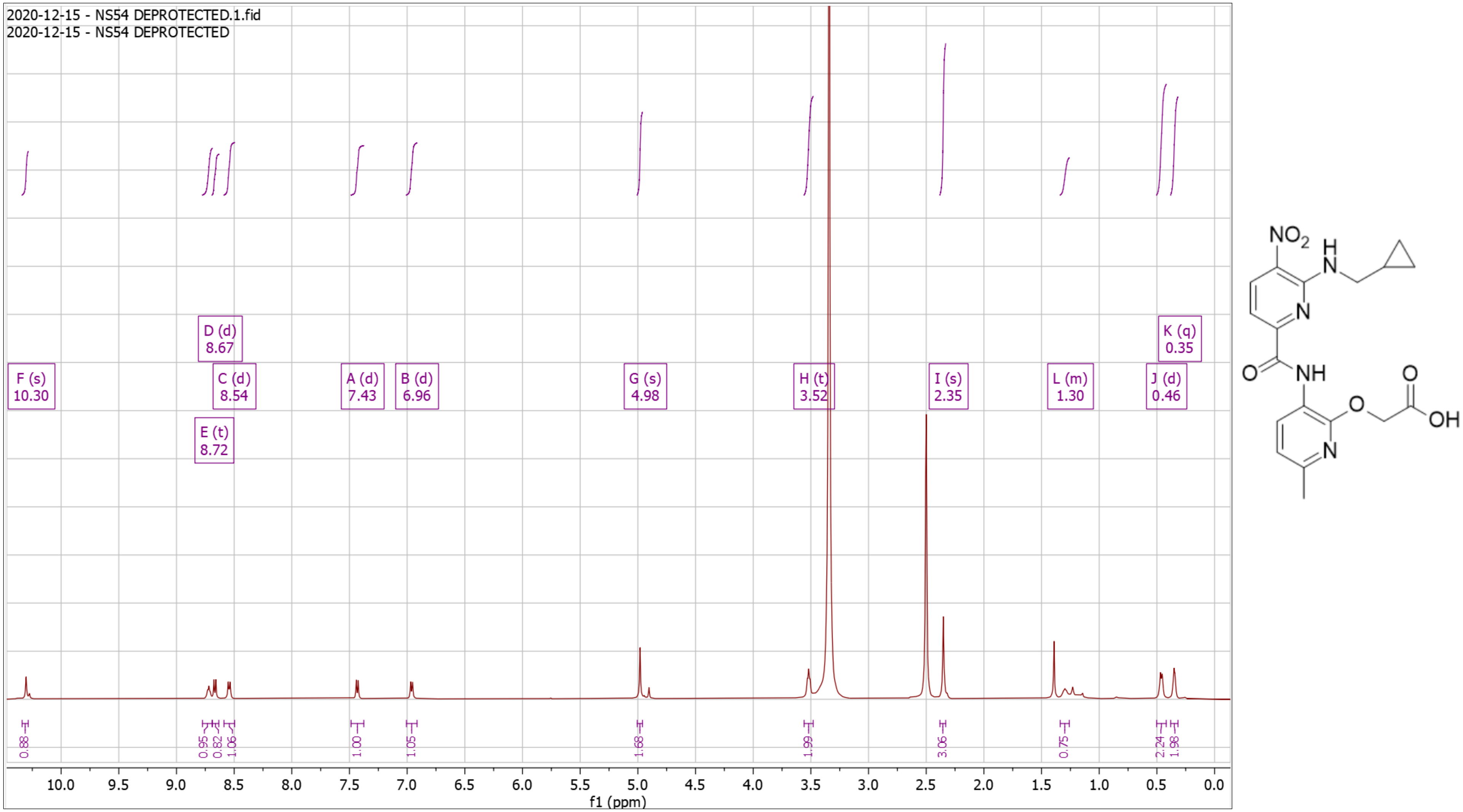

Supplementary Fig. 63. ^{1}H-NMR of NS55 Pro

^{1}H NMR (500 MHz, $CDCl_3$) δ 1.01 – 1.34 (m, 5H), 1.46 – 1.46 (s, 9H), 1.66 – 1.91 (m, 6H), 2.37 – 2.41 (s, 3H), 3.59 – 3.65 (t, J = 6.3 Hz, 2H), 4.84 – 4.89 (s, 2H), 6.79 – 6.85 (d, J = 7.9 Hz, 1H), 7.50 – 7.57 (d, J = 8.4 Hz, 1H), 8.31 – 8.37 (t, J = 5.8 Hz, 1H), 8.57 – 8.62 (d, J = 8.4 Hz, 1H), 8.66 – 8.71 (d, J = 8.0 Hz, 1H), 10.21 – 10.24 (s, 1H); HRMS (m/z): [M]+ calcd. for $C_{25}H_{33}N_5O_6$, 500.2504; found, 500.2500.

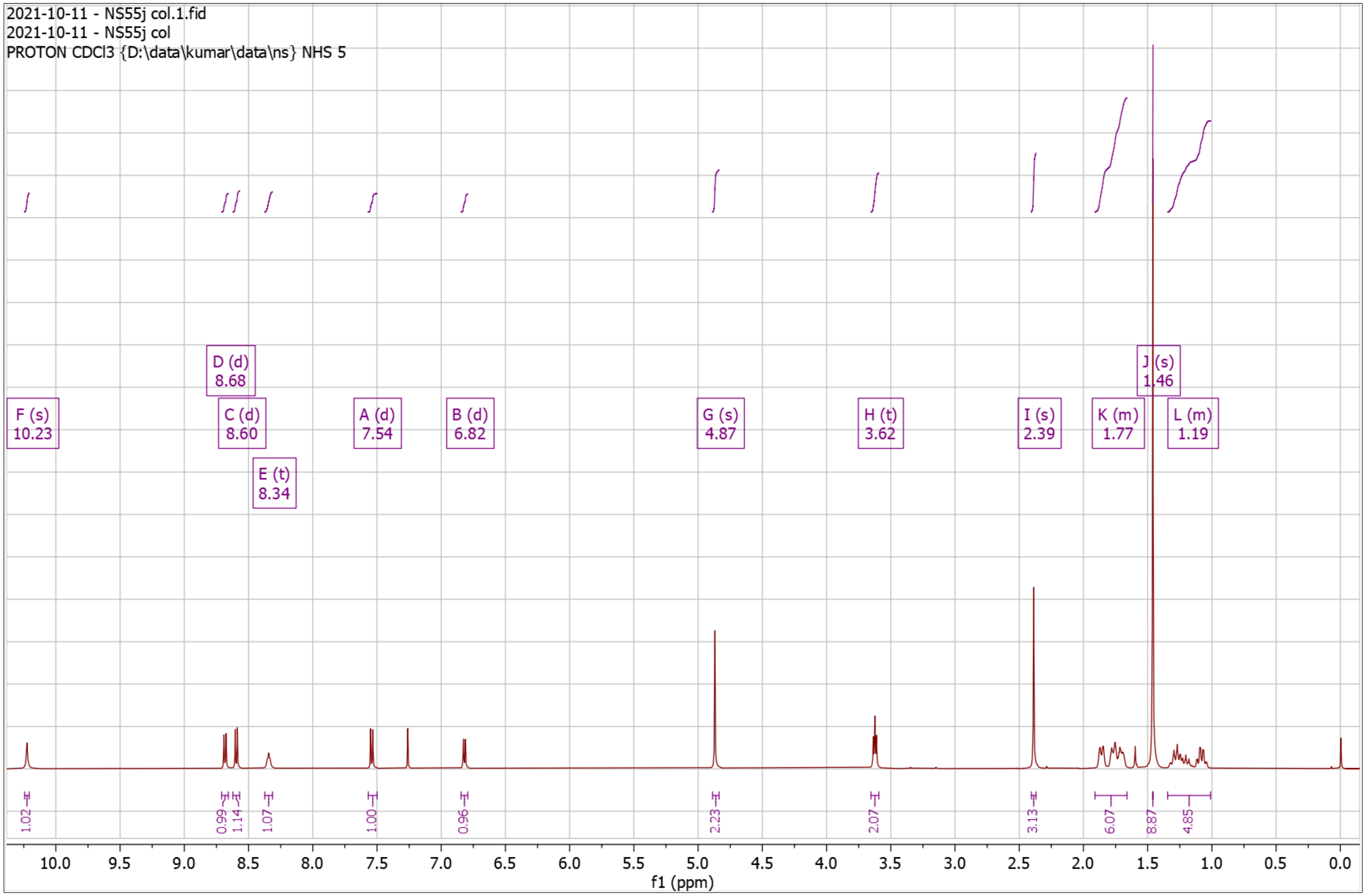

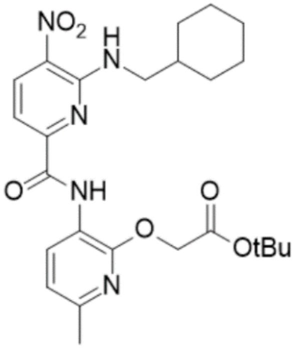

Supplementary Fig. 64. ^{1}H-NMR of NS55 Dep

^{1}H NMR (500 MHz, DMSO) δ 10.19 – 10.10 (s, 1H), 8.67 – 8.62 (d, *J* = 8.4 Hz, 1H), 8.60 – 8.50 (m, 2H), 7.48 – 7.39 (d, *J* = 8.4 Hz, 1H), 6.99 – 6.93 (d, *J* = 8.0 Hz, 1H), 4.99 – 4.95 (s, 2H), 3.59 – 3.53 (t, *J* = 6.4 Hz, 2H), 2.37 – 2.33 (s, 3H), 1.81 – 1.63 (m, 6H), 1.24 – 0.94 (m, 5H); HRMS (m/z): [M]+ calcd. for $C_{21}H_{25}N_5O_6$, 444.1878; found, 444.1878.

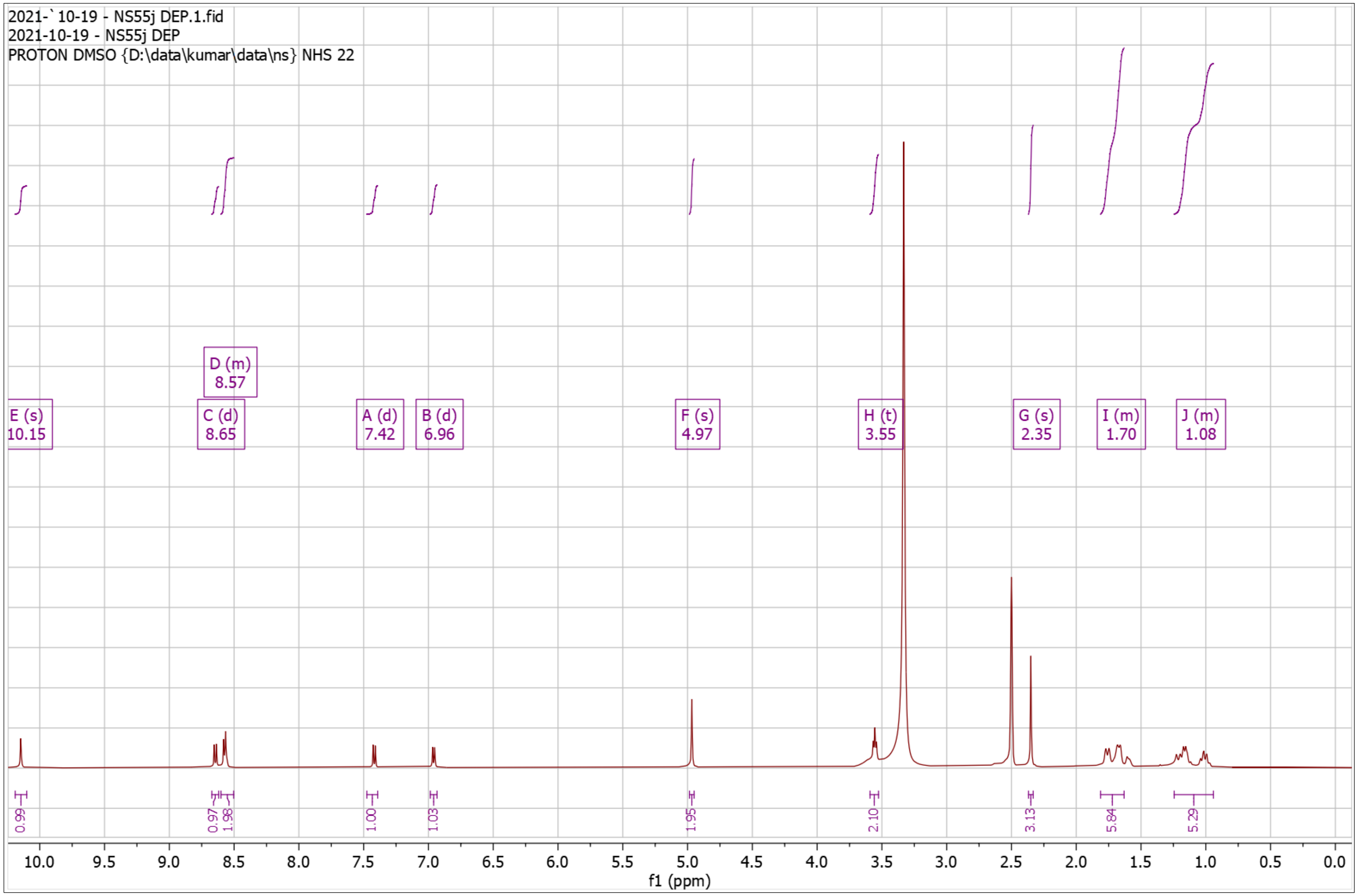

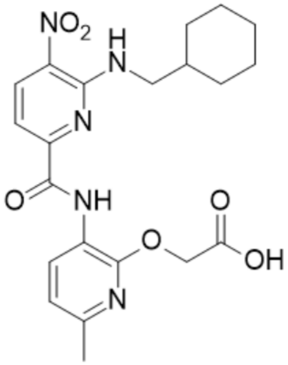

Supplementary Fig. 65. ^{13}C-NMR of NS55 Dep

^{13}C NMR (126 MHz, DMSO) δ 23.27, 25.47, 25.94, 30.45, 37.46, 46.79, 62.14, 109.03, 116.85, 118.94, 127.13, 129.82, 138.05, 149.60, 150.92, 151.09, 152.25, 160.39, 169.73.

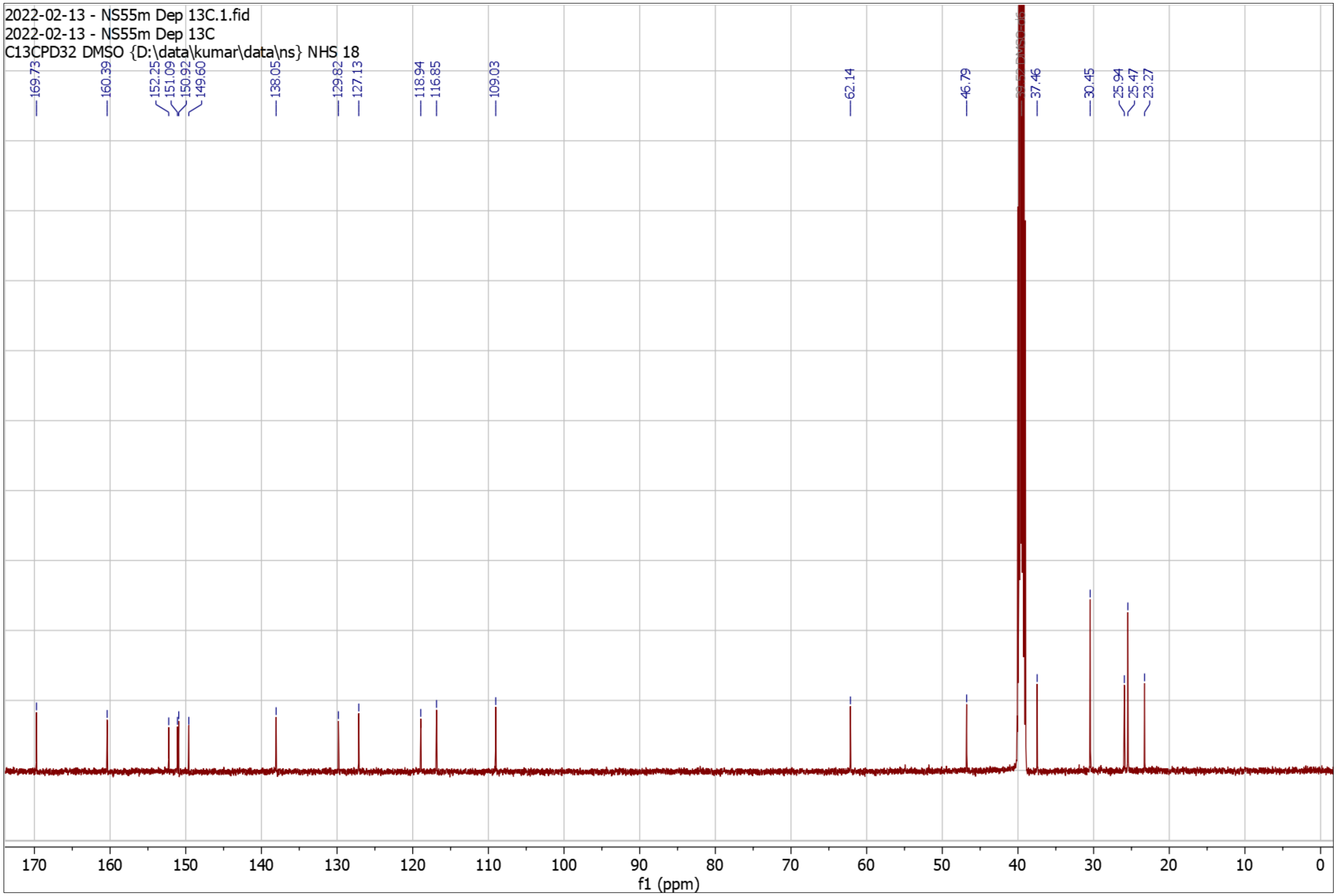

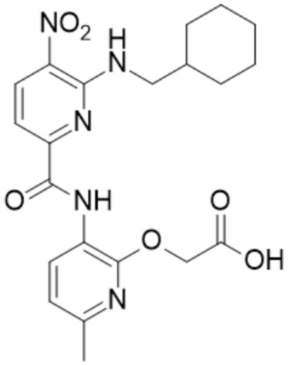

Supplementary Fig. 66. ^{1}H-NMR of NS56 Pro

^{1}H NMR (500 MHz, $CDCl_3$) δ 1.46 – 1.46 (s, 9H), 2.34 – 2.44 (s, 3H), 3.02 – 3.12 (t, *J* = 7.1 Hz, 2H), 3.97 – 4.12 (td, *J* = 7.1, 5.4 Hz, 2H), 4.62 – 4.69 (s, 2H), 6.77 – 6.86 (d, *J* = 7.9 Hz, 1H), 7.20 – 7.36 (m, 5H), 7.53 – 7.59 (d, *J* = 8.5 Hz, 1H), 8.21 – 8.30 (t, *J* = 5.3 Hz, 1H), 8.54 – 8.63 (d, *J* = 8.3 Hz, 1H), 8.64 – 8.73 (d, *J* = 7.9 Hz, 1H), 10.20 – 10.32 (s, 1H); HRMS (m/z): [M]+ calcd. for $C_{26}H_{29}N_5O_6$, 508.2191 ; found, 508.2190.

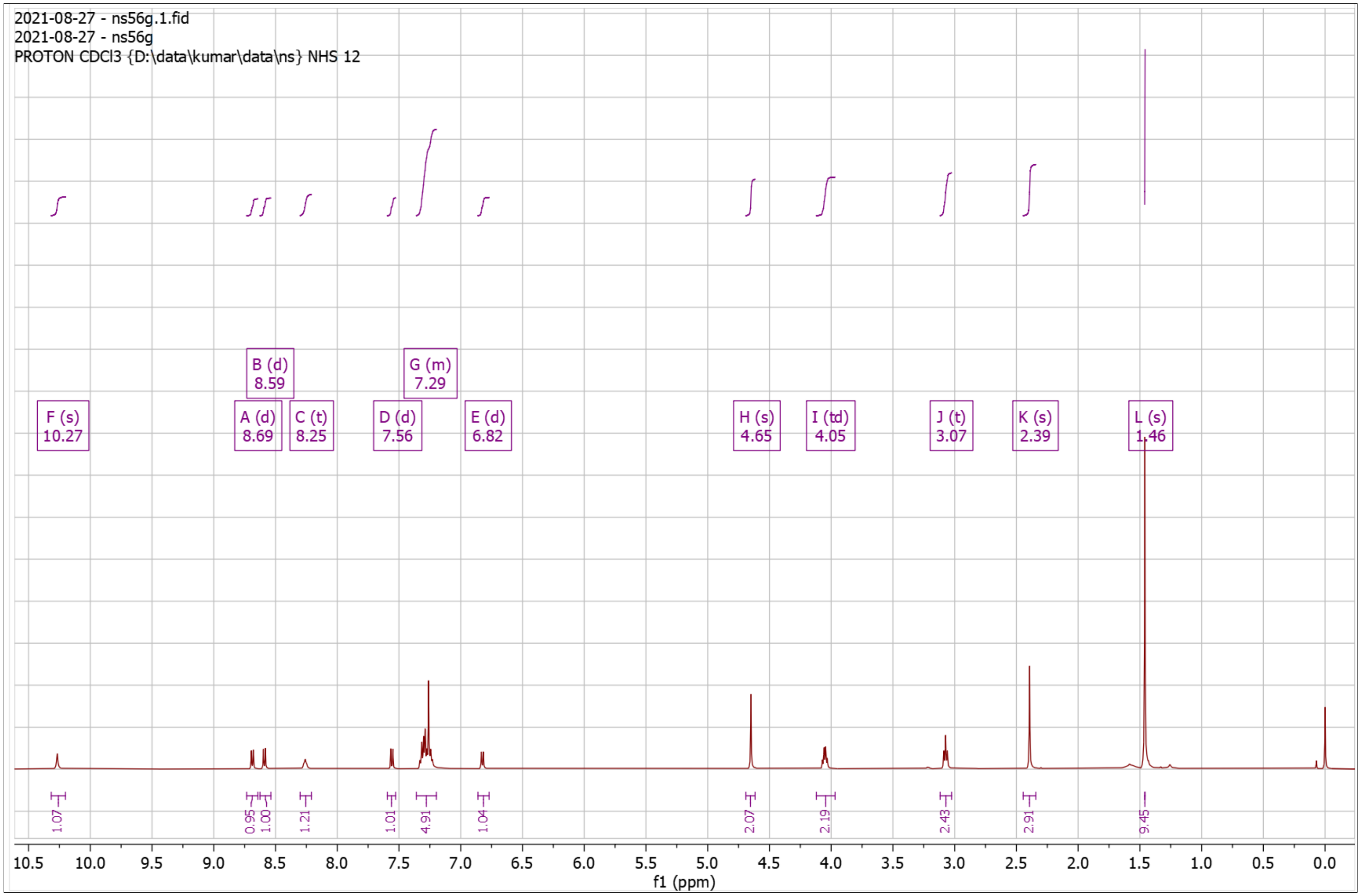

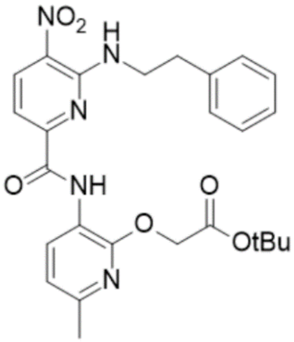

Supplementary Fig. 67. ^{1}H-NMR of NS56 Dep

^{1}H NMR (500 MHz, DMSO) δ 2.31 – 2.38 (s, 3H), 2.95 – 3.07 (t, *J* = 7.1 Hz, 2H), 3.88 – 4.00 (q, *J* = 6.7 Hz, 2H), 4.69 – 4.77 (s, 2H), 6.90 – 6.99 (d, *J* = 8.0 Hz, 1H), 7.12 – 7.29 (m, 5H), 7.36 – 7.44 (d, *J* = 8.4 Hz, 1H), 8.48 – 8.57 (d, *J* = 7.9 Hz, 1H), 8.57 – 8.61 (t, *J* = 5.8 Hz, 1H), 8.61 – 8.67 (d, *J* = 8.4 Hz, 1H), 10.10 – 10.17 (s, 1H); HRMS (m/z): [M]+ calcd. for $C_{22}H_{21}N_5O_6$, 452.1565; found, 452.1565.

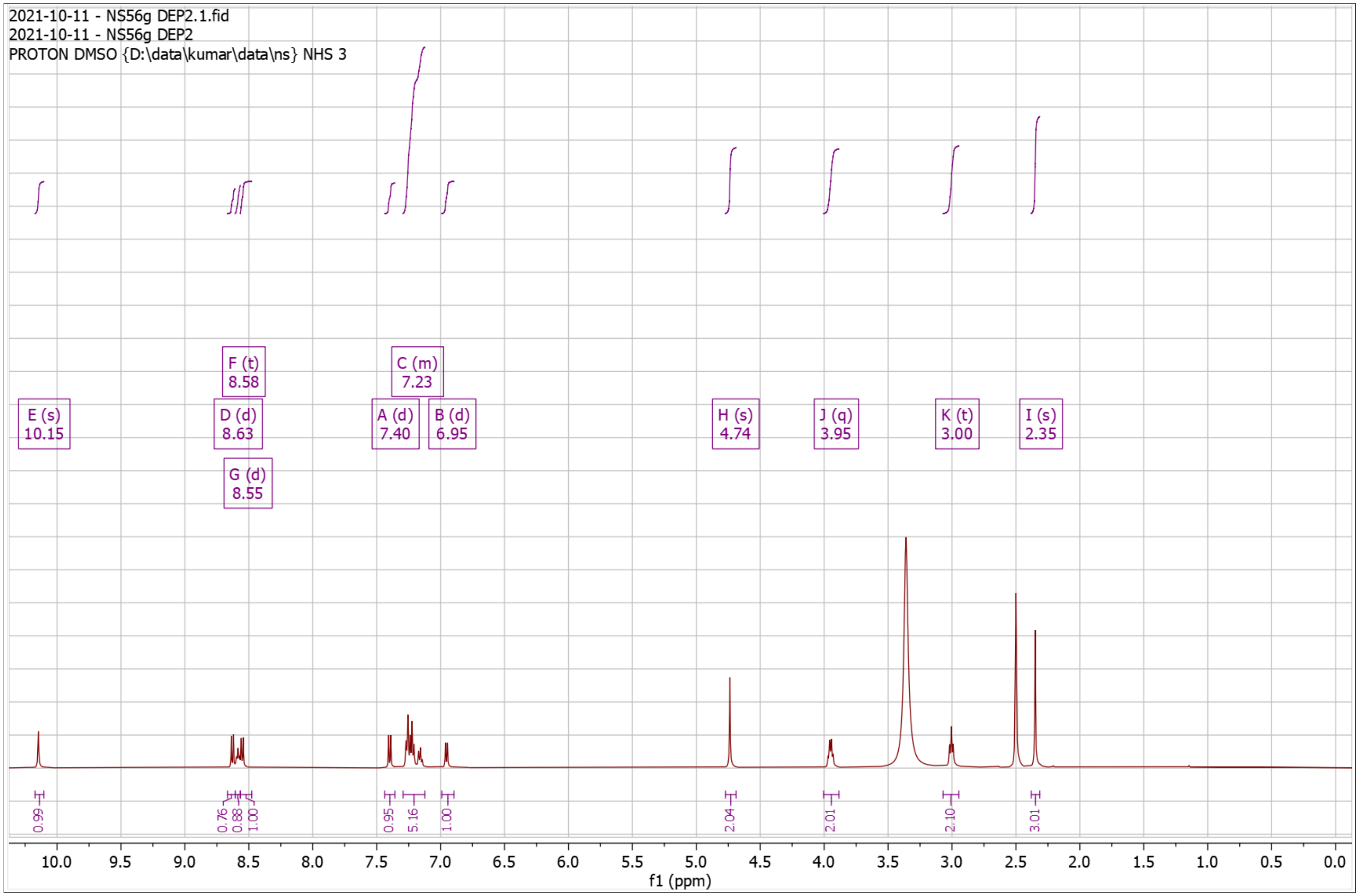

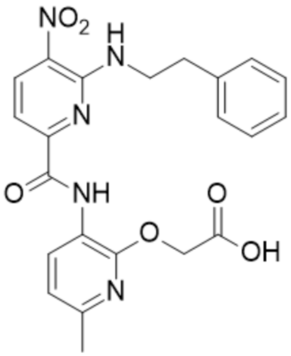

Supplementary Fig. 68. ^{1}H-NMR of NS57 Pro

^{1}H NMR (500 MHz, $CDCl_3$) δ 1.44 – 1.47 (s, 9H), 1.47 – 1.51 (t, *J* = 7.4 Hz, 3H), 2.26 – 2.50 (s, 3H), 3.27 – 3.47 (q, *J* = 7.4 Hz, 2H), 4.80 – 4.97 (s, 2H), 6.75 – 6.94 (d, *J* = 7.9 Hz, 1H), 8.05 – 8.13 (d, 1H), 8.56 – 8.79 (dd, *J* = 8.2, 5.9 Hz, 2H), 10.21 – 10.35 (s, 1H); HRMS (m/z): [M]+ calcd. for $C_{20}H_{24}N_4O_6S$, 449.1489; found, 449.1488.

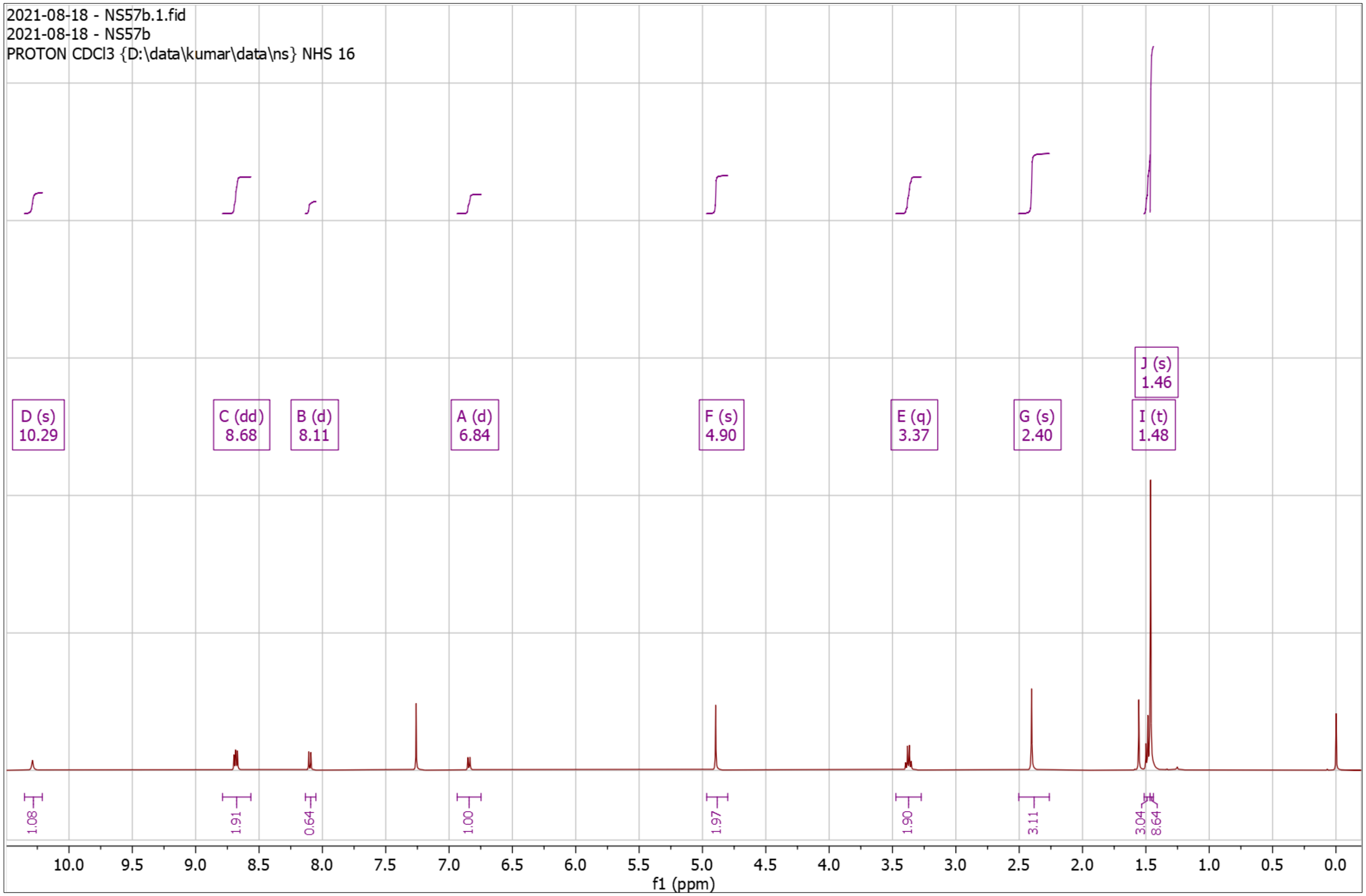

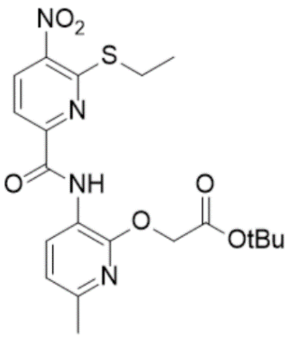

Supplementary Fig. 69. ^{1}H-NMR of NS57 Dep

^{1}H NMR (500 MHz, DMSO) δ 1.32 – 1.36 (t, *J* = 7.3 Hz, 3H), 2.31 – 2.37 (s, 3H), 3.09 – 3.20 (q, *J* = 7.3 Hz, 2H), 4.92 – 4.97 (s, 2H), 6.88 – 6.99 (d, *J* = 7.9 Hz, 1H), 7.99 – 8.06 (d, *J* = 8.4 Hz, 1H), 8.06 – 8.14 (d, *J* = 8.1 Hz, 1H), 8.46 – 8.54 (d, *J* = 7.9 Hz, 1H), 9.81 – 9.88 (s, 1H); HRMS (m/z): [M]+ calcd. for $C_{16}H_{16}N_4O_6S$, 393.0863; found, 393.0862.

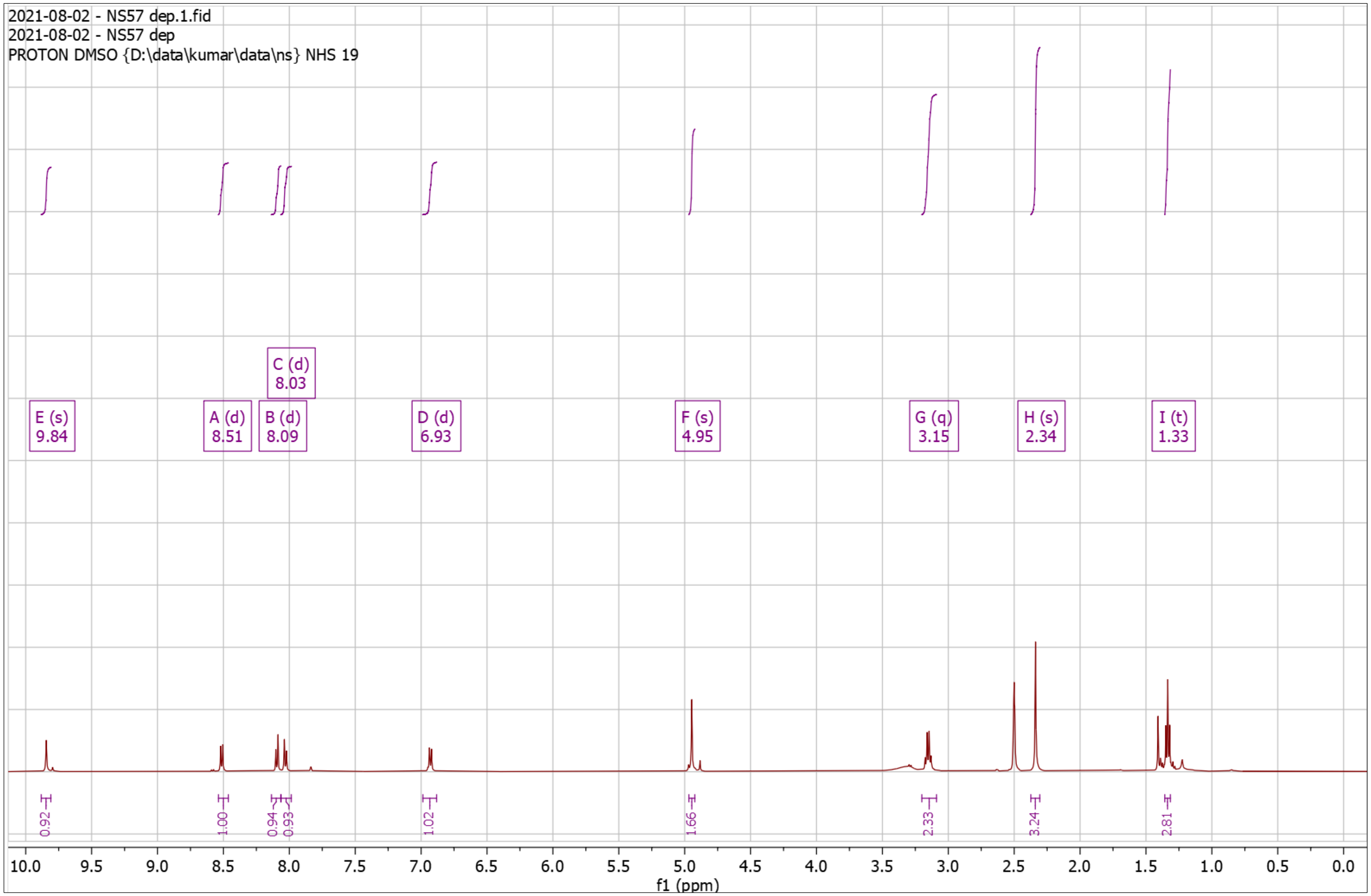

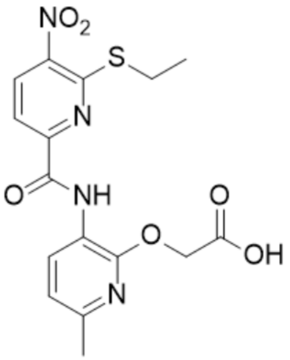

Supplementary Fig. 70. ^{1}H-NMR of NS59 Pro

^{1}H NMR (500 MHz, $CDCl_3$) δ 1.10 – 1.15 (d, J = 6.6 Hz, 6H), 1.44 – 1.47 (s, 9H), 1.99 – 2.11 (hept, J = 6.7 Hz, 1H), 2.38 – 2.42 (s, 3H), 3.25 – 3.30 (d, J = 6.7 Hz, 2H), 4.86 – 4.90 (s, 2H), 6.78 – 6.89 (d, J = 8.0 Hz, 1H), 8.03 – 8.13 (d, J = 8.4 Hz, 1H), 8.62 – 8.67 (d, J = 8.3 Hz, 1H), 8.67 – 8.72 (d, J = 7.9 Hz, 1H), 10.12 – 10.15 (s, 1H); HRMS (m/z): [M]+ calcd. for $C_{22}H_{28}N_4O_6S$, 477.1802; found, 477.1803.

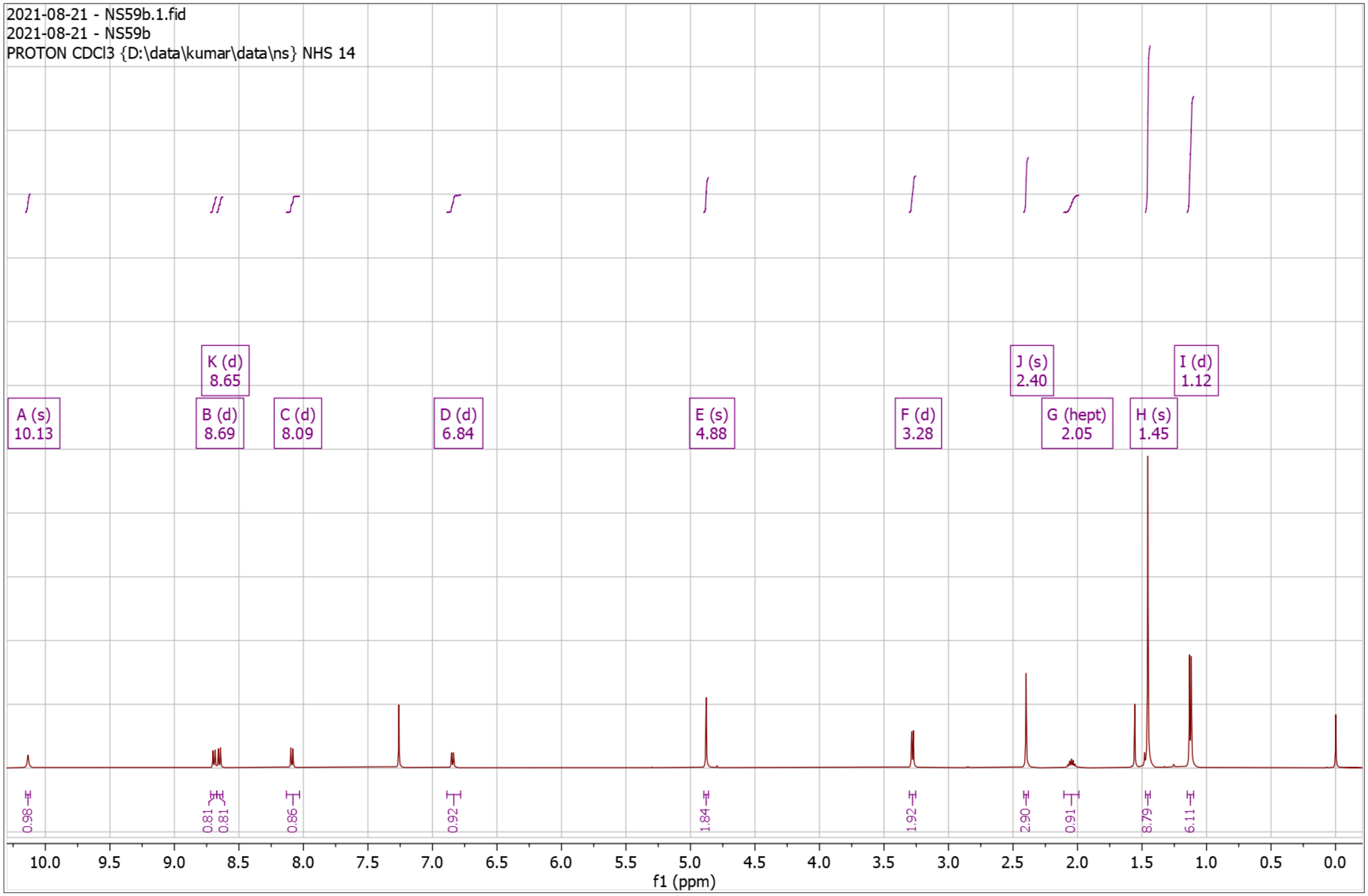

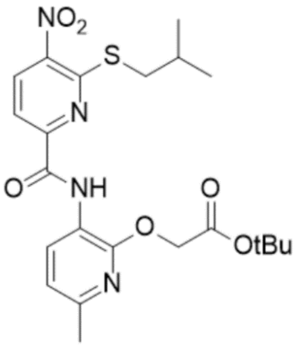

Supplementary Fig. 71. ^{1}H-NMR of NS59 Dep

^{1}H NMR (500 MHz, DMSO) δ 1.03 – 1.09 (d, J = 6.6 Hz, 6H), 1.89 – 1.99 (hept, J = 6.7 Hz, 1H), 2.31 – 2.36 (s, 3H), 3.01 – 3.05 (d, J = 6.7 Hz, 2H), 4.88 – 4.93 (s, 2H), 6.88 – 6.93 (d, J = 8.0 Hz, 1H), 8.01 – 8.06 (d, J = 8.2 Hz, 1H), 8.06 – 8.12 (d, J = 8.1 Hz, 1H), 8.47 – 8.55 (d, J = 7.9 Hz, 1H), 9.84 – 9.93 (s, 1H); HRMS (m/z): [M]+ calcd. for $C_{18}H_{20}N_4O_6S$, 421.1176; found, 421.1171.

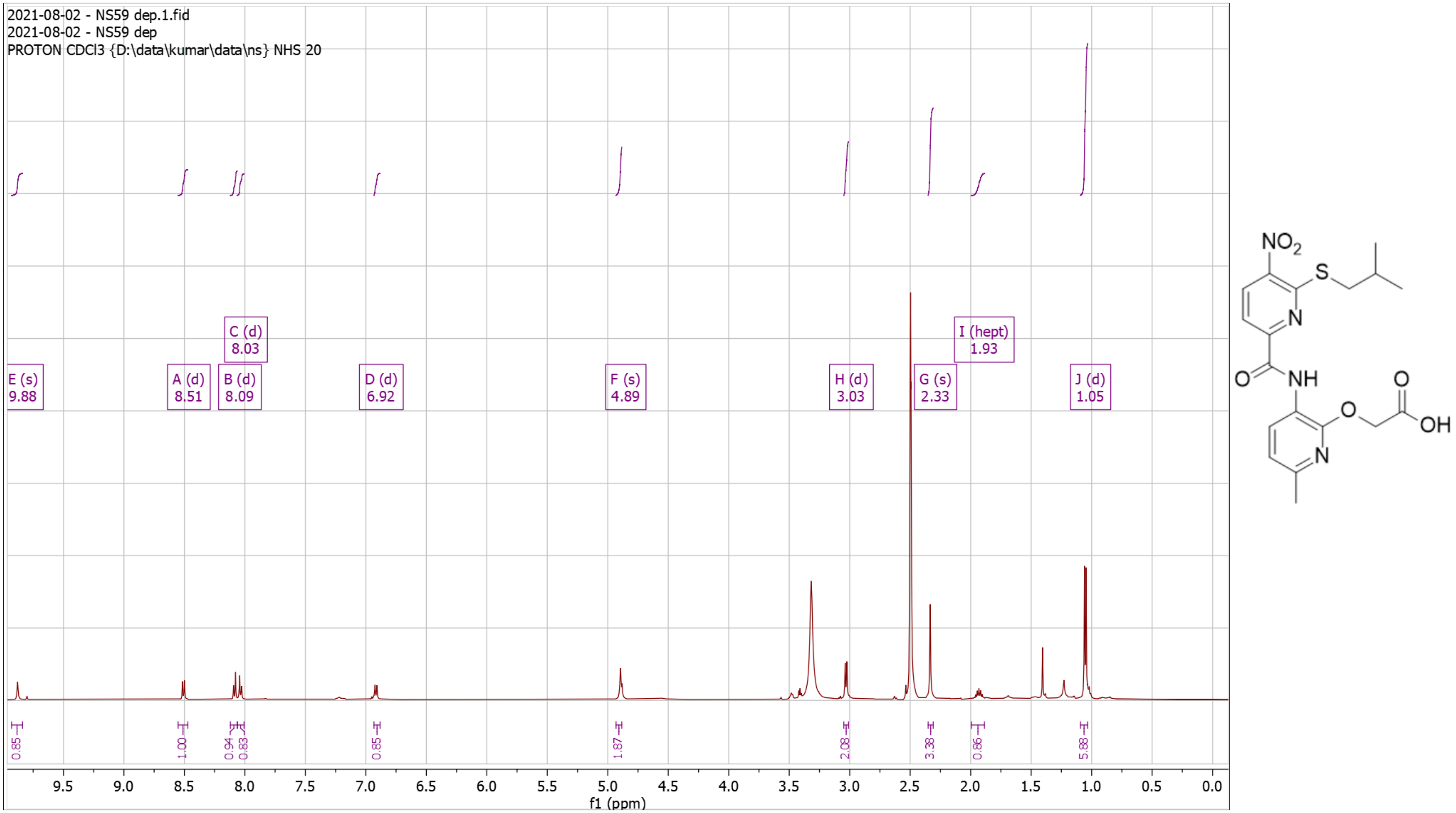

Supplementary Fig. 72. ^{1}H-NMR of NS60 Pro

^{1}H NMR (500 MHz, $CDCl_3$) δ 0.95 – 1.03 (t, *J* = 7.4 Hz, 3H), 1.46 – 1.49 (s, 9H), 1.49 – 1.61 (h, *J* = 7.3 Hz, 2H), 1.70 – 1.80 (ddd, *J* = 15.1, 8.6, 6.3 Hz, 2H), 2.35 – 2.39 (s, 3H), 2.95 – 3.01 (t, *J* = 7.4 Hz, 2H), 4.86 – 4.89 (s, 2H), 6.76 – 6.85 (d, *J* = 7.9 Hz, 1H), 7.58 – 7.66 (d, *J* = 8.1 Hz, 1H), 8.07 – 8.14 (d, *J* = 8.1 Hz, 1H), 8.61 – 8.67 (d, *J* = 7.8 Hz, 1H), 9.96 – 10.05 (s, 1H); HRMS (m/z): [M]+ calcd. for $C_{22}H_{28}N_4O_6S$, 477.1802; found, 477.1805.

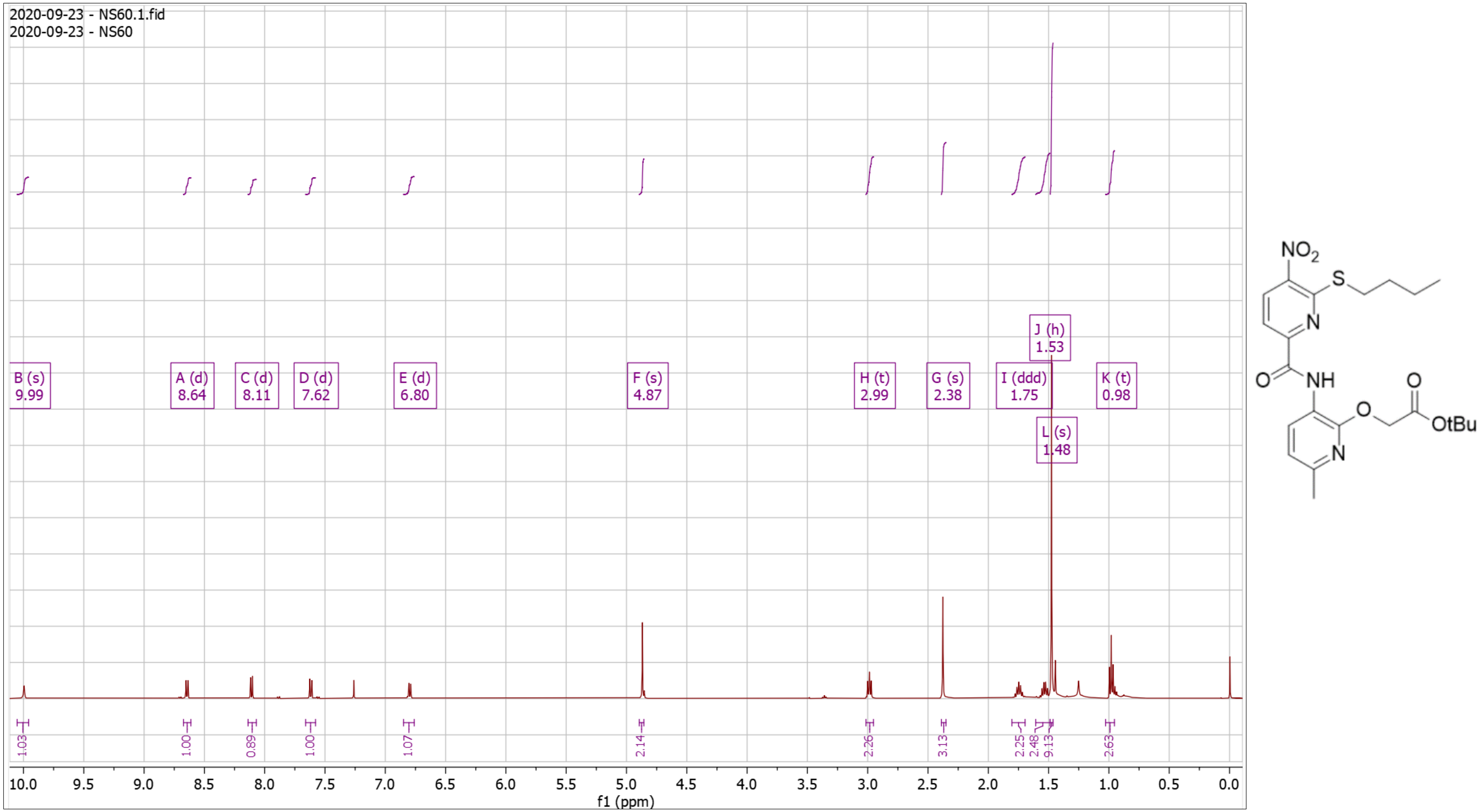

Supplementary Fig. 73. ^{1}H-NMR of NS60 Dep

^{1}H NMR (500 MHz, DMSO) δ 0.86 – 0.96 (t, J = 7.4 Hz, 3H), 1.41 – 1.50 (q, J = 7.4 Hz, 2H), 1.54 – 1.72 (p, J = 7.4 Hz, 2H), 2.26 – 2.39 (s, 3H), 3.03 – 3.16 (t, J = 7.3 Hz, 2H), 4.91 – 5.03 (s, 2H), 6.85 – 6.96 (d, J = 7.9 Hz, 1H), 7.95 – 8.03 (d, J = 8.3 Hz, 1H), 8.03 – 8.11 (d, J = 8.1 Hz, 1H), 8.44 – 8.55 (d, J = 7.9 Hz, 1H), 9.78 – 9.83 (s, 1H); HRMS (m/z): [M]+ calcd. for $C_{18}H_{20}N_4O_6S$, 421.1176; found, 421.1176.

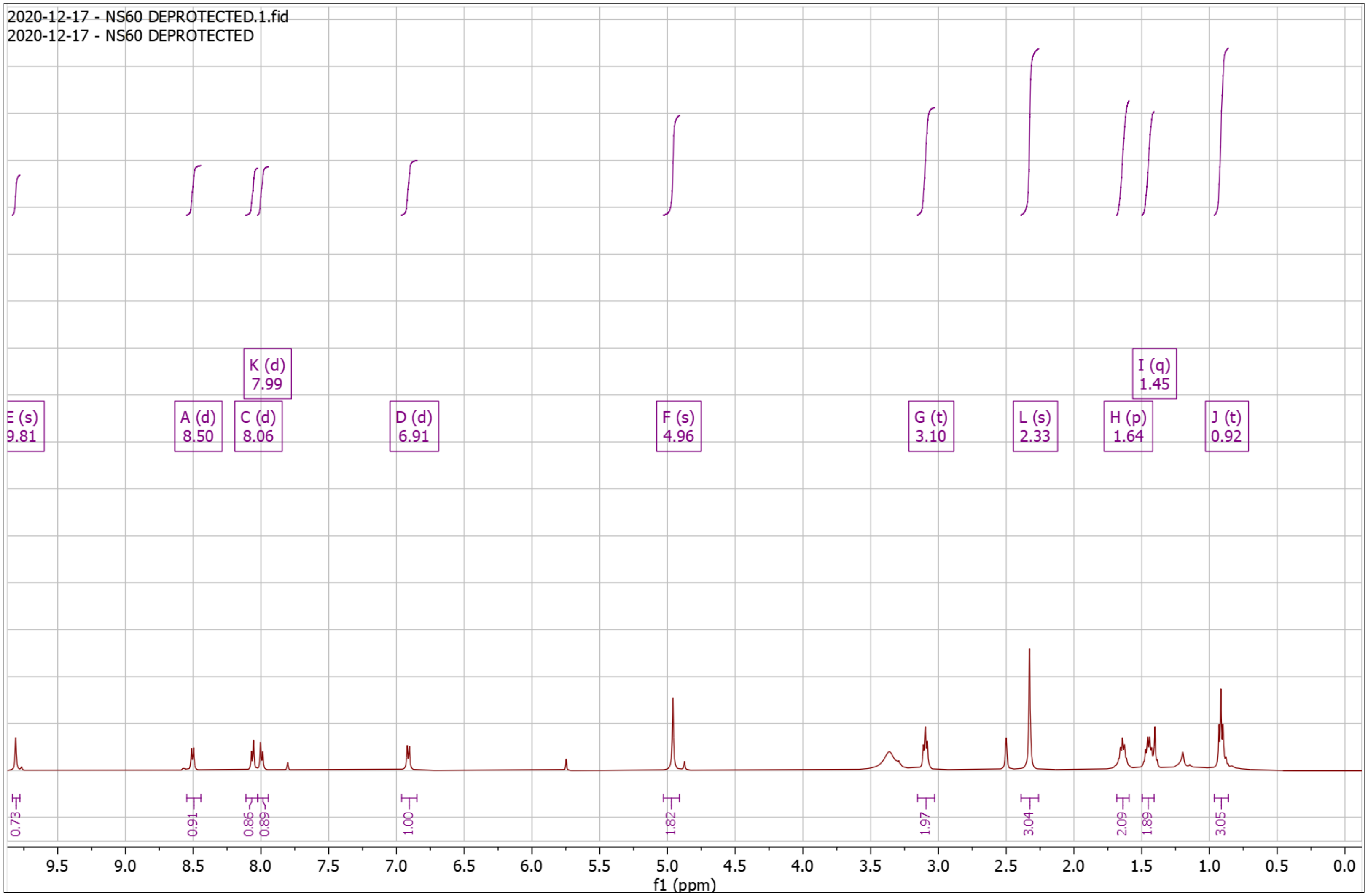

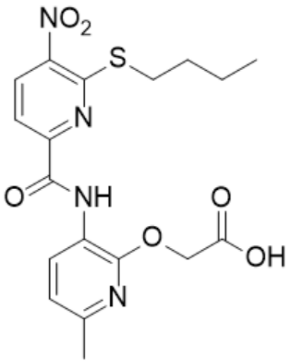

Supplementary Fig. 74. ^{1}H-NMR of NS61 Pro

^{1}H NMR (500 MHz, $CDCl_3$) δ 1.46 – 1.50 (s, 9H), 2.36 – 2.40 (s, 3H), 4.22 – 4.26 (s, 2H), 4.85 – 4.89 (s, 2H), 6.25 – 6.29 (d, *J* = 3.3 Hz, 1H), 6.29 – 6.35 (dd, *J* = 3.3, 1.9 Hz, 1H), 6.77 – 6.82 (d, *J* = 7.9 Hz, 1H), 7.35 – 7.41 (d, *J* = 1.9 Hz, 1H), 7.73 – 7.78 (d, *J* = 8.1 Hz, 1H), 8.07 – 8.12 (d, *J* = 8.0 Hz, 1H), 8.61 – 8.66 (d, *J* = 8.0 Hz, 1H), 9.98 – 10.01 (s, 1H); HRMS (m/z): [M]+ calcd. for $C_{23}H_{24}N_4O_7S$, 501.1439; found, 501.1434.

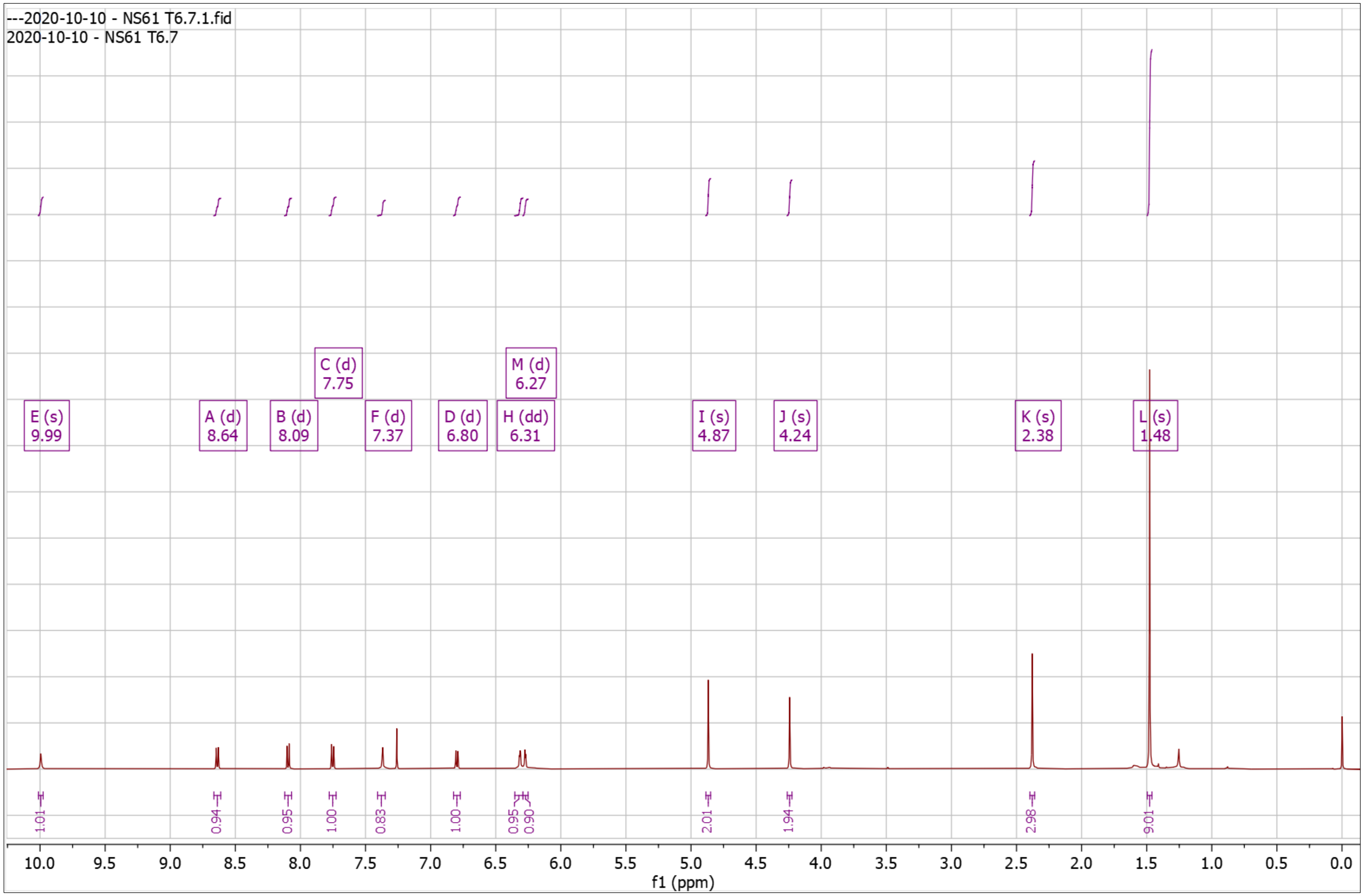

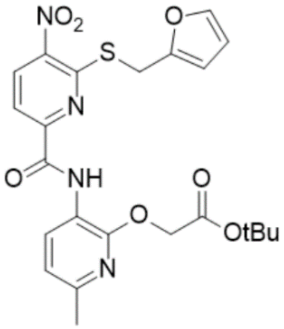

Supplementary Fig. 75. ^{1}H-NMR of NS61 Dep

^{1}H NMR (500 MHz, DMSO) δ 2.31 – 2.37 (s, 3H), 4.54 – 4.58 (s, 2H), 4.93 – 4.98 (s, 2H), 6.38 – 6.44 (dd, *J* = 3.2, 1.9 Hz, 1H), 6.44 – 6.50 (d, *J* = 3.3 Hz, 1H), 6.88 – 7.00 (d, *J* = 8.0 Hz, 1H), 7.58 – 7.65 (d, *J* = 1.9 Hz, 1H), 8.08 – 8.15 (d, *J* = 8.1 Hz, 1H), 8.17 – 8.27 (d, *J* = 8.2 Hz, 1H), 8.46 – 8.54 (d, *J* = 7.9 Hz, 1H), 9.80 – 9.89 (s, 1H), 12.77 – 12.97 (s, 1H); HRMS (m/z): [M]+ calcd. for $C_{19}H_{16}N_4O_7S$, 445.0813; found, 445.0821.

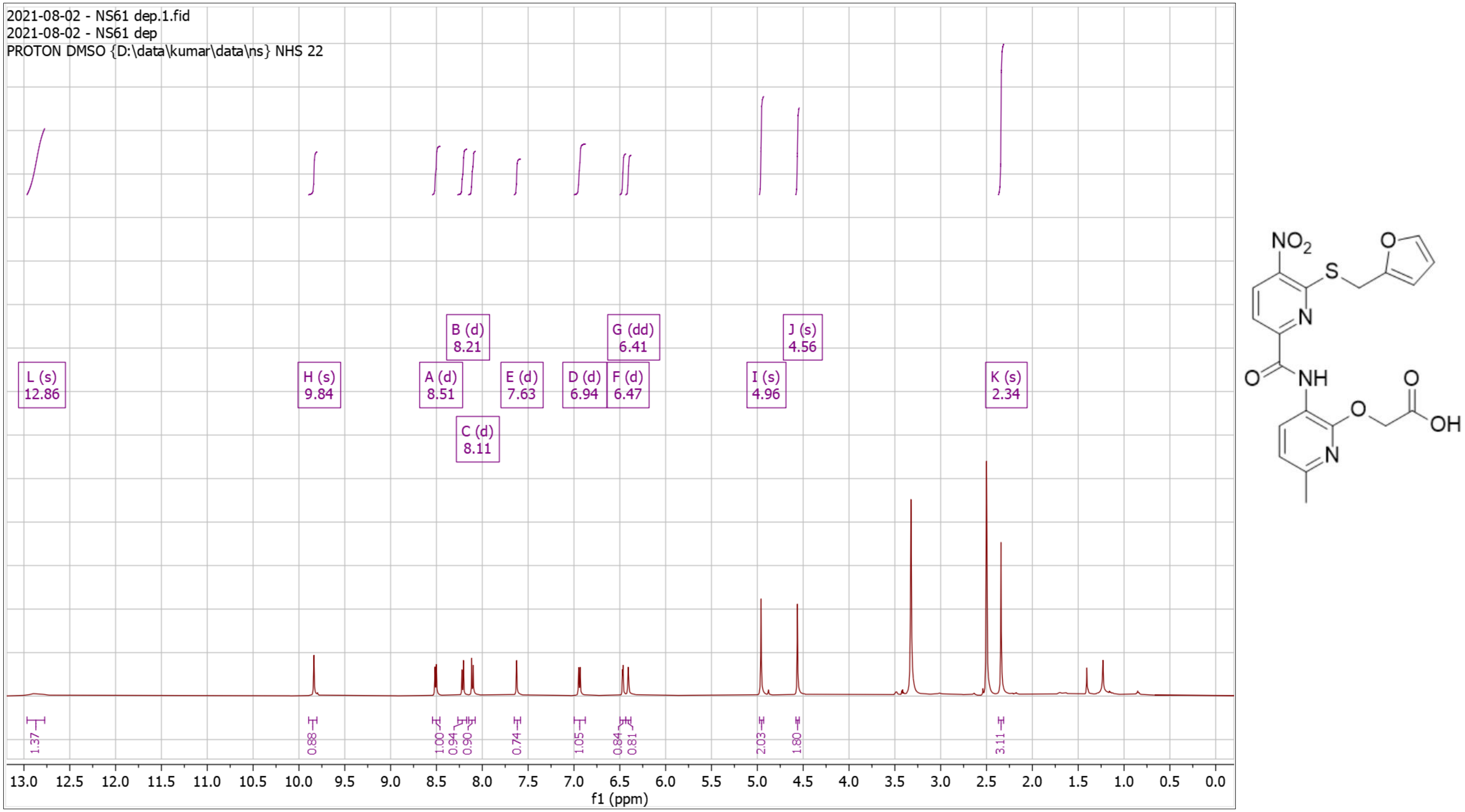

Supplementary Fig. 76. ^{1}H-NMR of NS62 Pro

^{1}H NMR (500 MHz, $CDCl_3$) δ 1.46 – 1.49 (s, 18H), 2.36 – 2.41 (s, 3H), 2.67 – 2.74 (t, *J* = 6.1 Hz, 2H), 4.00 – 4.07 (q, *J* = 6.0 Hz, 2H), 4.86 – 4.93 (s, 2H), 6.79 – 6.85 (d, *J* = 7.9 Hz, 1H), 7.55 – 7.61 (d, *J* = 8.4 Hz, 1H), 8.50 – 8.56 (t, *J* = 5.9 Hz, 1H), 8.57 – 8.64 (d, *J* = 8.4 Hz, 1H), 8.64 – 8.71 (d, *J* = 7.9 Hz, 1H), 10.21 – 10.32 (s, 1H); HRMS (m/z): [M]+ calcd. for $C_{25}H_{33}N_5O_8$, 532.2402; found, 532.2402.

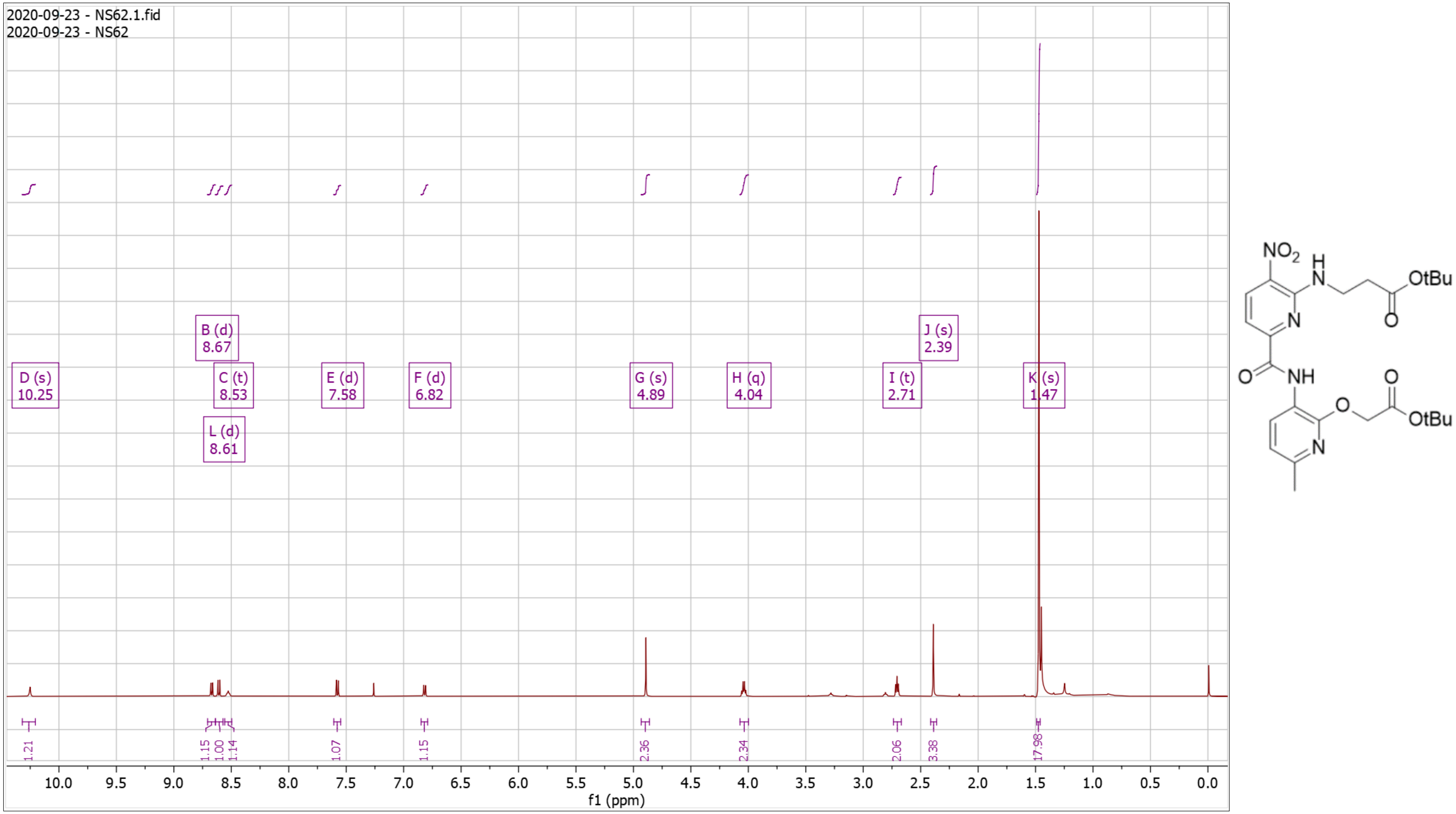

Supplementary Fig. 77. ^{1}H-NMR of NS62 Dep

^{1}H NMR (500 MHz, DMSO) δ 2.31 – 2.38 (s, 3H), 2.64 – 2.74 (t, *J* = 6.8 Hz, 2H), 3.83 – 3.94 (q, *J* = 6.5 Hz, 2H), 4.86 – 5.00 (s, 2H), 6.91 – 6.96 (d, *J* = 7.9 Hz, 1H), 7.41 – 7.48 (d, *J* = 8.7 Hz, 1H), 8.50 – 8.55 (d, *J* = 7.9 Hz, 1H), 8.59 – 8.72 (dd, 2H), 10.14 – 10.36 (s, 1H); HRMS (m/z): [M]+ calcd. for $C_{17}H_{17}N_5O_8$, 420.1150; found, 420.1143.

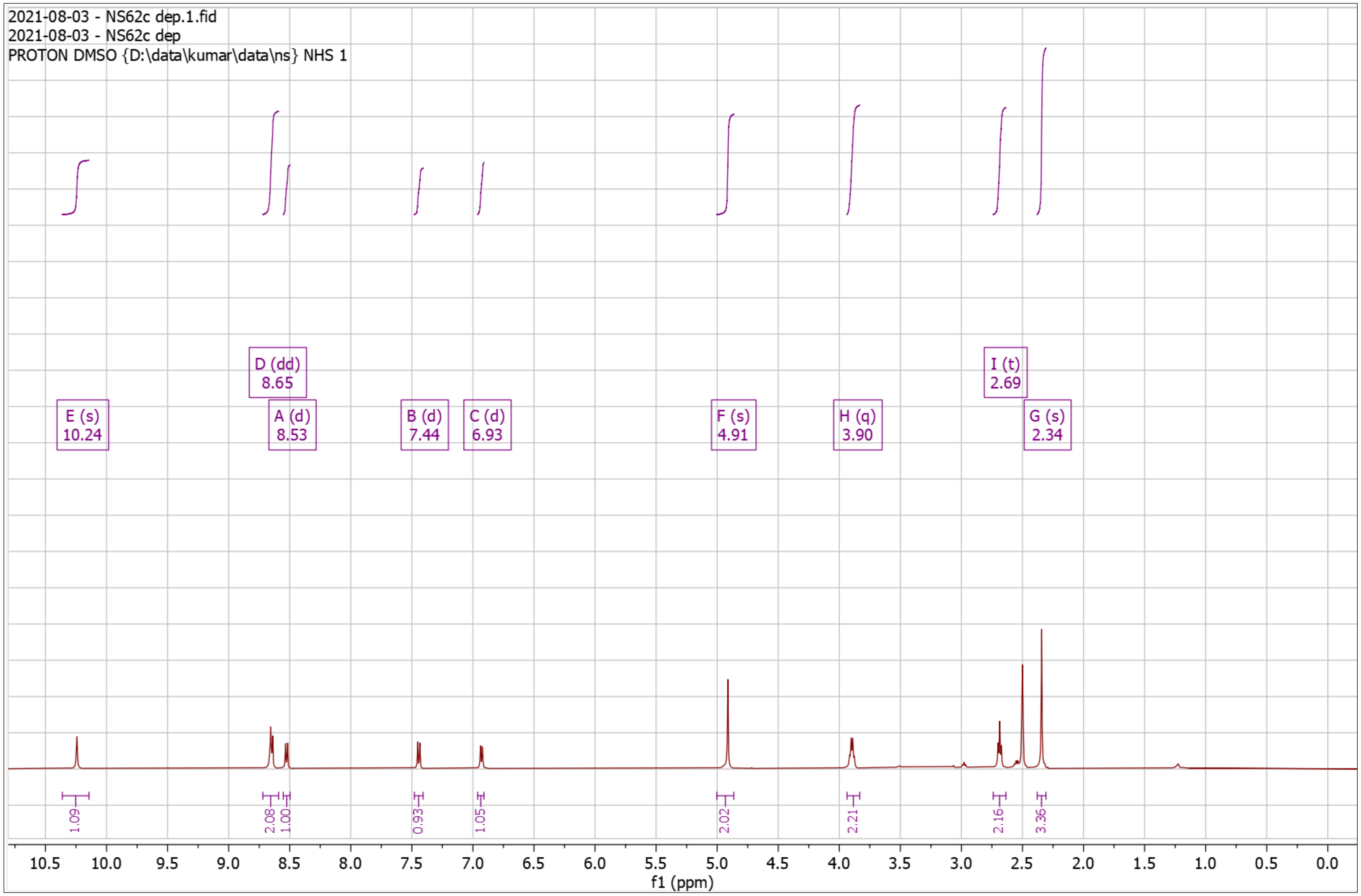

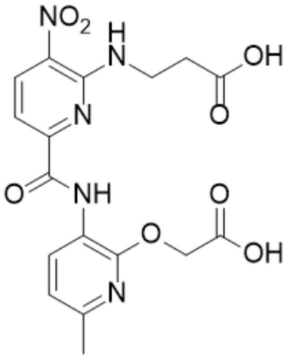

Supplementary Fig. 78. ^{1}H-NMR of NS71 Pro

^{1}H NMR (500 MHz, DMSO) δ 1.35 – 1.46 (s, 9H), 2.32 – 2.36 (s, 3H), 2.81 – 2.92 (t, *J* = 6.4 Hz, 2H), 3.88 – 3.97 (q, *J* = 6.0 Hz, 2H), 4.94 – 4.98 (s, 2H), 6.86 – 7.02 (d, *J* = 7.9 Hz, 1H), 7.37 – 7.50 (d, *J* = 8.3 Hz, 1H), 8.47 – 8.53 (d, *J* = 8.0 Hz, 1H), 8.61 – 8.71 (d, *J* = 8.3 Hz, 1H), 9.06 – 9.19 (t, *J* = 4.9 Hz, 1H), 10.19 – 10.30 (s, 1H); HRMS (m/z): [M]+ calcd. for $C_{20}H_{25}N_5O_9S$, 512.1446; found, 512.1439.

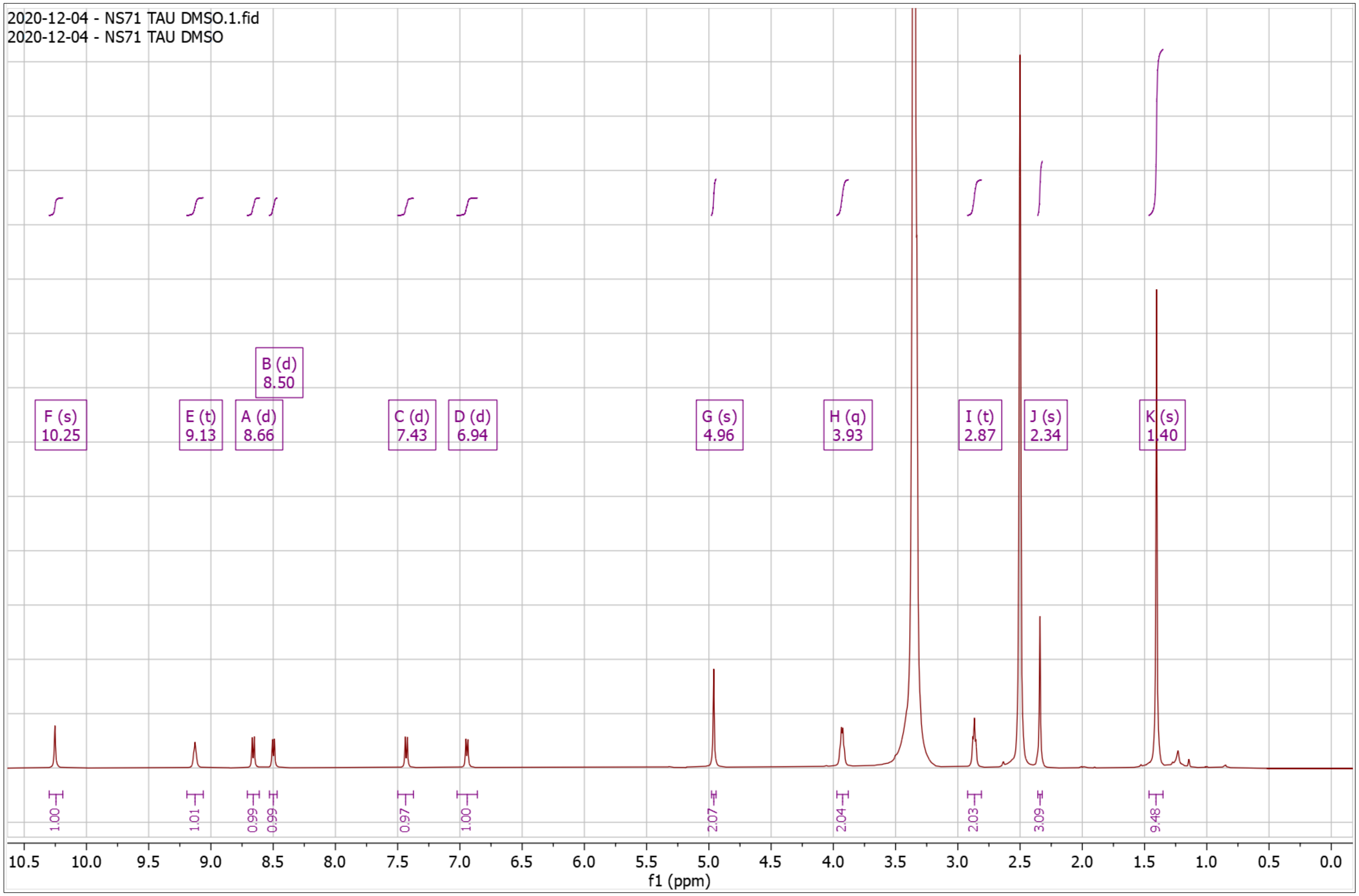

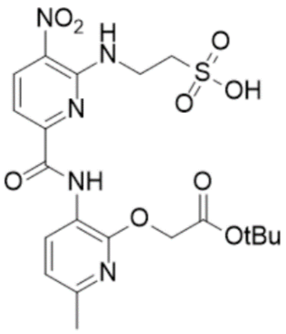

Supplementary Fig. 79. ^{1}H-NMR of NS71 Dep

^{1}H NMR (500 MHz, DMSO) δ 2.26 – 2.41 (s, 3H), 2.82 – 2.91 (t, J = 6.2 Hz, 2H), 3.90 – 4.02 (q, J = 5.8 Hz, 2H), 4.88 – 5.02 (s, 2H), 6.87 – 6.97 (d, J = 7.9 Hz, 1H), 7.37 – 7.48 (d, J = 8.4 Hz, 1H), 8.41 – 8.54 (d, J = 7.9 Hz, 1H), 8.59 – 8.70 (d, J = 8.4 Hz, 1H), 9.07 – 9.20 (t, J = 5.1 Hz, 1H), 10.23 – 10.42 (s, 1H); HRMS (m/z): [M]+ calcd. for $C_{16}H_{17}N_5O_9S$, 456.0820; found, 456.0820.

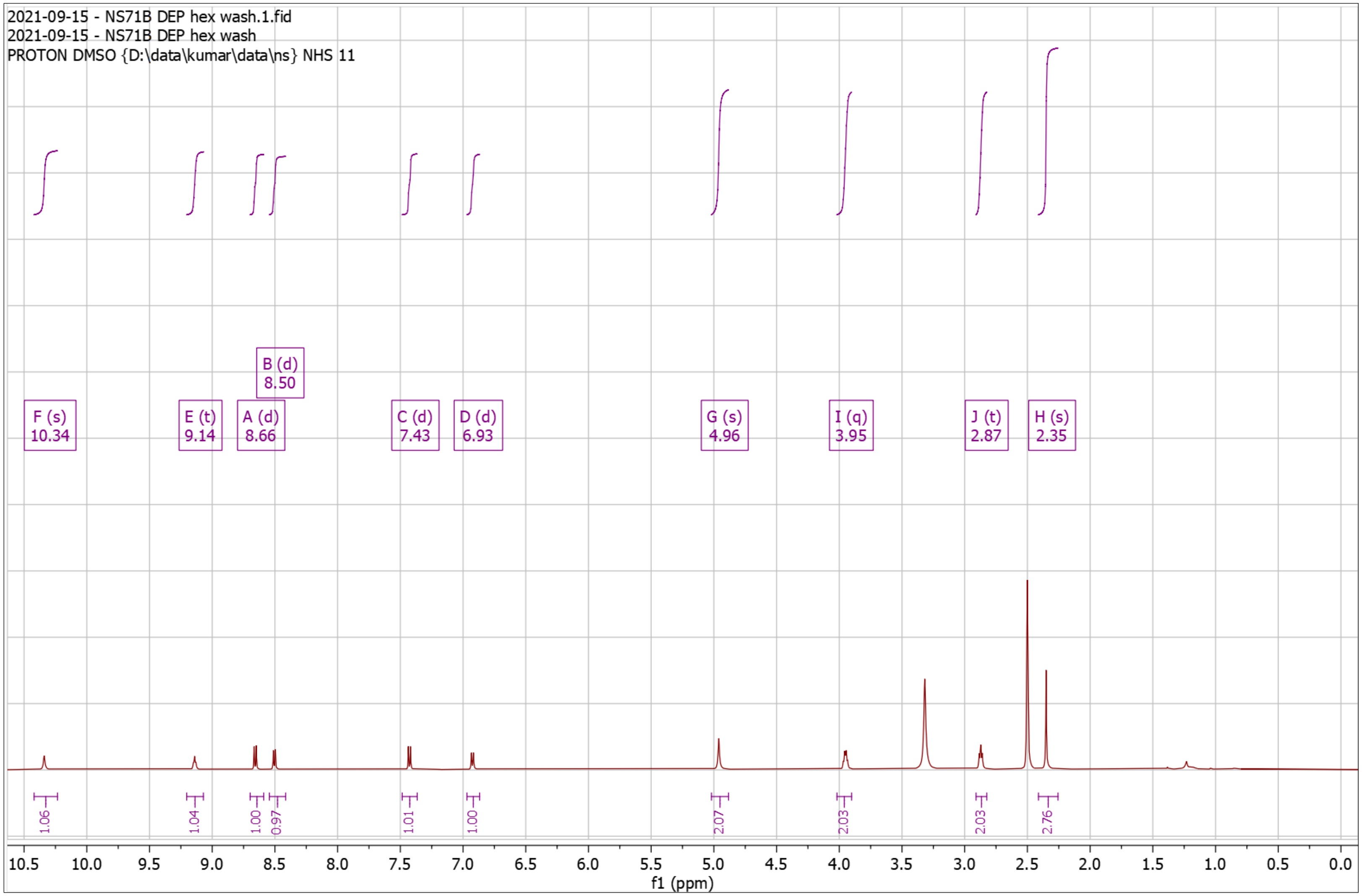

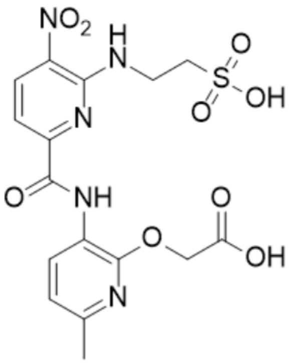

Supplementary Fig. 80. ^{1}H-NMR of NS72 Pro

^{1}H NMR (500 MHz, $CDCl_3$) δ 1.45 – 1.50 (s, 9H), 2.36 – 2.43 (s, 3H), 3.18 – 3.29 (t, J = 6.8 Hz, 2H), 4.07 – 4.17 (q, J = 6.5 Hz, 2H), 4.63 – 4.67 (s, 2H), 6.80 – 6.86 (d, J = 7.9 Hz, 1H), 7.04 – 7.10 (t, J = 7.5 Hz, 1H), 7.14 – 7.22 (m, 2H), 7.34 – 7.39 (d, J = 8.2 Hz, 1H), 7.49 – 7.55 (d, J = 8.4 Hz, 1H), 7.61 – 7.66 (d, J = 8.0 Hz, 1H), 8.05 – 8.08 (s, 1H), 8.29 – 8.33 (t, 1H), 8.53 – 8.58 (d, J = 8.4 Hz, 1H), 8.65 – 8.70 (d, J = 7.9 Hz, 1H), 10.17 – 10.28 (s, 1H); HRMS (m/z): [M]+ calcd. for $C_{28}H_{30}N_6O_6$, 547.2300; found, 547.2299.

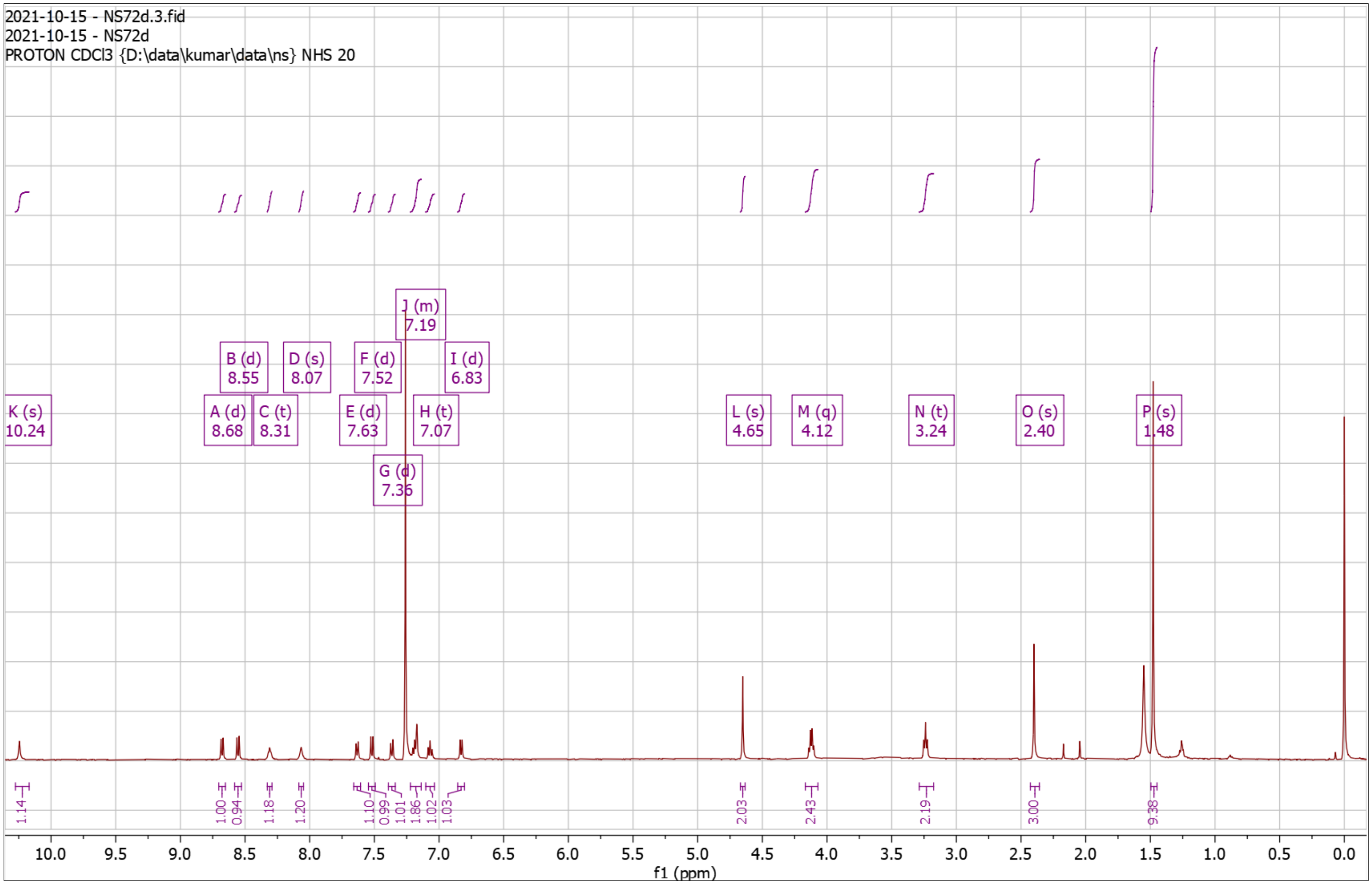

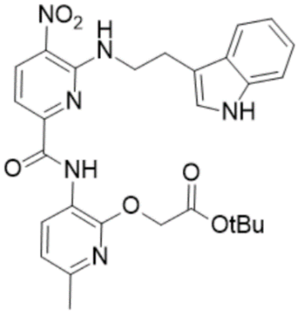

Supplementary Fig. 81. ^{1}H-NMR of NS72 Dep

^{1}H NMR (500 MHz, DMSO) δ 2.32 – 2.35 (s, 3H), 3.05 – 3.12 (t, *J* = 6.8 Hz, 2H), 3.93 – 4.04 (q, *J* = 6.5 Hz, 2H), 4.61 – 4.65 (s, 2H), 6.81 – 6.86 (t, *J* = 7.4 Hz, 1H), 6.86 – 6.92 (d, *J* = 7.9 Hz, 1H), 6.94 – 7.02 (t, *J* = 7.5 Hz, 1H), 7.22 – 7.25 (d, *J* = 2.3 Hz, 1H), 7.24 – 7.30 (d, *J* = 8.1 Hz, 1H), 7.31 – 7.38 (d, *J* = 8.4 Hz, 1H), 7.50 – 7.56 (d, *J* = 7.9 Hz, 1H), 8.48 – 8.53 (d, *J* = 7.8 Hz, 1H), 8.53 – 8.65 (dd, *J* = 9.6, 6.9 Hz, 2H), 10.33 – 10.41 (s, 1H), 10.79 – 10.87 (m, 1H). HRMS (m/z): [M]+ calcd. for $C_{24}H_{22}N_6O_6$, 491.1674; found, 491.1678.

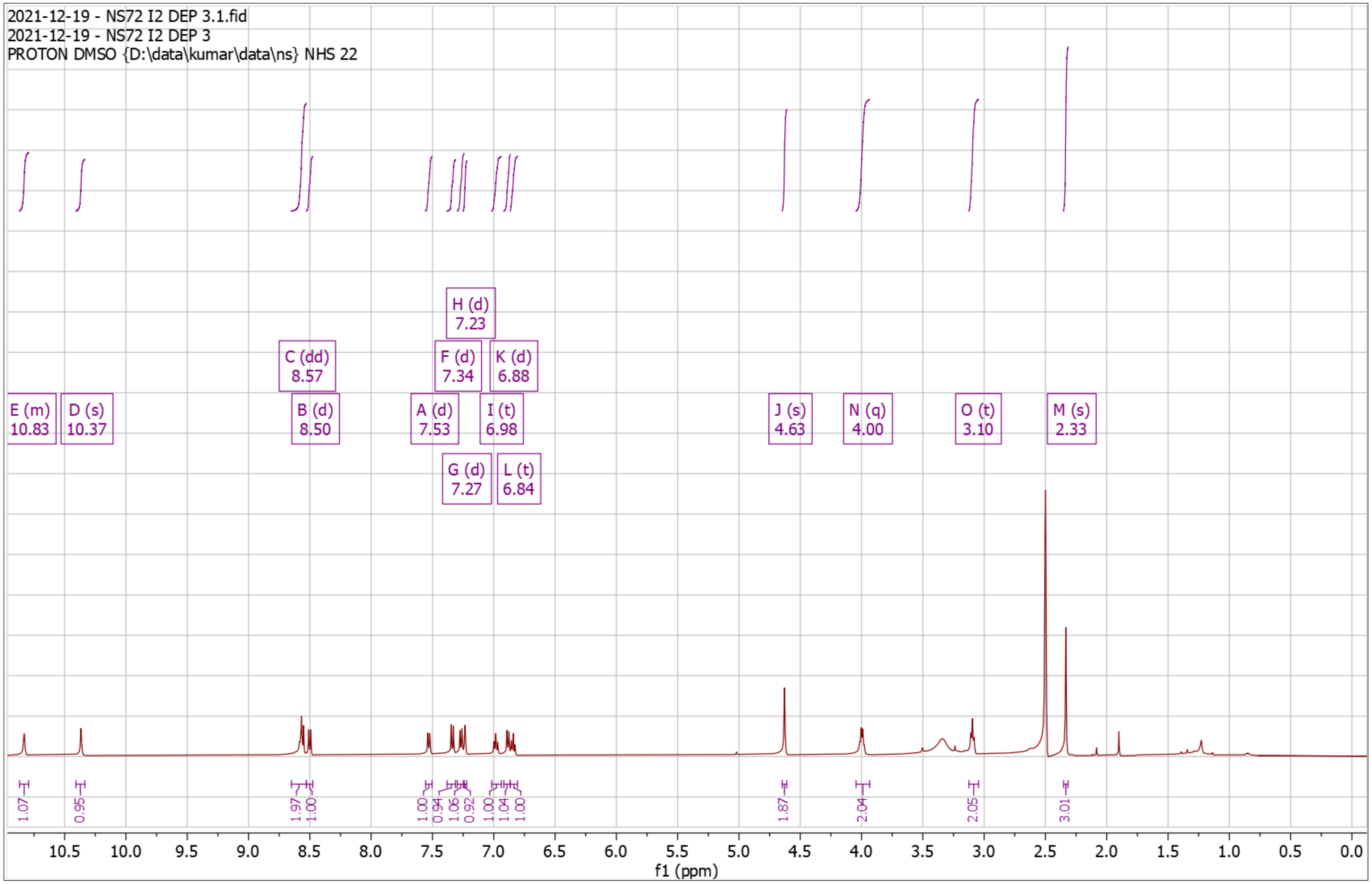

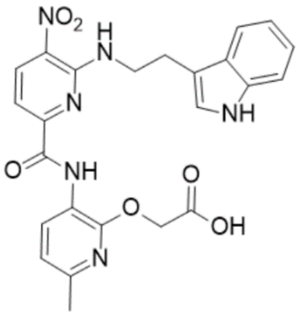

Supplementary Fig. 82. ^{1}H-NMR of NS119 Pro

^{1}H NMR (500 MHz, $CDCl_3$) δ 1.39 – 1.41 (s, 9H), 1.48 – 1.51 (s, 9H), 2.36 – 2.44 (s, 3H), 3.47 – 3.60 (q, *J* = 6.0 Hz, 2H), 3.84 – 3.99 (q, *J* = 5.9 Hz, 2H), 4.84 – 4.95 (s, 2H), 4.99 – 5.09 (s, 1H), 6.80 – 6.86 (d, *J* = 7.9 Hz, 1H), 7.56 – 7.62 (d, *J* = 8.4 Hz, 1H), 8.35 – 8.47 (s, 1H), 8.59 – 8.64 (d, *J* = 8.4 Hz, 1H), 8.65 – 8.70 (d, *J* = 7.9 Hz, 1H), 10.13 – 10.25 (s, 1H); HRMS (m/z): [M]+ calcd. for $C_{25}H_{34}N_6O_8$, 547.2511; found, 547.2510.

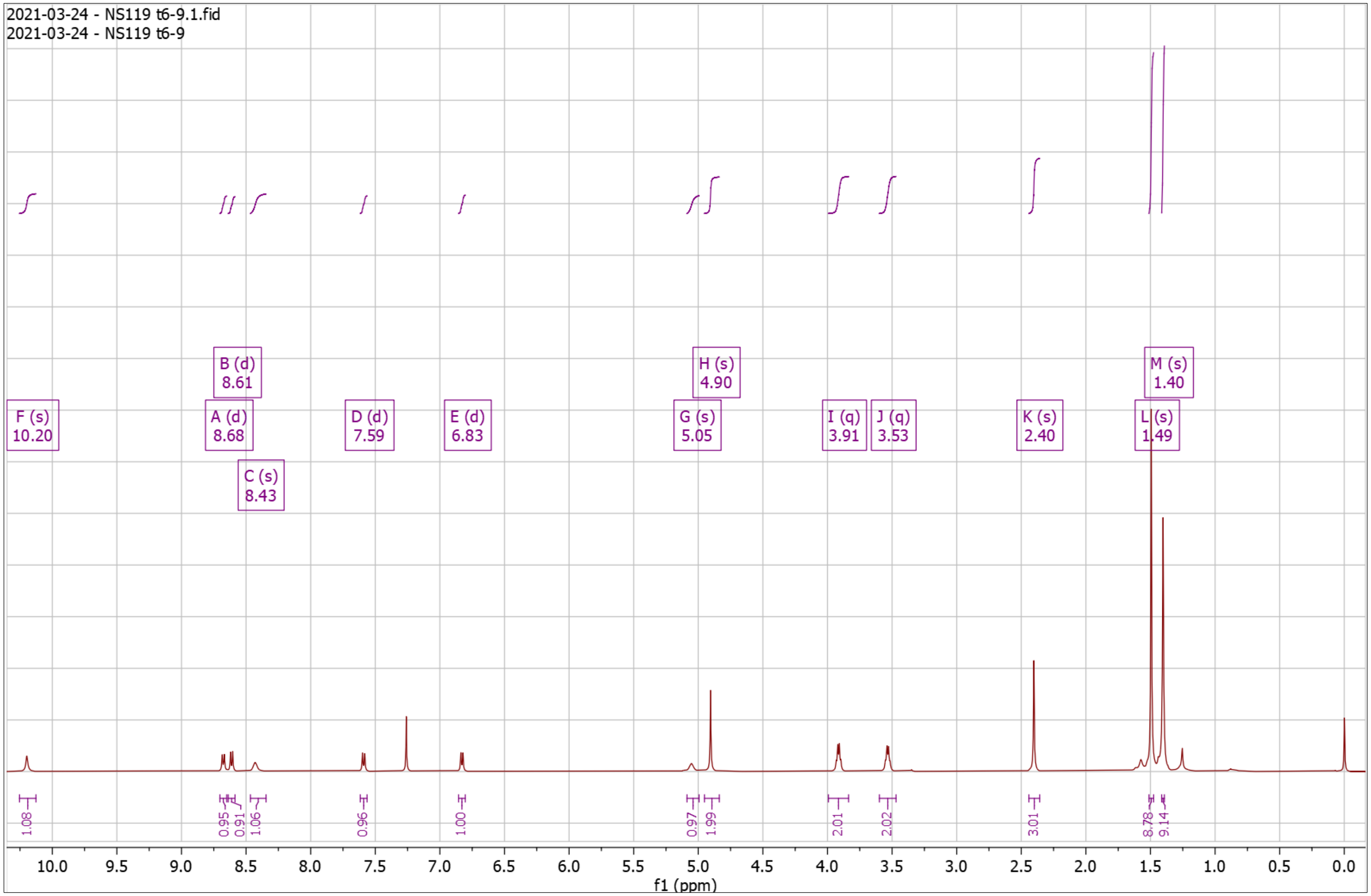

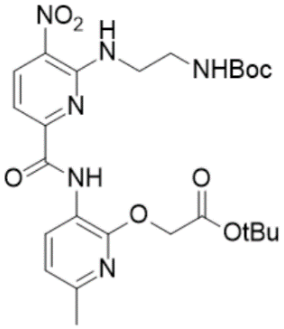

Supplementary Fig. 83. ¹H-NMR of NS119 Dep

¹H NMR (500 MHz, DMSO) δ 2.32 – 2.38 (s, 3H), 3.13 – 3.21 (t, *J* = 5.5 Hz, 2H), 3.89 – 3.98 (q, *J* = 5.6 Hz, 2H), 4.96 – 4.99 (s, 2H), 6.94 – 6.99 (d, *J* = 7.9 Hz, 1H), 7.48 – 7.53 (d, *J* = 8.4 Hz, 1H), 7.72 – 7.89 (s, 4H), 8.44 – 8.49 (d, *J* = 7.8 Hz, 1H), 8.58 – 8.66 (t, *J* = 5.9 Hz, 1H), 8.66 – 8.74 (d, *J* = 8.4 Hz, 1H), 10.07 – 10.21 (s, 1H); HRMS (m/z): [M]+ calcd. for $C_{16}H_{18}N_6O_6$, 391.1361; found, 391.1365.

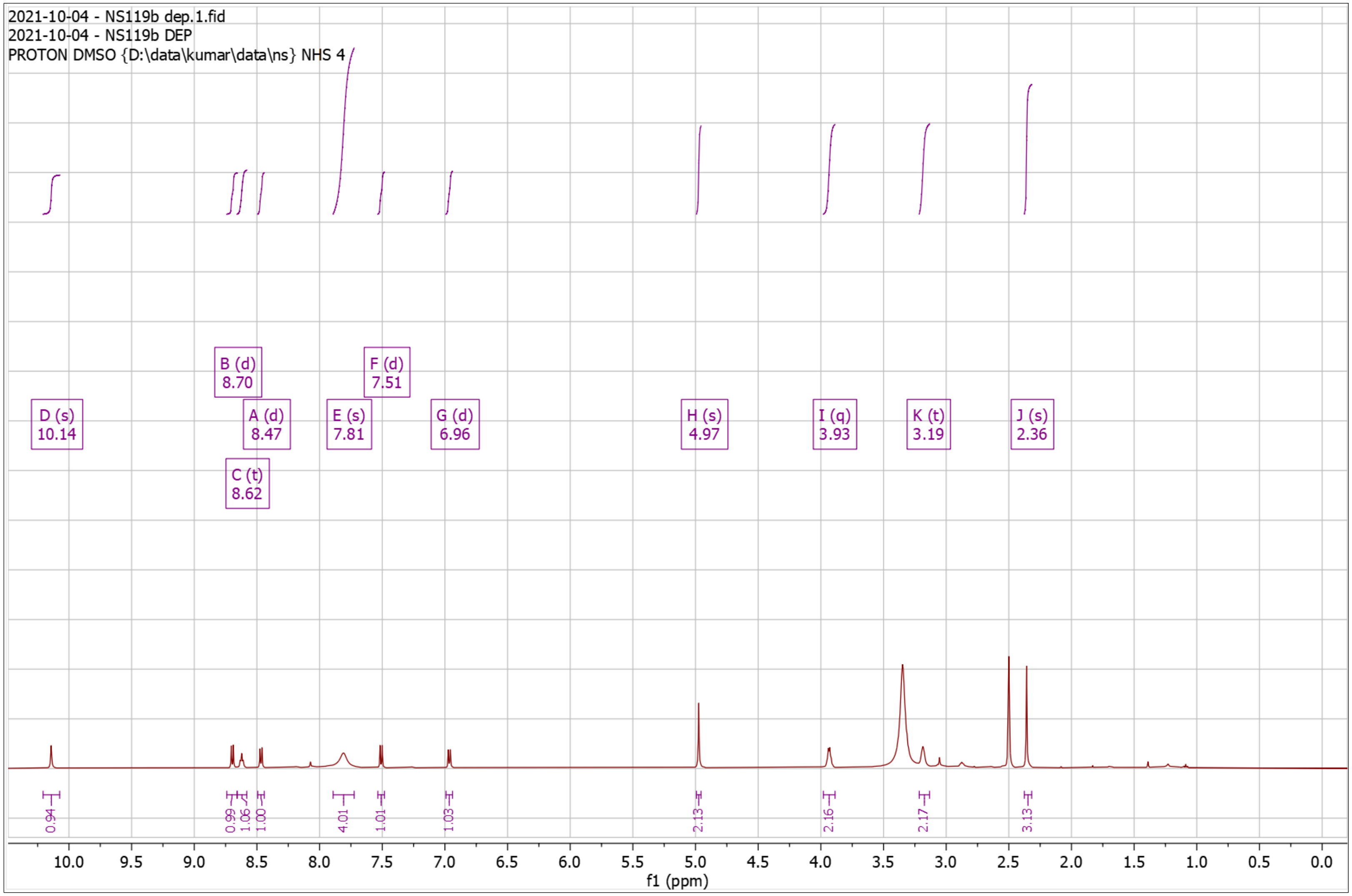

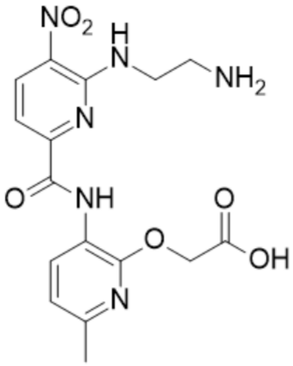

Supplementary Fig. 84. ^{1}H-NMR of NS137 Pro

^{1}H NMR (500 MHz, $CDCl_3$) δ 1.02 – 1.10 (d, *J* = 6.7 Hz, 6H), 1.41 – 1.49 (s, 9H), 1.97 – 2.14 (dp, *J* = 13.6, 6.9 Hz, 1H), 2.35 – 2.41 (s, 3H), 3.52 – 3.67 (t, *J* = 6.4 Hz, 2H), 4.79 – 4.94 (s, 2H), 6.72 – 6.88 (d, *J* = 7.8 Hz, 1H), 7.48 – 7.57 (d, *J* = 8.4 Hz, 1H), 8.25 – 8.42 (t, *J* = 6.0 Hz, 1H), 8.53 – 8.62 (d, *J* = 8.4 Hz, 1H), 8.62 – 8.69 (d, *J* = 7.9 Hz, 1H), 10.09 – 10.31 (s, 1H); HRMS (m/z): [M]+ calcd. for $C_{22}H_{29}N_5O_6$, 460.2191; found, 460.2188.

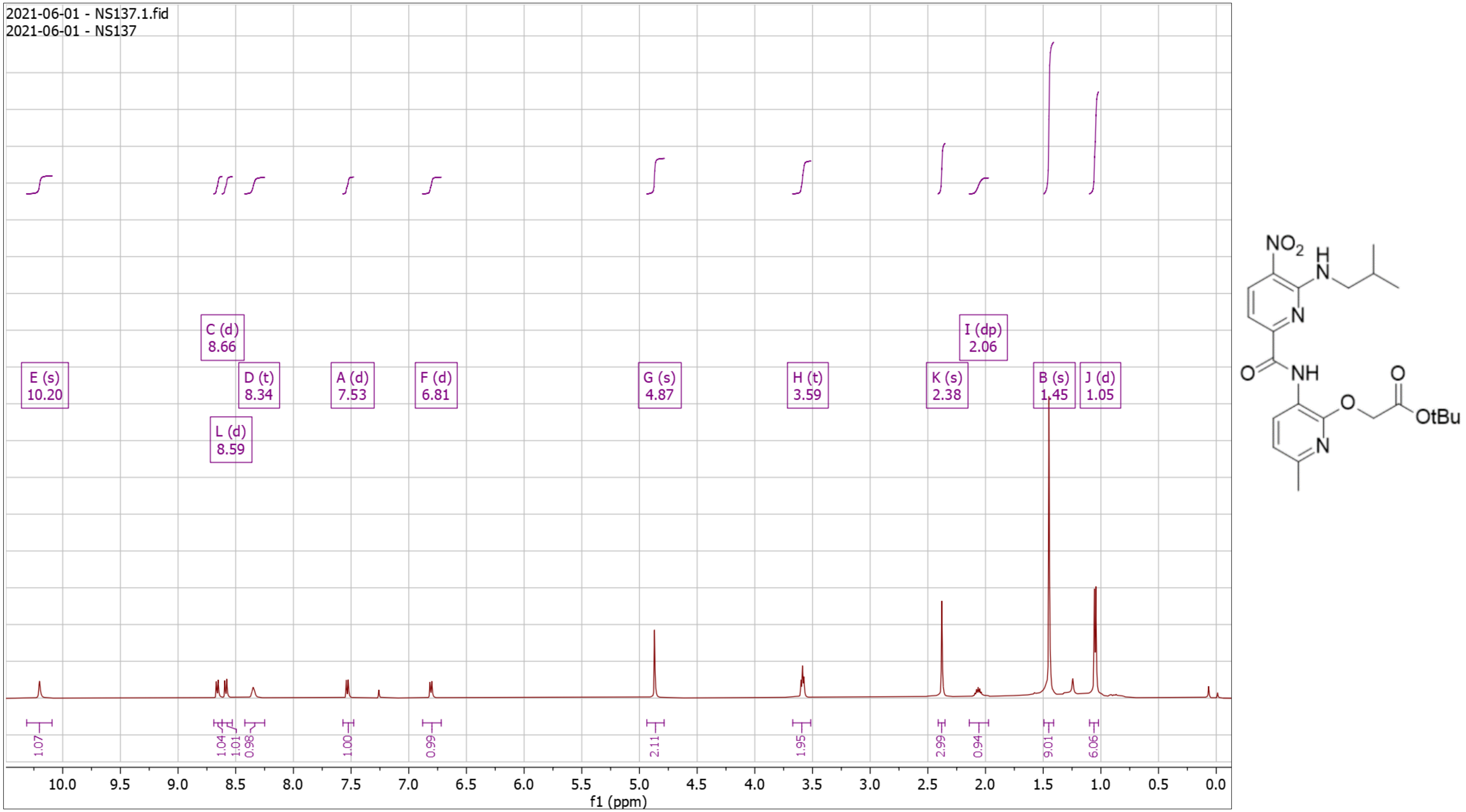

Supplementary Fig. 85. ^{1}H-NMR of NS137 Dep

^{1}H NMR (500 MHz, DMSO) δ 0.94 – 0.96 (d, J = 6.7 Hz, 6H), 1.99 – 2.10 (tt, J = 13.3, 6.4 Hz, 1H), 2.33 – 2.37 (s, 3H), 3.48 – 3.54 (t, J = 6.4 Hz, 2H), 4.96 – 4.99 (s, 2H), 6.93 – 6.99 (d, J = 7.9 Hz, 1H), 7.40 – 7.45 (d, J = 8.3 Hz, 1H), 8.51 – 8.58 (d, J = 8.0 Hz, 1H), 8.58 – 8.63 (t, J = 6.0 Hz, 1H), 8.62 – 8.70 (d, J = 8.4 Hz, 1H), 10.10 – 10.17 (s, 1H); HRMS (m/z): [M]+ calcd. for $C_{18}H_{21}N_5O_6$, 404.1565; found, 404.1565.

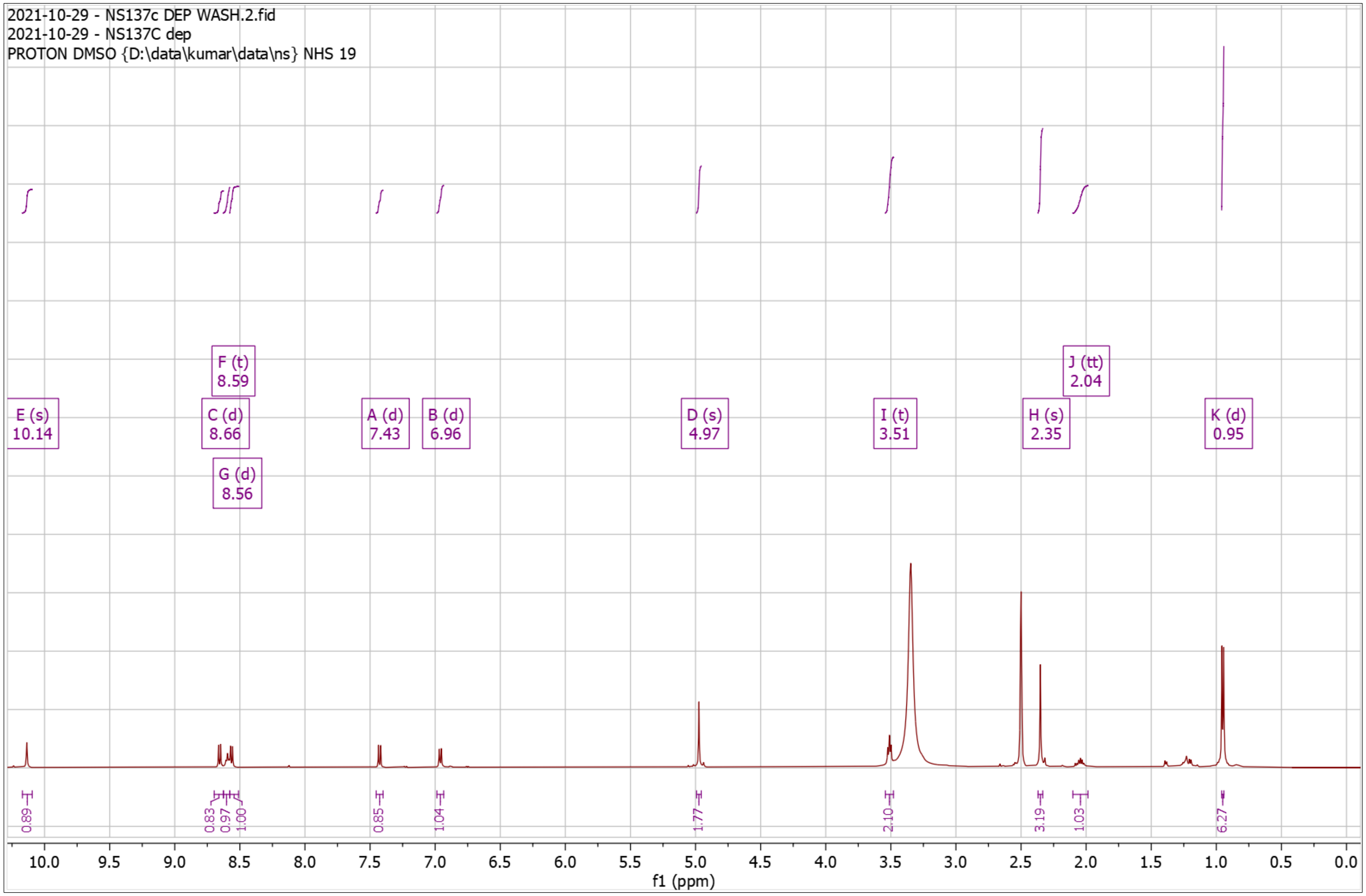

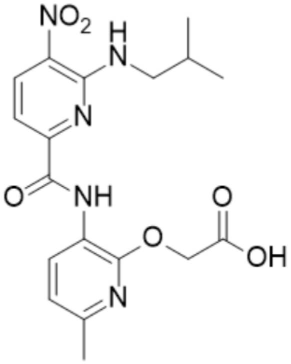

Supplementary Fig. 86. ^{1}H-NMR of NS157 Pro

^{1}H NMR (500 MHz, $CDCl_3$) δ 0.80 – 0.95 (m, 5H), 1.30 – 1.39 (m, 4H), 1.46 – 1.47 (s, 9H), 1.71 – 1.82 (p, *J* = 7.1 Hz, 2H), 2.36 – 2.41 (s, 3H), 3.71 – 3.81 (q, *J* = 6.5 Hz, 2H), 4.84 – 4.88 (s, 2H), 6.80 – 6.85 (d, *J* = 7.9 Hz, 1H), 7.53 – 7.58 (d, *J* = 8.3 Hz, 1H), 8.22 – 8.28 (t, *J* = 5.8 Hz, 1H), 8.58 – 8.63 (d, 1H), 8.65 – 8.71 (d, *J* = 7.9 Hz, 1H), 10.25 – 10.29 (s, 1H); HRMS (m/z): [M]+ calcd. for $C_{24}H_{33}N_5O_6$, 488.2504; found, 488.2503.

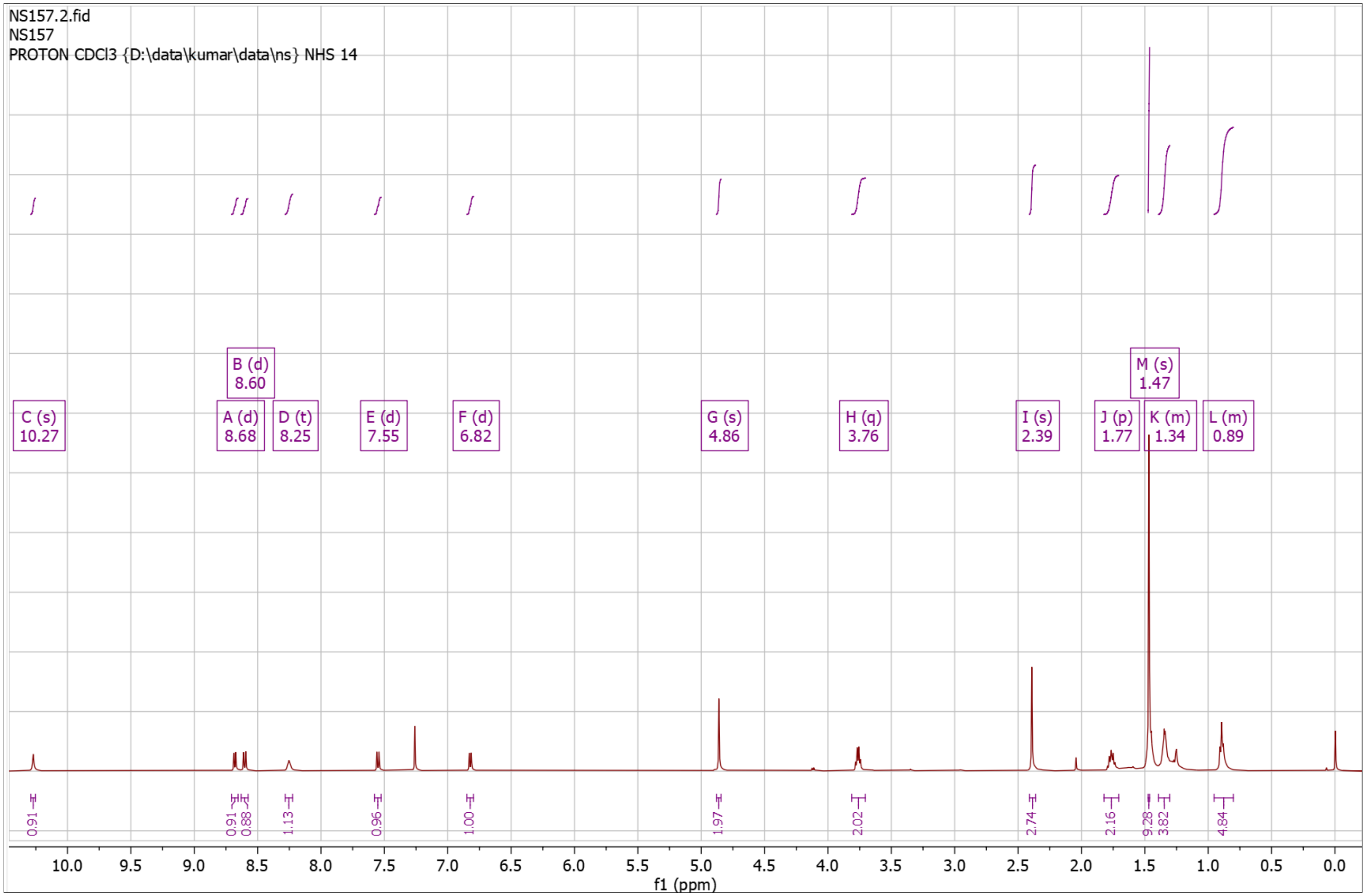

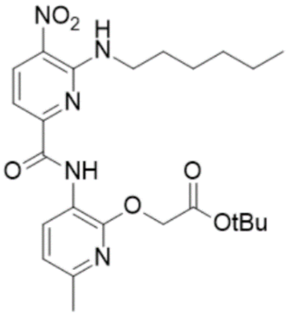

Supplementary Fig. 87. ^{1}H-NMR of NS157 Dep

^{1}H NMR (500 MHz, DMSO) δ 0.74 – 0.89 (m, 3H), 1.24 – 1.33 (m, 4H), 1.32 – 1.42 (p, J = 6.9 Hz, 2H), 1.60 – 1.72 (p, J = 7.2 Hz, 2H), 2.29 – 2.34 (s, 3H), 3.61 – 3.76 (q, J = 6.7 Hz, 2H), 4.64 – 4.77 (s, 2H), 6.80 – 6.91 (d, J = 7.9 Hz, 1H), 7.39 – 7.44 (d, J = 8.4 Hz, 1H), 8.45 – 8.53 (d, J = 7.9 Hz, 1H), 8.53 – 8.59 (t, J = 5.8 Hz, 1H), 8.59 – 8.67 (d, J = 8.4 Hz, 1H), 10.35 – 10.50 (s, 1H); HRMS (m/z): [M]+ calcd. for $C_{20}H_{25}N_5O_6$, 432.1878; found, 432.1882.

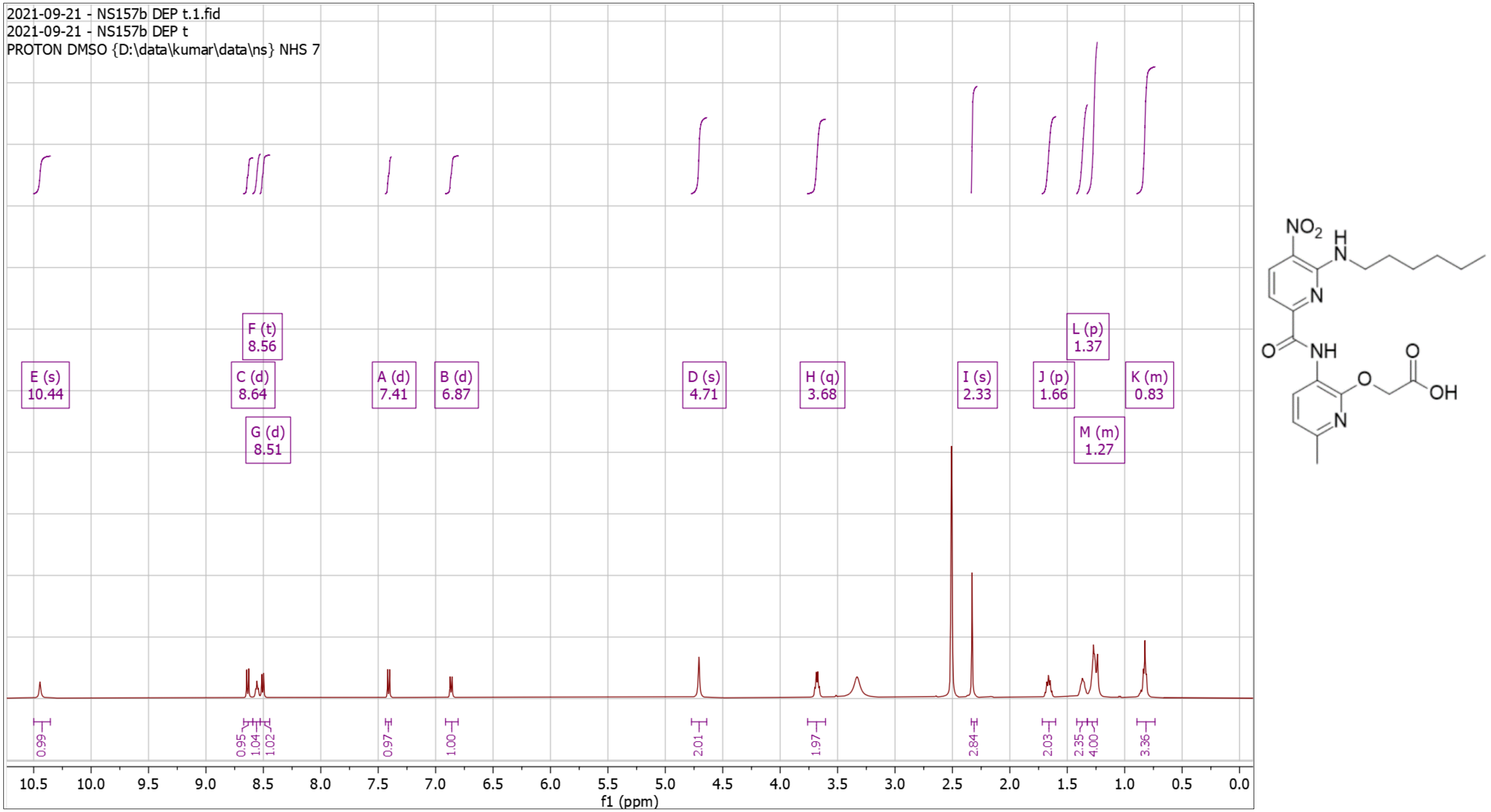

Supplementary Fig. 88. ^{1}H-NMR of NS158 Pro

^{1}H NMR (500 MHz, $CDCl_3$) δ 1.49 – 1.51 (s, 9H), 2.38 – 2.41 (s, 3H), 3.93 – 4.02 (d, *J* = 3.3 Hz, 4H), 4.88 – 4.92 (s, 2H), 6.79 – 6.84 (d, *J* = 7.9 Hz, 1H), 7.54 – 7.59 (d, *J* = 8.4 Hz, 1H), 8.41 – 8.45 (t, 1H), 8.57 – 8.62 (d, *J* = 8.4 Hz, 1H), 8.63 – 8.68 (d, *J* = 7.9 Hz, 1H), 10.19 – 10.23 (s, 1H); HRMS (m/z): [M]+ calcd. for $C_{20}H_{25}N_5O_7$, 448.1827; found, 448.1827.

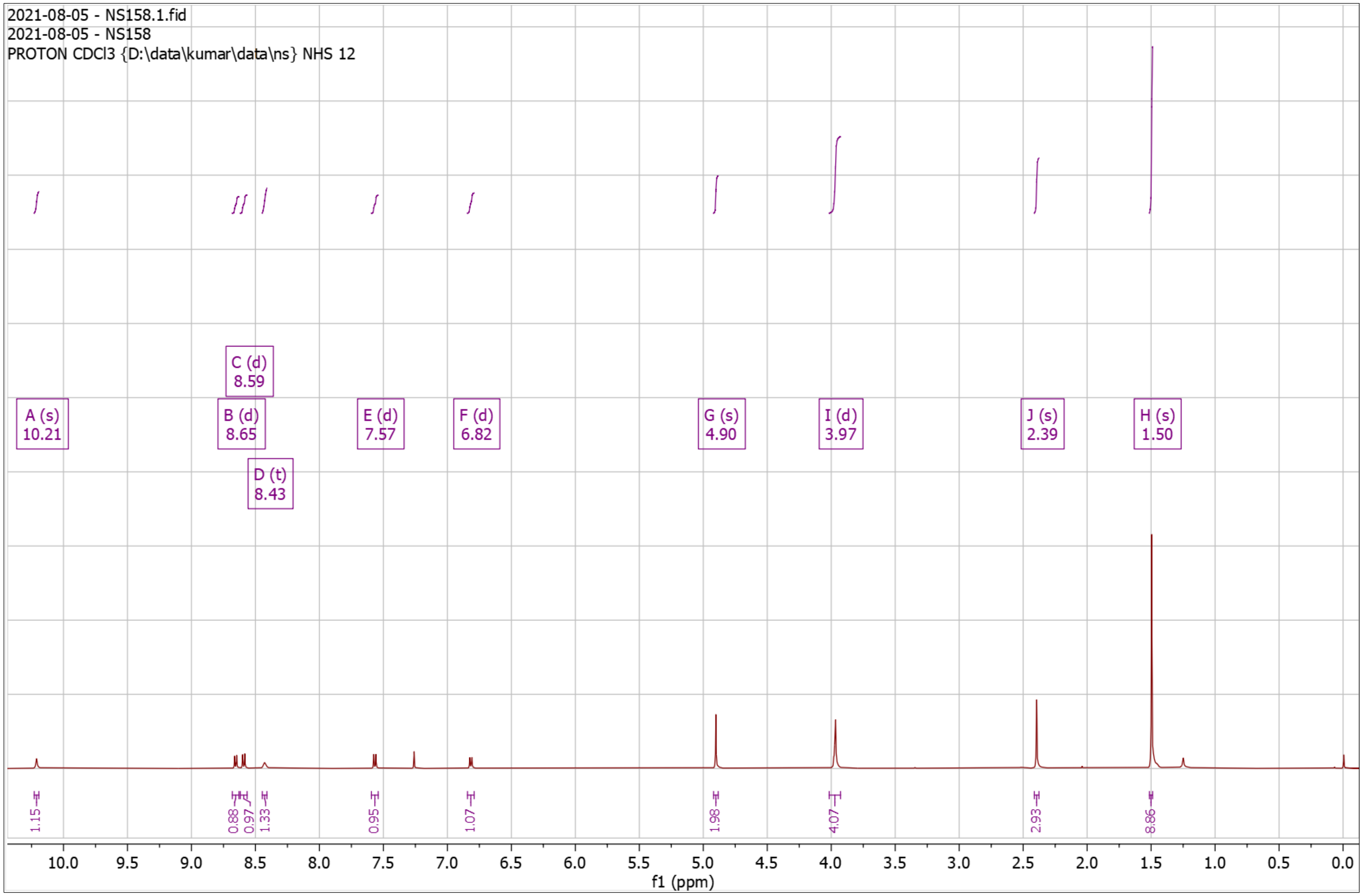

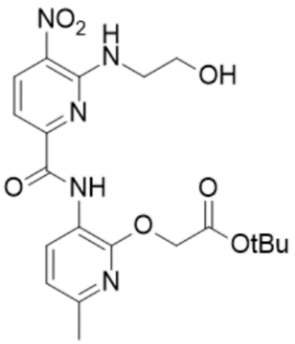

Supplementary Fig. 89. ^{1}H-NMR of NS158 Dep

^{1}H NMR (500 MHz, DMSO) δ 2.34 – 2.36 (s, 3H), 3.68 – 3.72 (t, *J* = 5.7 Hz, 2H), 3.72 – 3.78 (t, *J* = 5.5 Hz, 2H), 4.93 – 4.98 (s, 2H), 6.92 – 6.97 (d, *J* = 7.9 Hz, 1H), 7.38 – 7.49 (d, *J* = 8.4 Hz, 1H), 8.47 – 8.53 (d, *J* = 7.9 Hz, 1H), 8.53 – 8.59 (t, *J* = 5.6 Hz, 1H), 8.63 – 8.69 (d, *J* = 8.3 Hz, 1H), 10.31 – 10.33 (s, 1H); HRMS (m/z): [M]+ calcd. for $C_{16}H_{17}N_5O_7$, 392.1201; found, 392.1205.

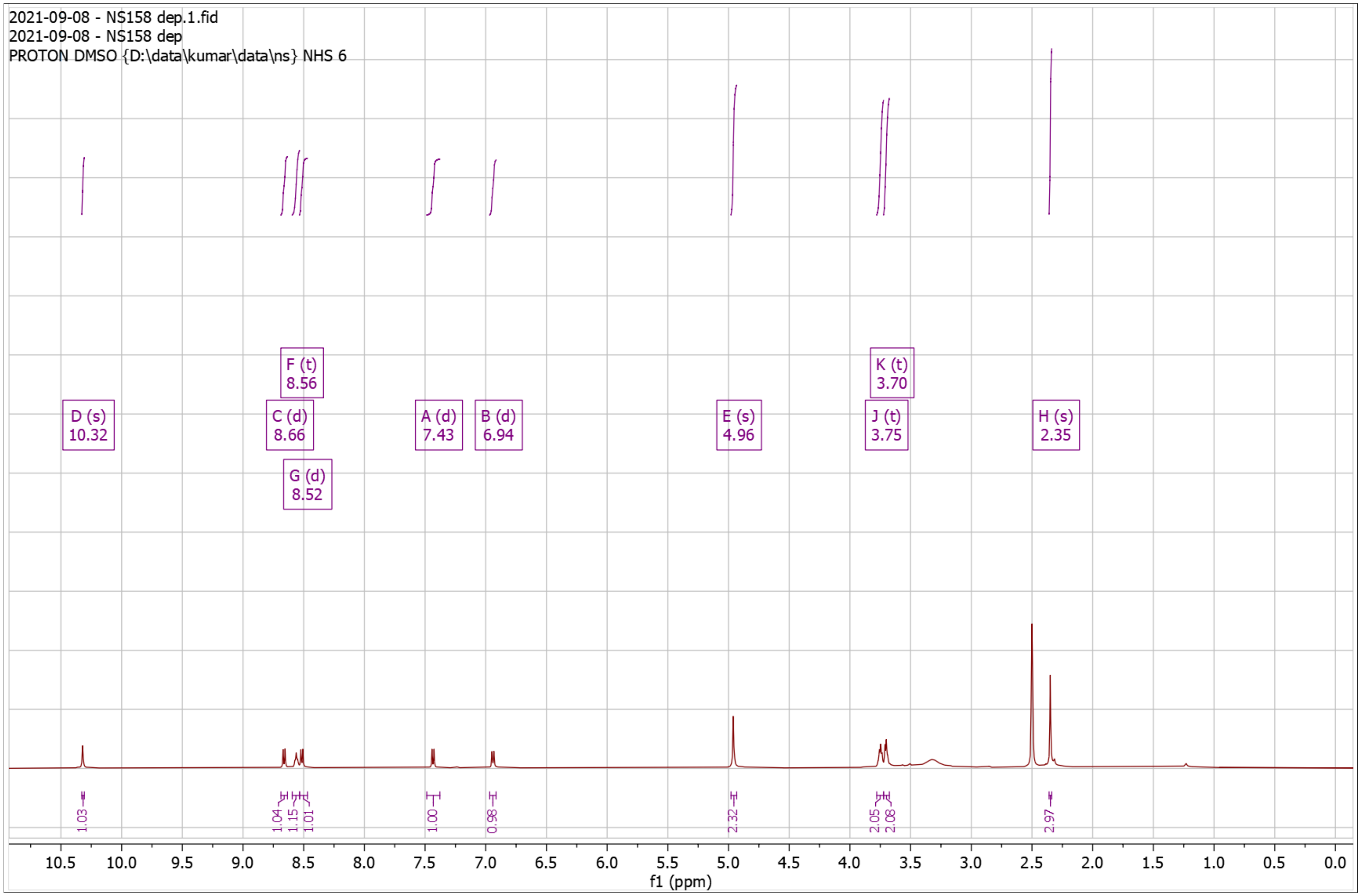

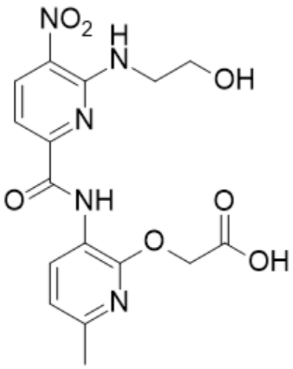

Supplementary Fig. 90. ^{1}H-NMR of NS160 Pro

^{1}H NMR (500 MHz, $CDCl_3$) δ 1.43 – 1.48 (s, 9H), 2.38 – 2.41 (s, 3H), 2.96 – 3.03 (t, J = 7.1 Hz, 2H), 3.97 – 4.04 (td, J = 7.1, 5.4 Hz, 2H), 4.67 – 4.71 (s, 2H), 4.80 – 4.94 (s, 1H), 6.74 – 6.80 (d, 2H), 6.80 – 6.85 (d, J = 8.0 Hz, 1H), 7.11 – 7.17 (d, 2H), 7.52 – 7.58 (d, J = 8.5 Hz, 1H), 8.18 – 8.29 (t, 1H), 8.52 – 8.63 (d, J = 8.5 Hz, 1H), 8.65 – 8.70 (d, J = 7.8 Hz, 1H), 10.24 – 10.28 (s, 1H); HRMS (m/z): [M]+ calcd. for $C_{26}H_{29}N_5O_7$, 524.2140; found, 524.2120.

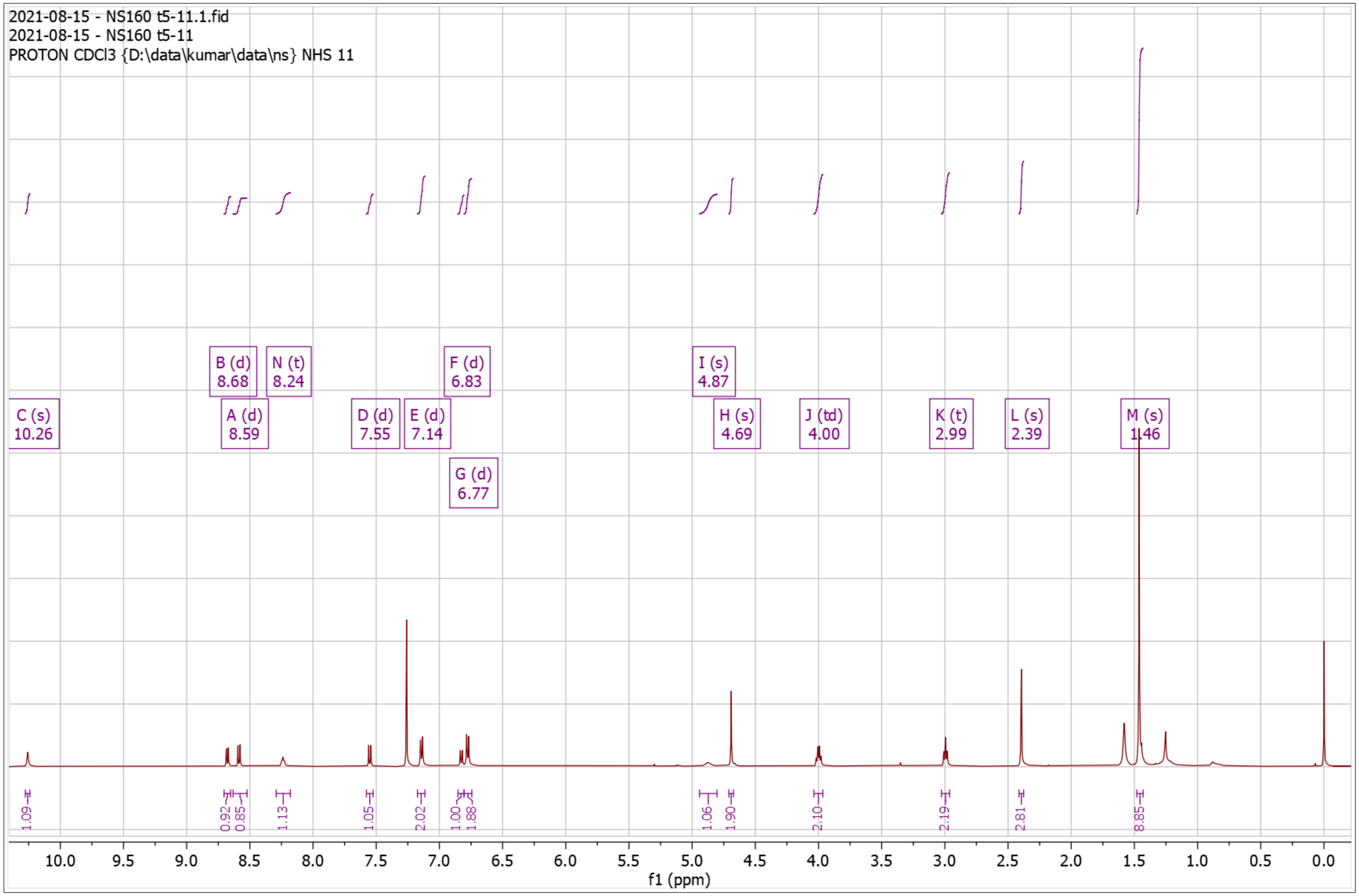

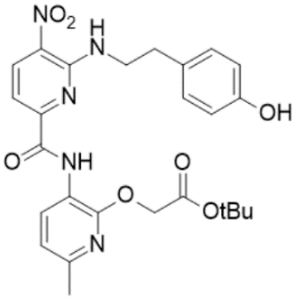

Supplementary Fig. 91. ^{1}H-NMR of NS160 Dep

^{1}H NMR (500 MHz, DMSO) δ 2.32 – 2.38 (s, 3H), 2.83 – 2.92 (t, *J* = 7.0 Hz, 2H), 3.83 – 3.95 (q, *J* = 6.7 Hz, 2H), 4.72 – 4.81 (s, 2H), 6.60 – 6.65 (d, *J* = 8.1 Hz, 2H), 6.93 – 6.99 (d, *J* = 7.9 Hz, 1H), 7.02 – 7.08 (d, *J* = 8.0 Hz, 2H), 7.35 – 7.46 (d, *J* = 8.4 Hz, 1H), 8.47 – 8.55 (t, *J* = 5.6 Hz, 1H), 8.55 – 8.59 (d, *J* = 7.9 Hz, 1H), 8.61 – 8.67 (d, *J* = 8.4 Hz, 1H), 9.08 – 9.21 (m, 1H), 10.15 – 10.27 (s, 1H); HRMS (m/z): [M]+ calcd. for $C_{22}H_{21}N_5O_7$, 468.1514; found, 468.1512.

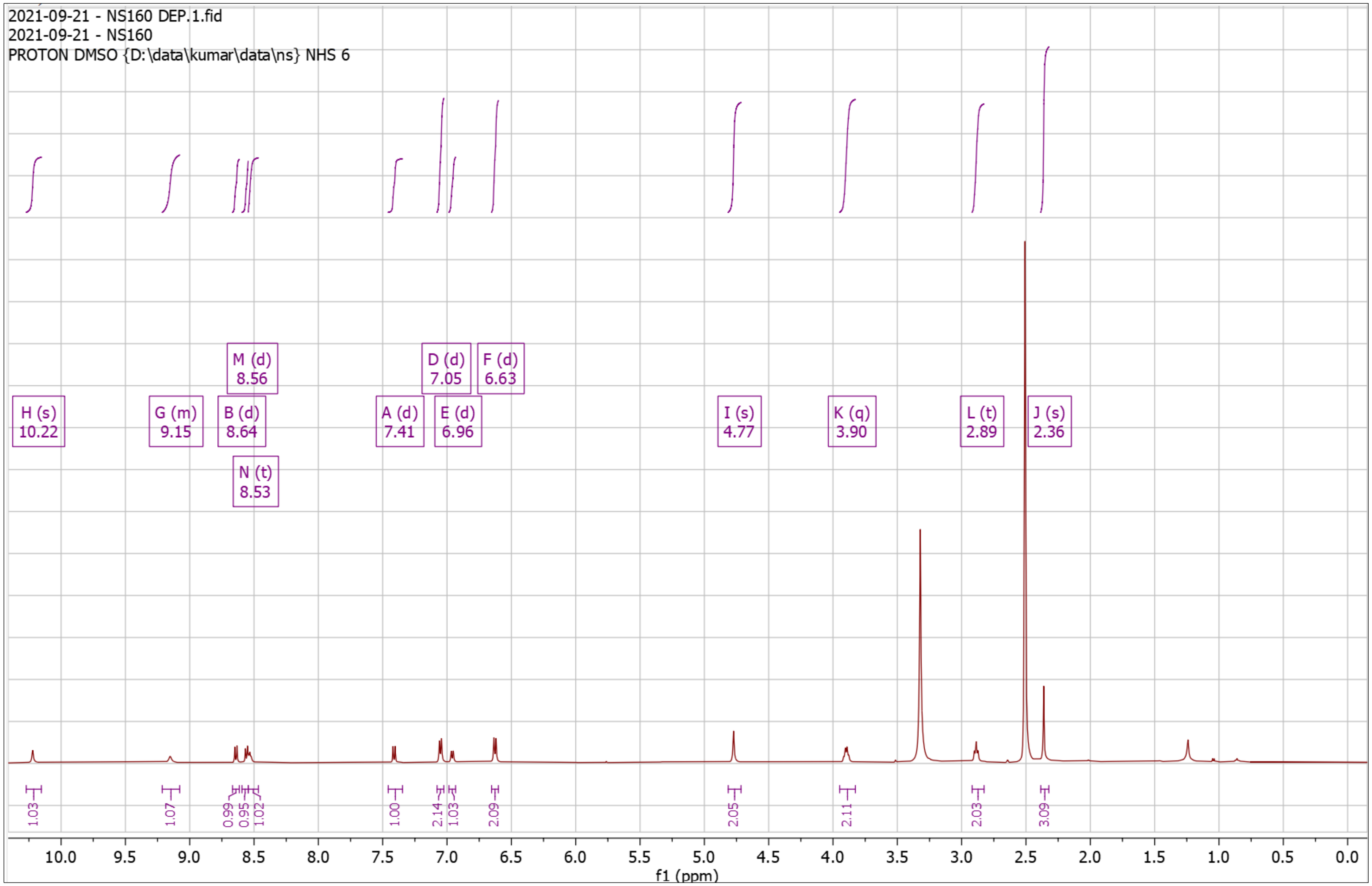

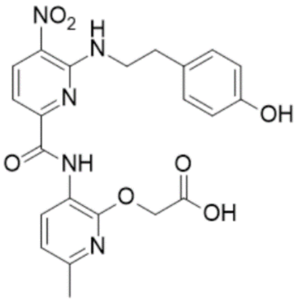

Supplementary Fig. 92. ^{1}H-NMR of NS161Pro

^{1}H NMR (500 MHz, $CDCl_3$) δ 1.44 – 1.44 (s, 9H), 2.39 – 2.42 (s, 3H), 2.54 – 2.58 (s, 3H), 4.88 – 4.92 (s, 2H), 5.05 – 5.18 (d, *J* = 5.3 Hz, 2H), 6.80 – 6.85 (d, *J* = 7.9 Hz, 1H), 7.61 – 7.66 (d, *J* = 8.4 Hz, 1H), 8.44 – 8.48 (s, 1H), 8.61 – 8.71 (m, 3H), 9.03 – 9.08 (t, *J* = 5.4 Hz, 1H), 10.20 – 10.24 (s, 1H); HRMS (m/z): [M]+ calcd. for $C_{24}H_{27}N_7O_6$, 510.2096; found, 510.2095.

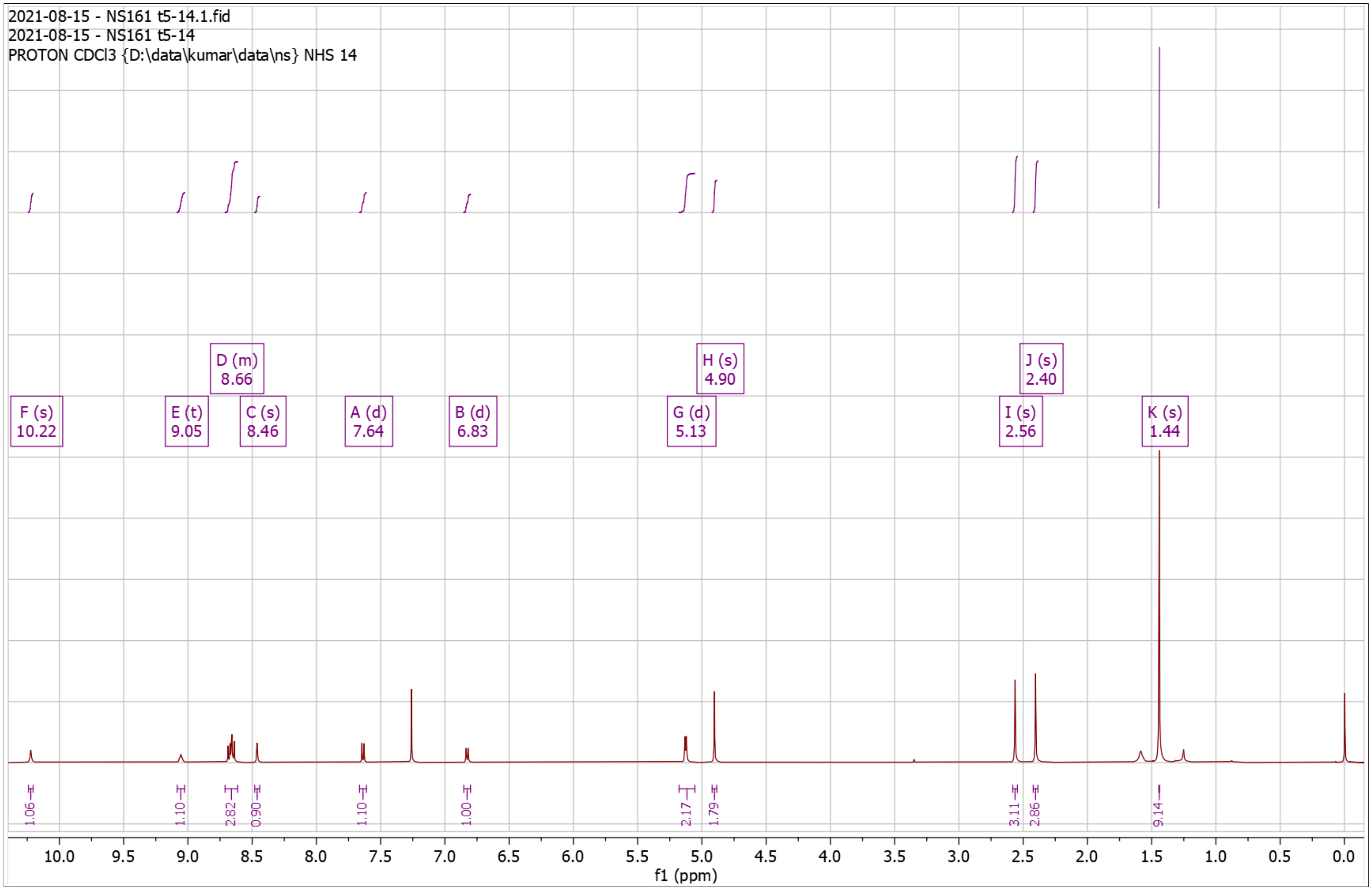

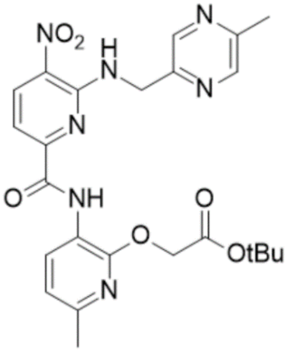

Supplementary Fig. 93. ¹H-NMR of NS161 Dep

¹H NMR (500 MHz, DMSO) δ 2.32 – 2.36 (s, 3H), 2.42 – 2.45 (s, 3H), 4.91 – 4.94 (s, 2H), 4.99 – 5.03 (d, *J* = 5.7 Hz, 2H), 6.90 – 6.95 (d, *J* = 7.9 Hz, 1H), 7.46 – 7.51 (d, *J* = 8.4 Hz, 1H), 8.44 – 8.49 (d, *J* = 7.8 Hz, 2H), 8.57 – 8.60 (s, 1H), 8.68 – 8.73 (d, *J* = 8.3 Hz, 1H), 9.21 – 9.27 (t, *J* = 5.7 Hz, 1H), 10.05 – 10.09 (s, 1H); HRMS (m/z): [M]+ calcd. for $C_{20}H_{19}N_7O_6$, 454.1470; found, 454.1470.

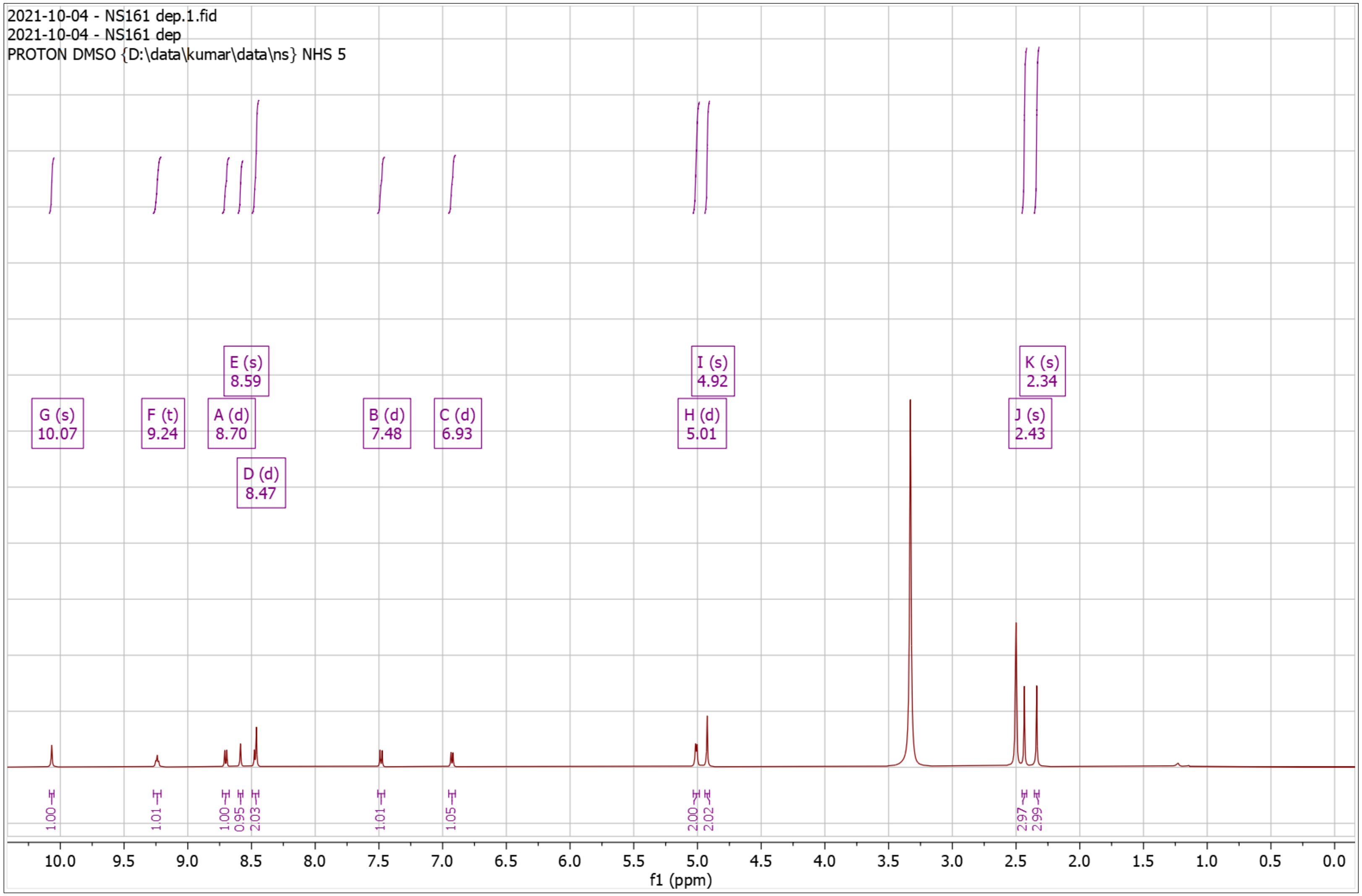

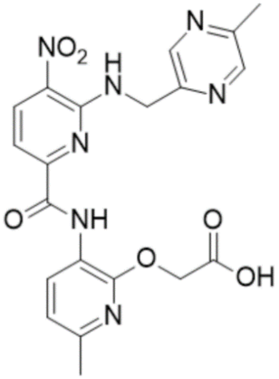

Supplementary Fig. 94. ^{1}H-NMR of NS122 Pro

^{1}H NMR (500 MHz, $CDCl_3$) δ 0.99 – 1.33 (m, 5H), 1.40 – 1.49 (s, 9H), 1.63 – 1.91 (m, 6H), 2.34 – 2.39 (s, 3H), 3.41 – 3.49 (t, J = 6.2 Hz, 2H), 4.85 – 4.87 (s, 2H), 4.95 – 5.01 (t, J = 5.7 Hz, 1H), 6.76 – 6.79 (d, J = 7.9 Hz, 1H), 7.57 – 7.62 (d, J = 7.8 Hz, 1H), 7.84 – 7.89 (d, J = 7.9 Hz, 1H), 8.32 – 8.39 (d, J = 8.1 Hz, 1H), 8.39 – 8.44 (d, J = 8.2 Hz, 1H), 8.65 – 8.71 (d, J = 7.9 Hz, 1H), 9.32 – 9.35 (s, 1H), 10.33 – 10.37 (s, 1H). HRMS (m/z): [M]+ calcd. for $C_{31}H_{36}ClN_7O_7$, 654.2438; found, 654.2424.

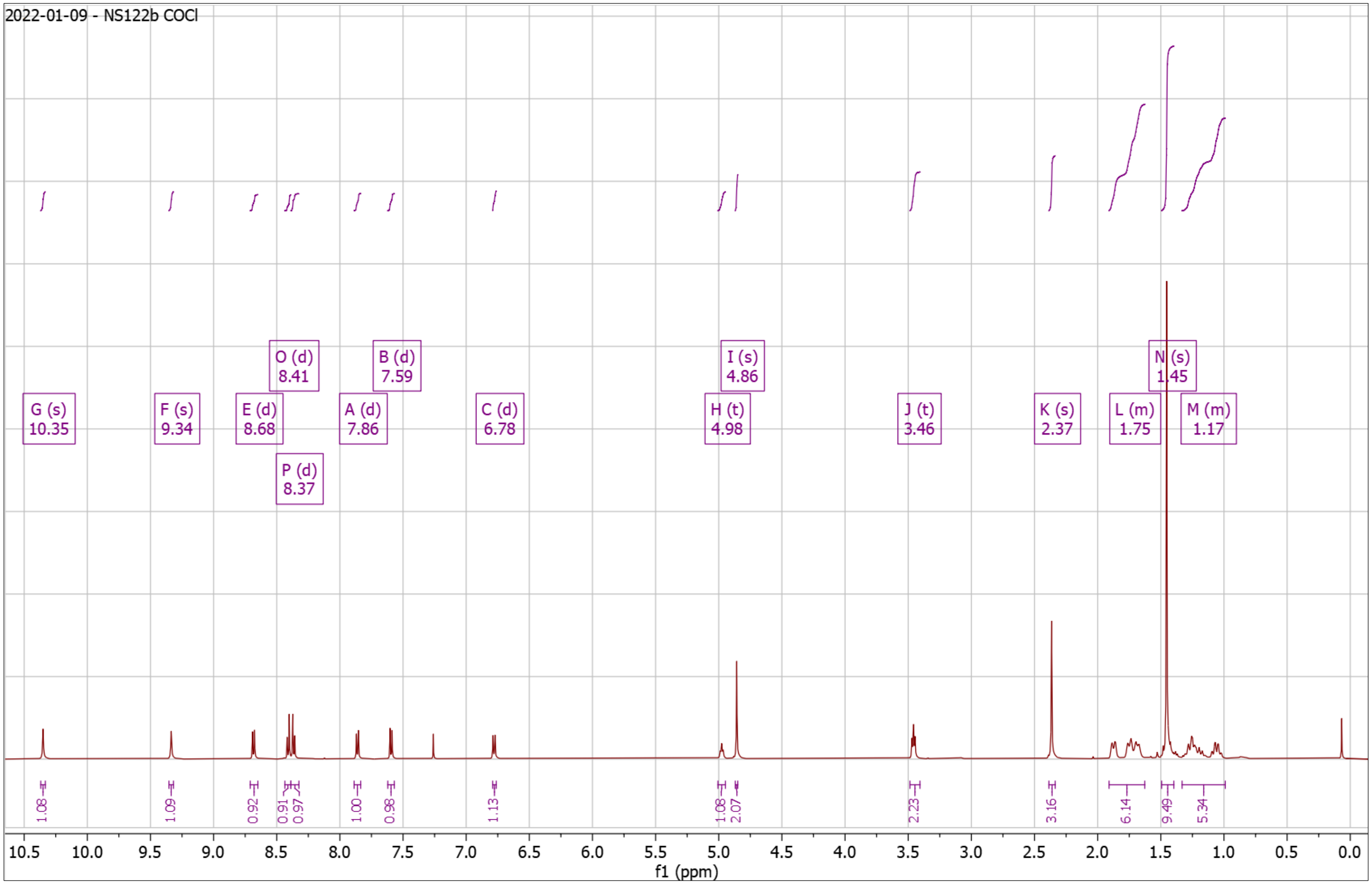

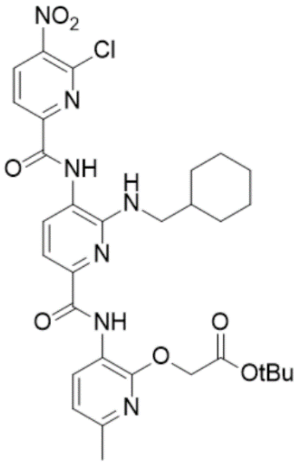

Supplementary Fig. 95. High Resolution Mass Spectrum for NS122 Pro

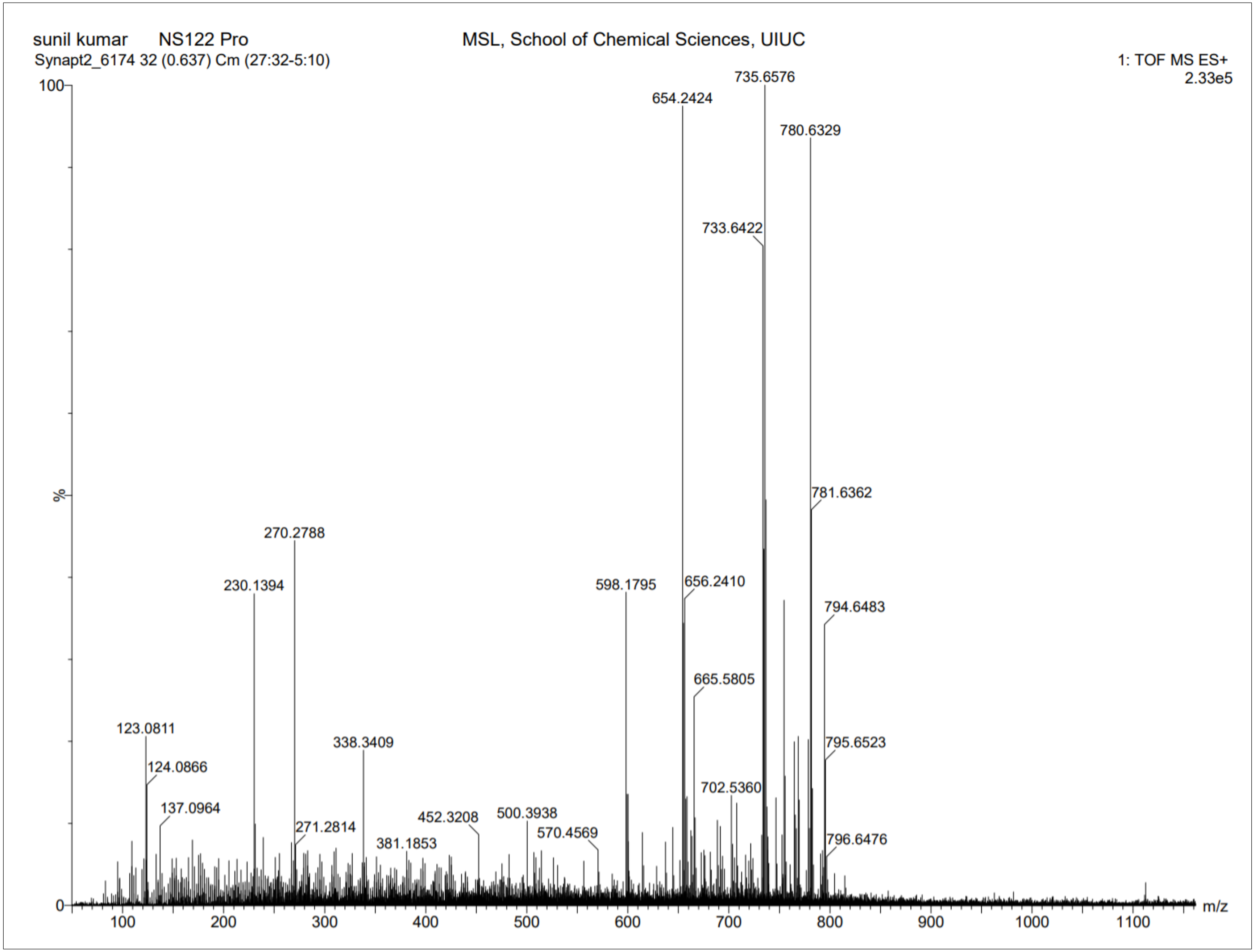

Supplementary Fig. 96. ¹H-NMR of NS122 Dep

¹H NMR (500 MHz, DMSO) δ 0.88 – 1.28 (m, 5H), 1.55 – 1.85 (dd, *J* = 85.2, 19.6 Hz, 6H), 2.33 – 2.36 (s, 3H), 4.94 – 4.98 (s, 2H), 6.67 – 6.78 (s, 1H), 6.92 – 6.97 (d, *J* = 7.9 Hz, 1H), 7.34 – 7.39 (d, *J* = 7.6 Hz, 1H), 7.66 – 7.72 (d, *J* = 7.6 Hz, 1H), 8.28 – 8.33 (d, *J* = 8.2 Hz, 1H), 8.60 – 8.68 (d, *J* = 7.8 Hz, 1H), 8.75 – 8.81 (d, *J* = 8.2 Hz, 1H), 10.24 – 10.33 (s, 1H), 10.33 – 10.43 (s, 1H). HRMS (m/z): [M]+ calcd. for $C_{27}H_{28}ClN_7O_7$, 598.1812; found, 598.1802.

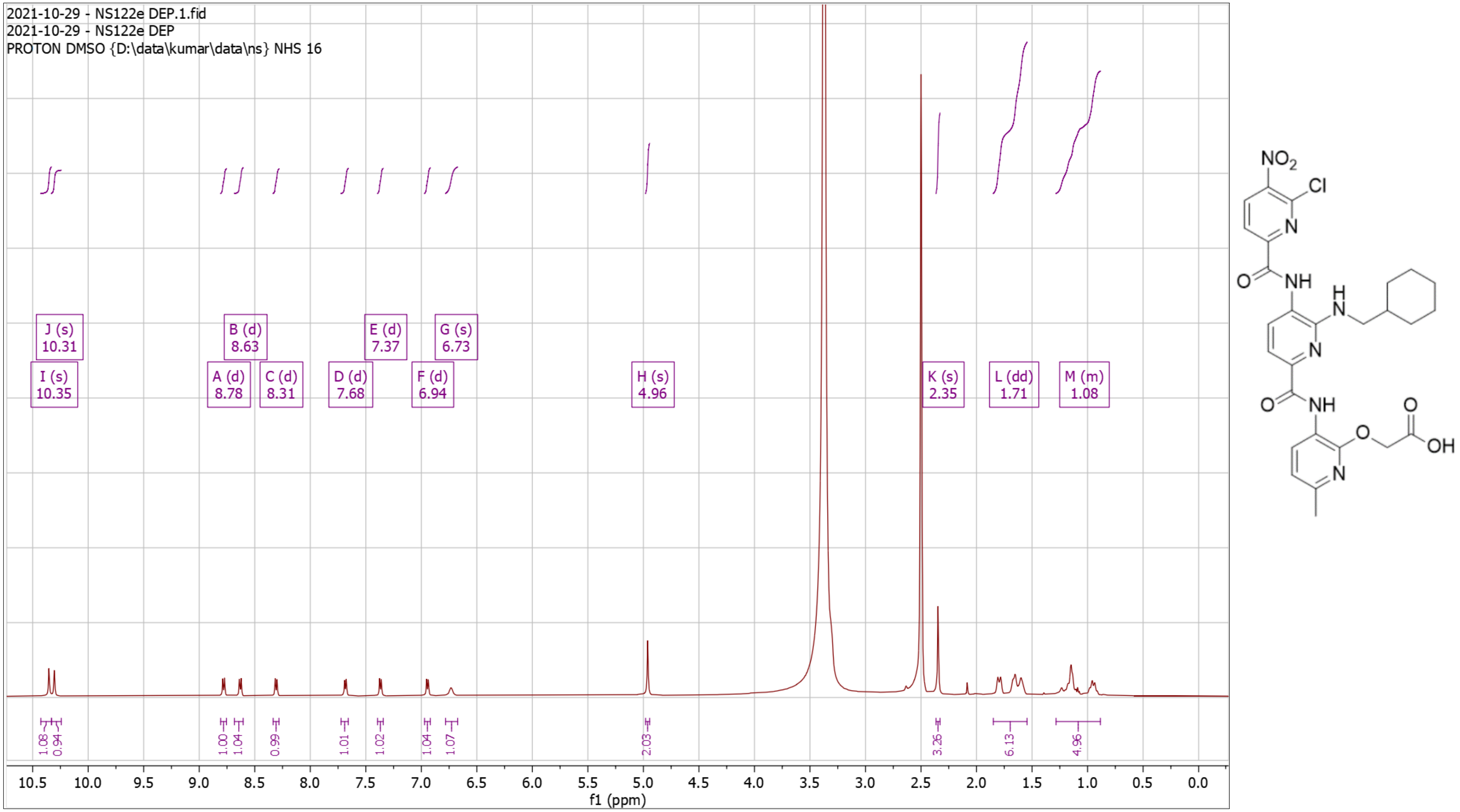

Supplementary Fig. 97. High Resolution Mass Spectrum for NS122 Dep

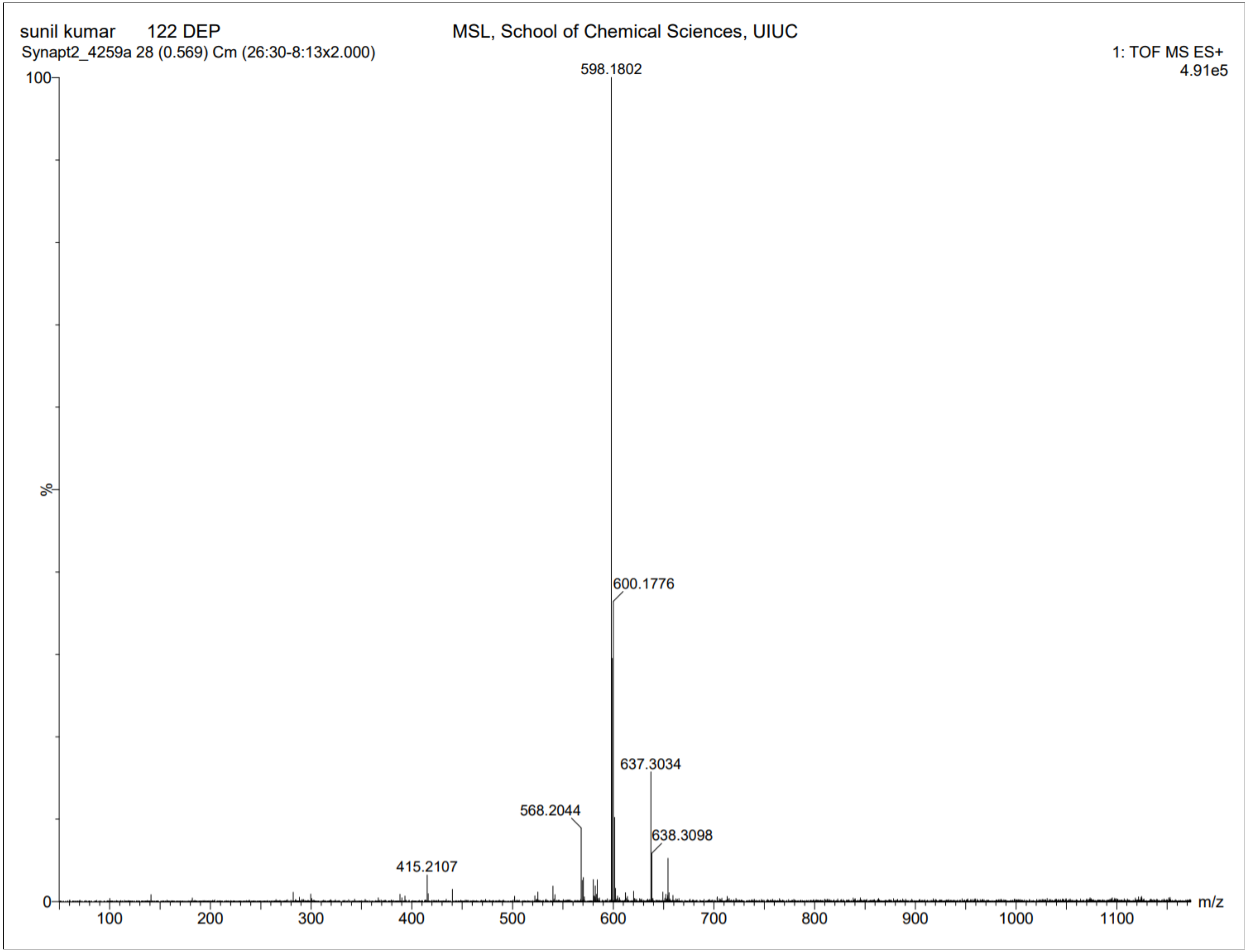

Supplementary Fig. 98. ^{1}H-NMR of NS123 Pro

^{1}H NMR (500 MHz, $CDCl_3$) δ 0.98 – 1.08 (m, 1H), 1.08 – 1.13 (t, J = 7.4 Hz, 3H), 1.15 – 1.34 (m, 4H), 1.44 – 1.47 (s, 9H), 1.64 – 1.92 (m, 8H), 2.36 – 2.40 (s, 3H), 3.44 – 3.49 (dd, J = 7.2, 4.3 Hz, 2H), 3.62 – 3.69 (td, J = 6.9, 5.3 Hz, 2H), 4.84 – 4.88 (s, 2H), 6.78 – 6.83 (d, J = 7.9 Hz, 1H), 7.58 – 7.64 (d, J = 8.4 Hz, 1H), 7.64 – 7.69 (d, J = 7.9 Hz, 1H), 7.92 – 7.98 (d, J = 7.9 Hz, 1H), 8.26 – 8.35 (t, J = 5.5 Hz, 1H), 8.62 – 8.68 (d, J = 8.4 Hz, 1H), 8.70 – 8.75 (d, J = 7.9 Hz, 1H), 9.38 – 9.41 (s, 1H), 10.36 – 10.40 (s, 1H) HRMS (m/z): [M]+ calcd. for $C_{34}H_{44}N_8O_7$, 677.3406; found, 677.3385.

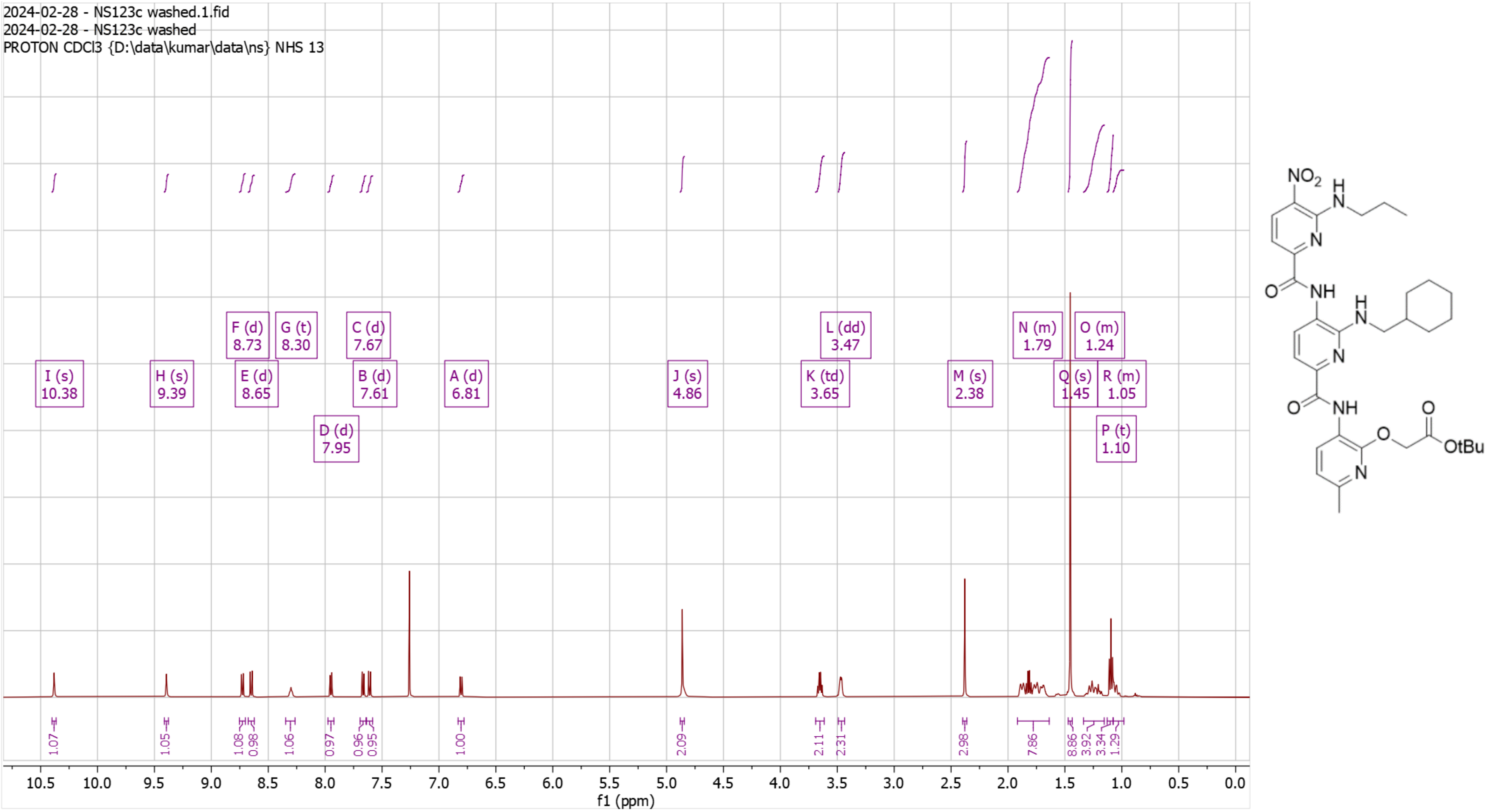

Supplementary Fig. 99. High Resolution Mass Spectrum for NS123 Pro

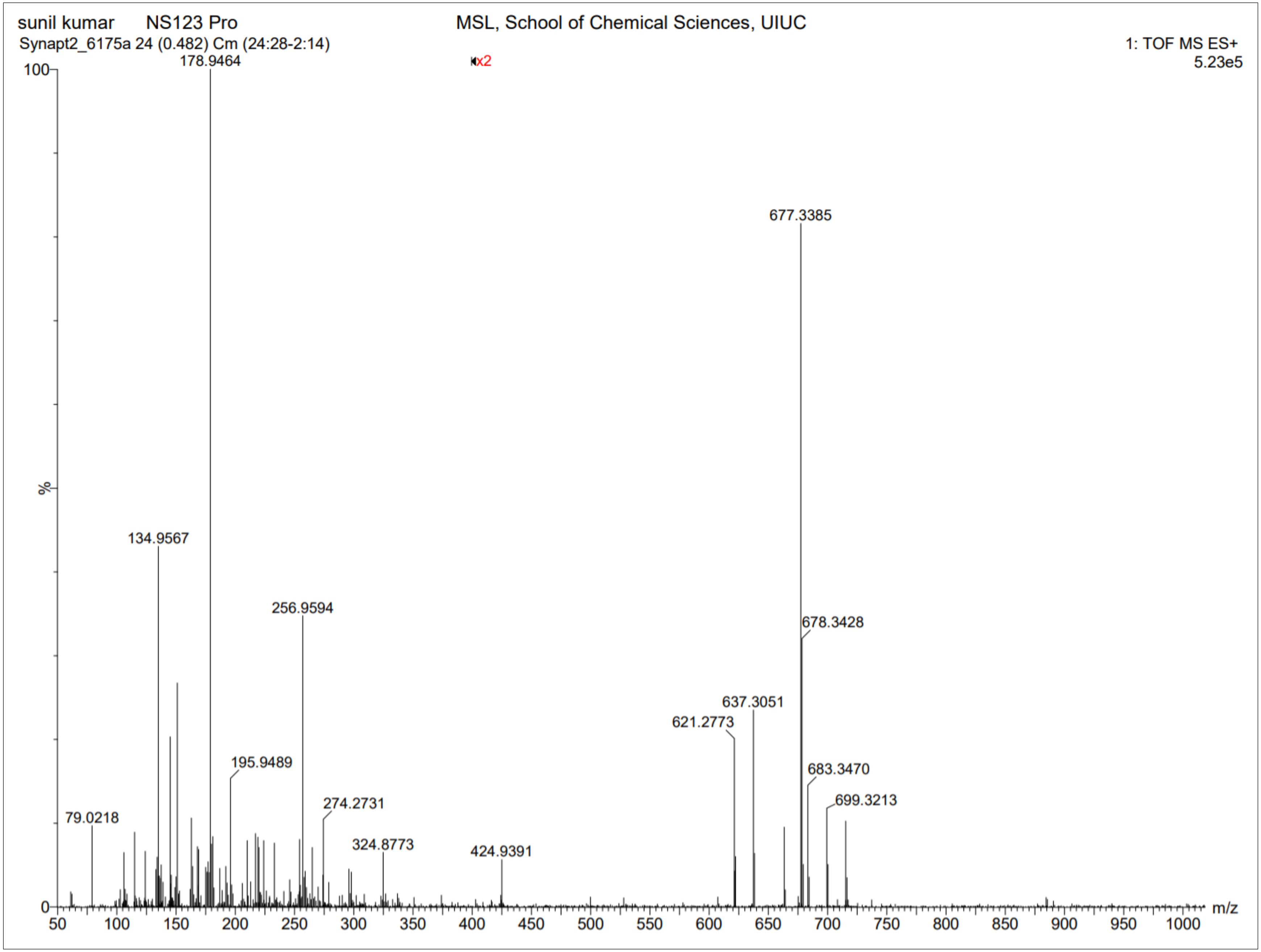

Supplementary Fig. 100. ^{1}H-NMR of NS123 Dep

^{1}H NMR (500 MHz, DMSO) δ 0.93 – 0.98 (t, J = 7.4 Hz, 3H), 1.10 – 1.31 (m, 7H), 1.64 – 1.88 (m, 7H), 2.33 – 2.34 (s, 3H), 3.68 – 3.80 (q, J = 6.6 Hz, 2H), 4.88 – 4.95 (s, 2H), 6.67 – 6.77 (t, J = 5.5 Hz, 1H), 6.87 – 6.94 (d, J = 8.0 Hz, 1H), 7.34 – 7.40 (d, J = 8.5 Hz, 1H), 7.40 – 7.46 (d, J = 7.7 Hz, 1H), 7.90 – 7.99 (t, J = 8.1 Hz, 1H), 8.49 – 8.56 (t, J = 5.8 Hz, 1H), 8.58 – 8.68 (dd, J = 9.8, 8.1 Hz, 2H), 9.76 – 9.86 (s, 1H), 10.35 – 10.41 (s, 1H). HRMS (m/z): [M]+ calcd. for $C_{30}H_{36}N_8O_7$, 621.2780; found, 621.2772.

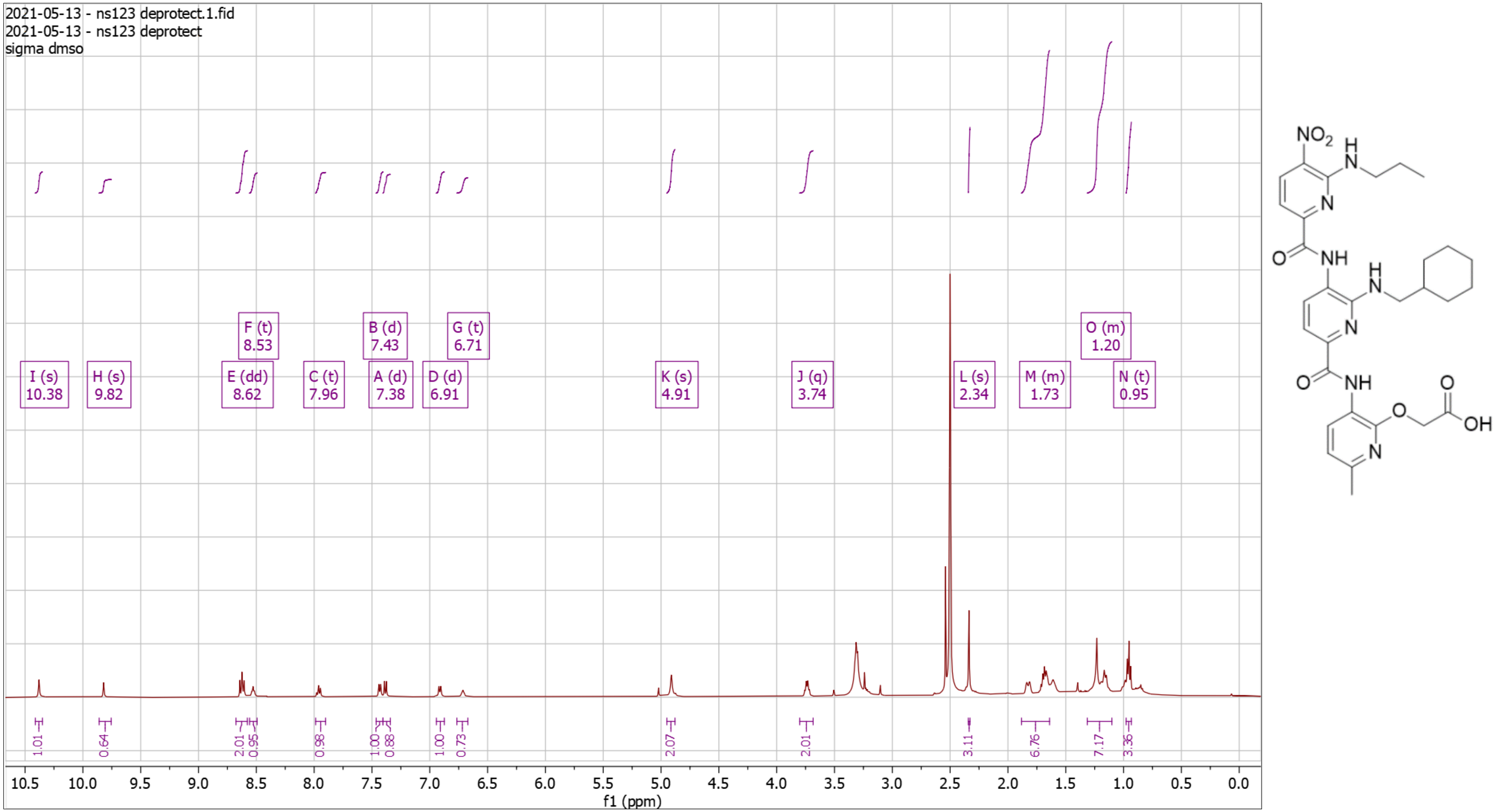

Supplementary Fig. 101. High Resolution Mass Spectrum for NS123 Dep

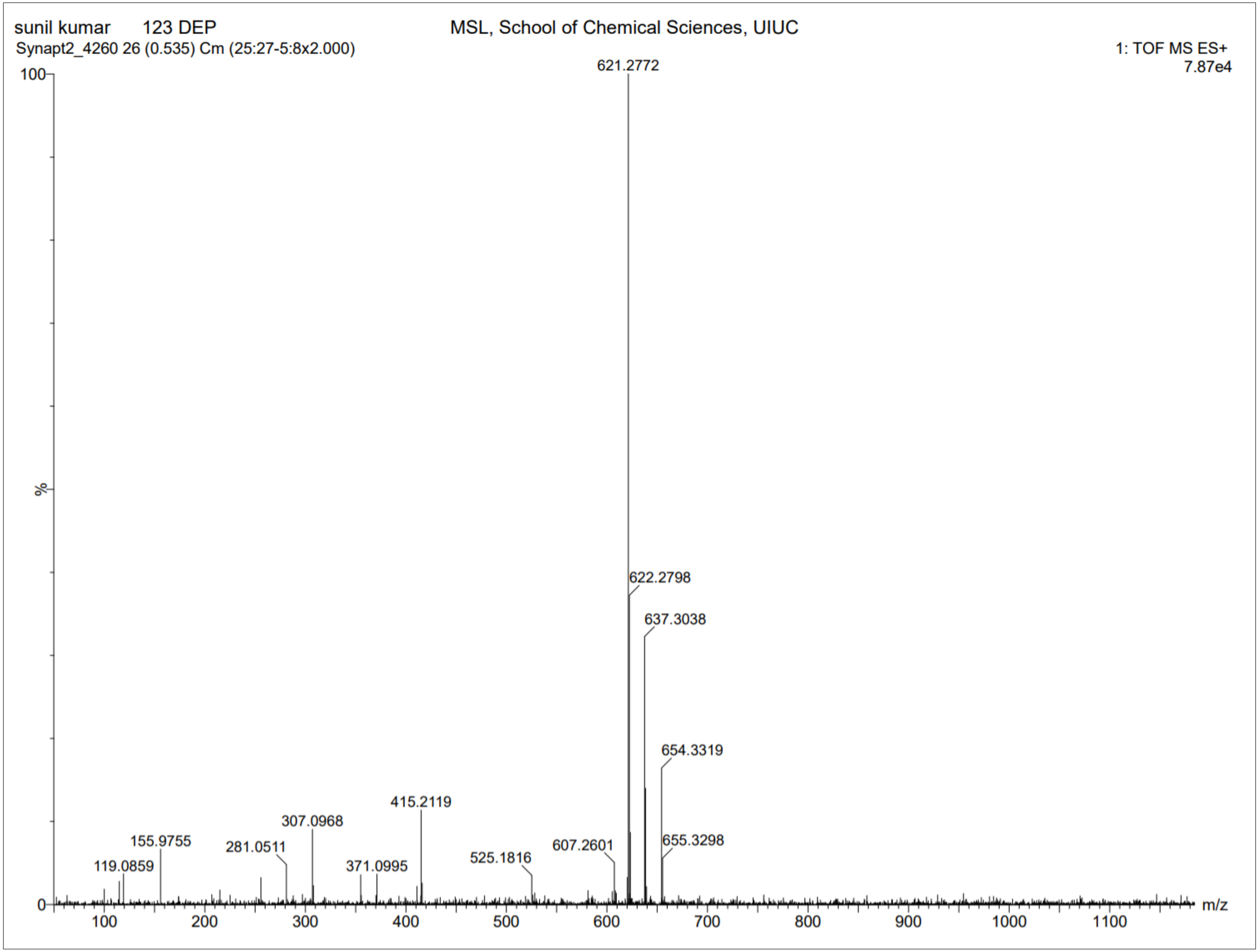

Supplementary Fig. 102. ^{1}H-NMR of NS125 Pro

^{1}H NMR (500 MHz, $CDCl_3$) δ 0.84 – 1.20 (m, 5H), 1.20 – 1.22 (s, 9H), 1.43 – 1.46 (s, 9H), 1.64 – 1.92 (m, 6H), 2.37 – 2.40 (s, 3H), 3.38 – 3.43 (q, *J* = 7.6 Hz, 2H), 3.43 – 3.45 (d, *J* = 6.8 Hz, 2H), 3.77 – 3.88 (q, *J* = 6.8 Hz, 2H), 4.81 – 4.86 (t, 1H), 4.86 – 4.90 (s, 2H), 6.78 – 6.83 (d, *J* = 7.9 Hz, 1H), 7.56 – 7.61 (d, *J* = 7.8 Hz, 1H), 7.65 – 7.70 (d, *J* = 8.4 Hz, 1H), 7.80 – 7.86 (d, *J* = 7.8 Hz, 1H), 8.34 – 8.38 (t, *J* = 7.8 Hz, 1H), 8.61 – 8.66 (d, *J* = 8.5 Hz, 1H), 8.72 – 8.77 (d, *J* = 7.9 Hz, 1H), 10.25 – 10.29 (s, 1H), 10.44 – 10.48 (s, 1H). HRMS (m/z): [M]+ calcd. for $C_{38}H_{51}N_9O_9$, 778.3883; found, 778.3856.

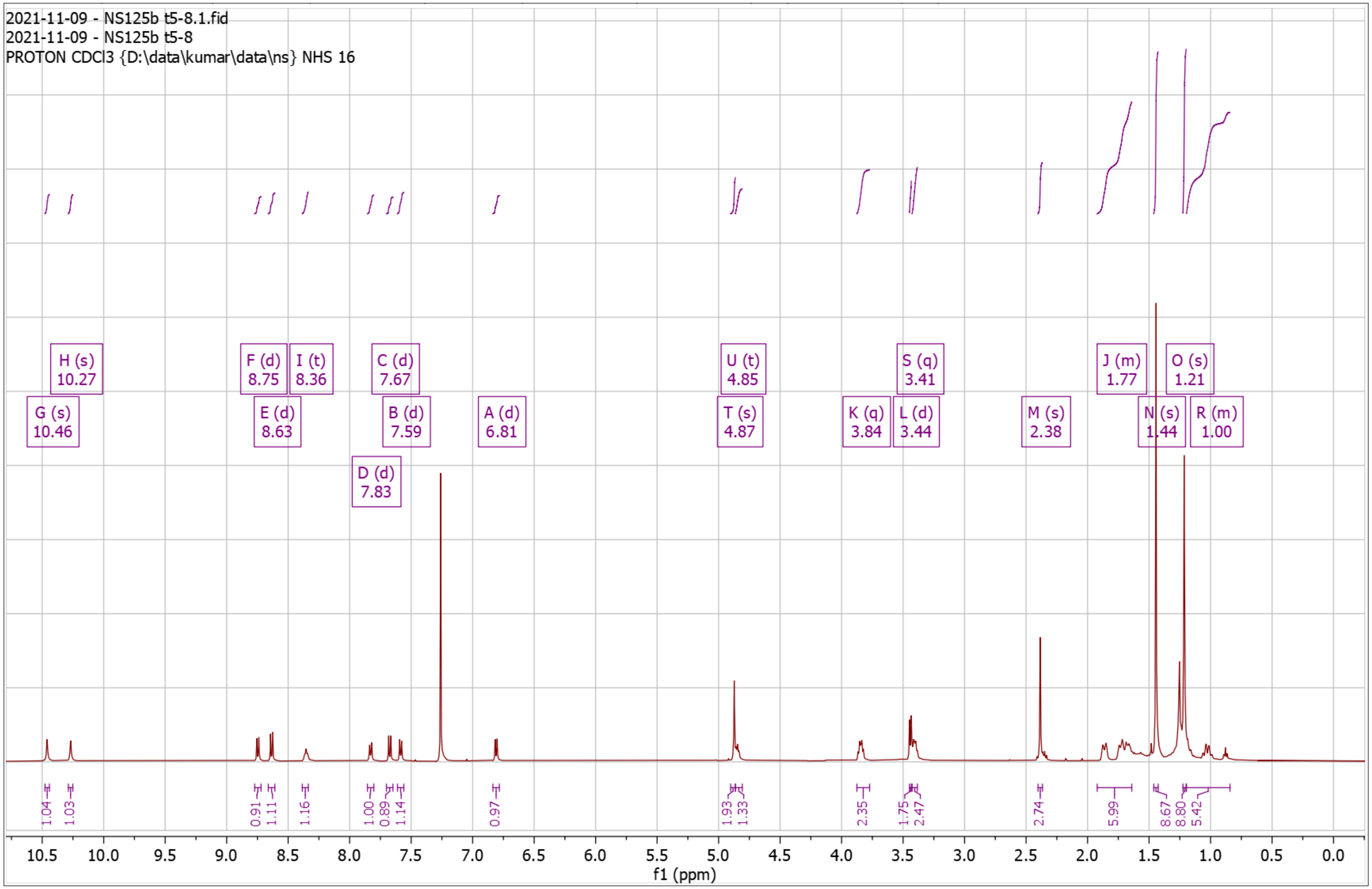

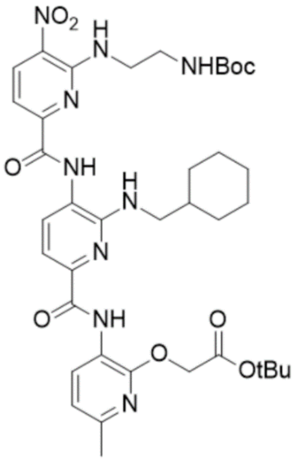

Supplementary Fig. 103. High Resolution Mass Spectrum for NS125 Pro

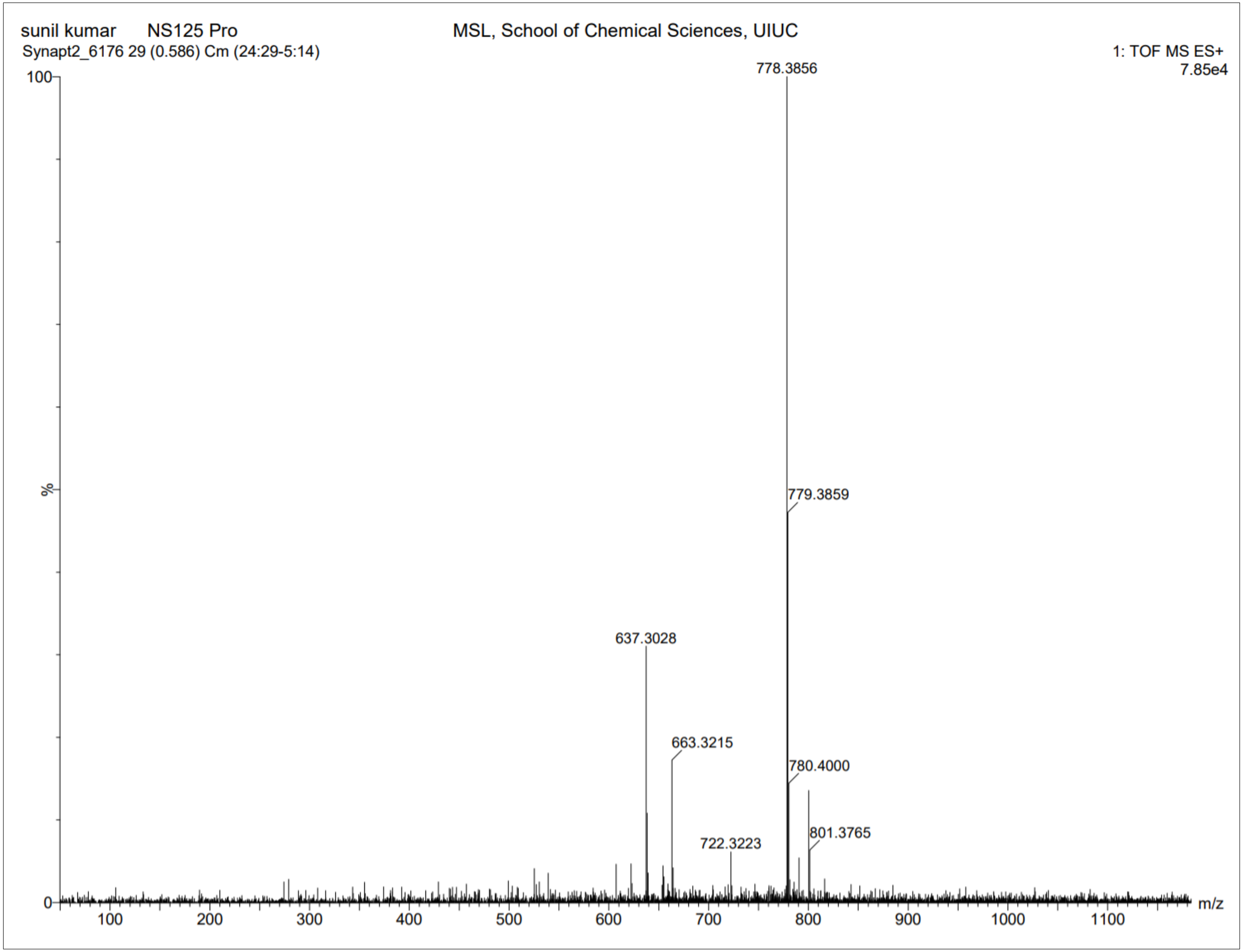

Supplementary Fig. 104. ^{1}H-NMR of NS125 Dep

^{1}H NMR (500 MHz, DMSO) δ 0.89 – 1.27 (dq, *J* = 98.6, 11.9 Hz, 6H), 1.56 – 1.87 (m, 7H), 2.32 – 2.35 (s, 3H), 3.07 – 3.13 (t, *J* = 5.7 Hz, 2H), 3.98 – 4.09 (q, *J* = 5.9 Hz, 2H), 4.92 – 4.96 (s, 2H), 6.79 – 6.83 (t, 1H), 6.91 – 6.96 (d, *J* = 7.9 Hz, 1H), 7.38 – 7.43 (d, *J* = 7.7 Hz, 1H), 7.44 – 7.49 (d, *J* = 8.4 Hz, 1H), 7.74 – 7.79 (d, *J* = 7.7 Hz, 1H), 8.59 – 8.73 (m, 3H), 10.33 – 10.36 (s, 1H). HRMS (m/z): [M]+ calcd. for $C_{29}H_{35}N_{9}O_{7}$, 622.2732; found, 622.2714.

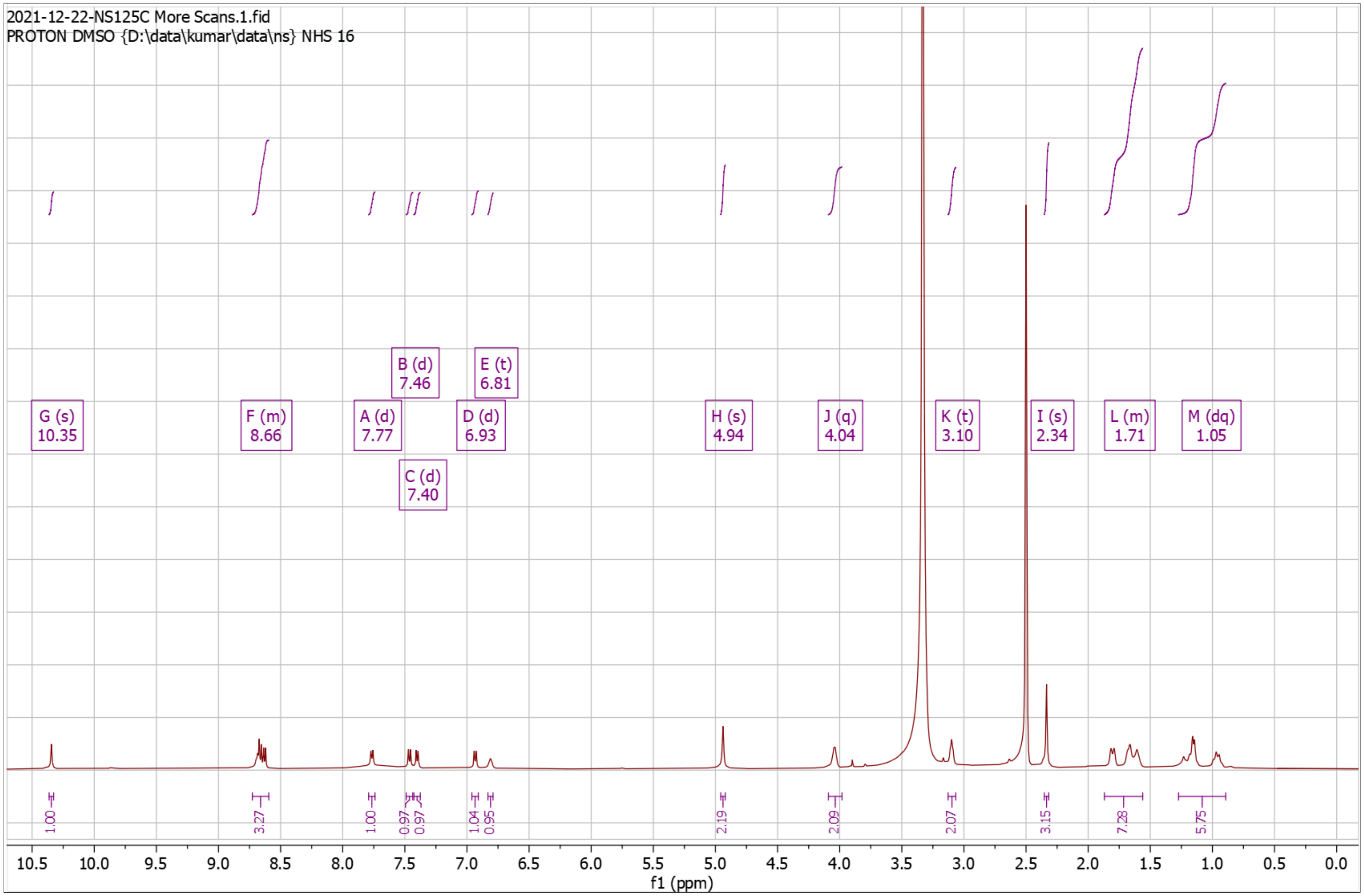

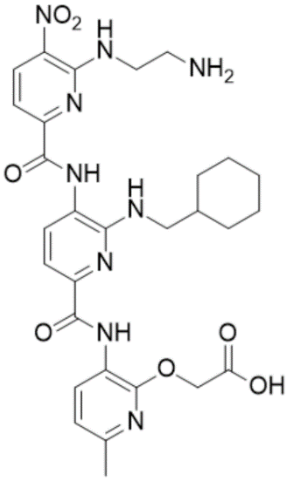

Supplementary Fig. 105. High Resolution Mass Spectrum for NS125 Dep

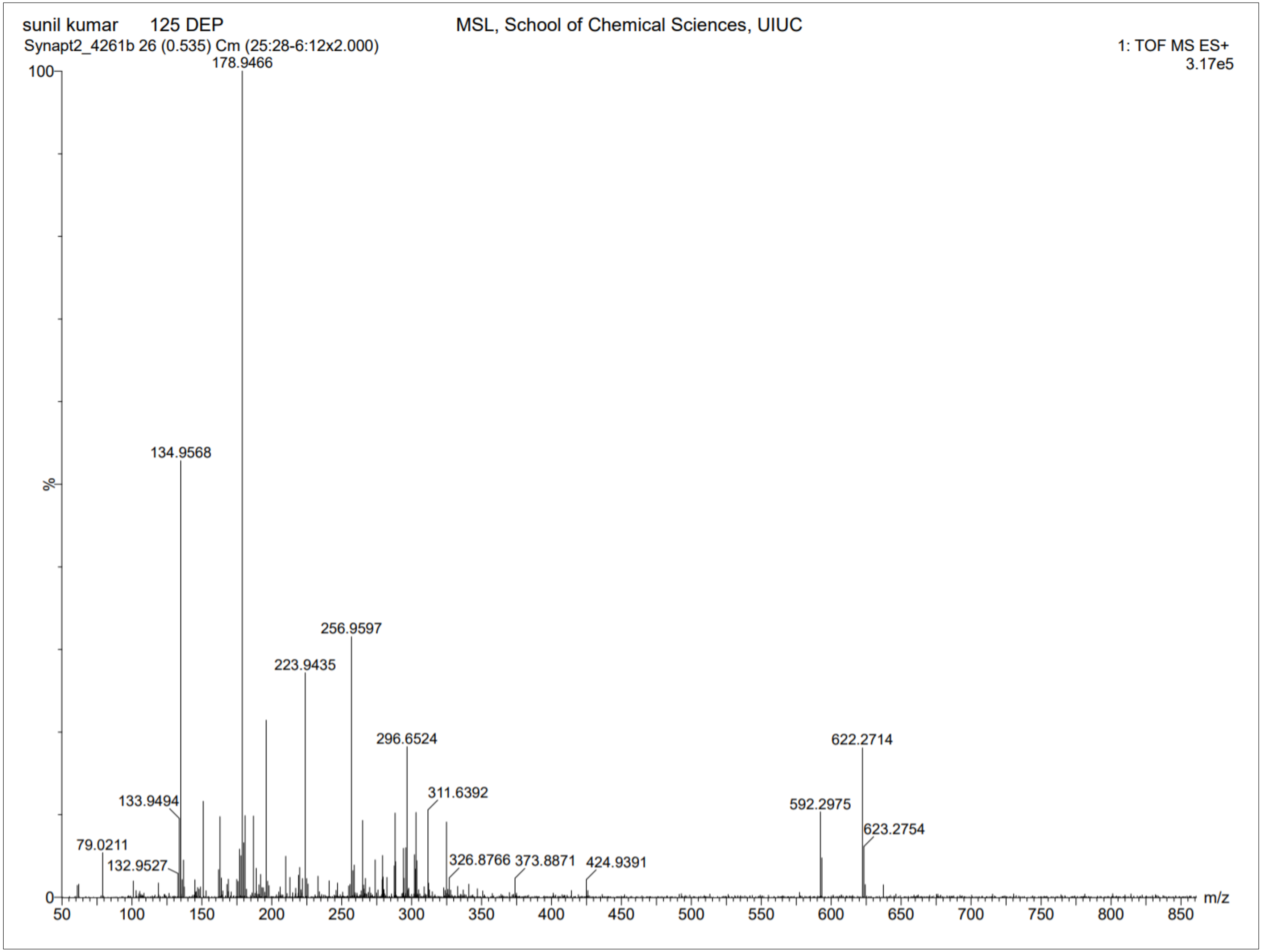

Supplementary Fig. 106. ^{1}H-NMR of NS126 Pro

^{1}H NMR (500 MHz, $CDCl_3$) δ 1.00 – 1.39 (m, 10H), 1.43 – 1.47 (s, 9H), 1.65 – 1.94 (m, 12H), 2.36 – 2.42 (s, 3H), 3.42 – 3.50 (t, *J* = 6.2 Hz, 2H), 3.50 – 3.59 (t, *J* = 6.1 Hz, 2H), 4.81 – 4.88 (s, 2H), 4.88 – 4.94 (t, *J* = 6.0 Hz, 1H), 6.76 – 6.85 (d, *J* = 8.0 Hz, 1H), 7.54 – 7.63 (d, *J* = 8.4 Hz, 1H), 7.63 – 7.71 (d, *J* = 7.8 Hz, 1H), 7.85 – 7.96 (d, *J* = 7.9 Hz, 1H), 8.30 – 8.41 (t, *J* = 5.7 Hz, 1H), 8.58 – 8.69 (d, *J* = 8.4 Hz, 1H), 8.69 – 8.78 (d, *J* = 7.9 Hz, 1H), 9.28 – 9.43 (s, 1H), 10.29 – 10.47 (s, 1H). HRMS (m/z): [M]+ calcd. for $C_{38}H_{50}N_8O_7$, 731.3875; found, 731.3856.

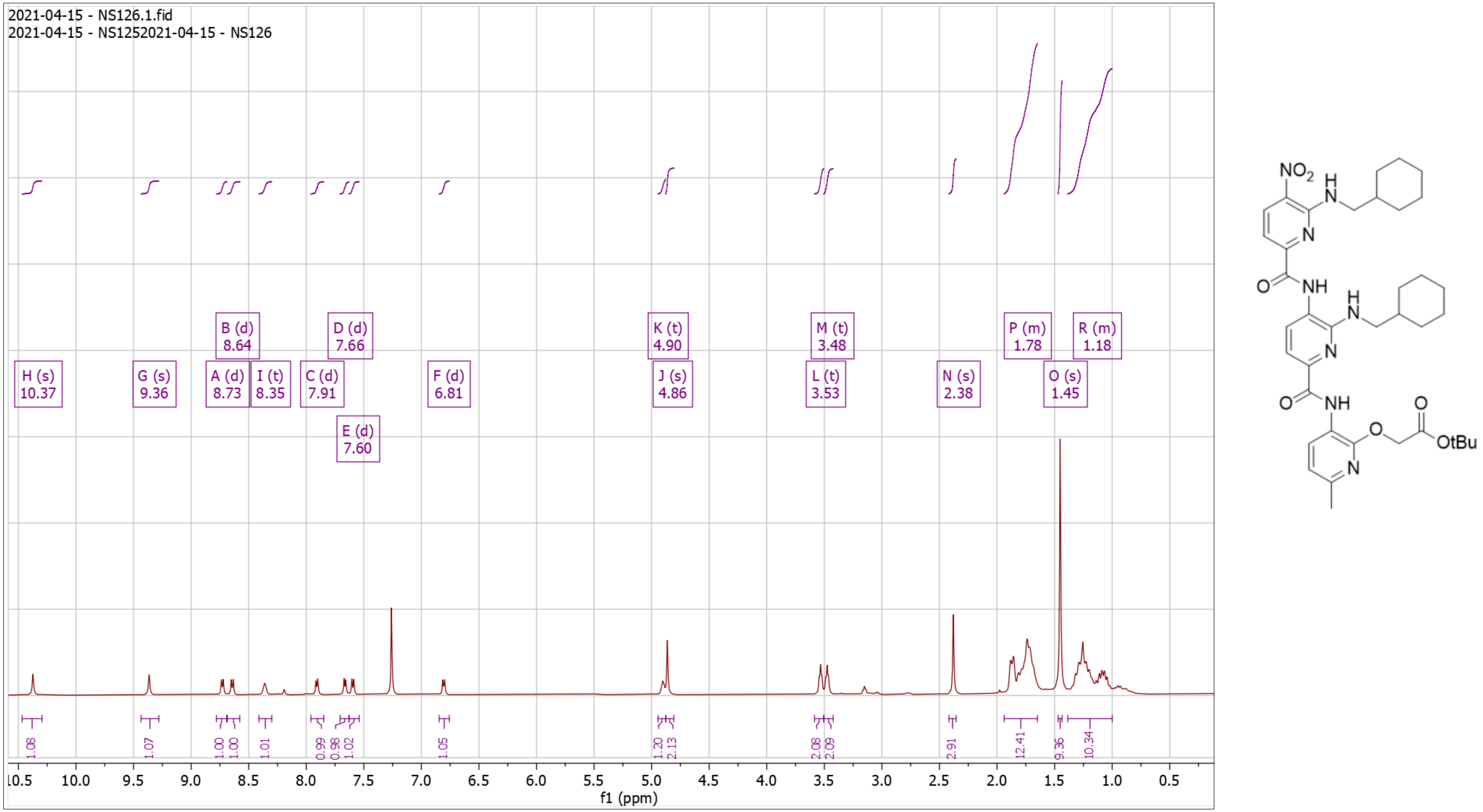

Supplementary Fig. 107. High Resolution Mass Spectrum for NS126 Pro

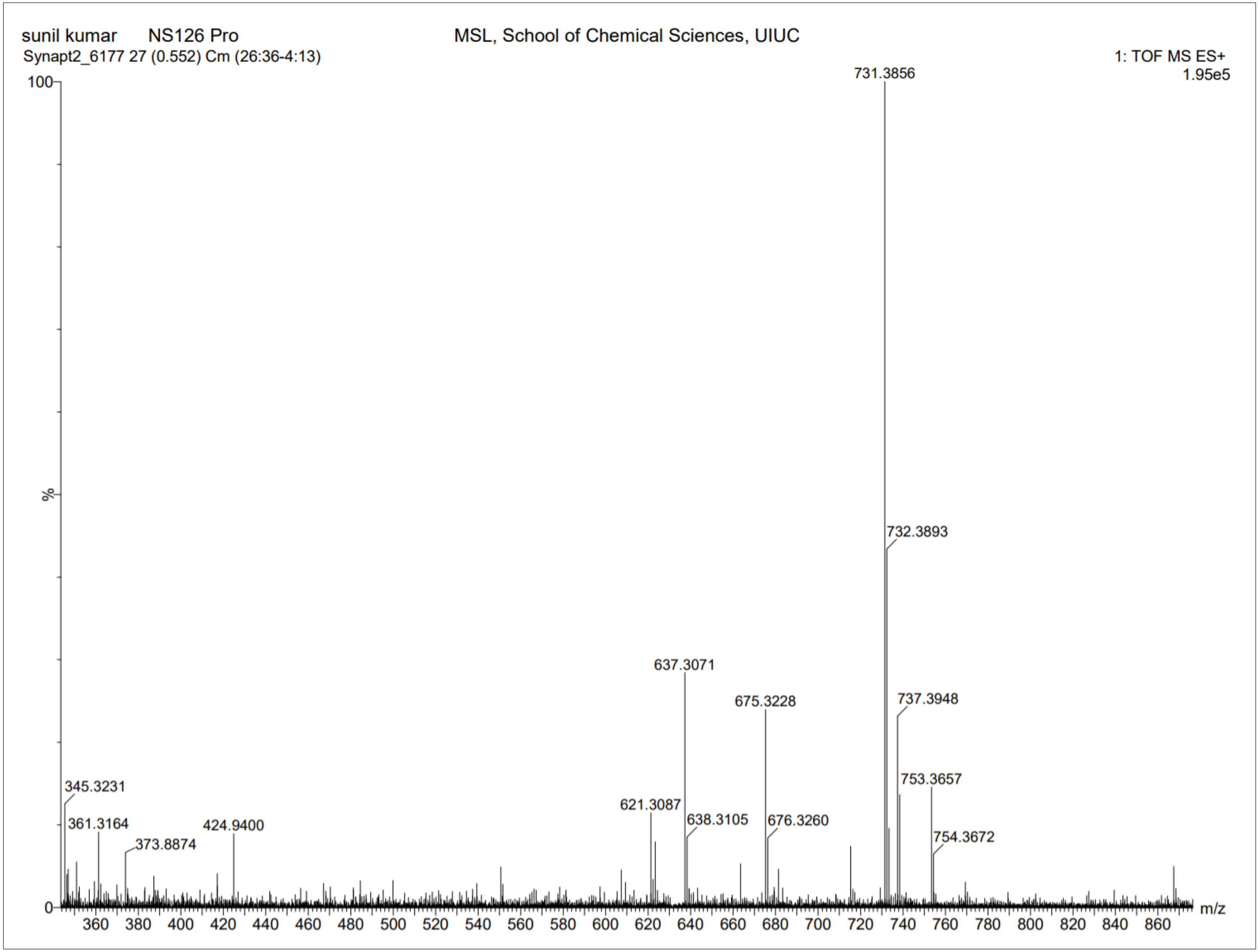

Supplementary Fig. 108. ^{1}H-NMR of NS126 Dep

^{1}H NMR (500 MHz, DMSO) δ 0.86 – 1.27 (m, 10H), 1.56 – 1.90 (m, 12H), 2.31 – 2.37 (s, 3H), 3.63 – 3.69 (t, *J* = 6.4 Hz, 2H), 4.92 – 4.96 (s, 2H), 6.72 – 6.80 (t, *J* = 5.8 Hz, 1H), 6.91 – 6.96 (d, *J* = 8.0 Hz, 1H), 7.36 – 7.48 (dd, *J* = 26.2, 8.1 Hz, 2H), 7.92 – 7.97 (d, *J* = 7.8 Hz, 1H), 8.46 – 8.53 (d, *J* = 6.2 Hz, 1H), 8.60 – 8.66 (dd, *J* = 8.2, 4.7 Hz, 2H), 9.78 – 9.81 (s, 1H), 10.35 – 10.38 (s, 1H). HRMS (m/z): [M]+ calcd. for $C_{34}H_{42}N_8O_7$, 675.3249; found, 675.3245.

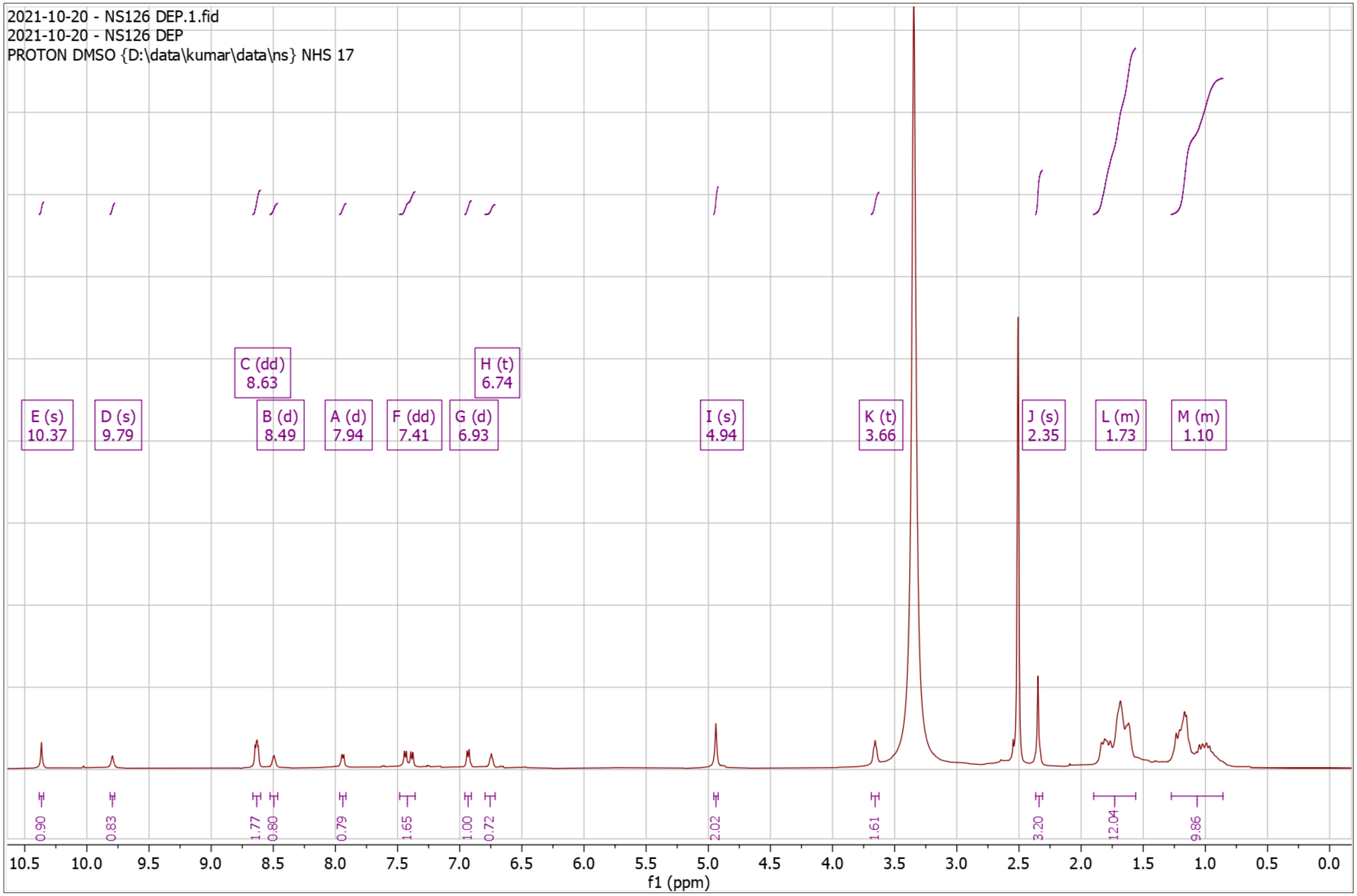

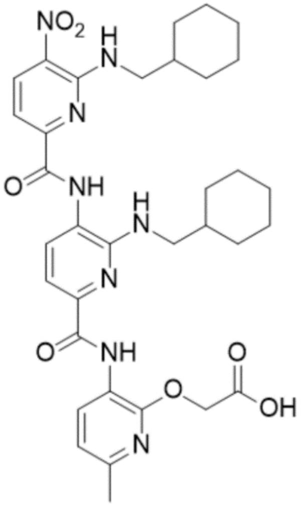

Supplementary Fig. 109. High Resolution Mass Spectrum for NS126 Dep

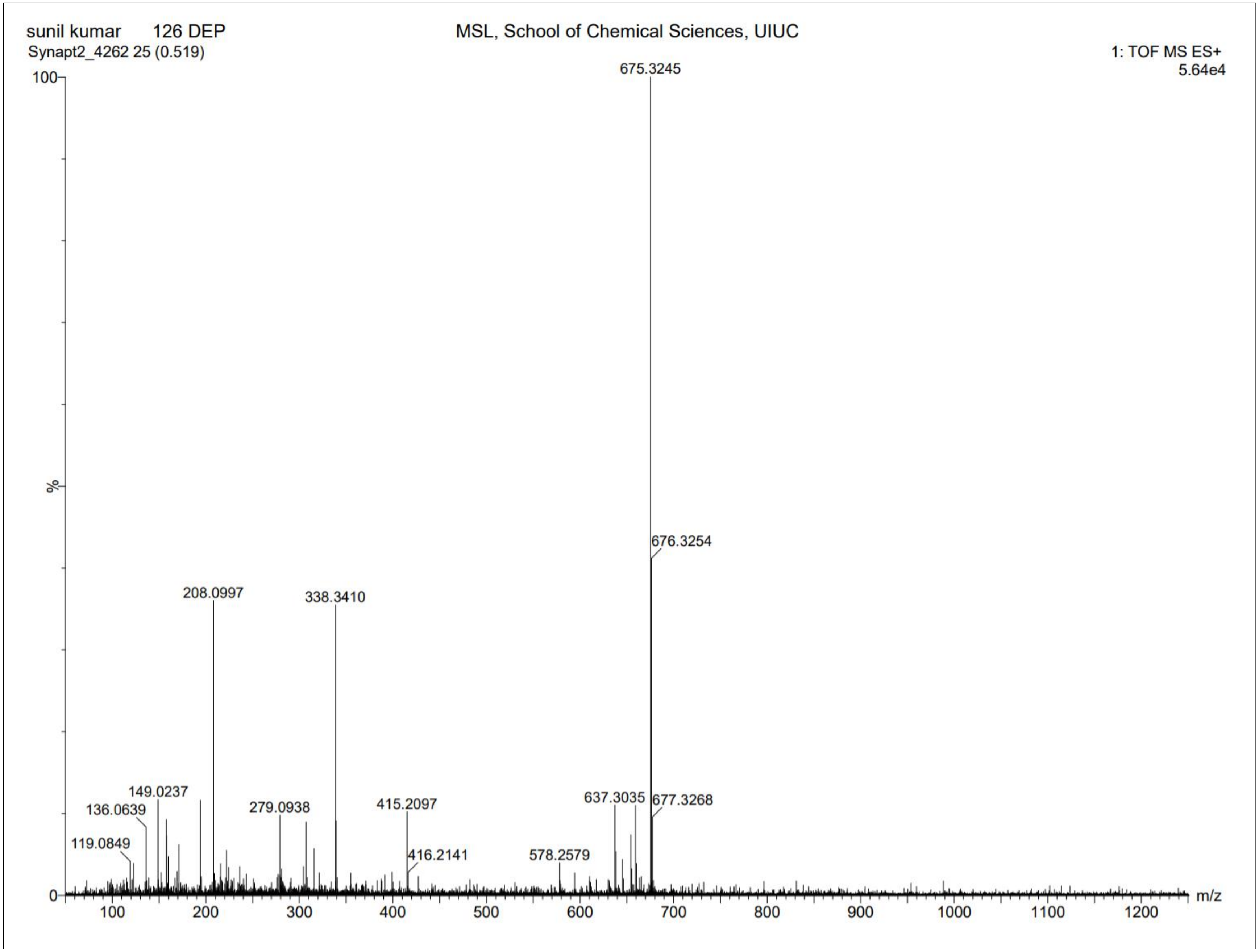

Supplementary Fig. 110. ^{1}H-NMR of NS127 Pro

^{1}H NMR (500 MHz, DMSO) δ 0.80 – 1.17 (m, 5H), 1.38 – 1.42 (s, 9H), 1.55 – 1.82 (m, 6H), 2.31 – 2.36 (s, 3H), 2.94 – 3.04 (t, *J* = 7.2 Hz, 2H), 3.30 – 3.31 (t, 3H), 3.99 – 4.07 (q, *J* = 6.8 Hz, 2H), 4.86 – 4.89 (s, 2H), 6.73 – 6.78 (t, *J* = 6.2 Hz, 1H), 6.92 – 6.97 (d, *J* = 7.9 Hz, 1H), 7.13 – 7.33 (m, 5H), 7.35 – 7.42 (d, *J* = 8.4 Hz, 1H), 7.42 – 7.47 (d, *J* = 7.7 Hz, 1H), 7.90 – 7.97 (d, *J* = 7.8 Hz, 1H), 8.52 – 8.58 (t, *J* = 5.8 Hz, 1H), 8.60 – 8.66 (dd, *J* = 8.1, 3.4 Hz, 2H), 9.82 – 9.87 (s, 1H), 10.33 – 10.37 (s, 1H). HRMS (m/z): [M]+ calcd. for $C_{39}H_{46}N_8O_7$, 739.3562; found, 739.3551.

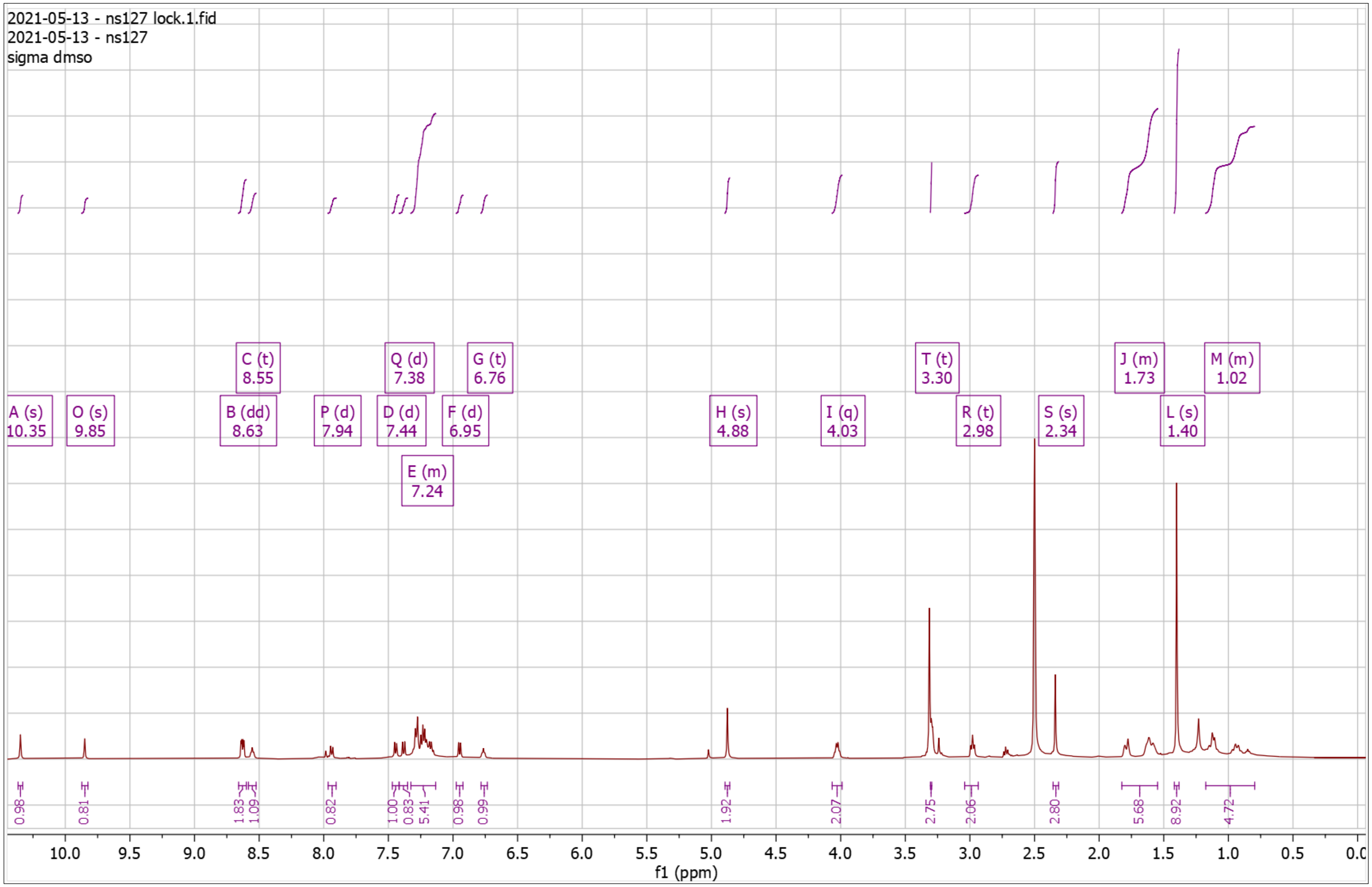

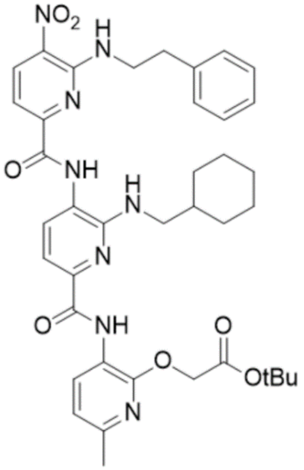

Supplementary Fig. 111. High Resolution Mass Spectrum for NS127 Pro

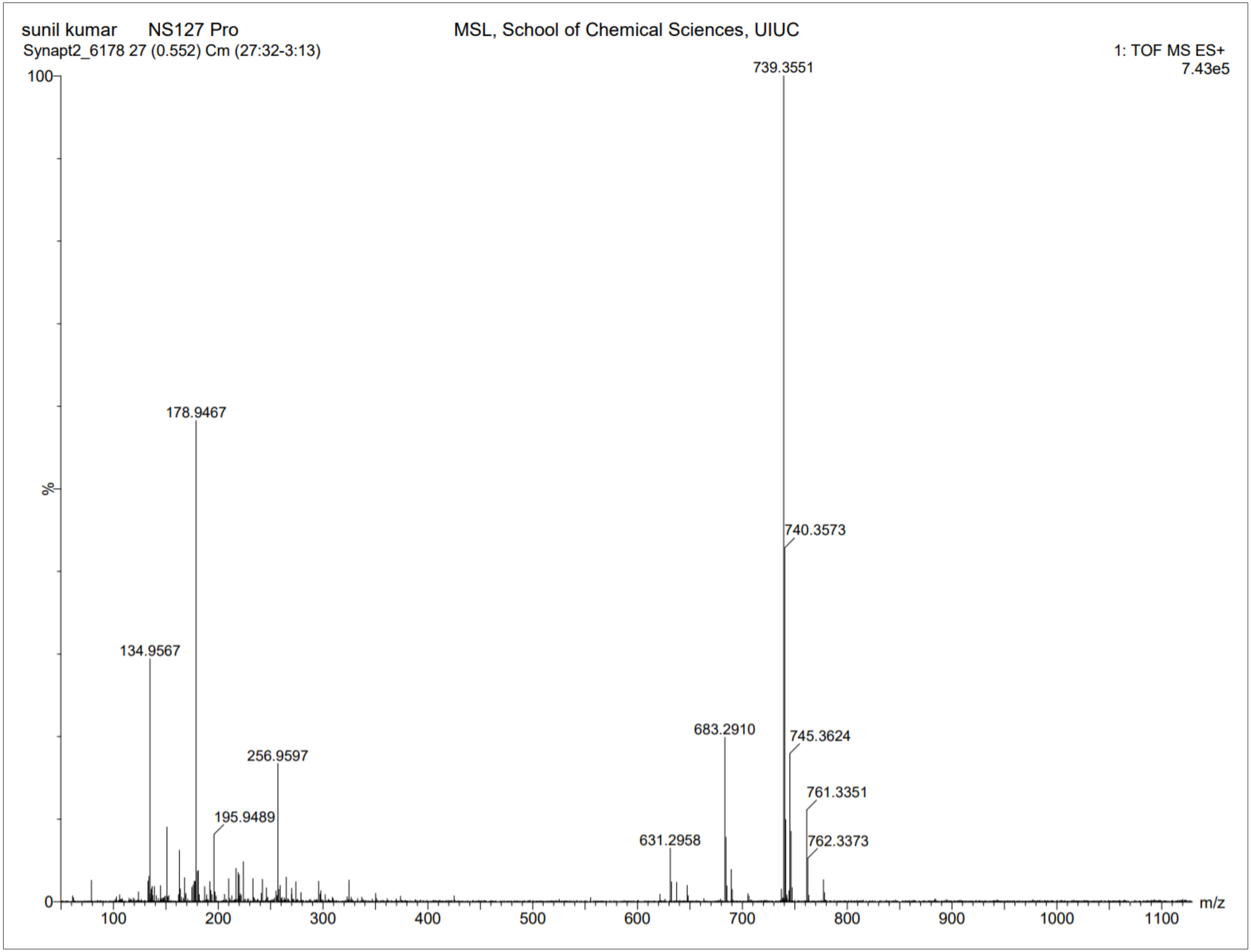

Supplementary Fig. 112. ^{1}H-NMR of NS127 Dep

^{1}H NMR (500 MHz, DMSO) δ 0.89 – 1.16 (m, 5H), 1.52 – 1.86 (m, 6H), 2.31 – 2.40 (s, 3H), 2.92 – 3.04 (t, *J* = 7.2 Hz, 2H), 3.23 – 3.33 (t, 2H), 3.99 – 4.07 (q, *J* = 6.7 Hz, 2H), 4.91 – 5.00 (s, 2H), 6.69 – 6.79 (t, *J* = 5.7 Hz, 1H), 6.88 – 6.98 (d, *J* = 7.9 Hz, 1H), 7.12 – 7.31 (m, 5H), 7.35 – 7.41 (d, *J* = 8.4 Hz, 1H), 7.41 – 7.48 (d, *J* = 7.7 Hz, 1H), 7.86 – 7.99 (d, *J* = 7.7 Hz, 1H), 8.48 – 8.58 (t, *J* = 5.8 Hz, 1H), 8.59 – 8.66 (dd, *J* = 8.2, 4.5 Hz, 2H), 9.80 – 9.88 (s, 1H), 10.33 – 10.39 (s, 1H). HRMS (m/z): [M]+ calcd. for $C_{35}H_{38}N_8O_7$, 683.2936; found, 683.2925.

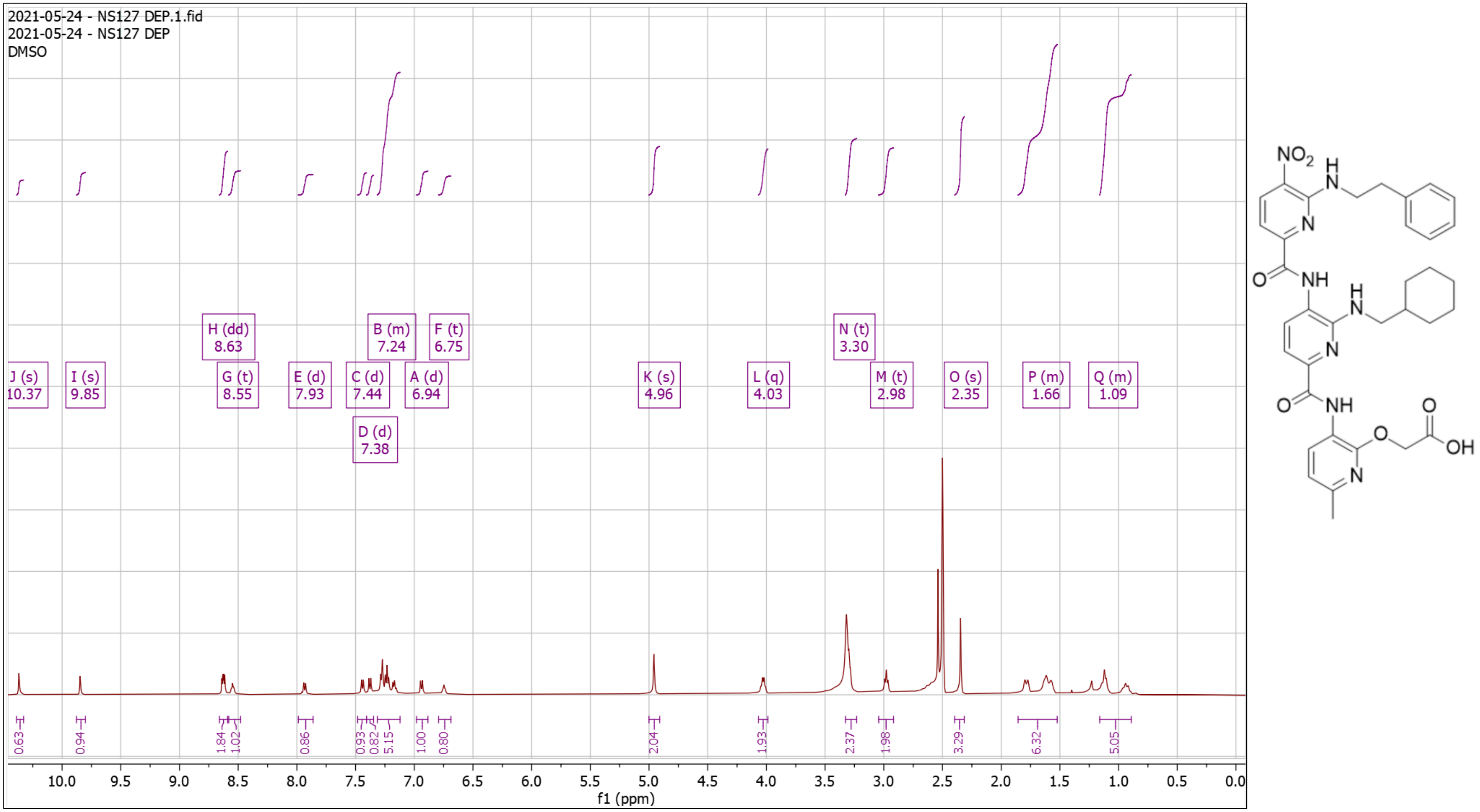

Supplementary Fig. 113. High Resolution Mass Spectrum for NS127 Dep

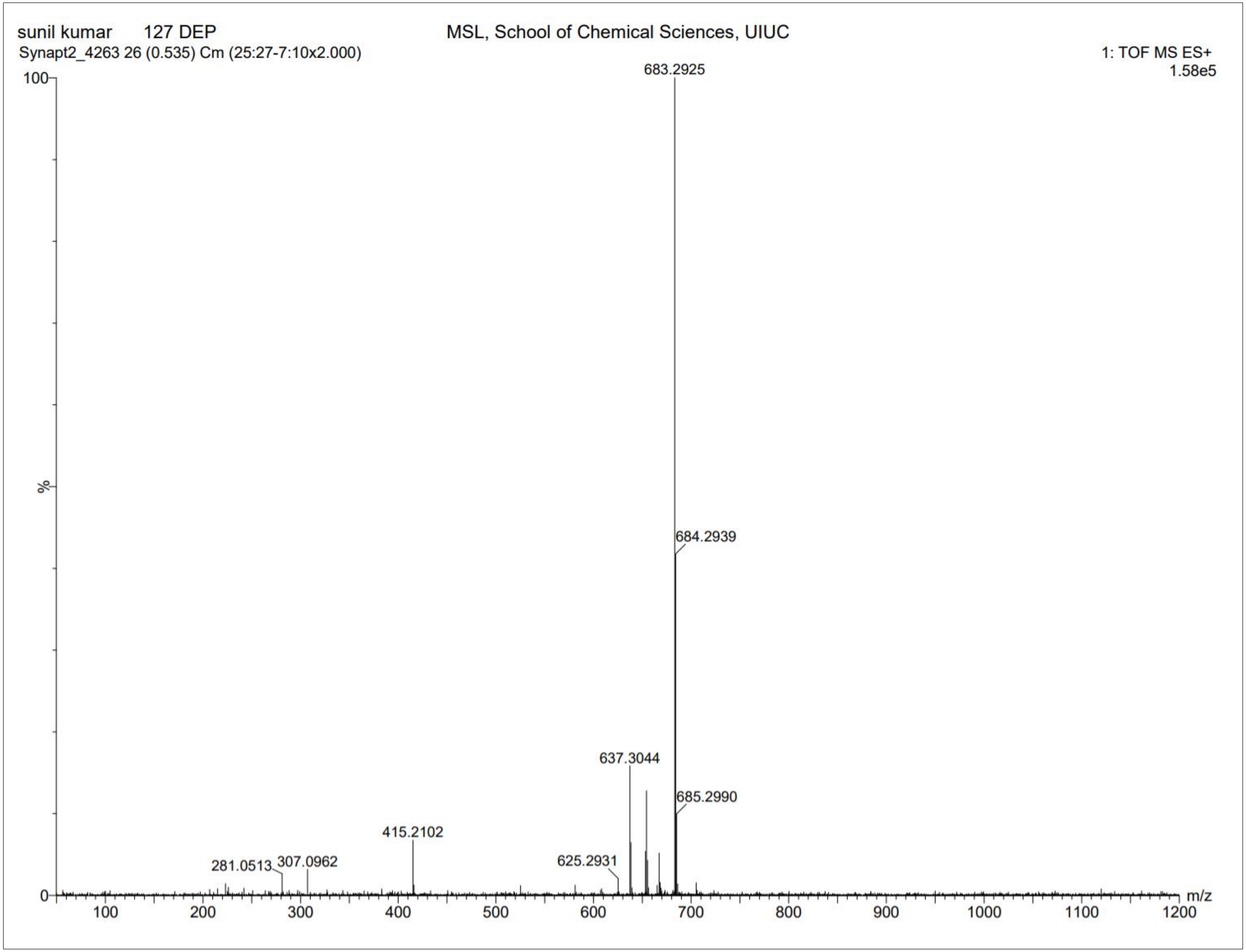

Supplementary Fig. 114. ^{1}H-NMR of NS129 Pro

^{1}H NMR (500 MHz, DMSO) δ 0.90 – 1.17 (dd, *J* = 81.8, 10.1 Hz, 5H), 1.29 – 1.49 (s, 9H), 1.50 – 1.87 (m, 6H), 2.30 – 2.37 (s, 3H), 3.27 – 3.40 (d, *J* = 6.7 Hz, 2H), 4.83 – 4.88 (s, 2H), 4.88 – 4.99 (s, 2H), 6.28 – 6.36 (s, 1H), 6.91 – 6.97 (d, *J* = 7.9 Hz, 1H), 7.38 – 7.49 (t, *J* = 7.5 Hz, 2H), 7.66 – 7.73 (s, 1H), 8.04 – 8.17 (d, *J* = 7.7 Hz, 1H), 8.59 – 8.68 (m, 2H), 8.96 – 9.04 (s, 1H), 10.17 – 10.29 (s, 1H), 10.31 – 10.45 (s, 1H). HRMS (m/z): [M]+ calcd. for $C_{35}H_{42}N_{10}O_{7}$, 715.3311; found, 715.3275.

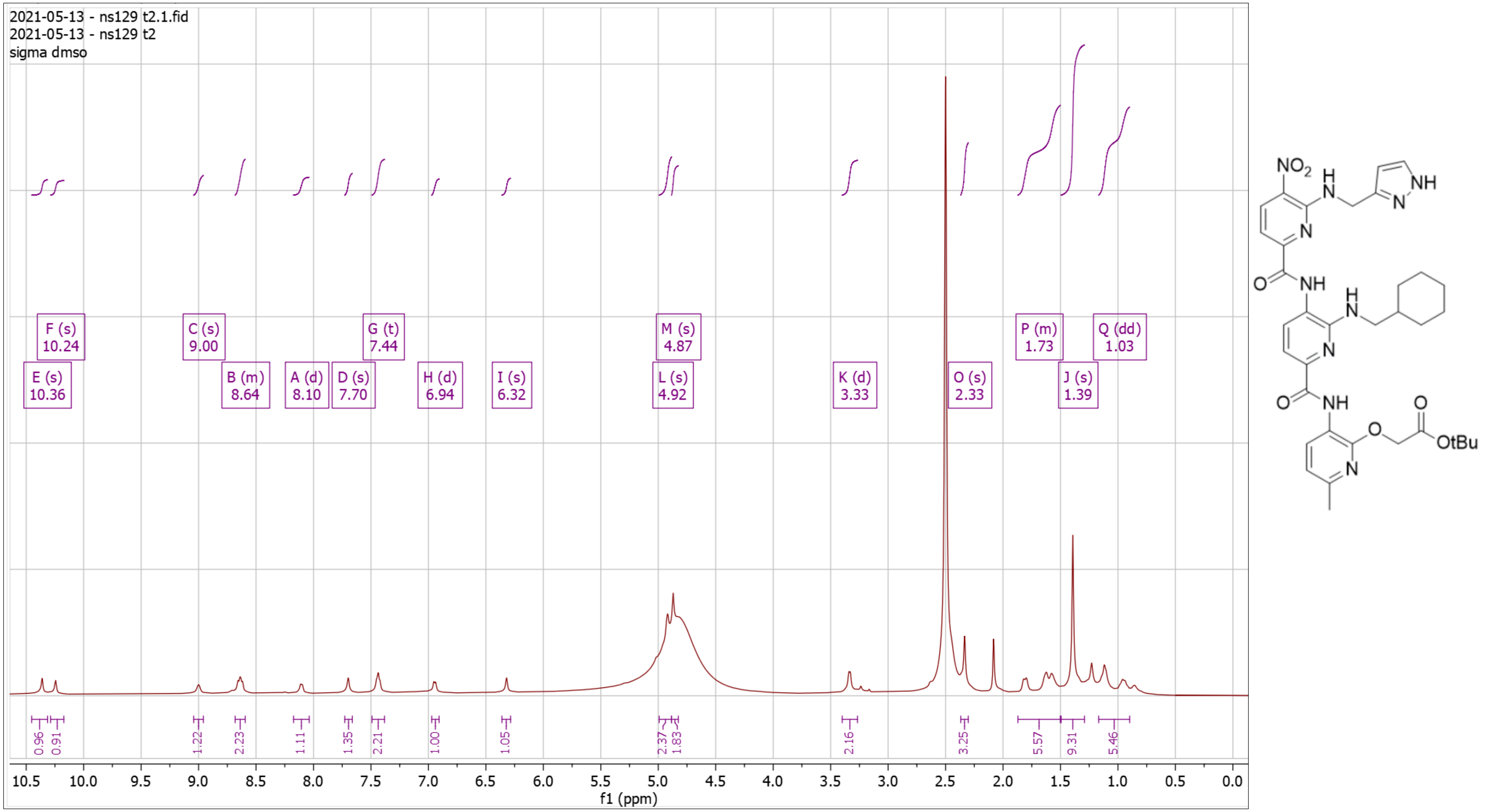

Supplementary Fig. 115. High Resolution Mass Spectrum for NS129 Pro

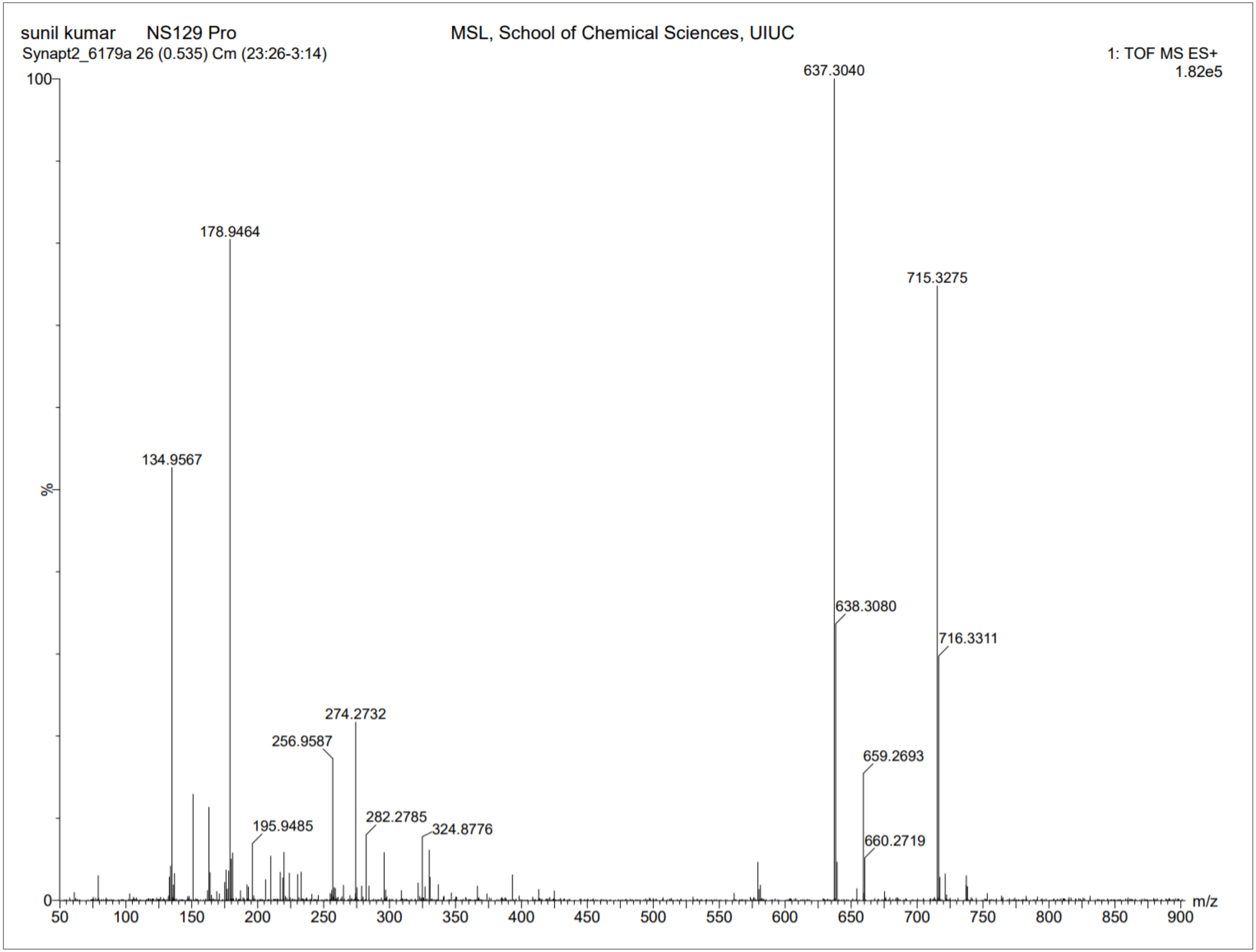

Supplementary Fig. 116. ^{1}H-NMR of NS129 Dep

^{1}H NMR (500 MHz, DMSO) δ 0.78 – 1.32 (m, 5H), 1.50 – 1.95 (m, 6H), 2.33 – 2.37 (s, 3H), 3.31 – 3.36 (d, *J* = 6.1 Hz, 2H), 4.87 – 4.92 (d, *J* = 5.1 Hz, 2H), 4.95 – 4.98 (s, 2H), 6.26 – 6.30 (s, 1H), 6.92 – 6.97 (d, *J* = 8.0 Hz, 1H), 7.10 – 7.14 (s, 1H), 7.16 – 7.22 (d, *J* = 10.3 Hz, 1H), 7.40 – 7.48 (dd, *J* = 13.0, 8.0 Hz, 2H), 7.62 – 7.66 (s, 1H), 8.13 – 8.18 (d, *J* = 8.0 Hz, 1H), 8.61 – 8.68 (t, *J* = 8.2 Hz, 2H), 8.97 – 9.03 (d, *J* = 5.8 Hz, 1H), 10.27 – 10.31 (s, 1H), 10.38 – 10.41 (s, 1H). HRMS (m/z): [M]+ calcd. for $C_{31}H_{34}N_{10}O_7$, 659.2685; found, 659.2673.

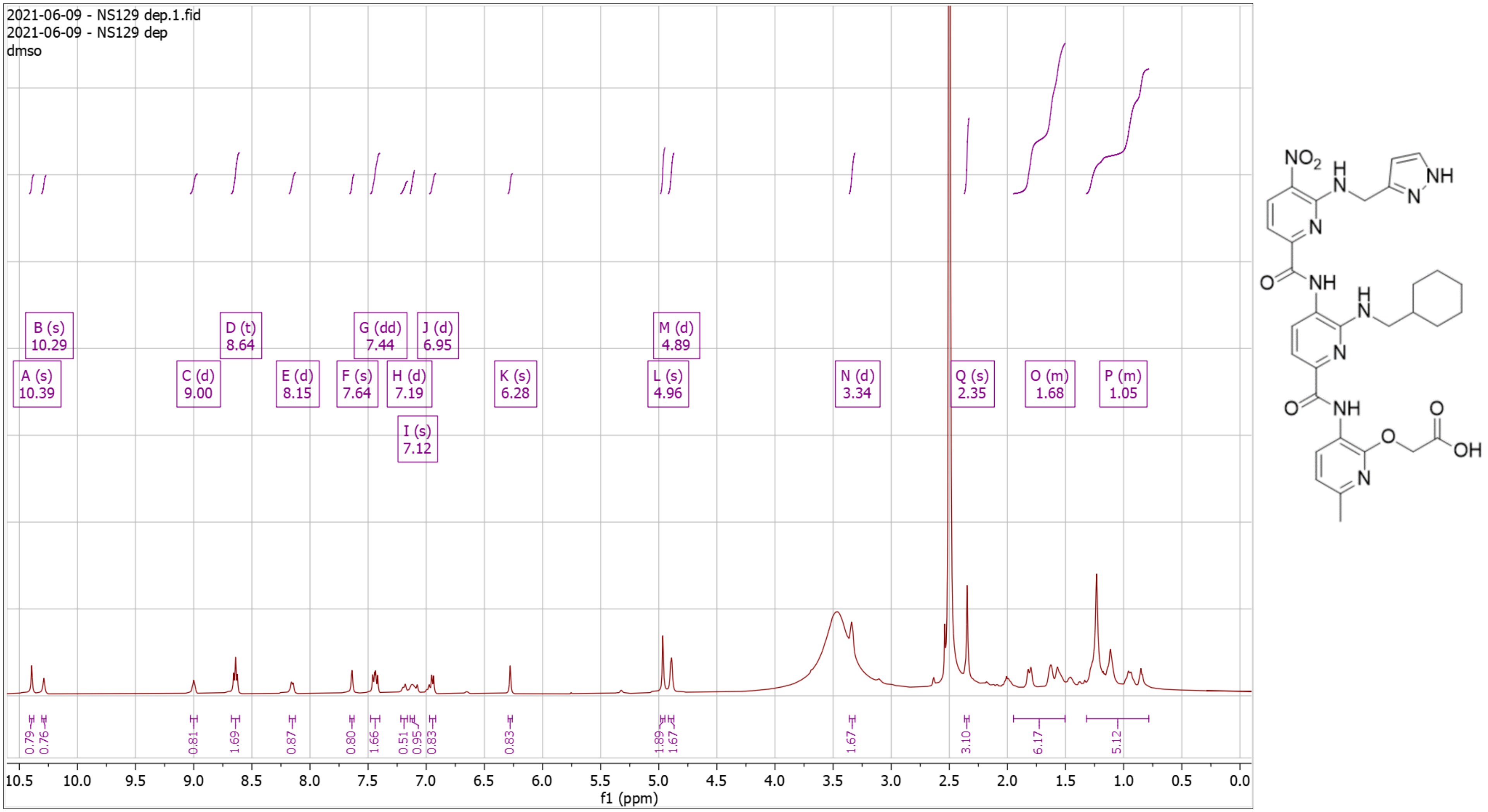

Supplementary Fig. 117. High Resolution Mass Spectrum for NS129 Dep

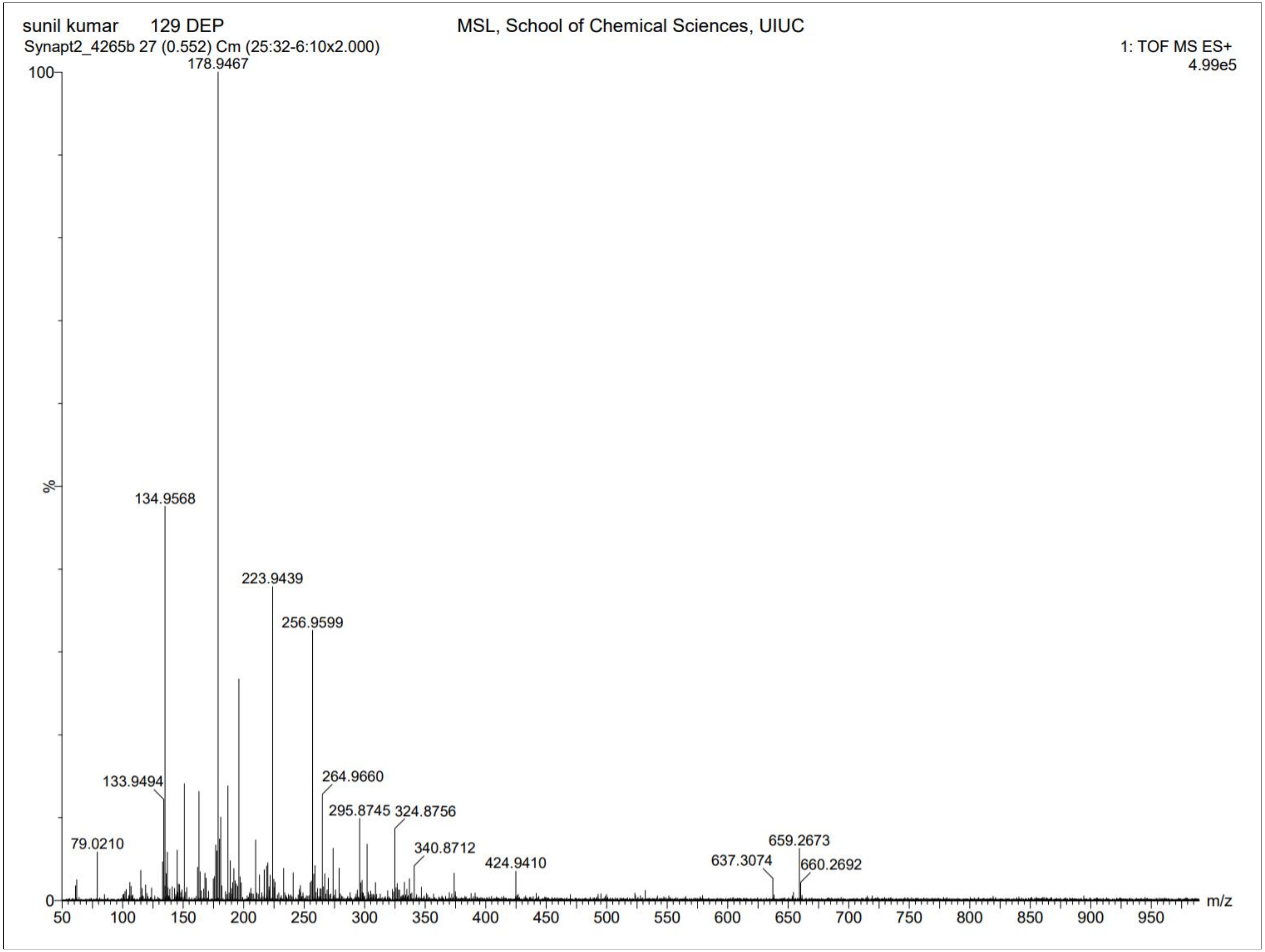

Supplementary Fig. 118. ^{1}H-NMR of NS130 Pro

^{1}H NMR (500 MHz, $CDCl_3$) δ 0.76 – 1.21 (m, 5H), 1.24 – 1.33 (s, 9H), 1.33 – 1.39 (s, 9H), 1.56 – 1.91 (m, 6H), 2.30 – 2.33 (s, 3H), 2.54 – 2.60 (t, *J* = 6.1 Hz, 2H), 3.38 – 3.43 (d, *J* = 6.8 Hz, 2H), 3.97 – 4.04 (q, *J* = 6.2 Hz, 2H), 4.77 – 4.82 (s, 2H), 6.72 – 6.77 (d, *J* = 8.0 Hz, 1H), 7.54 – 7.60 (dd, *J* = 8.1, 3.4 Hz, 2H), 7.98 – 8.04 (d, *J* = 7.9 Hz, 1H), 8.55 – 8.60 (d, *J* = 8.4 Hz, 1H), 8.65 – 8.70 (d, *J* = 7.9 Hz, 1H), 9.52 – 9.56 (s, 1H), 10.31 – 10.34 (s, 1H). HRMS (m/z): [M]+ calcd. for $C_{38}H_{50}N_8O_9$, 763.3774; found, 763.3752.

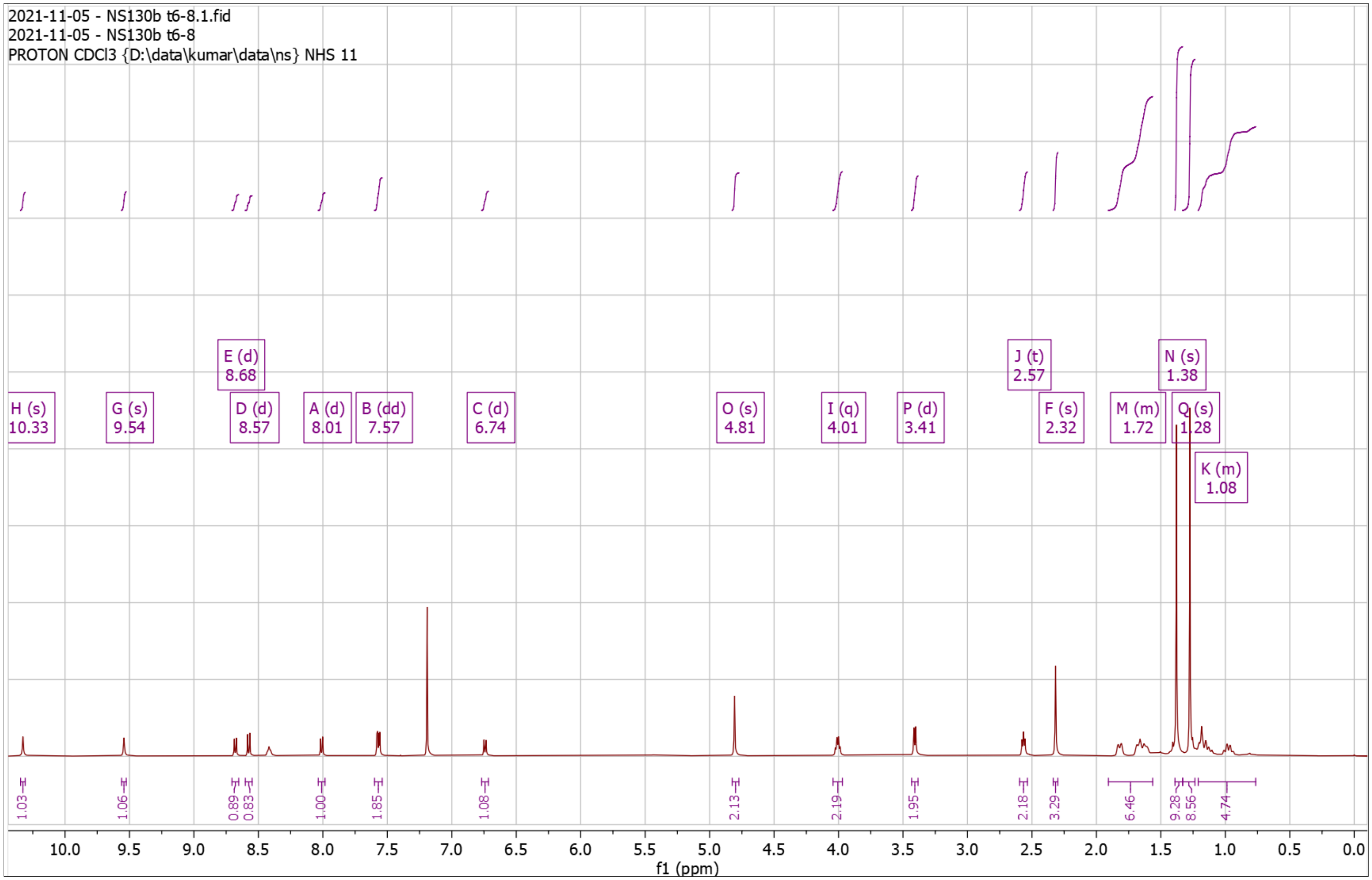

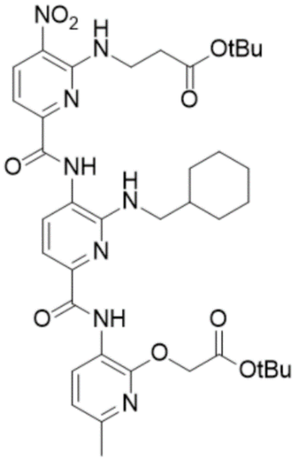

Supplementary Fig. 119. High Resolution Mass Spectrum for NS130 Pro

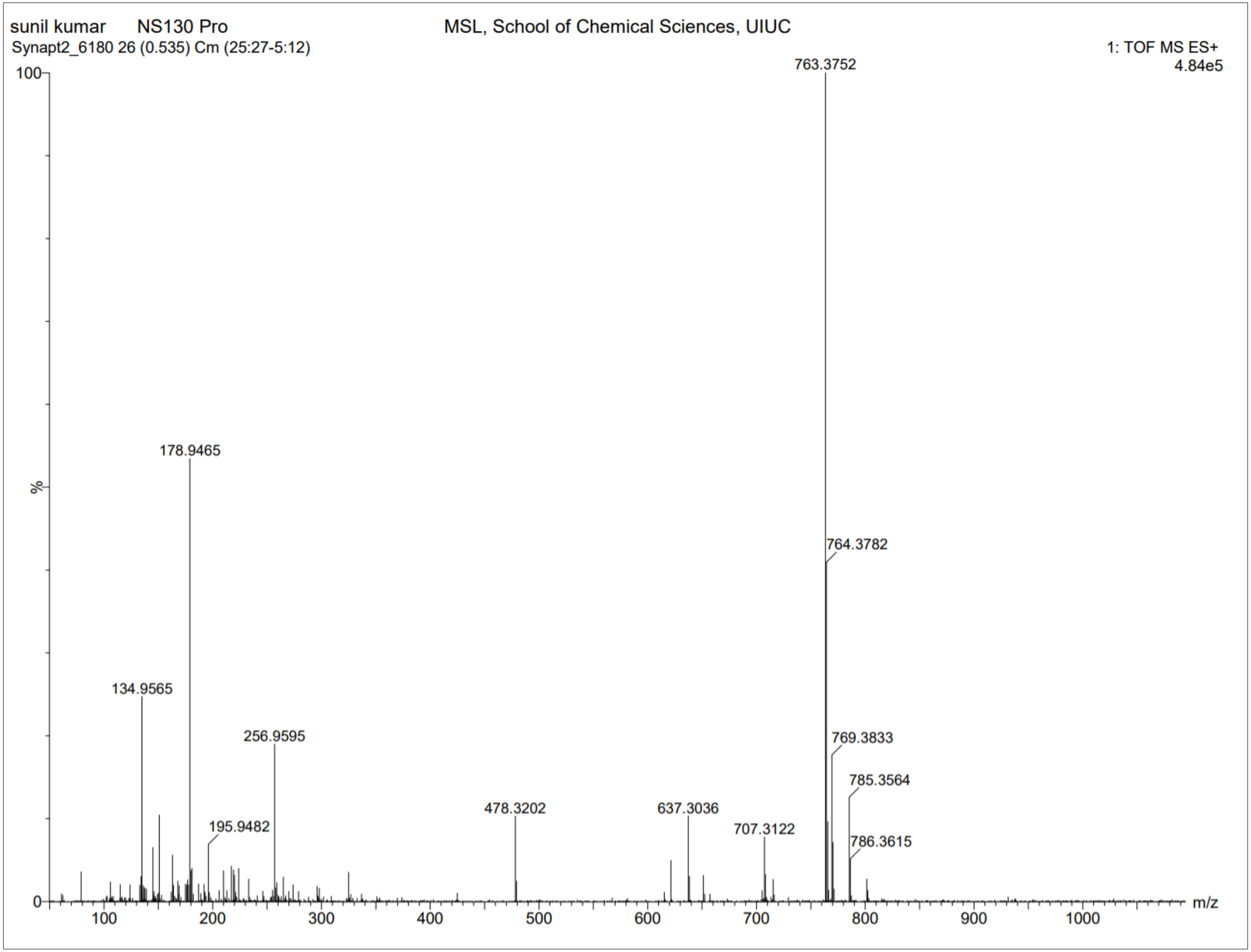

Supplementary Fig. 120. ^{1}H-NMR of NS130 Dep

^{1}H NMR (500 MHz, DMSO) δ 0.90 – 1.27 (dt, J = 96.8, 13.0 Hz, 5H), 1.56 – 1.86 (dd, J = 92.9, 19.4 Hz, 6H), 2.34 – 2.35 (s, 3H), 2.61 – 2.67 (t, J = 6.4 Hz, 2H), 3.98 – 4.06 (q, J = 6.3 Hz, 2H), 4.94 – 4.98 (s, 2H), 6.69 – 6.75 (t, J = 5.8 Hz, 1H), 6.92 – 6.97 (d, J = 8.0 Hz, 1H), 7.38 – 7.44 (d, J = 8.1 Hz, 2H), 7.84 – 7.89 (d, J = 7.7 Hz, 1H), 8.61 – 8.67 (dd, J = 8.2, 4.9 Hz, 2H), 8.68 – 8.74 (t, J = 5.9 Hz, 1H), 9.91 – 9.95 (s, 1H), 10.35 – 10.38 (s, 1H). HRMS (m/z): [M]+ calcd. for $C_{30}H_{34}N_8O_9$, 651.2522; found, 651.2520.

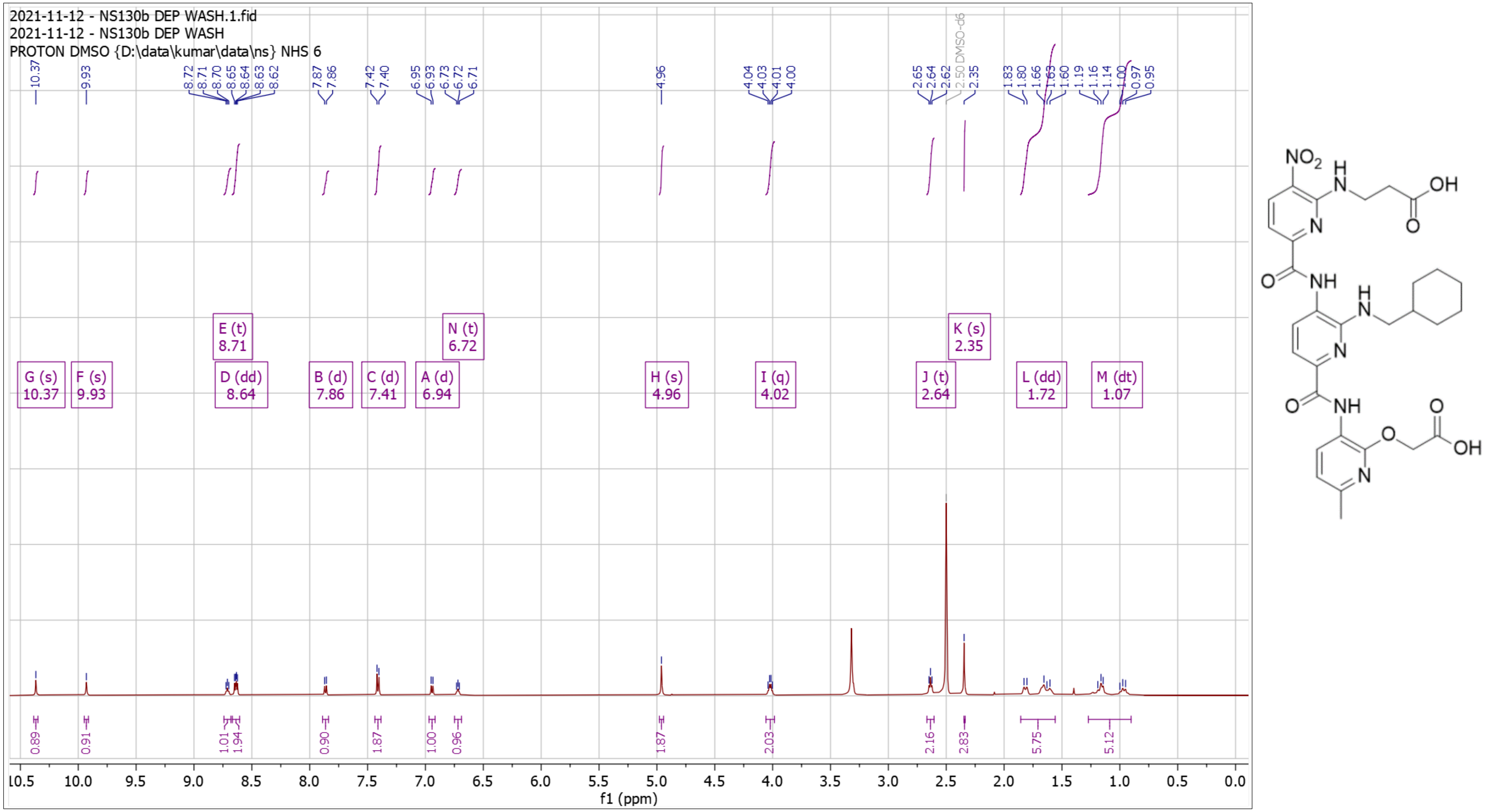

Supplementary Fig. 121. High Resolution Mass Spectrum for NS130 Dep

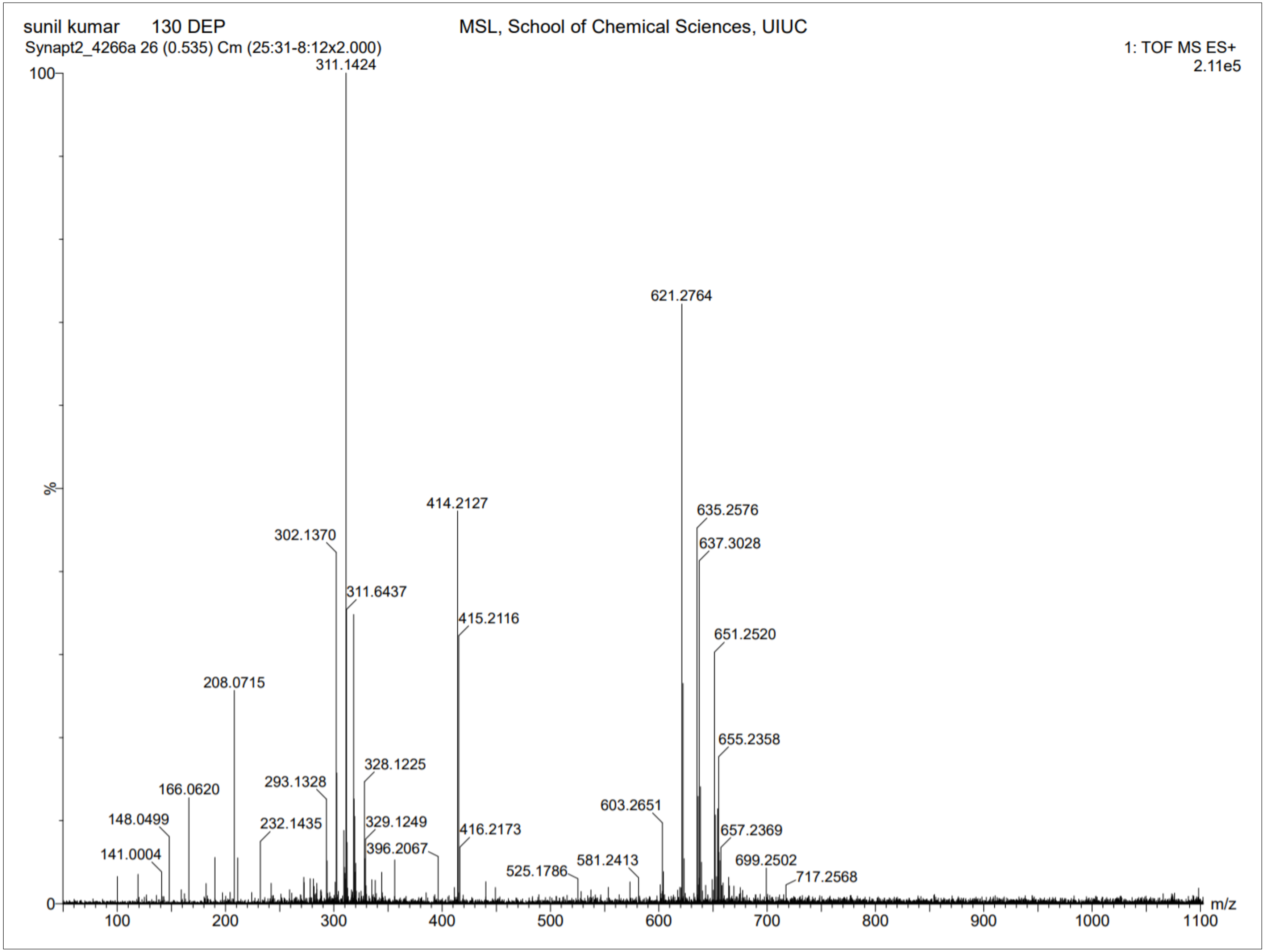

Supplementary Fig. 122. ^{1}H-NMR of NS131 Pro

^{1}H NMR (500 MHz, DMSO) δ 0.91 – 1.30 (m, 5H), 1.39 – 1.40 (s, 9H), 1.60 – 1.87 (m, 6H), 2.32 – 2.35 (s, 3H), 2.75 – 2.84 (t, *J* = 6.2 Hz, 2H), 3.97 – 4.13 (q, *J* = 6.1 Hz, 2H), 4.80 – 4.97 (s, 2H), 6.72 – 6.84 (t, *J* = 5.4 Hz, 1H), 6.91 – 6.98 (d, *J* = 7.9 Hz, 1H), 7.35 – 7.45 (dd, *J* = 8.1, 4.4 Hz, 2H), 7.85 – 7.94 (d, *J* = 7.6 Hz, 1H), 8.57 – 8.67 (dd, *J* = 8.2, 4.0 Hz, 2H), 9.05 – 9.14 (t, *J* = 5.5 Hz, 1H), 10.28 – 10.34 (s, 1H), 10.34 – 10.42 (s, 1H). HRMS (m/z): [M]+ calcd. for $C_{33}H_{42}N_8O_{10}S$, 743.2817; found, 743.2793.

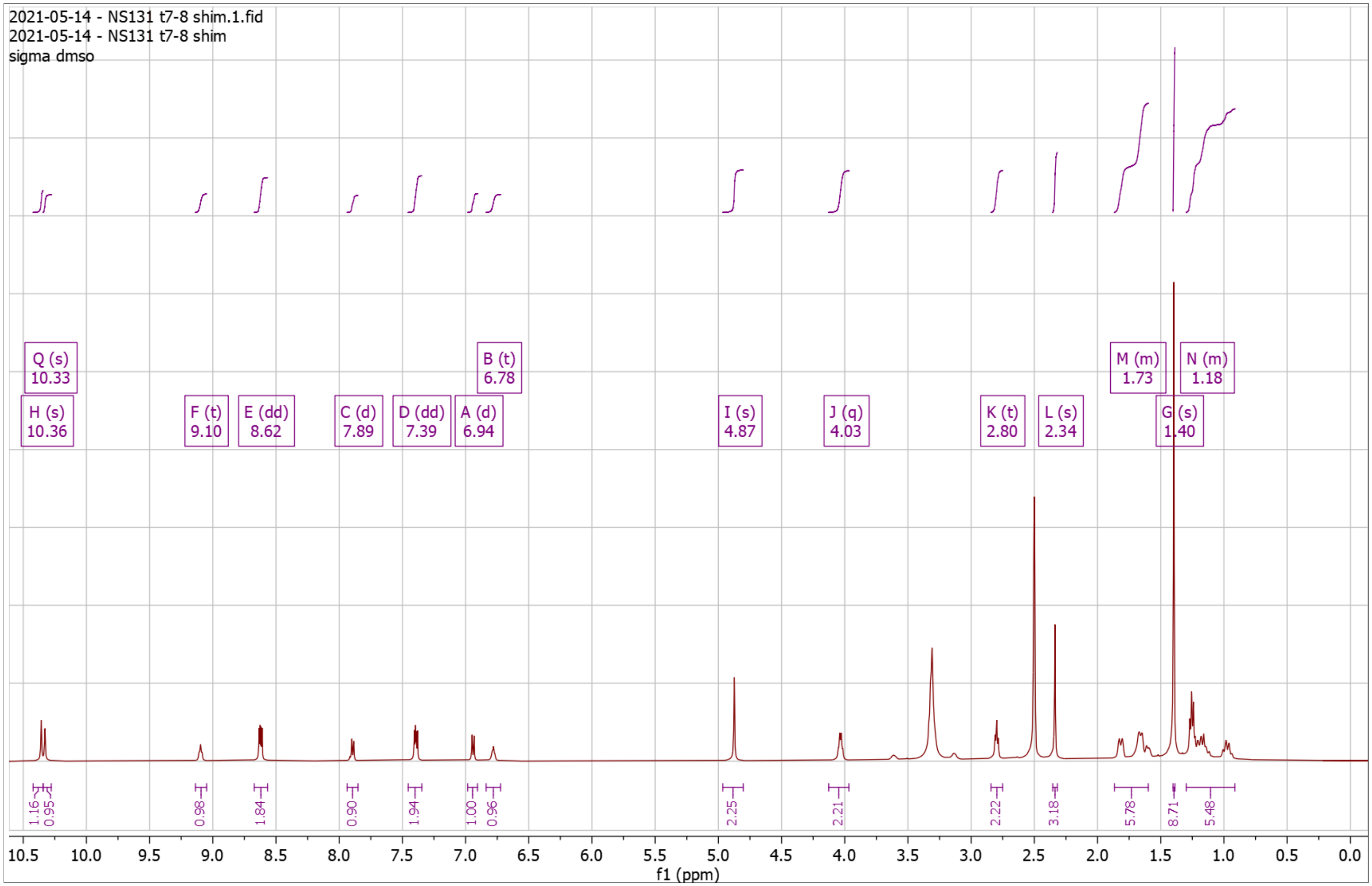

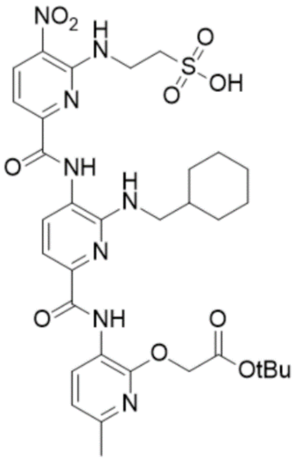

Supplementary Fig. 123. High Resolution Mass Spectrum for NS131 Pro

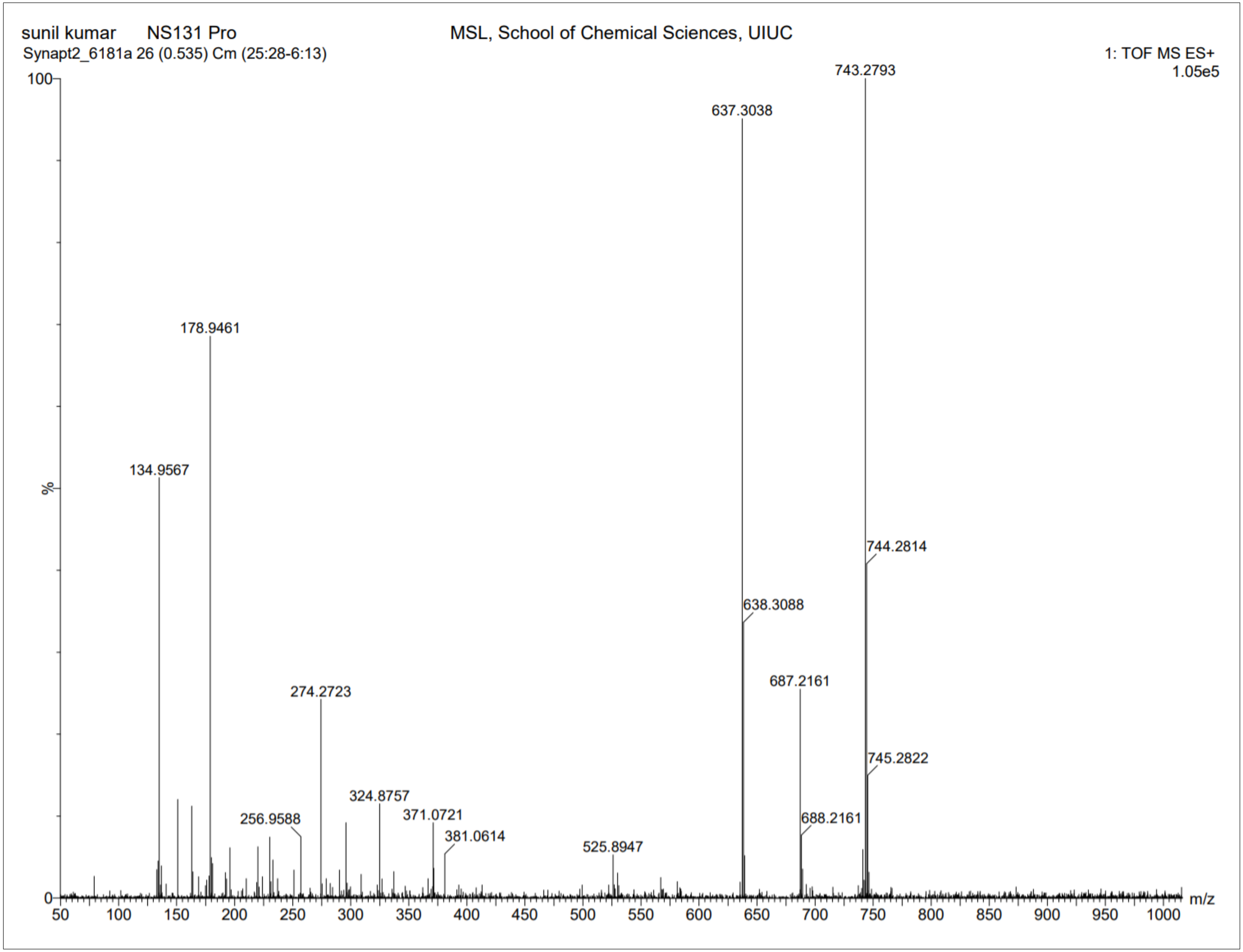

Supplementary Fig. 124. ^{1}H-NMR of NS131 Dep

^{1}H NMR (500 MHz, DMSO) δ 0.84 – 1.19 (m, 5H), 1.53 – 1.78 (m, 6H), 2.25 – 2.29 (s, 3H), 2.69 – 2.75 (t, J = 6.3 Hz, 2H), 3.92 – 4.00 (q, J = 6.1 Hz, 2H), 4.86 – 4.90 (s, 2H), 6.67 – 6.74 (t, J = 5.6 Hz, 1H), 6.84 – 6.90 (d, J = 7.9 Hz, 1H), 7.29 – 7.35 (dd, J = 8.1, 4.3 Hz, 2H), 7.80 – 7.85 (d, J = 7.7 Hz, 1H), 8.53 – 8.61 (dd, J = 8.1, 3.2 Hz, 2H), 9.00 – 9.06 (t, J = 5.6 Hz, 1H), 10.25 – 10.28 (s, 1H), 10.31 – 10.35 (s, 1H). HRMS (m/z): [M]+ calcd. for $C_{29}H_{34}N_8O_{10}S$, 687.2191; found, 687.2180.

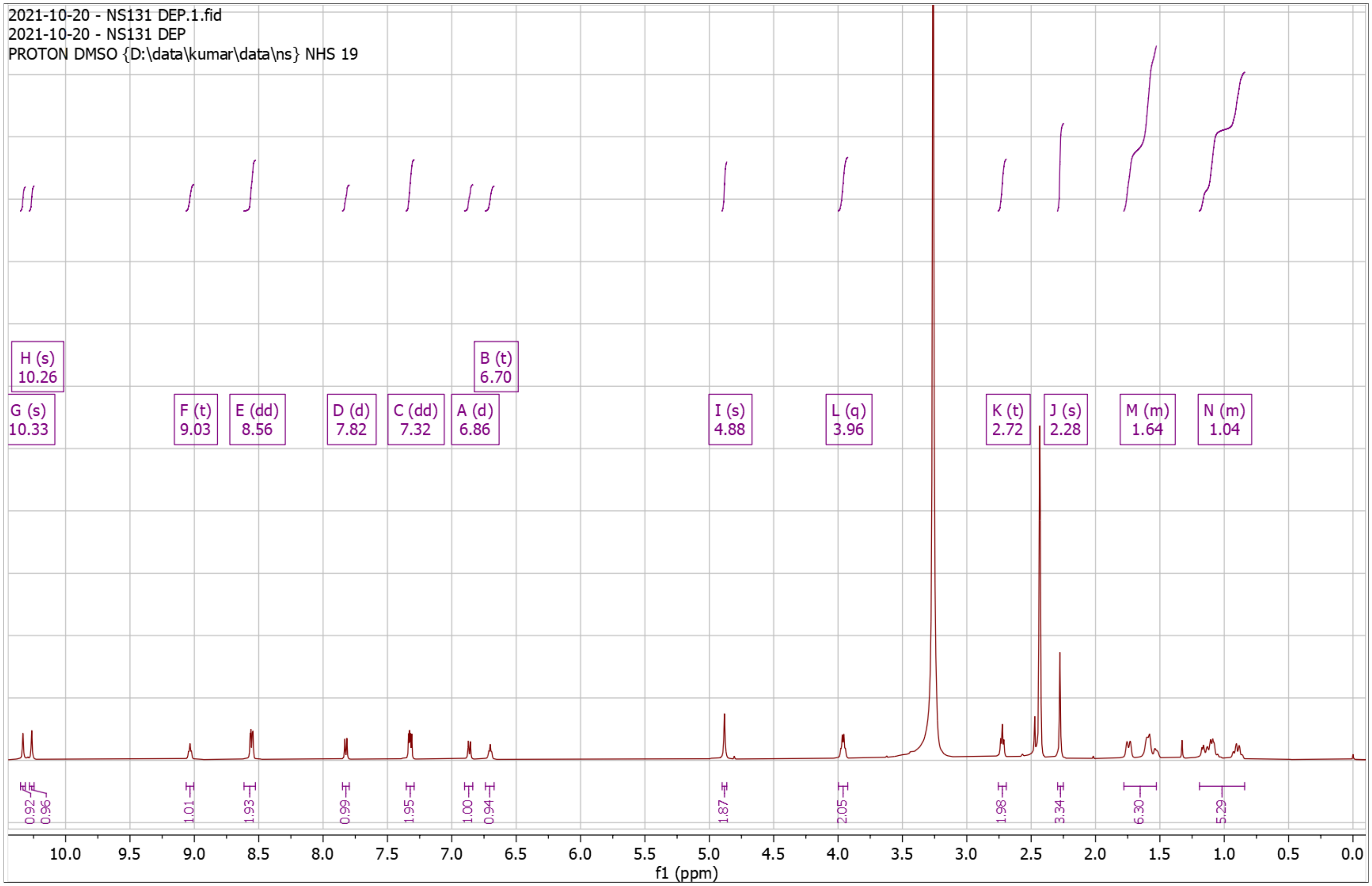

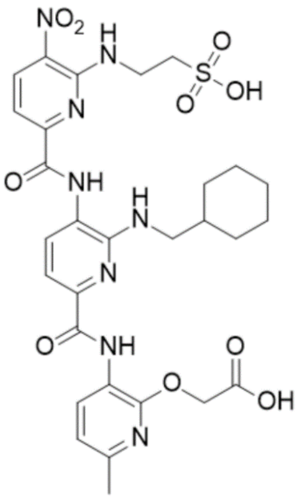

Supplementary Fig. 125. High Resolution Mass Spectrum for NS131 Dep

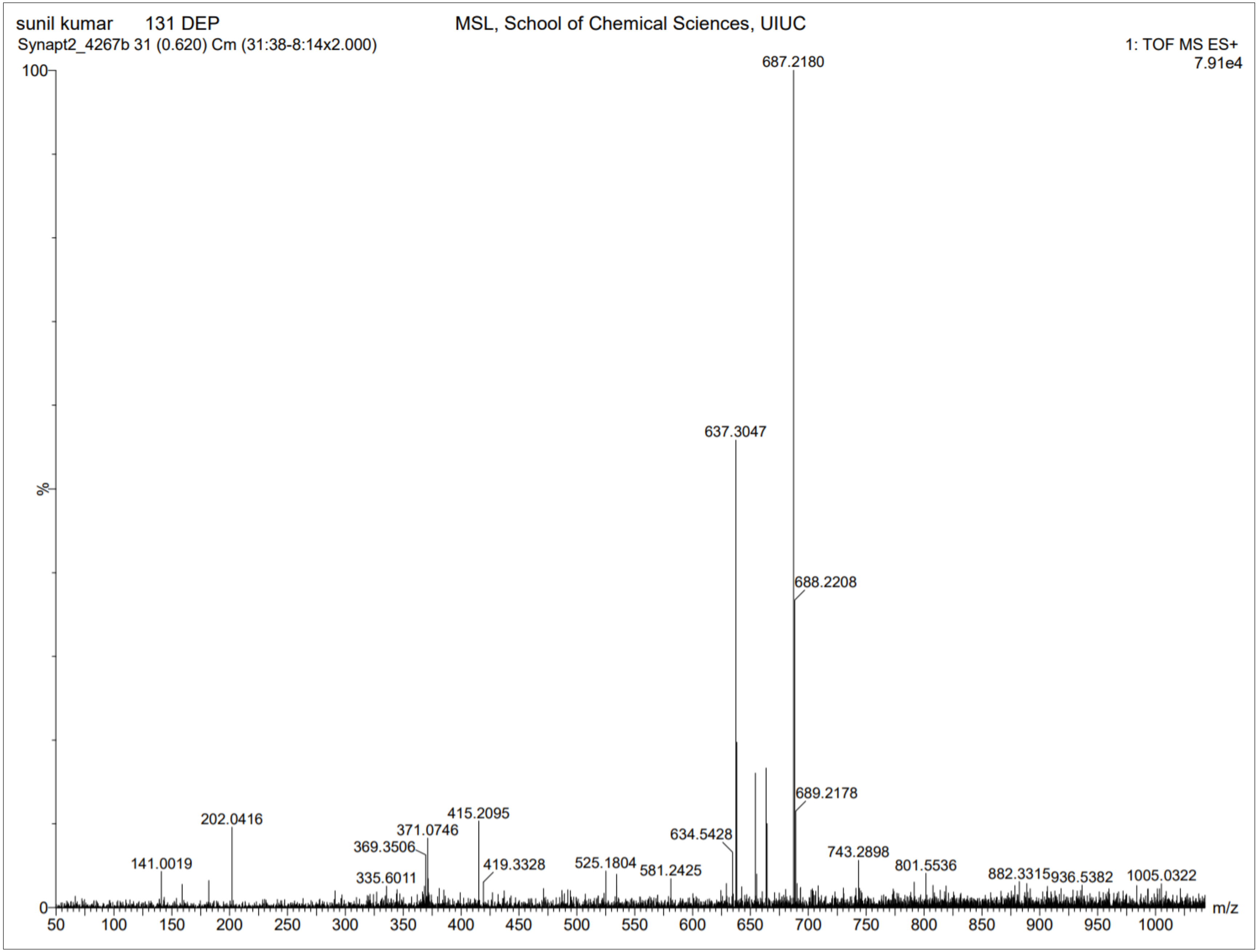

Supplementary Fig. 126. ^{1}H-NMR of NS132 Pro

^{1}H NMR (500 MHz, DMSO) δ 0.81 – 1.18 (dt, *J* = 94.1, 11.7 Hz, 5H), 1.36 – 1.42 (s, 9H), 1.50 – 1.80 (m, 6H), 2.33 – 2.35 (s, 3H), 3.06 – 3.13 (t, *J* = 7.1 Hz, 2H), 3.23 – 3.29 (t, *J* = 6.1 Hz, 2H), 3.98 – 4.13 (q, *J* = 6.8 Hz, 2H), 4.83 – 4.90 (s, 2H), 6.71 – 6.76 (t, *J* = 5.6 Hz, 1H), 6.76 – 6.82 (t, *J* = 7.5 Hz, 1H), 6.91 – 6.97 (d, *J* = 7.9 Hz, 1H), 6.97 – 7.03 (t, *J* = 7.5 Hz, 1H), 7.19 – 7.23 (d, *J* = 2.3 Hz, 1H), 7.27 – 7.31 (d, *J* = 8.1 Hz, 1H), 7.34 – 7.39 (d, *J* = 8.4 Hz, 1H), 7.41 – 7.47 (d, *J* = 7.7 Hz, 1H), 7.51 – 7.58 (d, *J* = 7.9 Hz, 1H), 7.92 – 7.97 (d, *J* = 7.9 Hz, 1H), 8.55 – 8.68 (m, 3H), 9.81 – 9.87 (s, 1H), 10.31 – 10.38 (s, 1H), 10.79 – 10.85 (m, 1H). HRMS (m/z): [M]+ calcd. for $C_{41}H_{47}N_9O_7$, 778.3671; found, 778.3665.

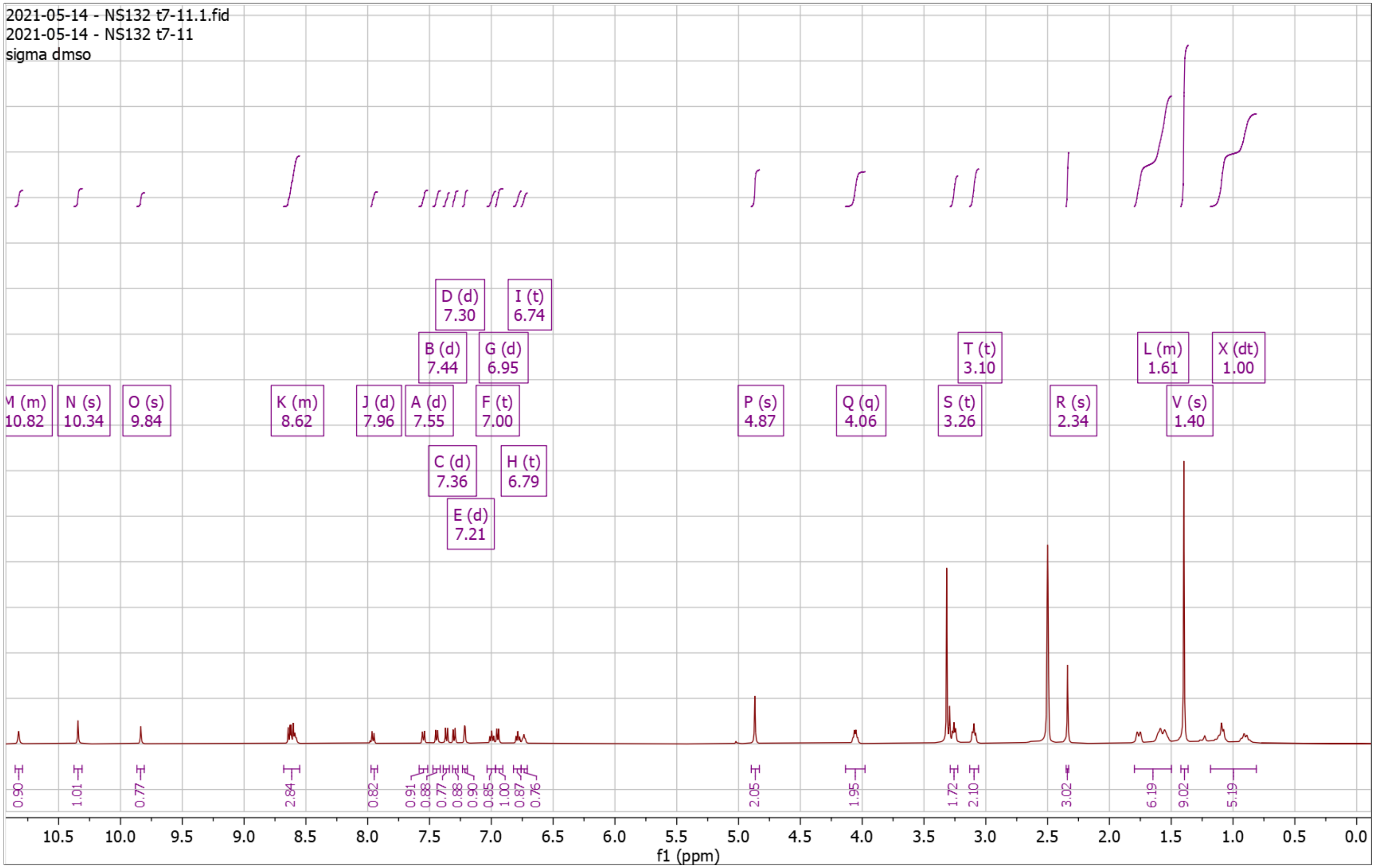

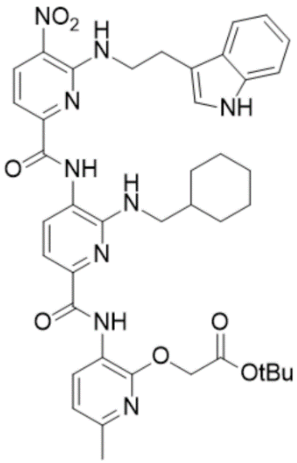

Supplementary Fig. 127. High Resolution Mass Spectrum for NS132 Pro

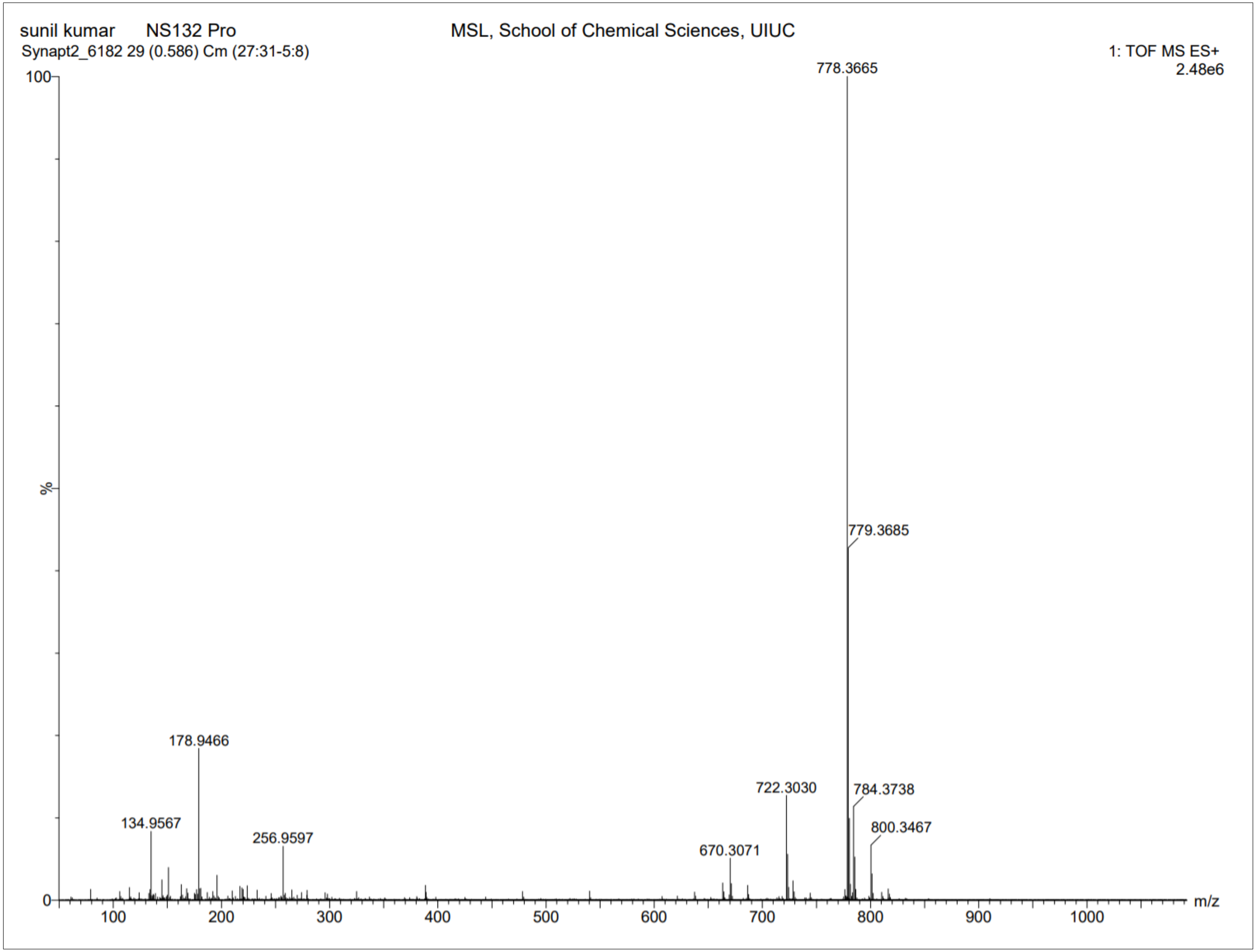

Supplementary Fig. 128. ^{1}H-NMR of NS132 Dep

^{1}H NMR (500 MHz, DMSO) δ 0.84 – 1.13 (m, 5H), 1.49 – 1.83 (m, 6H), 2.33 – 2.36 (s, 3H), 3.06 – 3.16 (t, *J* = 7.1 Hz, 2H), 3.22 – 3.30 (t, *J* = 6.2 Hz, 2H), 3.99 – 4.14 (q, *J* = 6.7 Hz, 2H), 4.94 – 4.98 (s, 2H), 6.69 – 6.77 (t, *J* = 5.7 Hz, 1H), 6.77 – 6.83 (t, *J* = 7.4 Hz, 1H), 6.91 – 6.96 (d, *J* = 7.9 Hz, 1H), 6.97 – 7.03 (t, *J* = 7.6 Hz, 1H), 7.20 – 7.24 (d, *J* = 2.3 Hz, 1H), 7.28 – 7.33 (d, *J* = 8.1 Hz, 1H), 7.33 – 7.41 (d, *J* = 8.5 Hz, 1H), 7.41 – 7.47 (d, *J* = 7.7 Hz, 1H), 7.53 – 7.58 (d, *J* = 7.9 Hz, 1H), 7.93 – 7.98 (d, *J* = 7.7 Hz, 1H), 8.56 – 8.66 (td, *J* = 13.7, 12.0, 6.8 Hz, 3H), 9.83 – 9.87 (s, 1H), 10.33 – 10.38 (s, 1H), 10.83 – 10.87 (s, 1H), 12.88 – 13.07 (s, 1H). HRMS (m/z): [M]+ calcd. for $C_{37}H_{39}N_{9}O_{7}$, 722.3045; found, 722.3034.

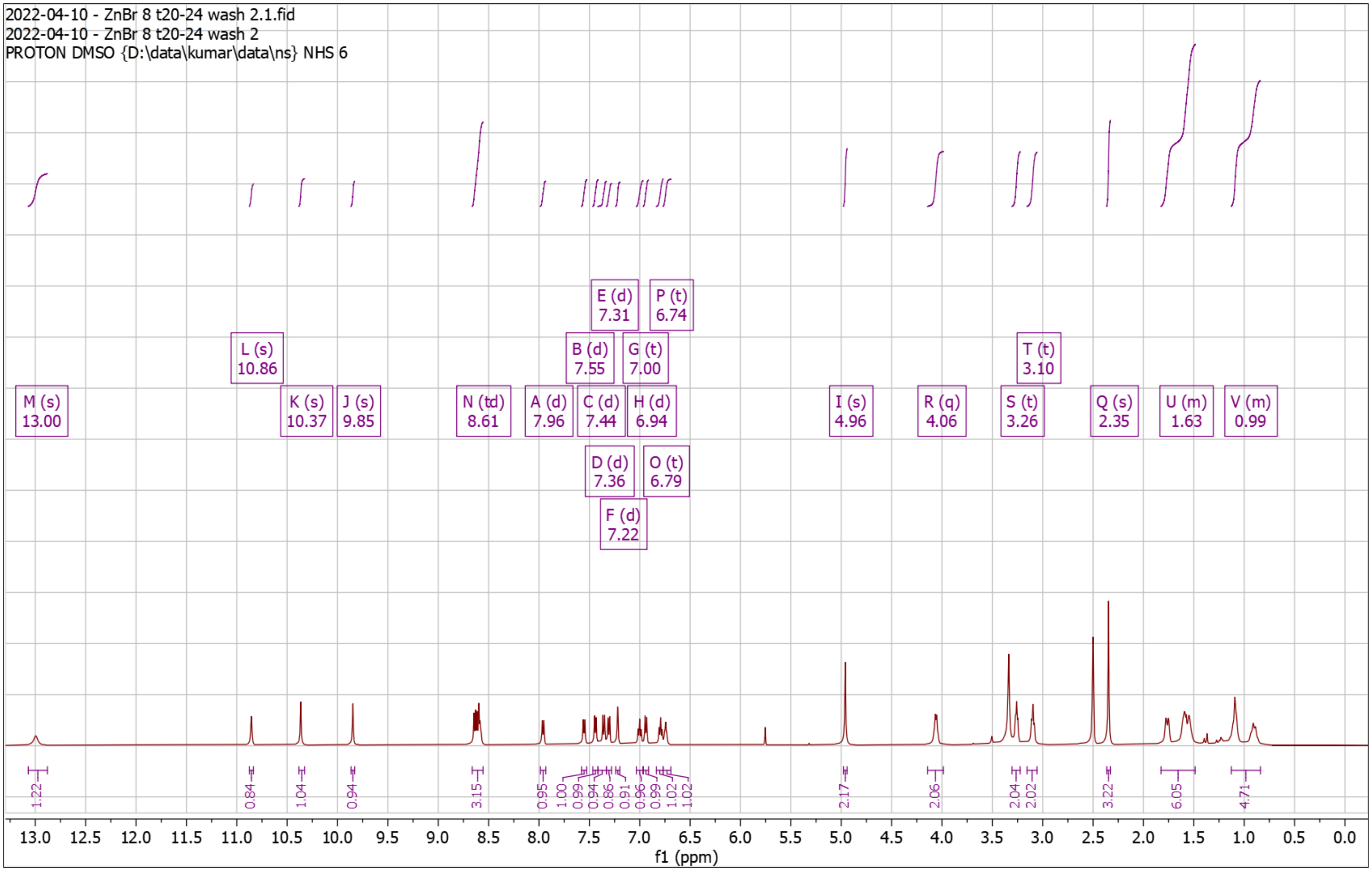

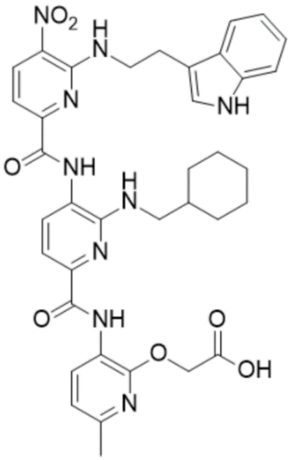

Supplementary Fig. 129. ^{13}C-NMR of NS132 Dep

13C NMR (126 MHz, DMSO) δ 23.25, 24.73, 25.52, 26.06, 30.69, 37.21, 41.66, 47.07, 54.90, 69.78, 109.64, 110.08, 111.38, 111.43, 116.49, 118.10, 118.16, 119.69, 120.91, 122.40, 123.03, 126.35, 127.18, 129.20, 132.17, 136.32, 137.37, 143.33, 148.53, 150.91, 151.67, 153.73, 162.21.

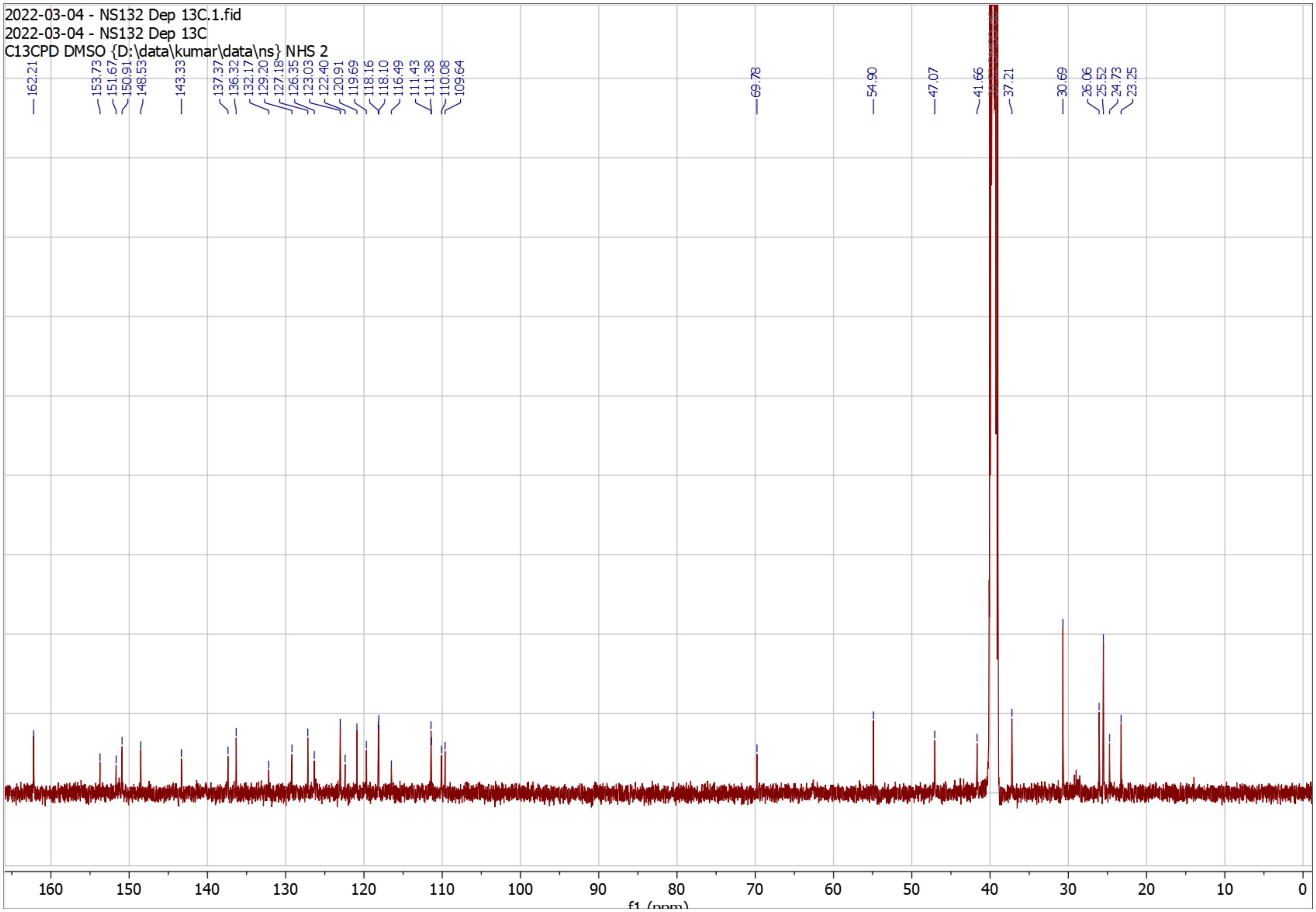

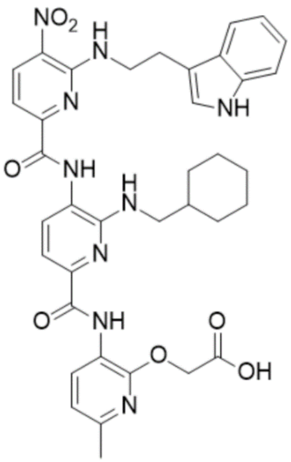

Supplementary Fig. 130. High Resolution Mass Spectrum for NS132 Dep

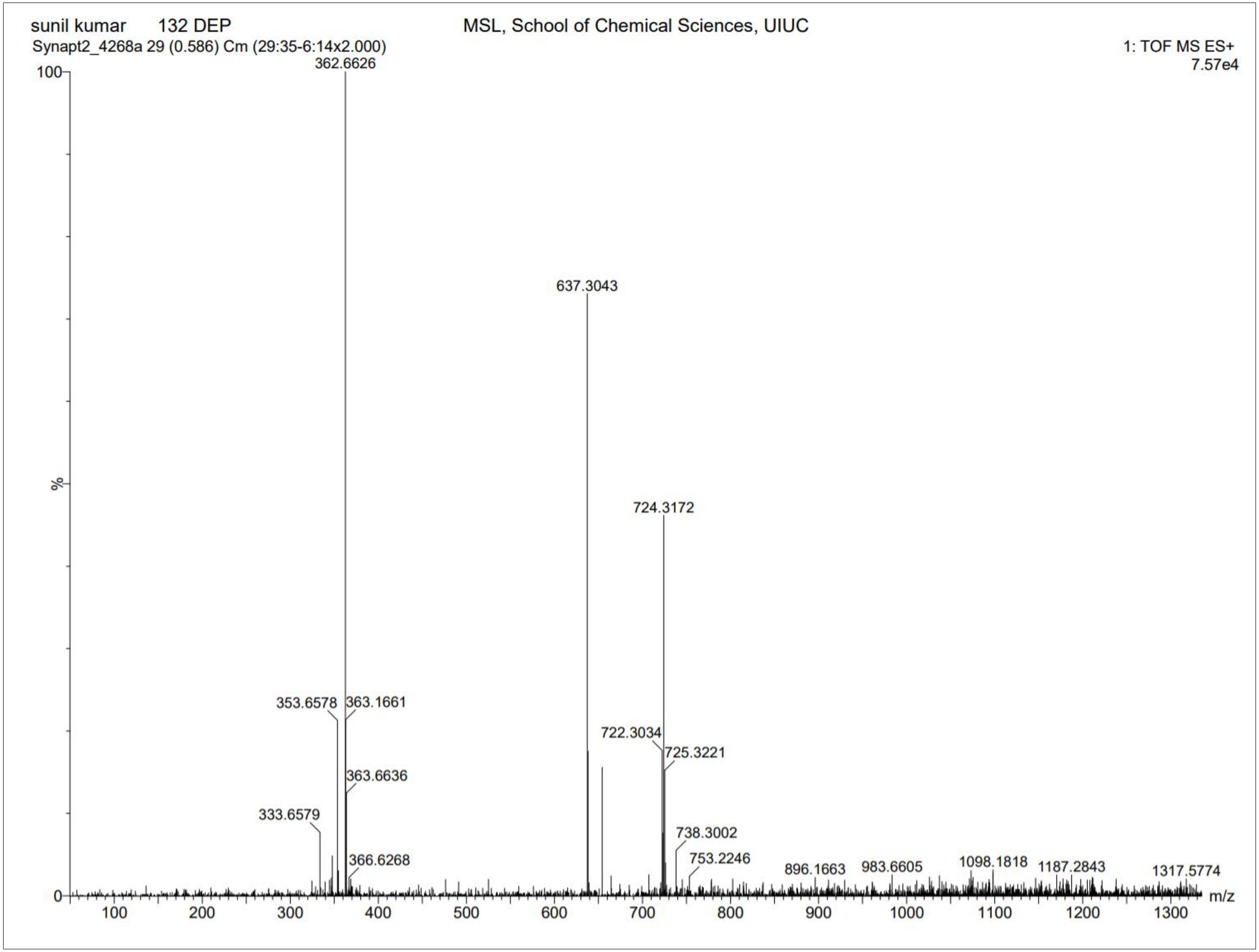

Supplementary Fig. 131. ^{1}H-NMR of NS138 Pro

^{1}H NMR (500 MHz, $CDCl_3$) δ 0.72 – 1.08 (m, 3H), 1.08 – 1.10 (d, *J* = 6.7 Hz, 6H), 1.10 – 1.33 (m, 3H), 1.45 – 1.46 (s, 9H), 1.64 – 1.91 (m, 5H), 2.02 – 2.13 (dt, *J* = 12.9, 6.5 Hz, 1H), 2.36 – 2.39 (s, 3H), 3.44 – 3.50 (t, *J* = 6.2 Hz, 2H), 3.50 – 3.55 (dd, *J* = 6.7, 5.4 Hz, 2H), 4.83 – 4.88 (s, 2H), 6.78 – 6.85 (d, *J* = 7.9 Hz, 1H), 7.58 – 7.65 (d, *J* = 8.4 Hz, 1H), 7.65 – 7.69 (d, *J* = 7.9 Hz, 1H), 7.90 – 7.94 (d, *J* = 7.9 Hz, 1H), 8.34 – 8.41 (s, 1H), 8.63 – 8.68 (d, *J* = 8.4 Hz, 1H), 8.71 – 8.75 (d, *J* = 7.9 Hz, 1H), 9.33 – 9.38 (s, 1H), 10.35 – 10.42 (s, 1H). HRMS (m/z): [M]+ calcd. for $C_{35}H_{46}N_8O_7$, 691.3562; found, 691.3537.

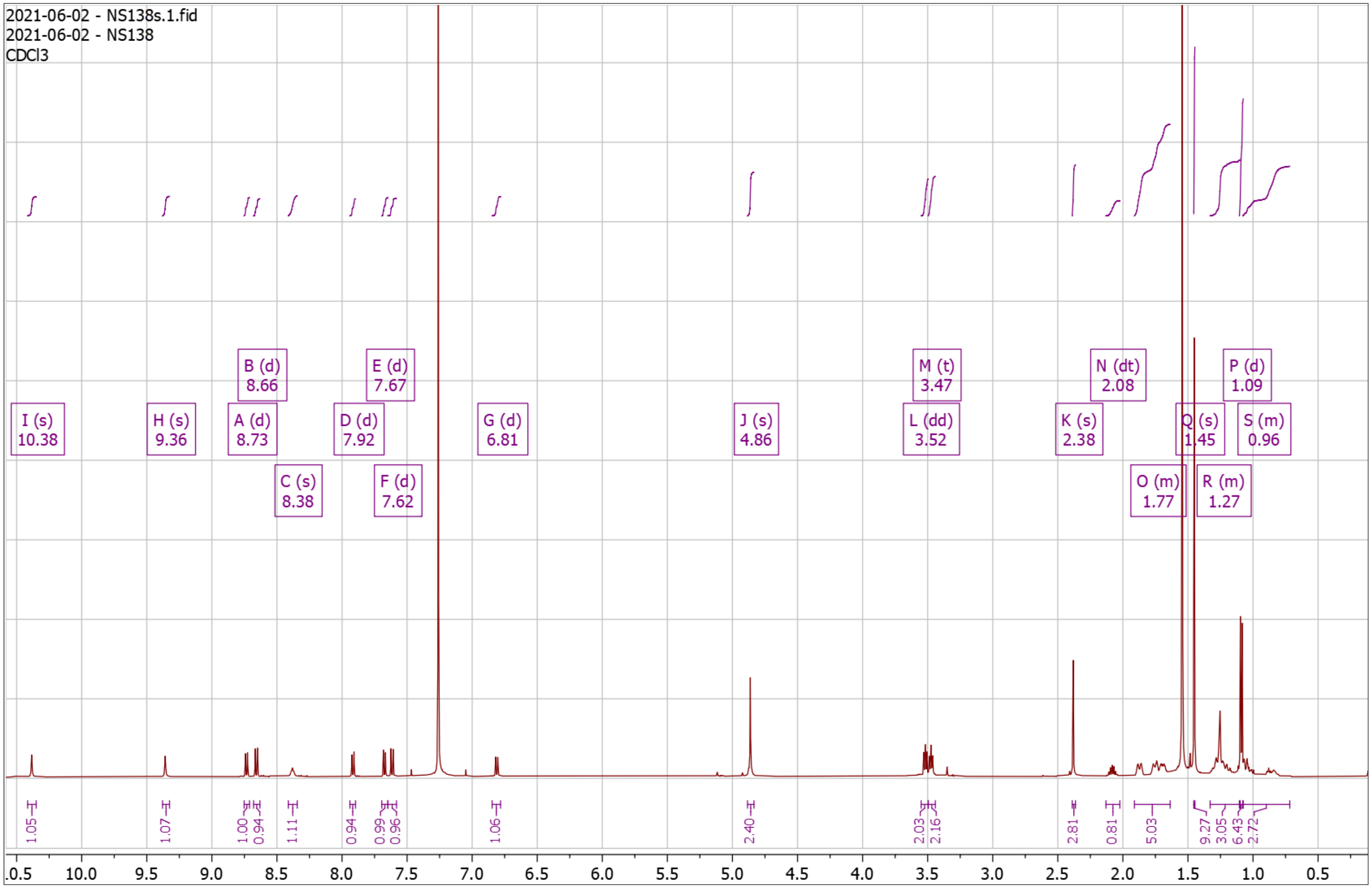

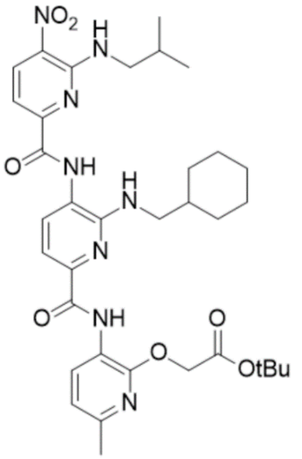

Supplementary Fig. 132. High Resolution Mass Spectrum for NS138 Pro

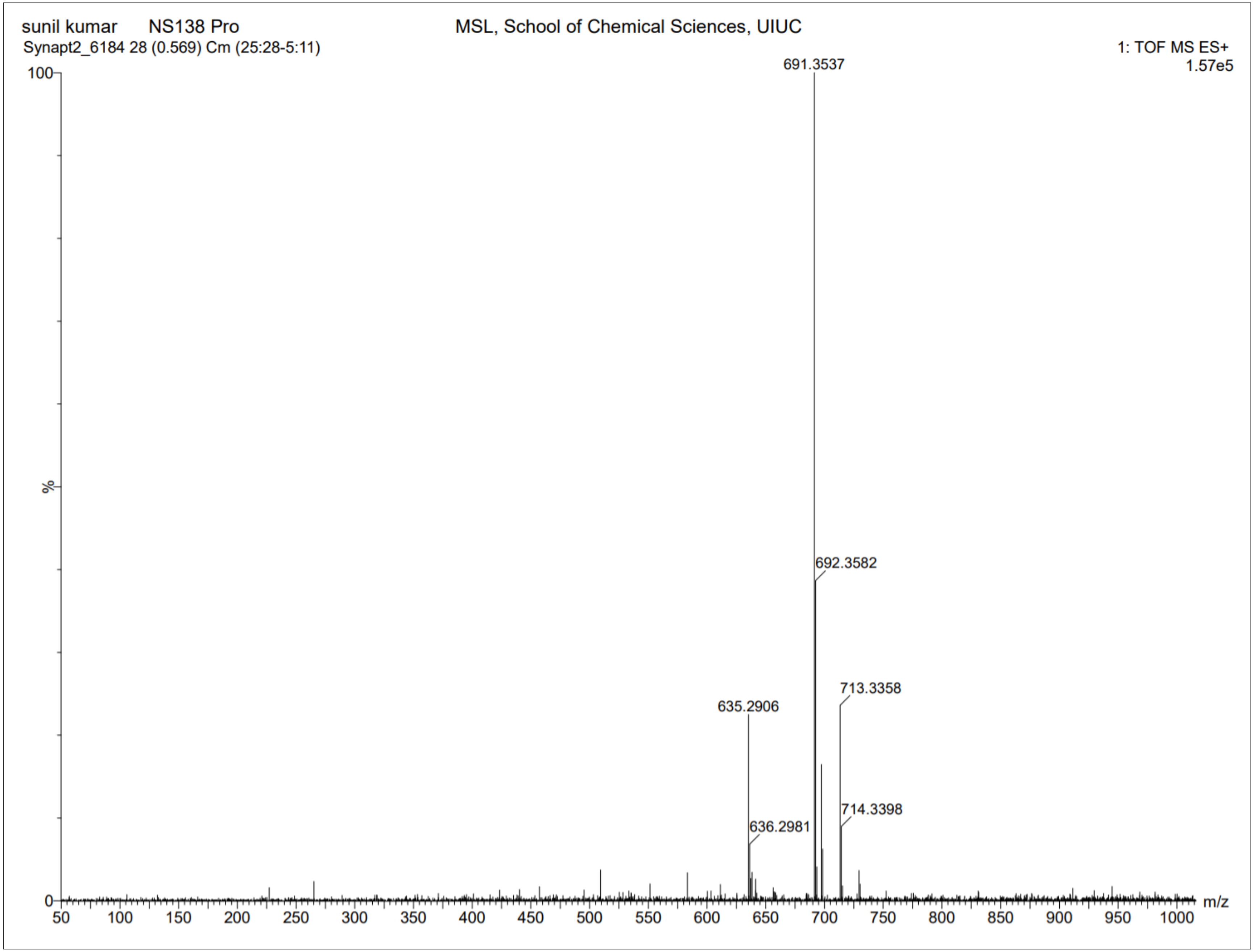

Supplementary Fig. 133. ^{1}H-NMR of NS138 Dep

^{1}H NMR (500 MHz, DMSO) δ 0.94 – 1.00 (d, *J* = 6.6 Hz, 5H), 1.58 – 1.86 (m, 6H), 1.10 – 1.29 (m, 5H), 1.99 – 2.07 (dq, *J* = 14.2, 7.7, 7.2 Hz, 1H), 2.25 – 2.40 (s, 3H), 3.59 – 3.65 (t, *J* = 6.3 Hz, 2H), 4.94 – 4.98 (s, 2H), 6.69 – 6.75 (t, *J* = 5.7 Hz, 1H), 6.91 – 6.96 (d, *J* = 7.9 Hz, 1H), 7.36 – 7.46 (dd, *J* = 22.4, 8.1 Hz, 2H), 7.90 – 7.94 (d, *J* = 7.8 Hz, 1H), 8.49 – 8.56 (t, *J* = 5.9 Hz, 1H), 8.60 – 8.63 (d, *J* = 5.0 Hz, 1H), 8.63 – 8.66 (d, *J* = 5.5 Hz, 1H), 9.77 – 9.80 (s, 1H), 10.32 – 10.37 (s, 1H). HRMS (m/z): [M]+ calcd. for $C_{31}H_{38}N_8O_7$, 635.2936; found, 635.2932.

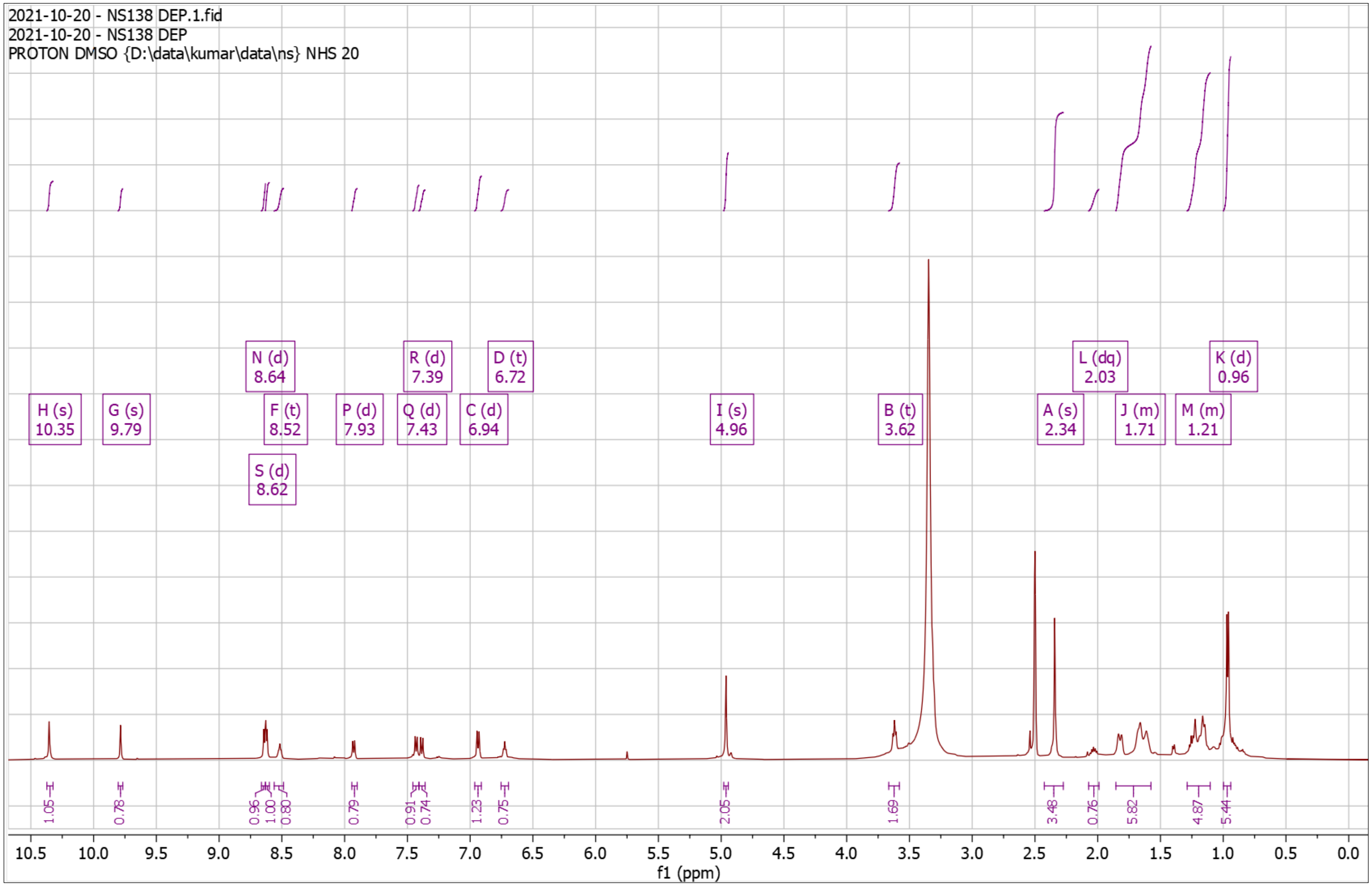

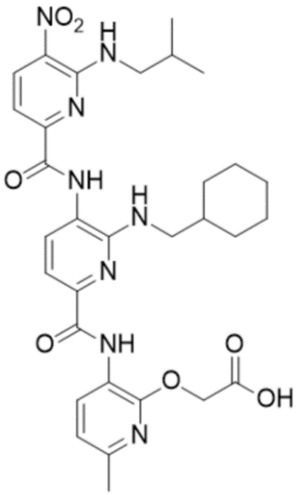

Supplementary Fig. 134. High Resolution Mass Spectrum for NS138 Dep

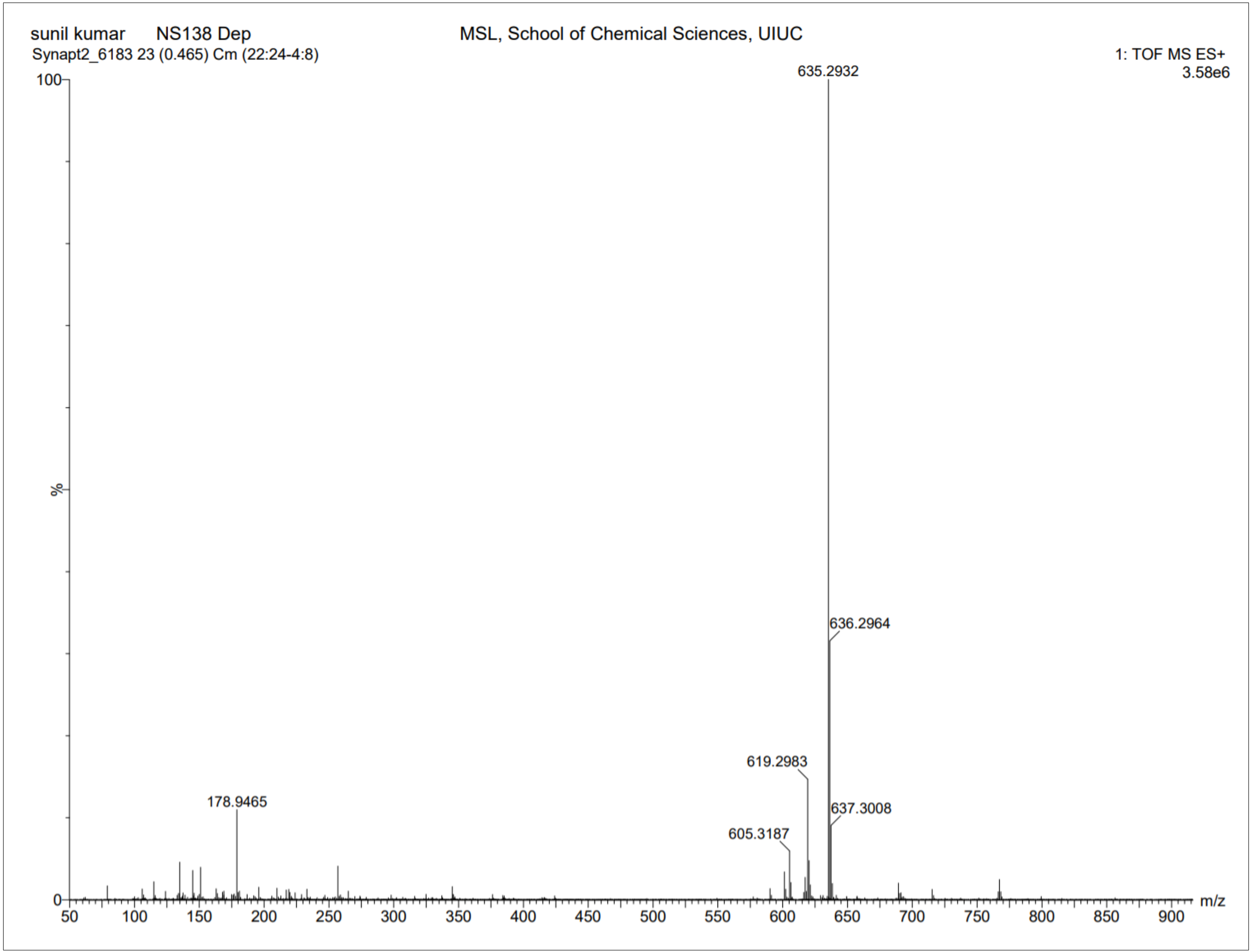

Supplementary Fig. 135. ^{1}H-NMR of NS165 Pro

^{1}H NMR (500 MHz, CDCl3) δ 0.85 – 0.92 (m, 3H), 1.00 – 1.38 (m, 8H), 1.44 – 1.46 (s, 9H), 1.64 – 1.92 (m, 8H), 2.35 – 2.40 (s, 3H), 2.93 – 3.01 (t, J = 7.7 Hz, 3H), 3.38 – 3.51 (t, J = 6.2 Hz, 2H), 3.59 – 3.72 (td, J = 7.0, 5.2 Hz, 2H), 4.81 – 4.87 (s, 2H), 4.87 – 4.95 (t, J = 5.7 Hz, 1H), 6.72 – 6.83 (d, J = 7.9 Hz, 1H), 7.52 – 7.62 (d, J = 8.4 Hz, 1H), 7.62 – 7.69 (d, J = 7.8 Hz, 1H), 7.86 – 7.97 (d, J = 7.8 Hz, 1H), 8.20 – 8.32 (t, J = 5.3 Hz, 1H), 8.58 – 8.67 (d, J = 8.4 Hz, 1H), 8.67 – 8.74 (d, J = 7.9 Hz, 1H), 9.32 – 9.42 (s, 1H), 10.30 – 10.40 (s, 1H). HRMS (m/z): [M]+ calcd. for $C_{37}H_{50}N_8O_7$, 719.3875; found, 719.3874.

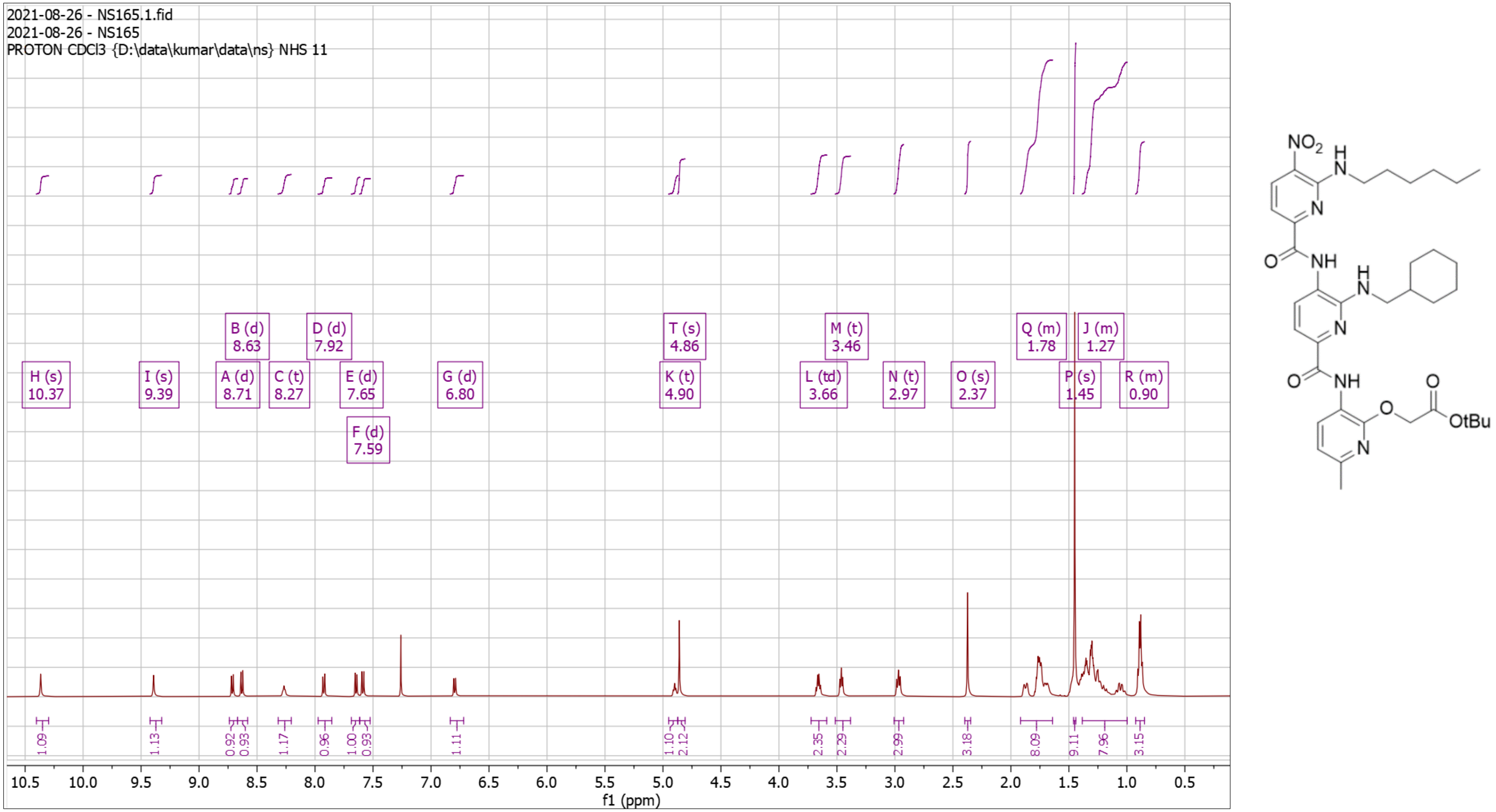

Supplementary Fig. 136. High Resolution Mass Spectrum for NS165 Pro

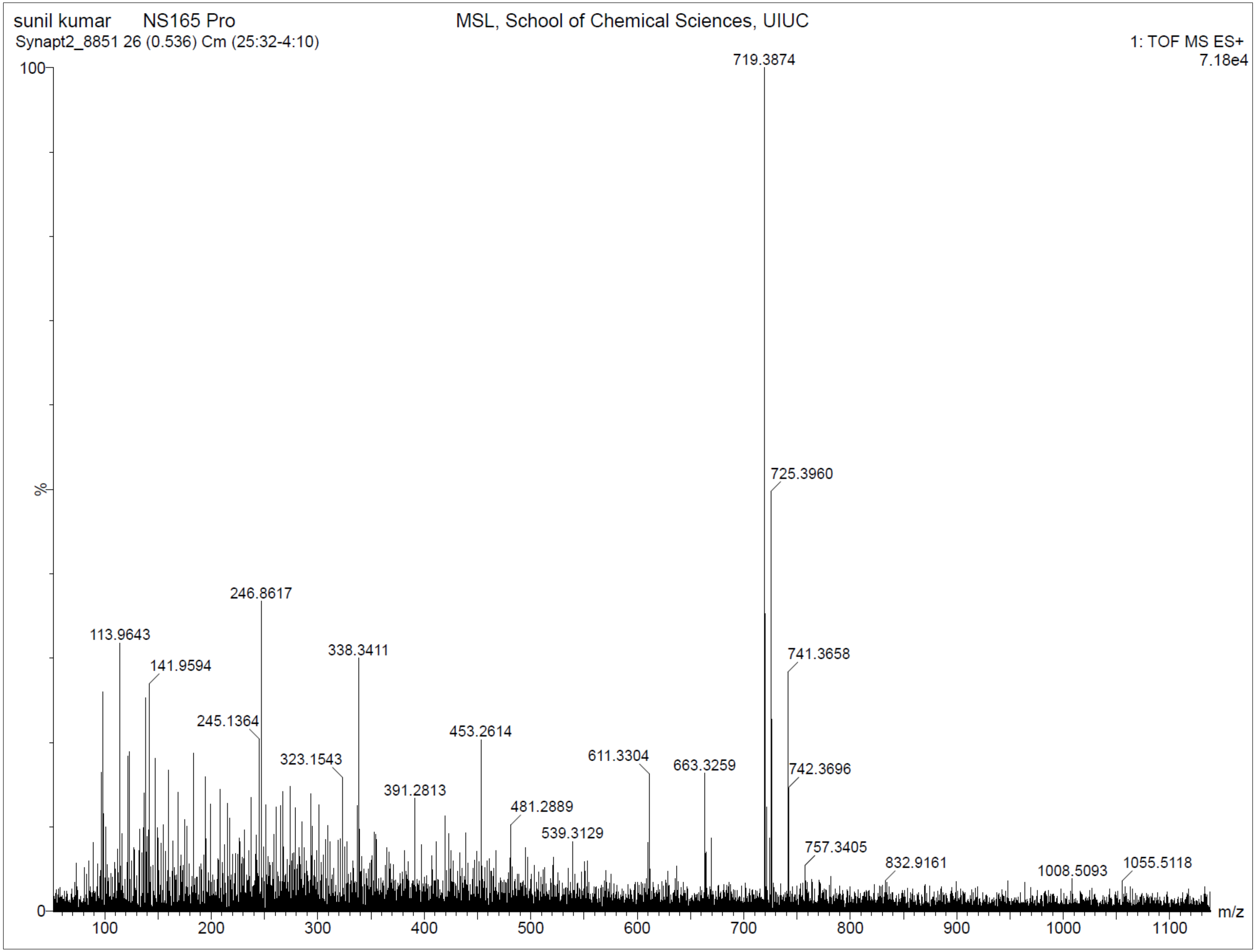

Supplementary Fig. 137. ^{1}H-NMR of NS165 Dep

^{1}H NMR (500 MHz, DMSO) δ 0.76 – 0.84 (t, J = 6.9 Hz, 3H), 0.92 – 1.41 (m, 11H), 1.57 – 1.87 (m, 8H), 2.29 – 2.39 (s, 3H), 3.29 – 3.33 (t, 2H), 3.70 – 3.81 (q, J = 6.6 Hz, 2H), 4.93 – 5.02 (s, 2H), 6.73 – 6.80 (t, J = 5.7 Hz, 1H), 6.91 – 6.96 (d, J = 7.9 Hz, 1H), 7.34 – 7.41 (d, J = 8.4 Hz, 1H), 7.41 – 7.46 (d, J = 7.8 Hz, 1H), 7.94 – 7.99 (d, J = 7.8 Hz, 1H), 8.45 – 8.57 (t, J = 5.7 Hz, 1H), 8.57 – 8.69 (dd, J = 8.2, 3.0 Hz, 2H), 9.75 – 9.82 (s, 1H), 10.29 – 10.43 (s, 1H). HRMS (m/z): [M]+ calcd. for $C_{33}H_{42}N_8O_7$, 663.3249; found, 663.3253.

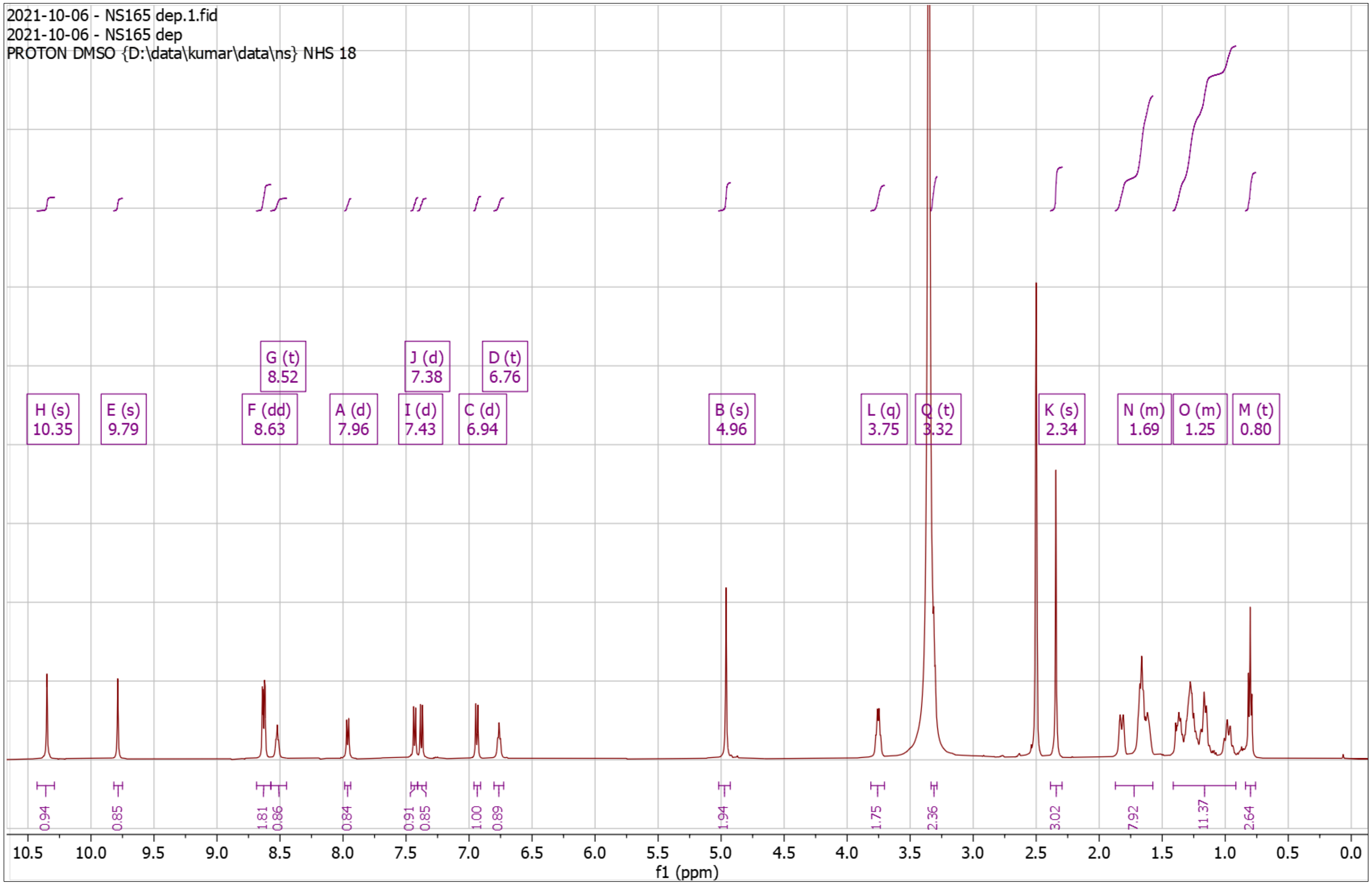

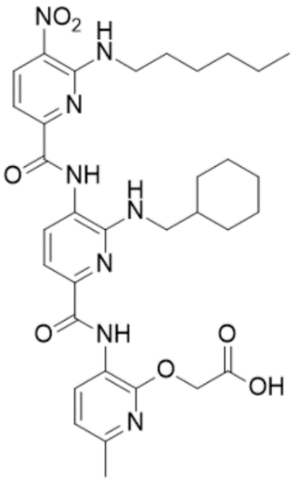

Supplementary Fig. 138. High Resolution Mass Spectrum for NS165 Dep

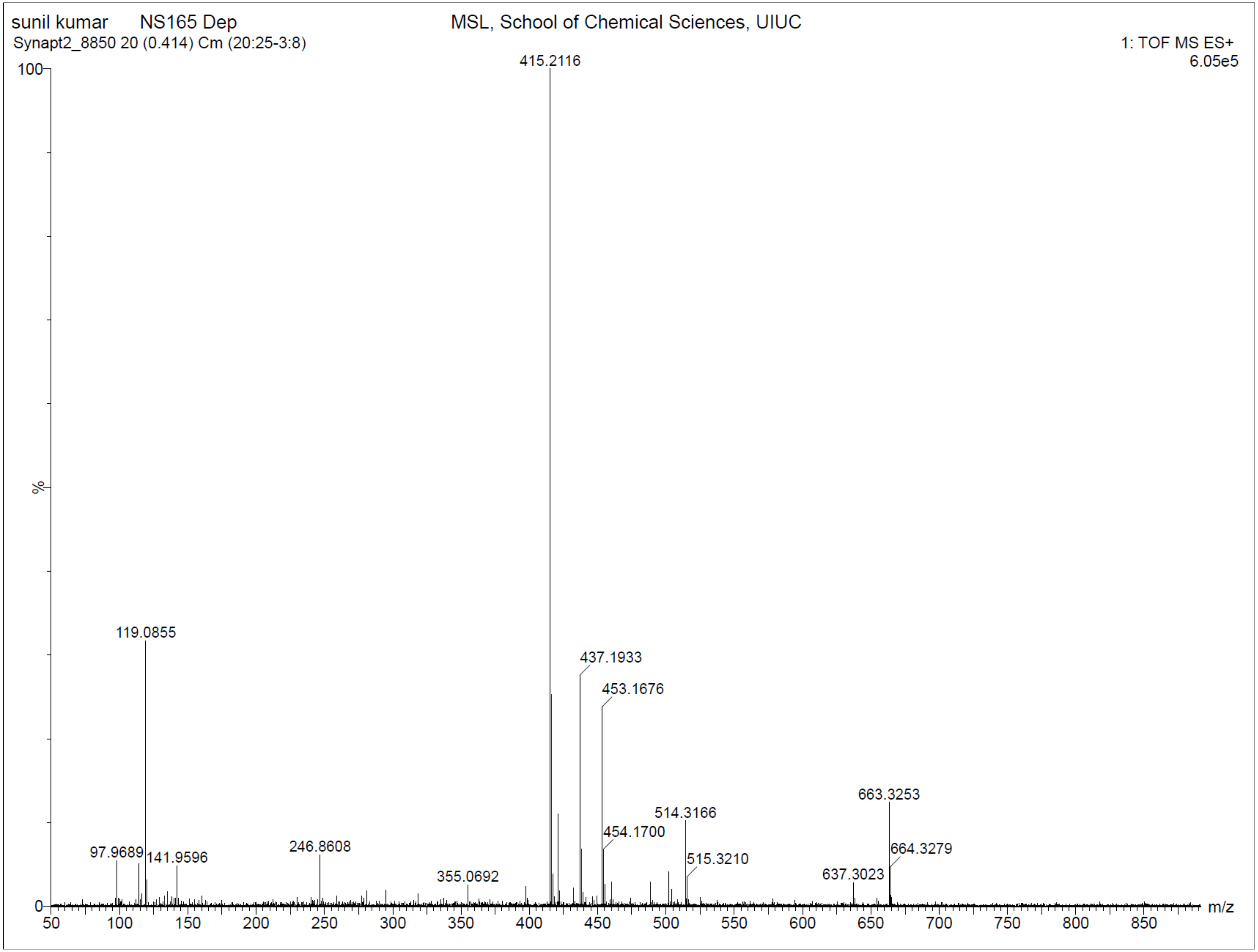

Supplementary Fig. 139. ^{1}H-NMR of NS166 Pro

^{1}H NMR (500 MHz, $CDCl_3$) δ 0.87 – 1.35 (m, 5H), 1.44 – 1.48 (s, 9H), 1.65 – 1.92 (m, 6H), 2.33 – 2.41 (s, 3H), 3.38 – 3.47 (t, J = 6.2 Hz, 2H), 3.86 – 3.94 (q, J = 5.4 Hz, 2H), 3.97 – 4.04 (t, J = 4.9 Hz, 2H), 4.82 – 4.89 (s, 2H), 5.23 – 5.29 (t, J = 5.5 Hz, 1H), 6.72 – 6.80 (d, J = 7.9 Hz, 1H), 7.56 – 7.64 (d, J = 8.2 Hz, 2H), 8.06 – 8.16 (d, J = 7.8 Hz, 1H), 8.47 – 8.51 (t, J = 5.6 Hz, 1H), 8.54 – 8.62 (d, J = 8.4 Hz, 1H), 8.63 – 8.71 (d, J = 7.8 Hz, 1H), 9.45 – 9.53 (s, 1H), 10.26 – 10.33 (s, 1H). HRMS (m/z): [M]+ calcd. for $C_{33}H_{42}N_8O_8$, 679.3198; found, 679.3203.

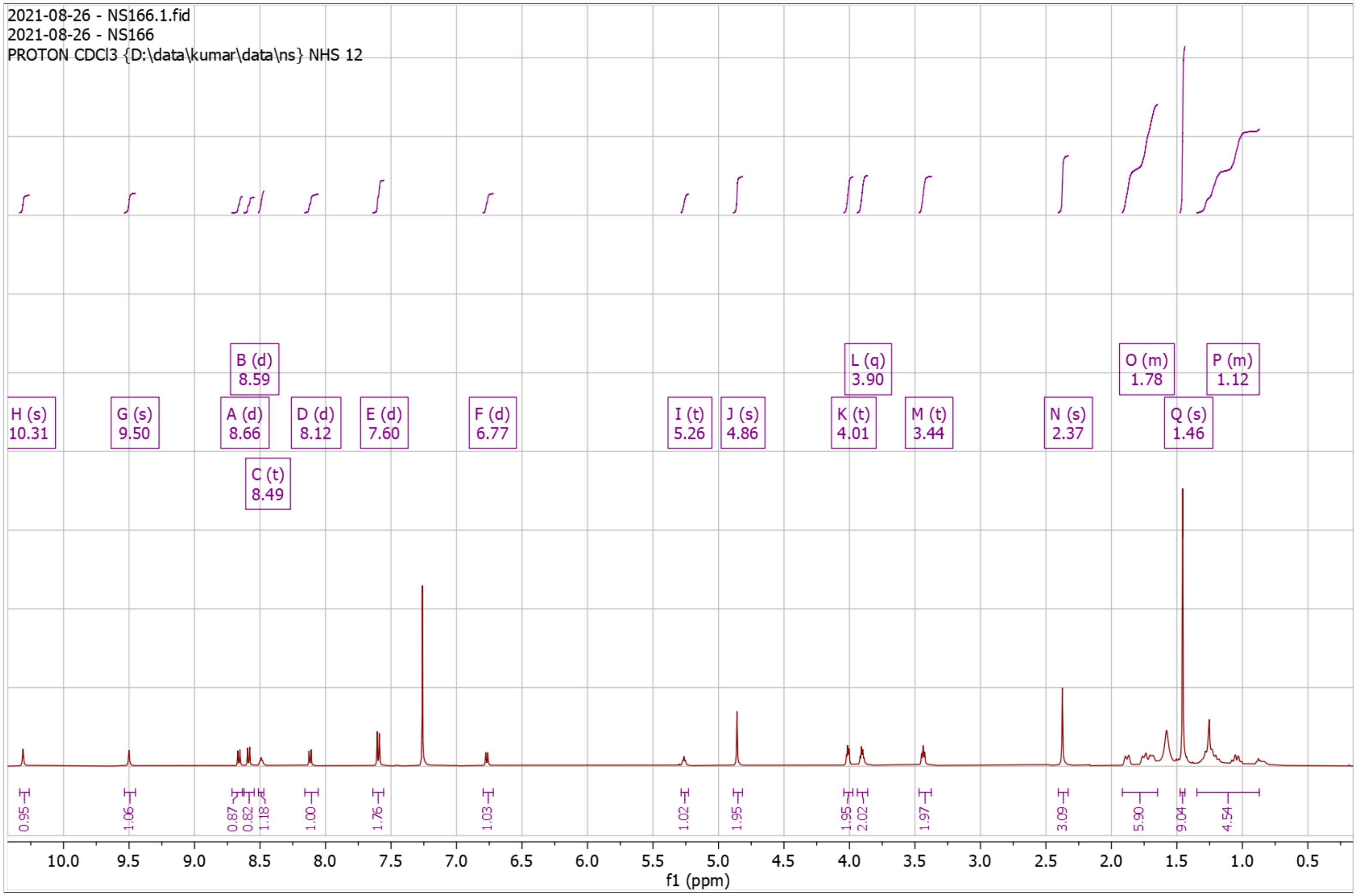

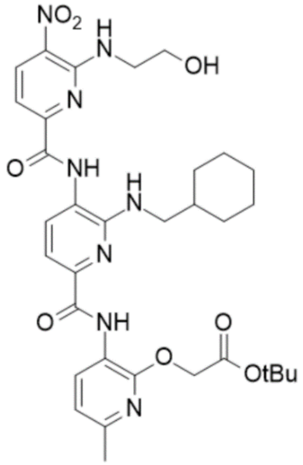

Supplementary Fig. 140. High Resolution Mass Spectrum for NS166 Pro

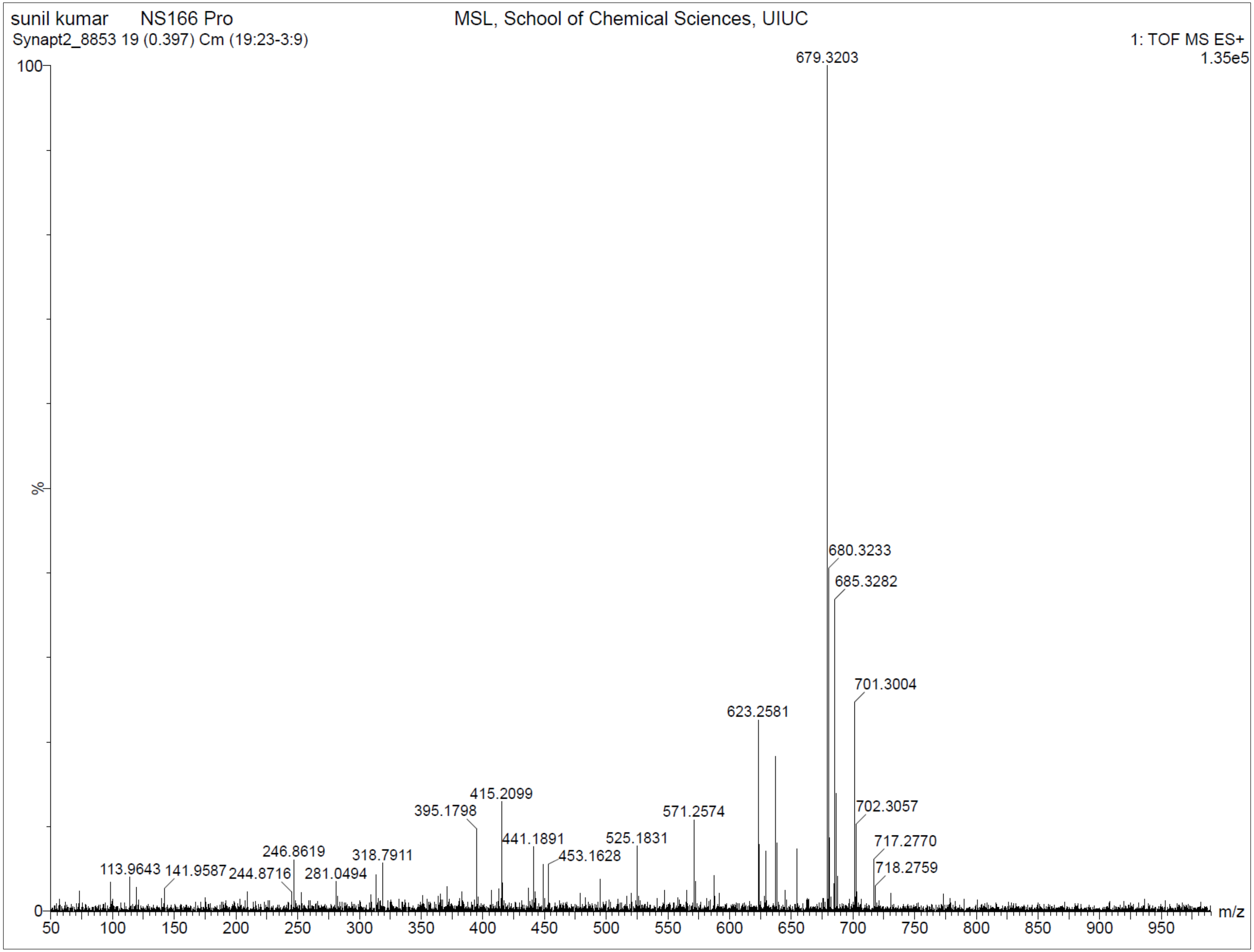

Supplementary Fig. 141. ^{1}H-NMR of NS166 Dep

^{1}H NMR (500 MHz, DMSO) δ 0.90 – 1.25 (dt, J = 96.3, 12.3 Hz, 5H), 1.54 – 1.85 (m, 6H), 2.29 – 2.32 (s, 3H), 3.27 – 3.32 (t, J = 6.1 Hz, 2H), 3.64 – 3.70 (t, J = 5.5 Hz, 2H), 3.83 – 3.90 (q, J = 5.5 Hz, 2H), 4.75 – 4.79 (s, 2H), 6.65 – 6.75 (t, J = 5.6 Hz, 1H), 6.78 – 6.88 (d, J = 7.9 Hz, 1H), 7.33 – 7.47 (dd, J = 8.1, 5.5 Hz, 2H), 7.88 – 7.94 (d, J = 7.8 Hz, 1H), 8.52 – 8.60 (m, 2H), 8.60 – 8.70 (d, J = 8.4 Hz, 1H), 9.89 – 10.03 (s, 1H), 10.34 – 10.48 (s, 1H). HRMS (m/z): [M]+ calcd. for $C_{29}H_{34}N_8O_8$, 623.2572; found, 623.2581.

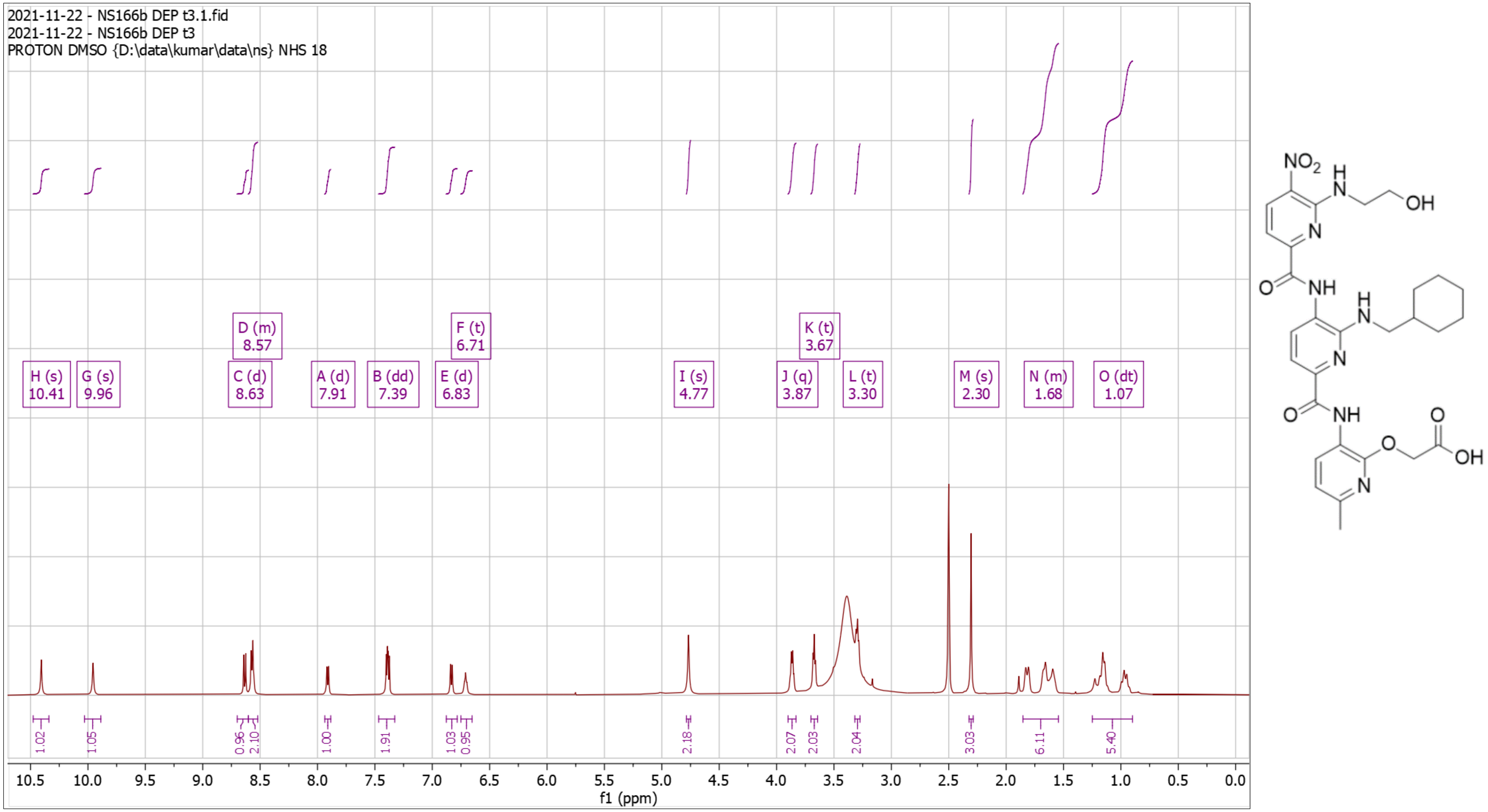

Supplementary Fig. 142. High Resolution Mass Spectrum for NS166 Dep

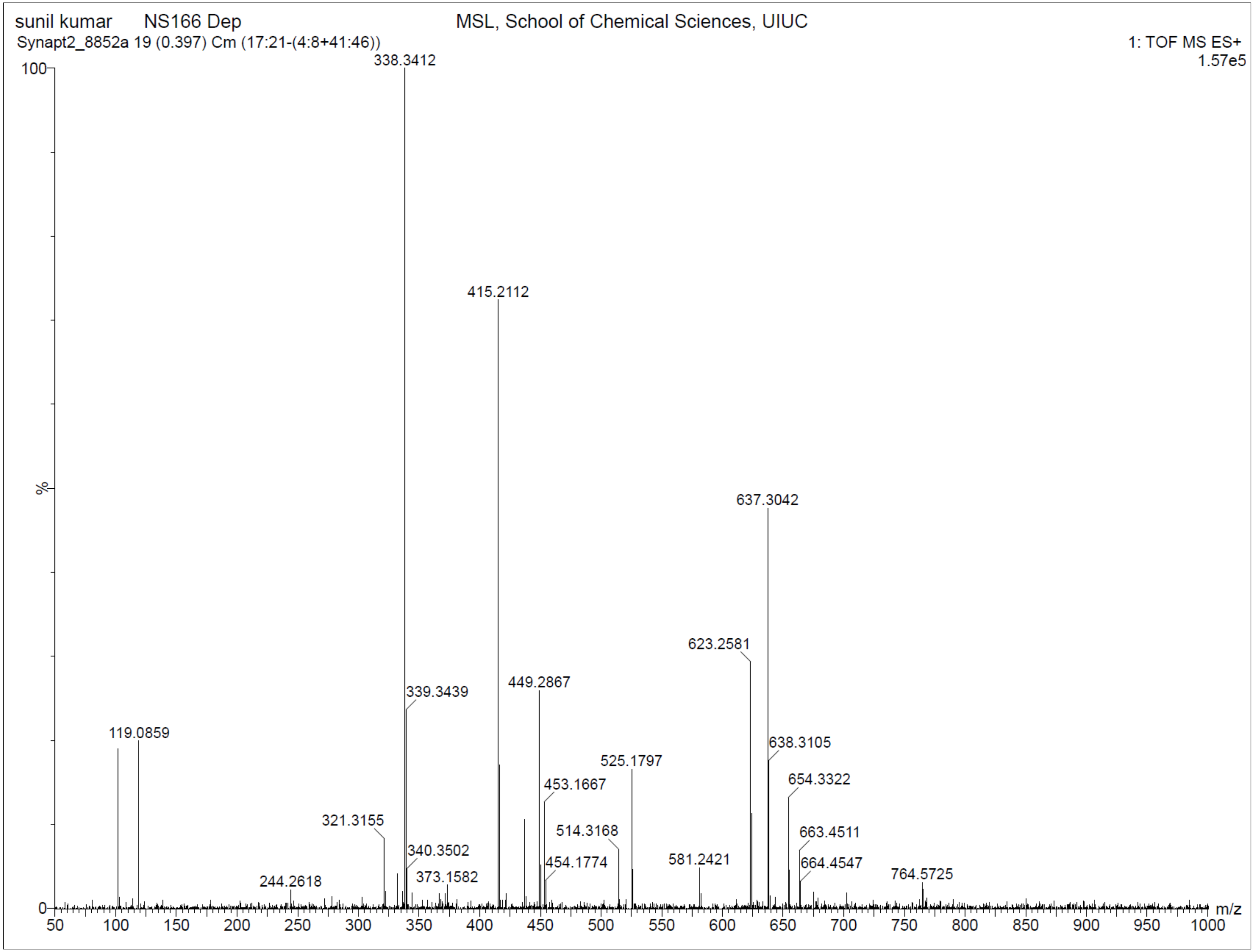

Supplementary Fig. 143. ^{1}H-NMR of NS168 Pro

^{1}H NMR (500 MHz, $CDCl_3$) δ 0.85 – 1.30 (m, 5H), 1.45 – 1.49 (s, 9H), 1.53 – 1.90 (m, 6H), 2.31 – 2.44 (s, 3H), 2.89 – 3.05 (t, J = 7.0 Hz, 2H), 3.36 – 3.47 (t, J = 6.2 Hz, 2H), 3.83 – 3.95 (q, J = 6.6 Hz, 2H), 4.80 – 4.94 (m, 3H), 5.32 – 5.45 (s, 1H), 6.66 – 6.76 (d, J = 8.3 Hz, 2H), 6.76 – 6.83 (d, J = 7.9 Hz, 1H), 7.04 – 7.10 (d, J = 8.1 Hz, 2H), 7.54 – 7.61 (d, J = 8.3 Hz, 1H), 7.60 – 7.68 (d, J = 7.8 Hz, 1H), 7.84 – 7.93 (d, J = 7.8 Hz, 1H), 8.25 – 8.36 (t, J = 5.4 Hz, 1H), 8.56 – 8.65 (d, J = 8.3 Hz, 1H), 8.68 – 8.74 (d, J = 7.9 Hz, 1H), 9.27 – 9.41 (s, 1H), 10.35 – 10.42 (s, 1H). HRMS (m/z): [M]+ calcd. for $C_{39}H_{46}N_8O_8$, 755.3511; found, 755.3497.

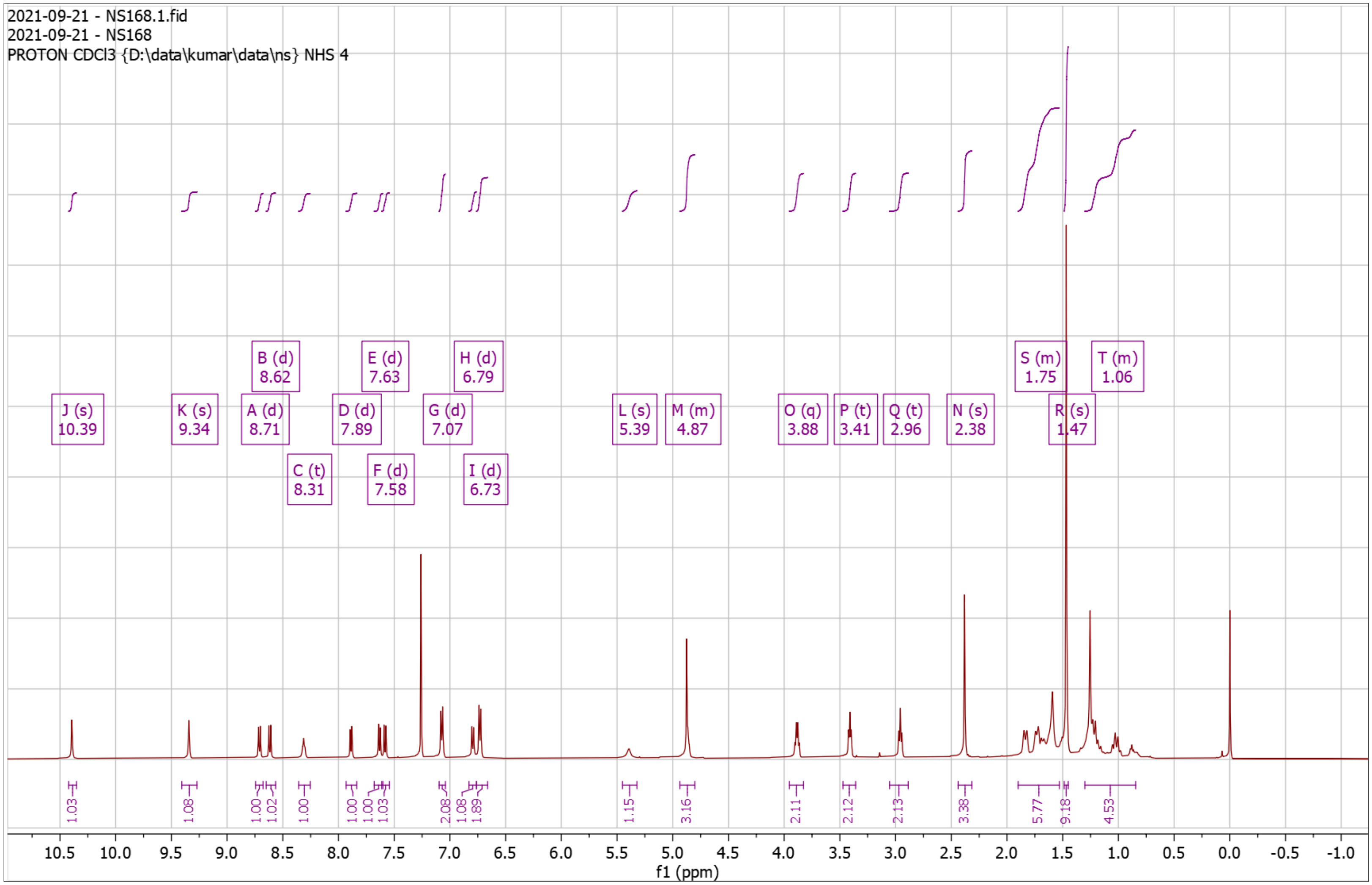

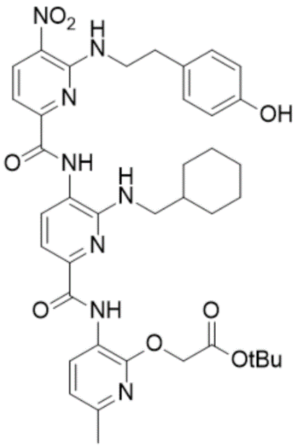

Supplementary Fig. 144. High Resolution Mass Spectrum for NS168 Pro

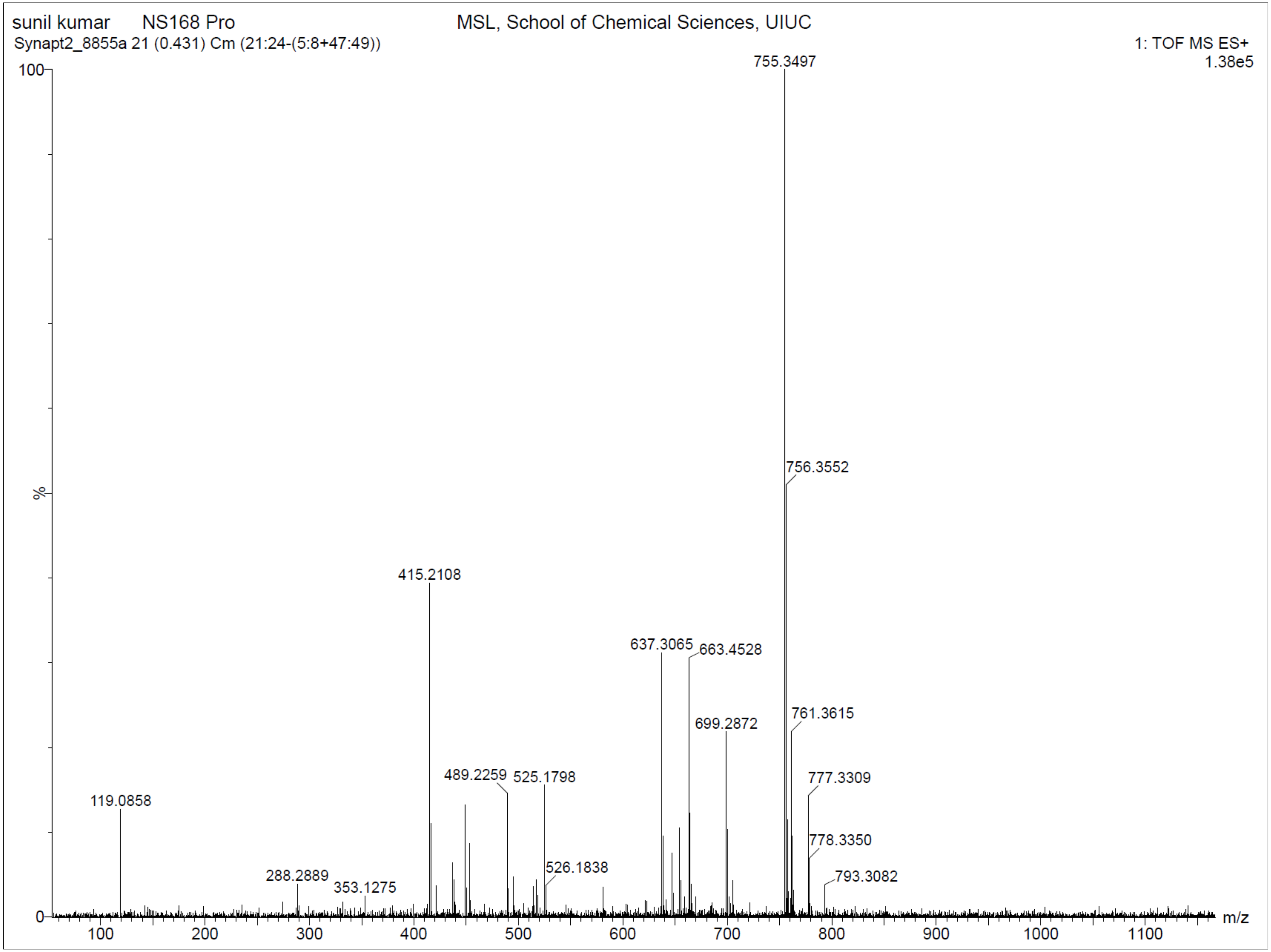

Supplementary Fig. 145. ^{1}H-NMR of NS168 Dep

^{1}H NMR (500 MHz, DMSO) δ 0.77 – 1.21 (m, 5H), 1.49 – 1.90 (m, 6H), 2.33 – 2.37 (s, 3H), 2.82 – 2.89 (t, *J* = 7.1 Hz, 2H), 3.27 – 3.31 (t, *J* = 6.4 Hz, 2H), 3.92 – 3.99 (q, *J* = 6.7 Hz, 2H), 4.96 – 4.98 (s, 2H), 6.60 – 6.65 (d, *J* = 8.3 Hz, 2H), 6.73 – 6.79 (t, *J* = 5.7 Hz, 1H), 6.92 – 6.97 (d, *J* = 7.9 Hz, 1H), 7.04 – 7.09 (d, *J* = 8.1 Hz, 2H), 7.35 – 7.40 (d, *J* = 8.4 Hz, 1H), 7.41 – 7.47 (d, *J* = 7.7 Hz, 1H), 7.90 – 7.95 (d, *J* = 7.8 Hz, 1H), 8.47 – 8.52 (t, *J* = 5.7 Hz, 1H), 8.60 – 8.67 (dd, *J* = 8.1, 6.3 Hz, 2H), 9.14 – 9.17 (s, 1H), 9.84 – 9.87 (s, 1H), 10.35 – 10.39 (s, 1H). HRMS (m/z): [M]+ calcd. for $C_{35}H_{38}N_{8}O_{8}$, 699.2885; found, 699.2873.

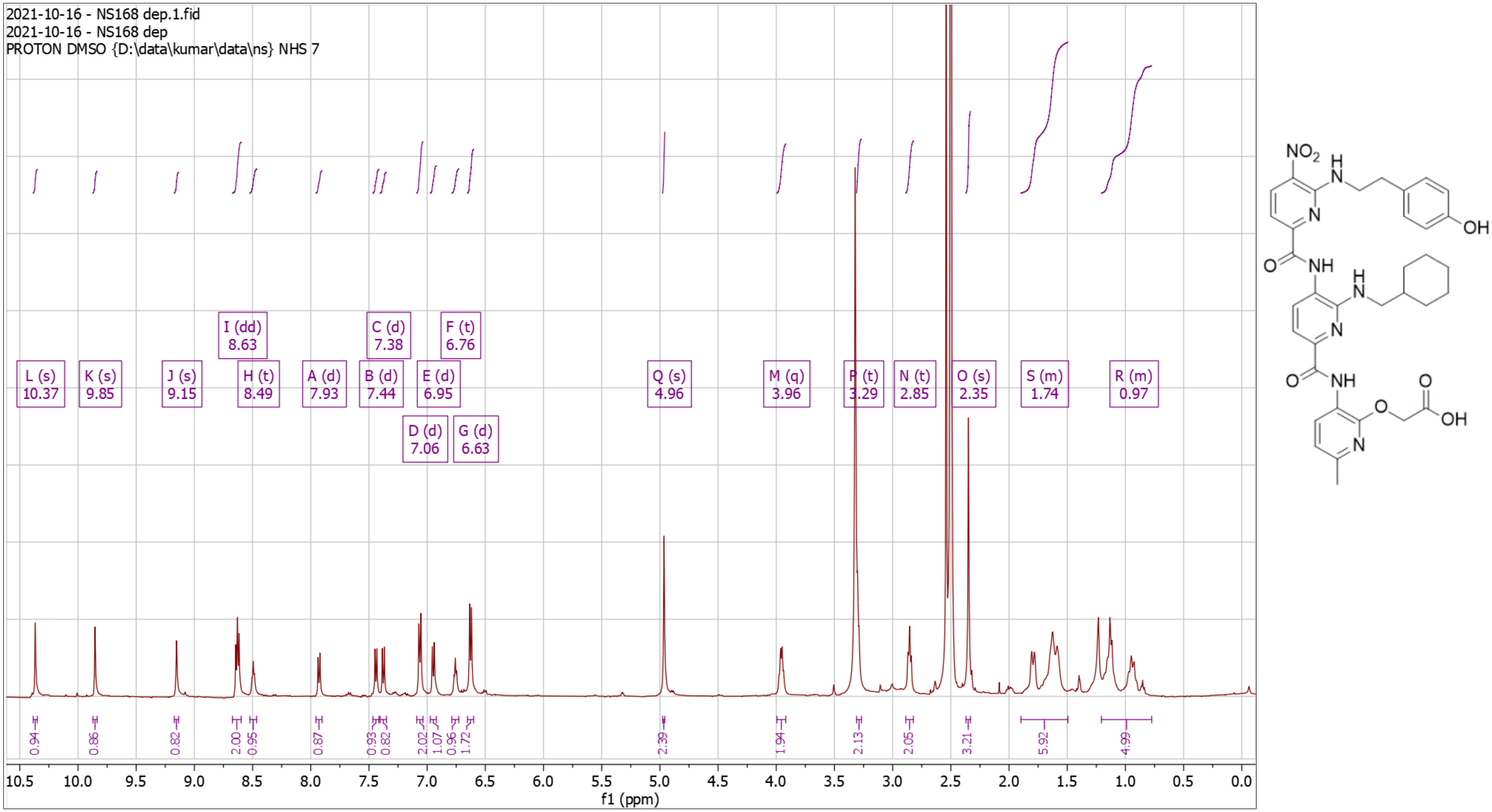

Supplementary Fig. 146. High Resolution Mass Spectrum for NS168 Dep

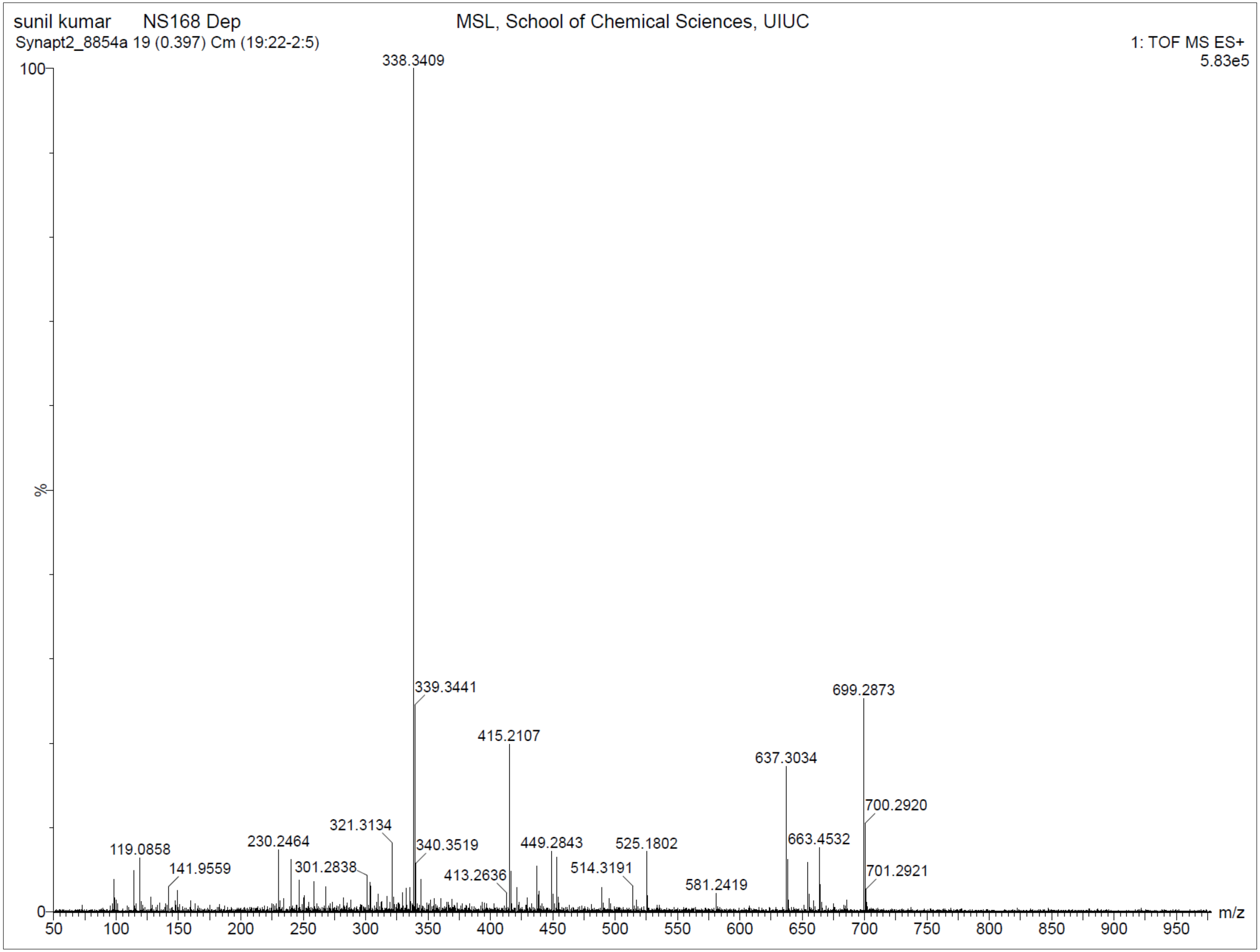

Supplementary Fig. 147. ^{1}H-NMR of NS169 Pro

^{1}H NMR (500 MHz, CDCl3) δ 0.71 – 1.38 (m, 5H), 1.44 – 1.47 (s, 9H), 1.55 – 1.96 (m, 6H), 2.37 – 2.40 (s, 3H), 2.50 – 2.55 (s, 3H), 3.49 – 3.54 (d, J = 6.8 Hz, 2H), 4.85 – 4.89 (s, 2H), 4.93 – 4.97 (d, J = 5.6 Hz, 2H), 6.79 – 6.84 (d, J = 7.9 Hz, 1H), 7.63 – 7.69 (dd, J = 8.1, 3.4 Hz, 2H), 8.21 – 8.29 (m, 2H), 8.61 – 8.67 (m, 2H), 8.73 – 8.78 (d, J = 7.9 Hz, 1H), 8.86 – 8.92 (t, J = 5.6 Hz, 1H), 9.93 – 9.96 (s, 1H), 10.40 – 10.43 (s, 1H). HRMS (m/z): [M]+ calcd. for $C_{37}H_{44}N_{10}O_{7}$, 741.3467; found, 741.3457.

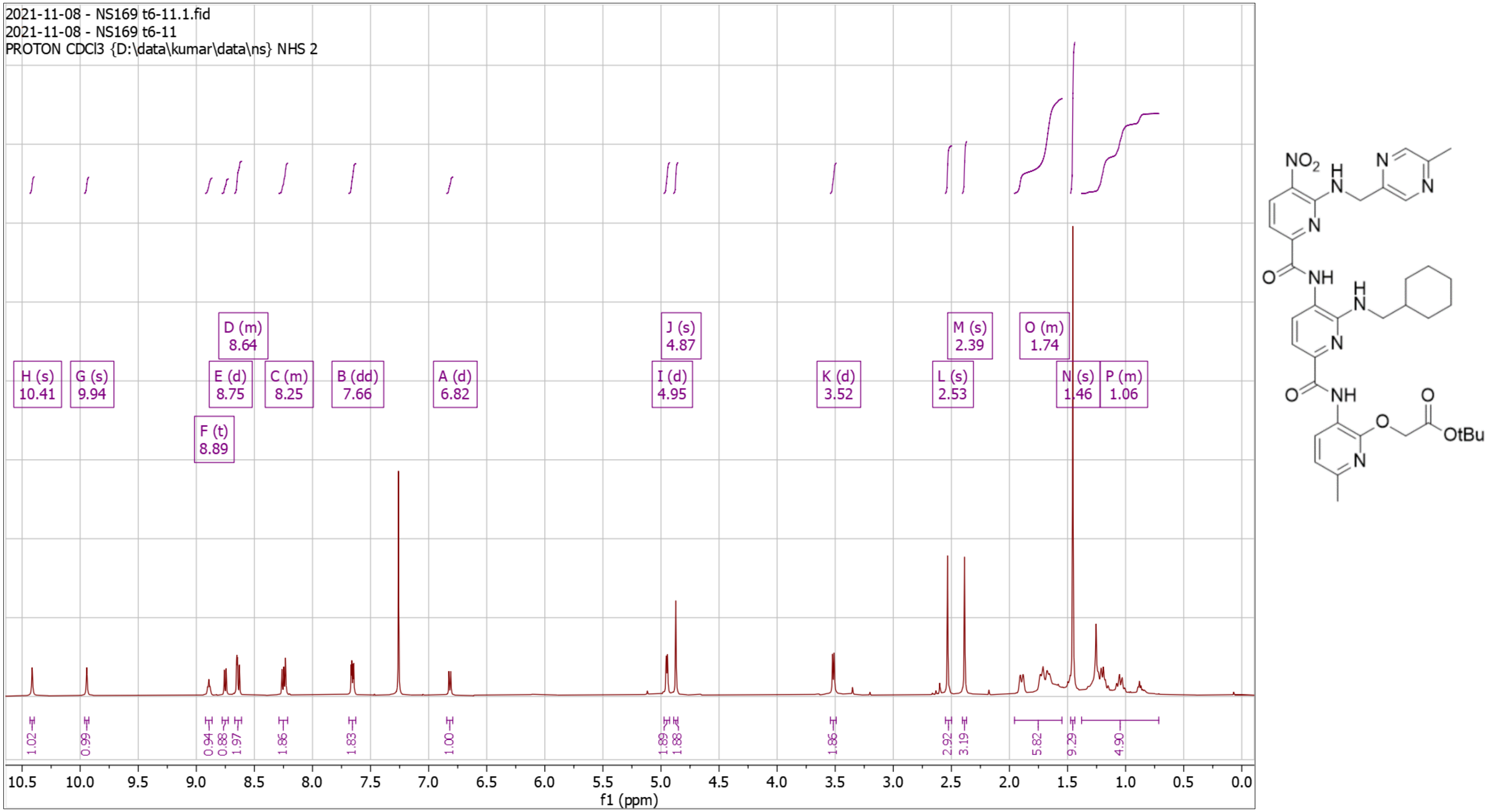

Supplementary Fig. 148. High Resolution Mass Spectrum for NS169 Pro

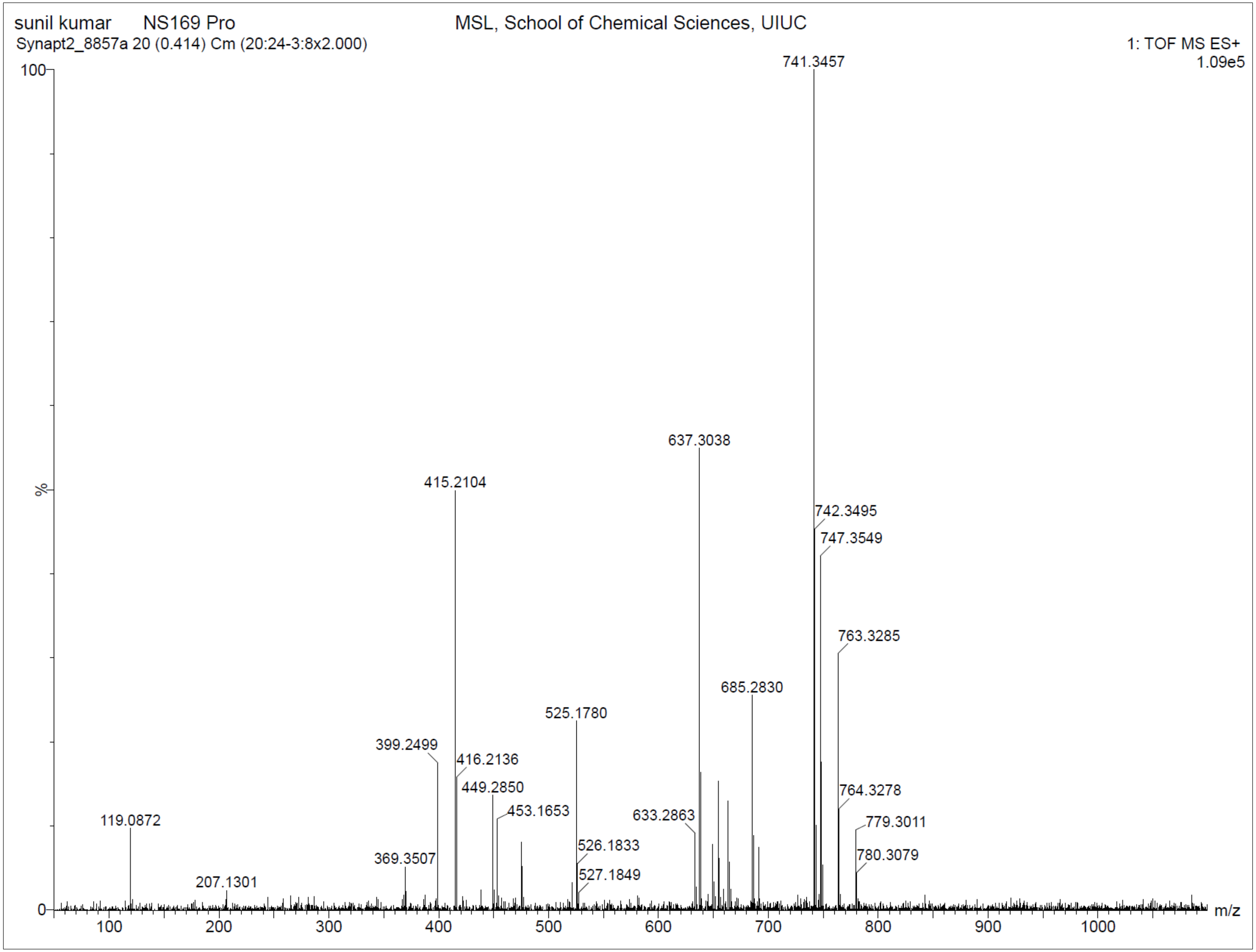

Supplementary Fig. 149. ^{1}H-NMR of NS169 Dep

^{1}H NMR (500 MHz, DMSO) δ 0.90 – 1.19 (dd, J = 83.9, 10.2 Hz, 5H), 1.50 – 1.85 (m, 6H), 2.34 – 2.36 (s, 3H), 2.45 – 2.46 (s, 3H), 3.33 – 3.37 (d, J = 6.9 Hz, 2H), 4.92 – 4.99 (s, 2H), 5.05 – 5.16 (d, J = 5.6 Hz, 2H), 6.74 – 6.84 (t, J = 5.9 Hz, 1H), 6.91 – 6.97 (d, J = 8.0 Hz, 1H), 7.36 – 7.48 (dd, J = 8.1, 5.4 Hz, 2H), 7.87 – 7.94 (d, J = 7.8 Hz, 1H), 8.38 – 8.46 (s, 1H), 8.60 – 8.73 (m, 3H), 9.18 – 9.26 (t, J = 5.6 Hz, 1H), 9.96 – 10.05 (s, 1H), 10.31 – 10.42 (s, 1H). HRMS (m/z): [M]+ calcd. for $C_{33}H_{36}N_{10}O_7$, 685.2841; found, 685.2844.

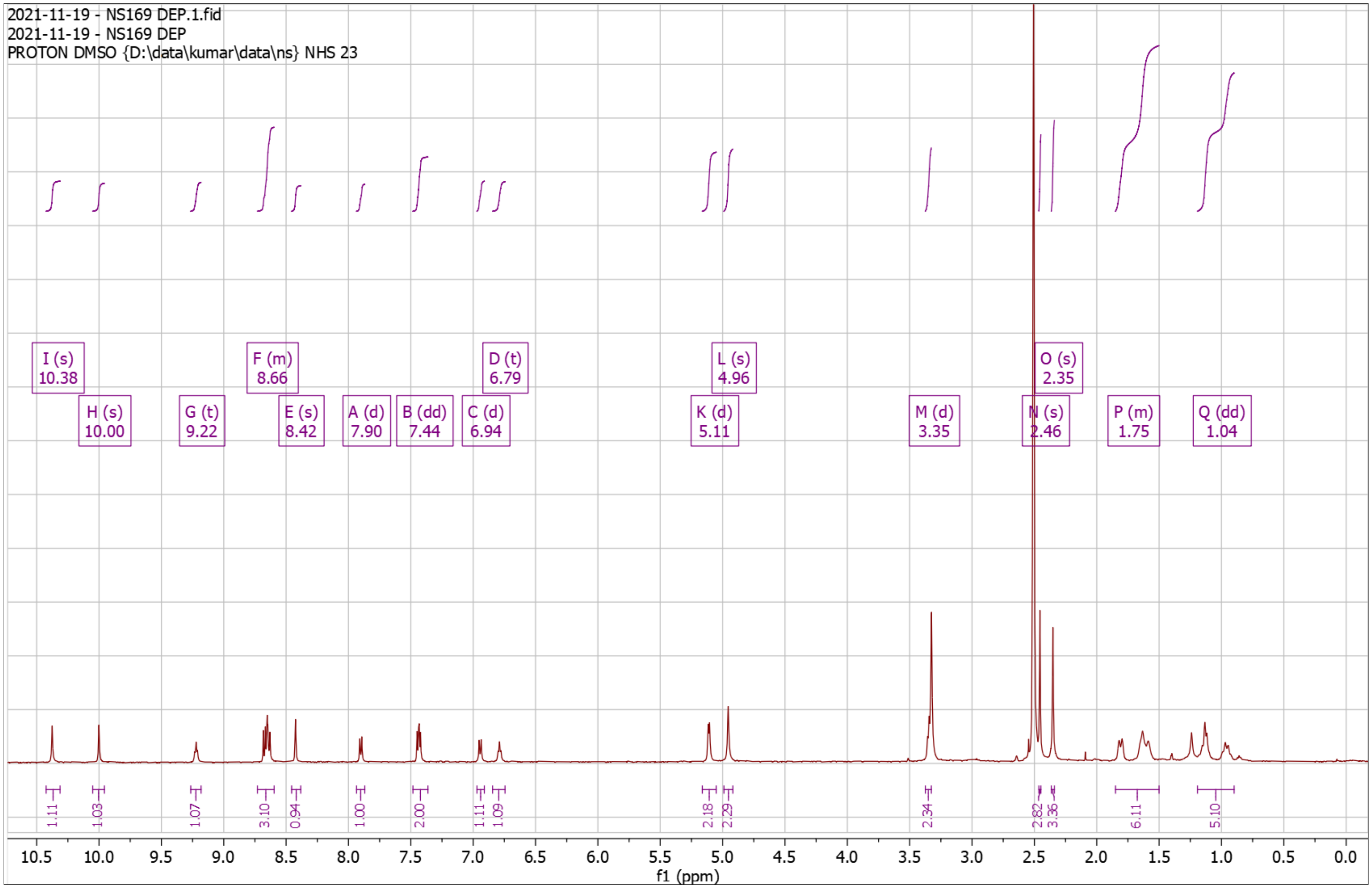

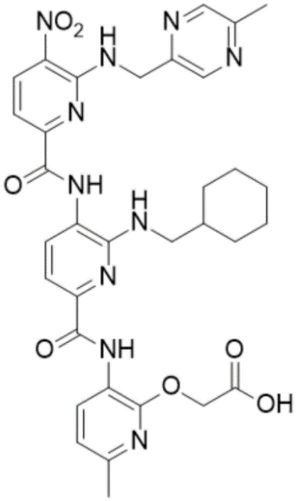

Supplementary Fig. 150. High Resolution Mass Spectrum for NS169 Dep

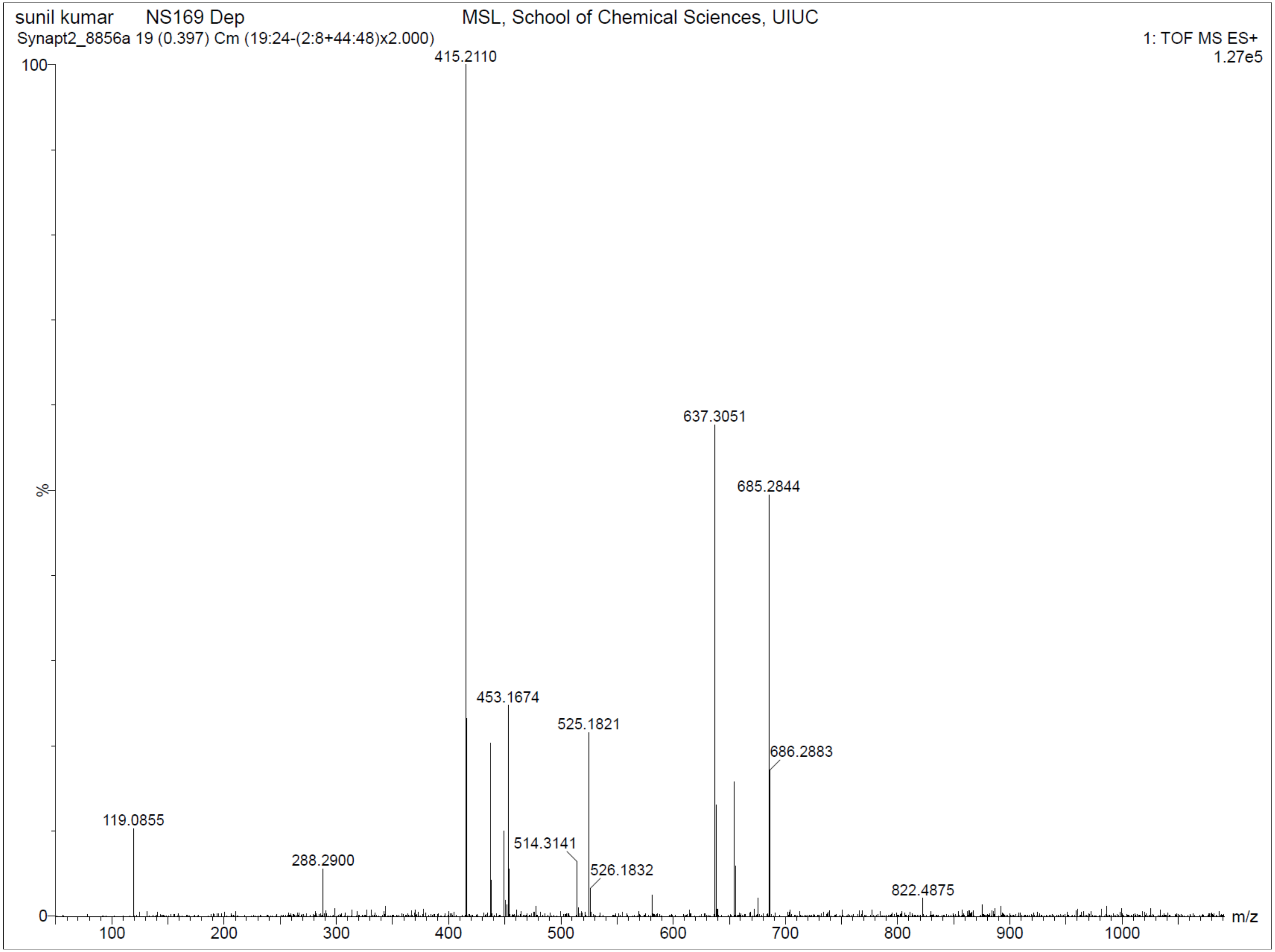

Supplementary Fig. 151. ^{1}**H-NMR of NS174 Pro**

^{1}H NMR (500 MHz, CDCl3) δ 0.82 – 1.34 (m, 5H), 1.42 – 1.50 (s, 9H), 1.64 – 1.93 (m, 6H), 2.09 – 2.11 (t, J = 2.7 Hz, 1H), 2.36 – 2.39 (s, 3H), 2.64 – 2.69 (td, J = 6.6, 2.7 Hz, 2H), 3.43 – 3.52 (d, J = 5.7 Hz, 2H), 3.85 – 3.93 (q, J = 6.3 Hz, 2H), 4.84 – 4.88 (s, 2H), 4.93 – 5.00 (s, 1H), 6.77 – 6.82 (d, J = 7.9 Hz, 1H), 7.62 – 7.67 (dd, J = 8.1, 2.5 Hz, 2H), 7.91 – 7.97 (d, J = 7.9 Hz, 1H), 8.47 – 8.53 (t, J = 5.9 Hz, 1H), 8.63 – 8.68 (d, J = 8.4 Hz, 1H), 8.68 – 8.73 (d, J = 7.9 Hz, 1H), 9.36 – 9.40 (s, 1H), 10.34 – 10.38 (s, 1H). HRMS (m/z): [M]+ calcd. for $C_{35}H_{42}N_8O_7$, 687.3249; found, 687.3240.

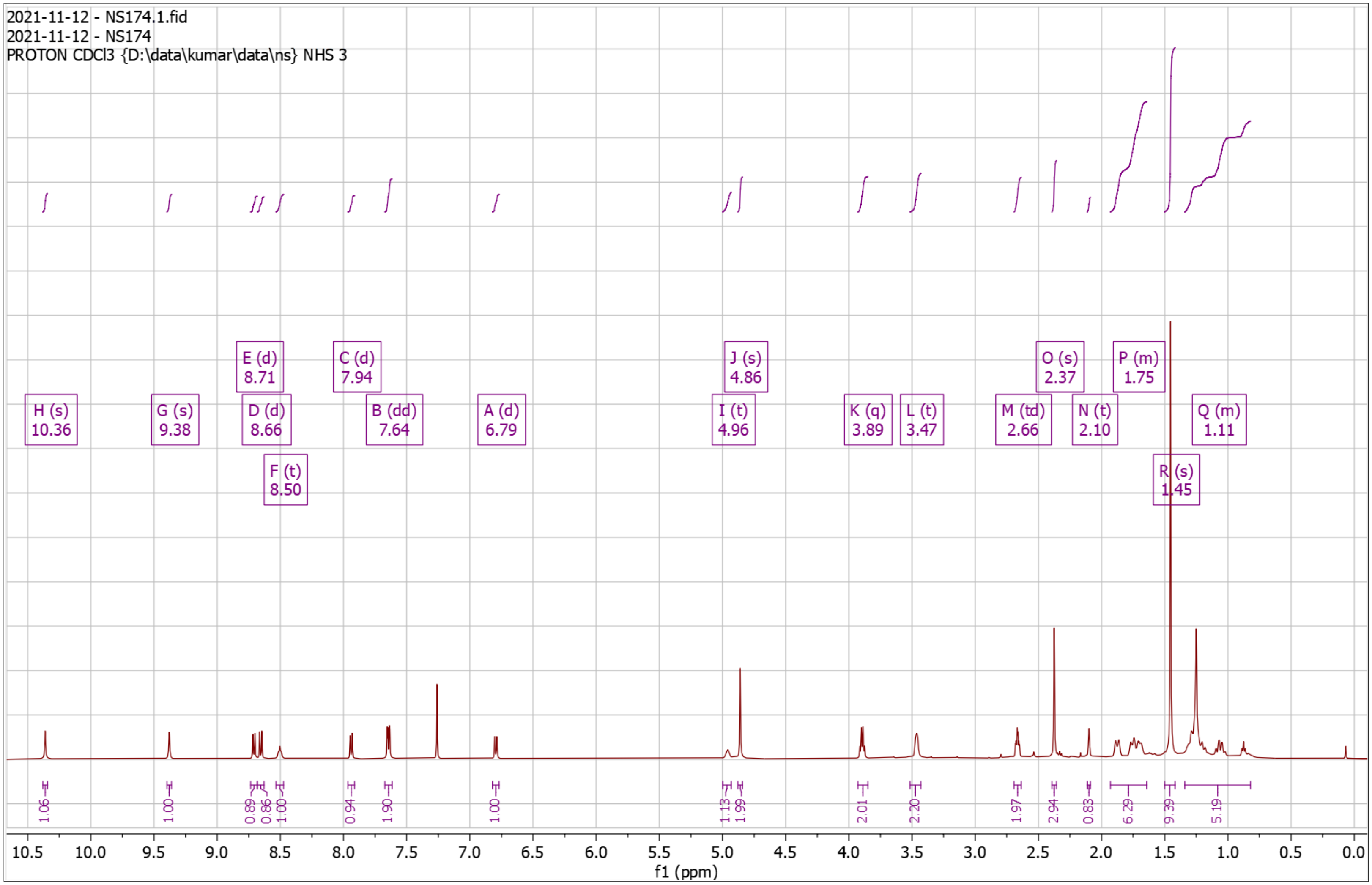

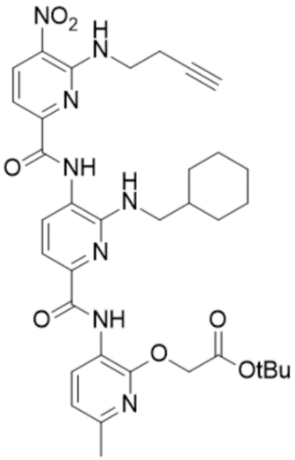

Supplementary Fig. 152. High Resolution Mass Spectrum for NS174 Pro

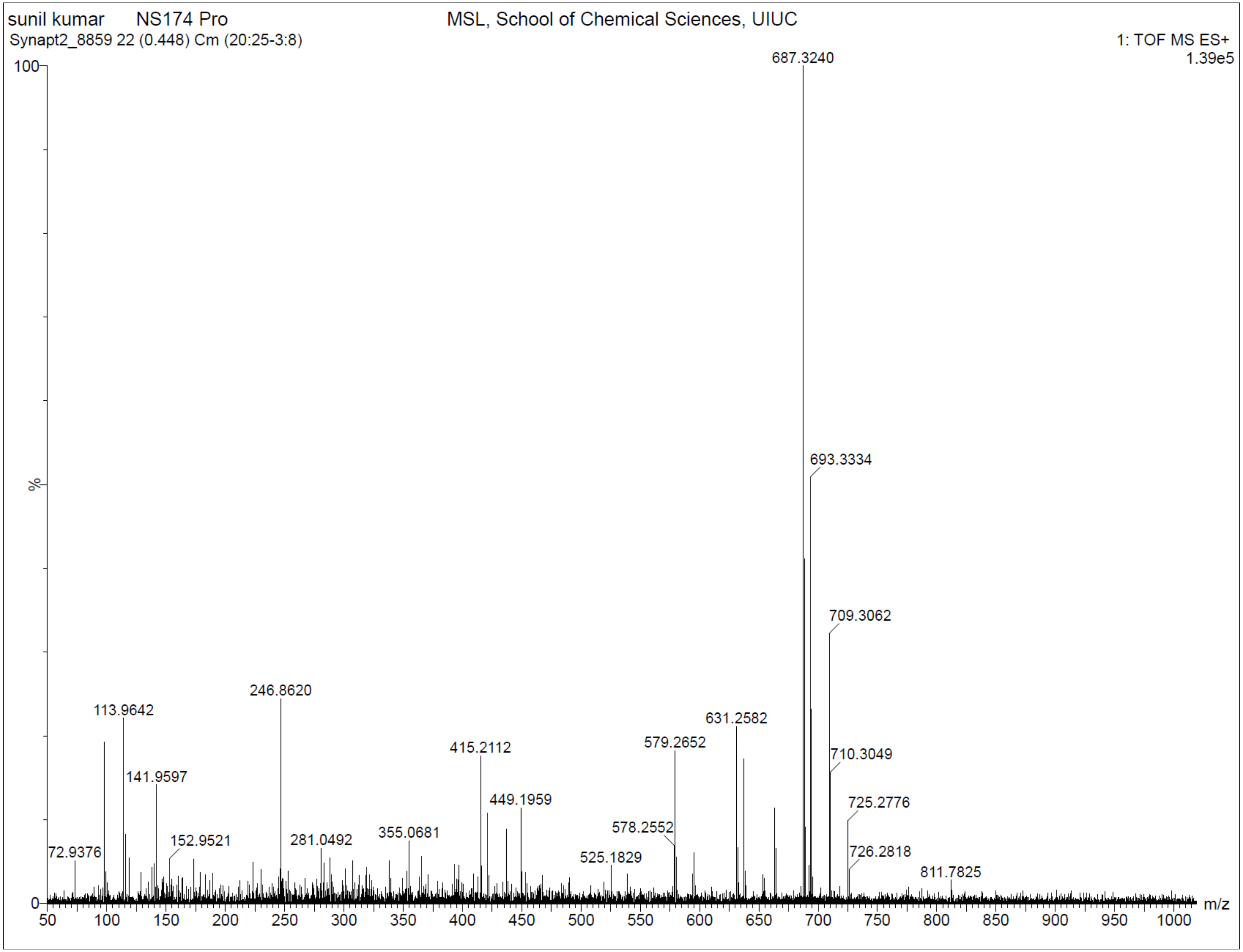

Supplementary Fig. 153. ^{1}H-NMR of NS174 Dep

^{1}H NMR (500 MHz, DMSO) δ 0.90 – 1.28 (m, 5H), 1.55 – 1.88 (m, 6H), 2.32 – 2.36 (s, 3H), 2.57 – 2.63 (td, J = 6.8, 2.7 Hz, 2H), 2.84 – 2.91 (t, J = 2.6 Hz, 1H), 3.28 – 3.34 (t, J = 6.3 Hz, 2H), 3.86 – 3.99 (q, J = 6.6 Hz, 2H), 4.94 – 4.97 (s, 2H), 6.65 – 6.77 (t, J = 5.8 Hz, 1H), 6.88 – 6.99 (d, J = 8.0 Hz, 1H), 7.38 – 7.47 (d, J = 8.1 Hz, 2H), 7.83 – 7.91 (d, J = 7.7 Hz, 1H), 8.58 – 8.71 (m, 3H), 9.85 – 9.95 (s, 1H), 10.32 – 10.42 (s, 1H), 12.80 – 13.13 (s, 1H). HRMS (m/z): [M]+ calcd. for $C_{31}H_{34}N_8O_7$, 631.2623; found, 631.2605.

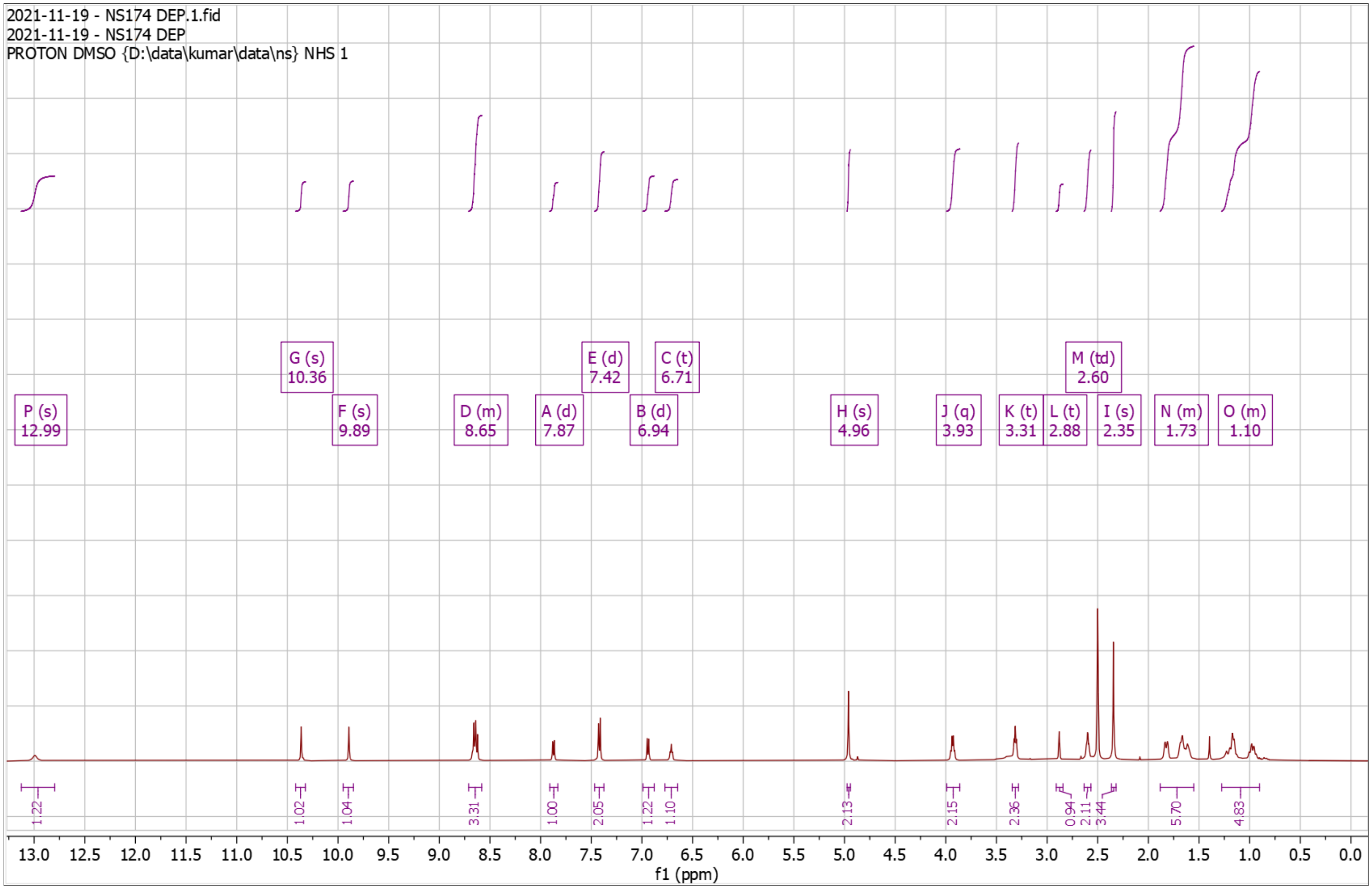

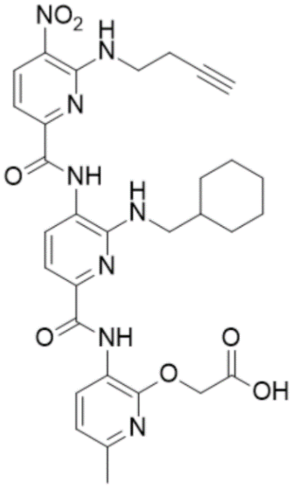

Supplementary Fig. 154. High Resolution Mass Spectrum for NS174 Dep

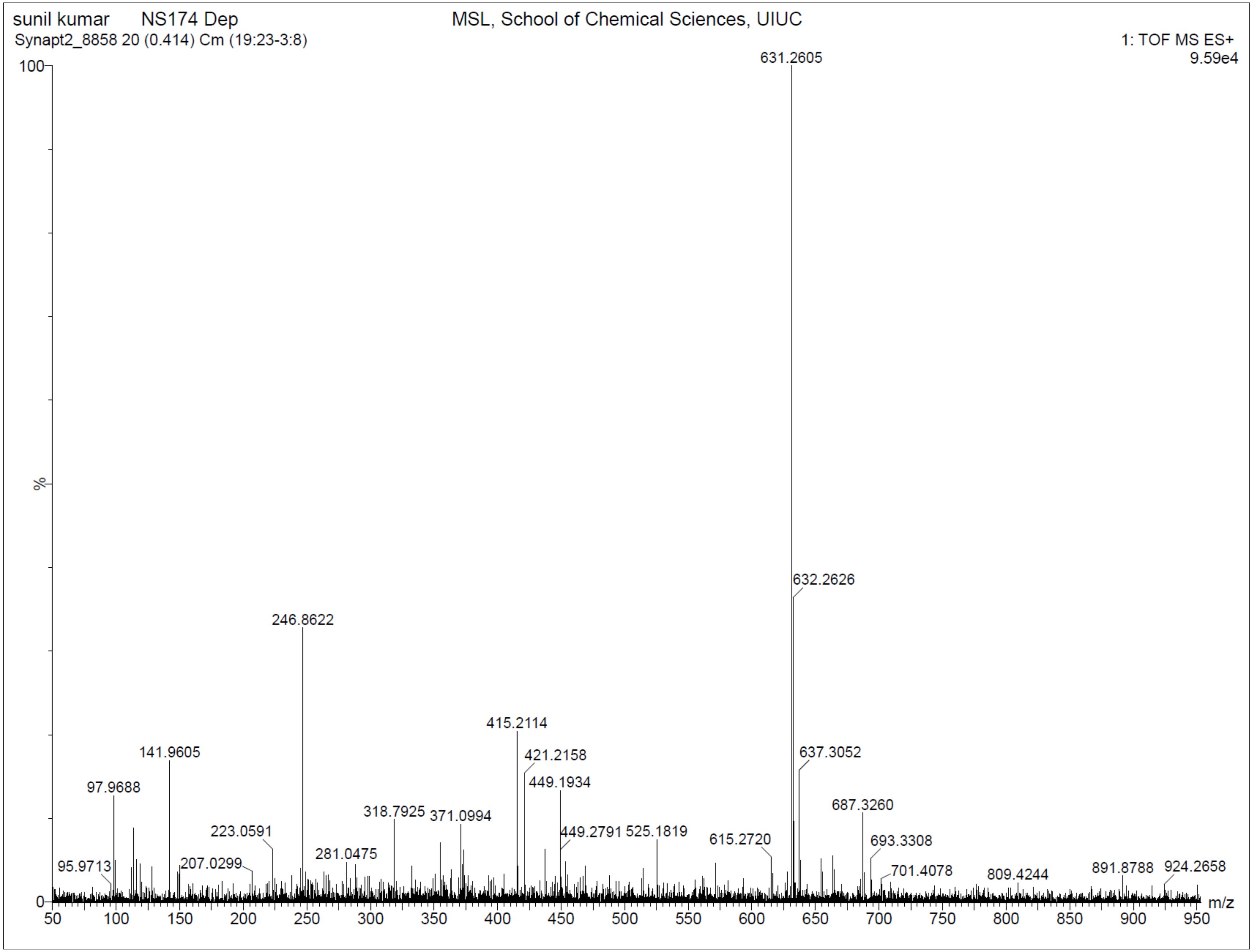

Supplementary Fig. 155. ^{1}H-NMR of NS163

^{1}H NMR (500 MHz, DMSO) δ 0.99 – 1.33 (m, 4H), 1.39 – 1.80 (m, 6H), 2.30 – 2.38 (d, J = 4.0 Hz, 3H), 3.05 – 3.14 (d, J = 13.0 Hz, 2H), 3.97 – 4.11 (q, J = 6.7 Hz, 2H), 4.94 – 4.97 (s, 1H), 5.00 – 5.04 (s, 1H), 6.77 – 6.83 (dd, J = 9.7, 4.8 Hz, 1H), 6.92 – 6.97 (dd, J = 8.0, 4.6 Hz, 1H), 6.98 – 7.04 (dt, J = 9.3, 4.6 Hz, 1H), 7.20 – 7.23 (d, J = 2.5 Hz, 1H), 7.29 – 7.32 (d, J = 7.6 Hz, 1H), 7.35 – 7.38 (d, J = 8.4 Hz, 1H), 7.42 – 7.45 (dd, J = 7.7, 3.3 Hz, 1H), 7.54 – 7.58 (dd, J = 8.0, 3.2 Hz, 1H), 7.94 – 7.98 (d, J = 7.7 Hz, 1H), 8.56 – 8.65 (m, 3H), 9.83 – 9.86 (s, 1H), 10.32 – 10.39 (s, 1H), 10.83 – 10.85 (d, J = 2.7 Hz, 1H).HRMS (m/z): [M]+ calcd. for $C_{37}H_{41}N_{10}O_{7}$, 737.3160; found, 737.3156.

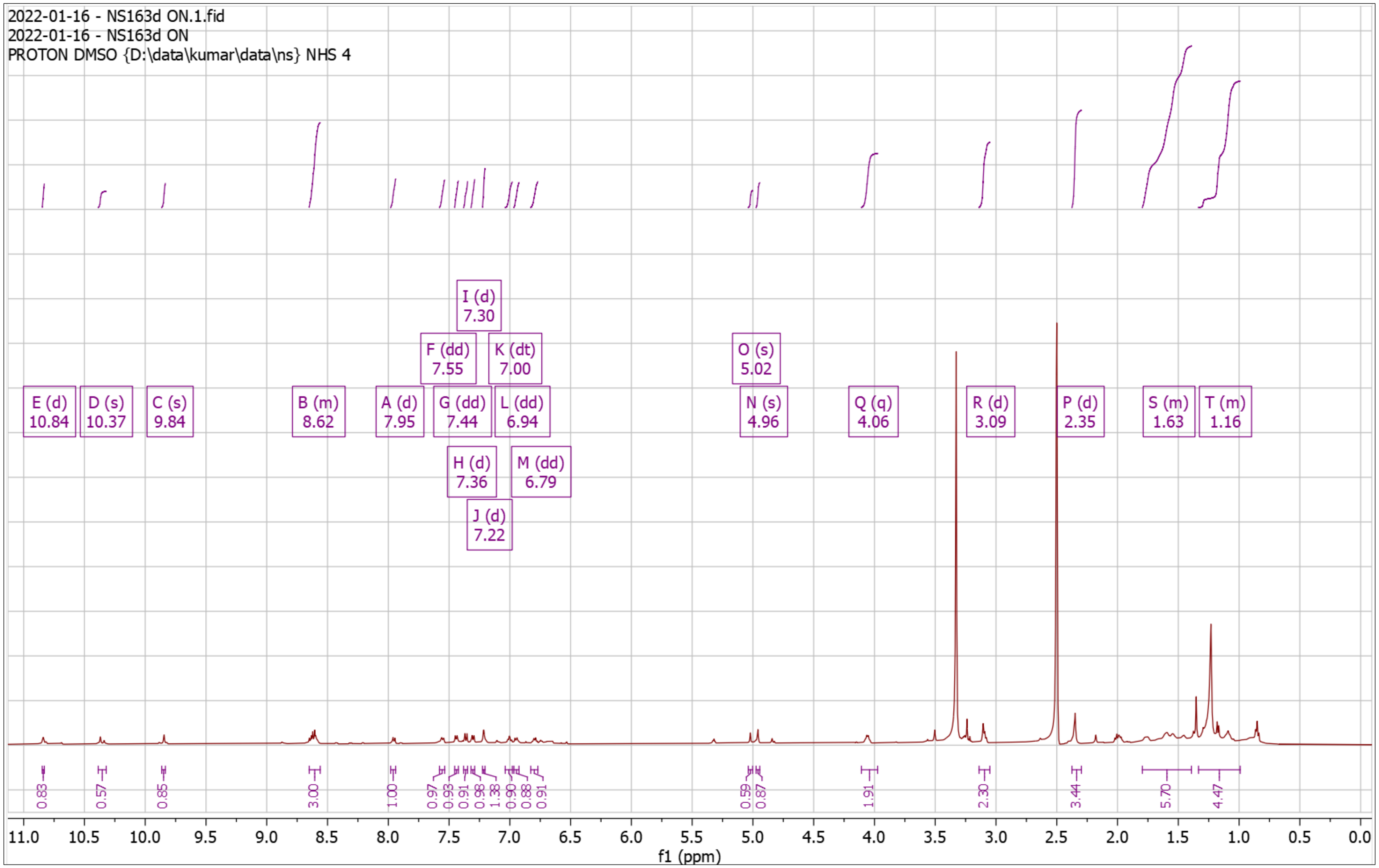

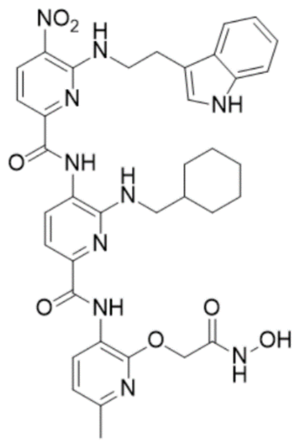

Supplementary Fig. 156. High Resolution Mass Spectrum for NS163

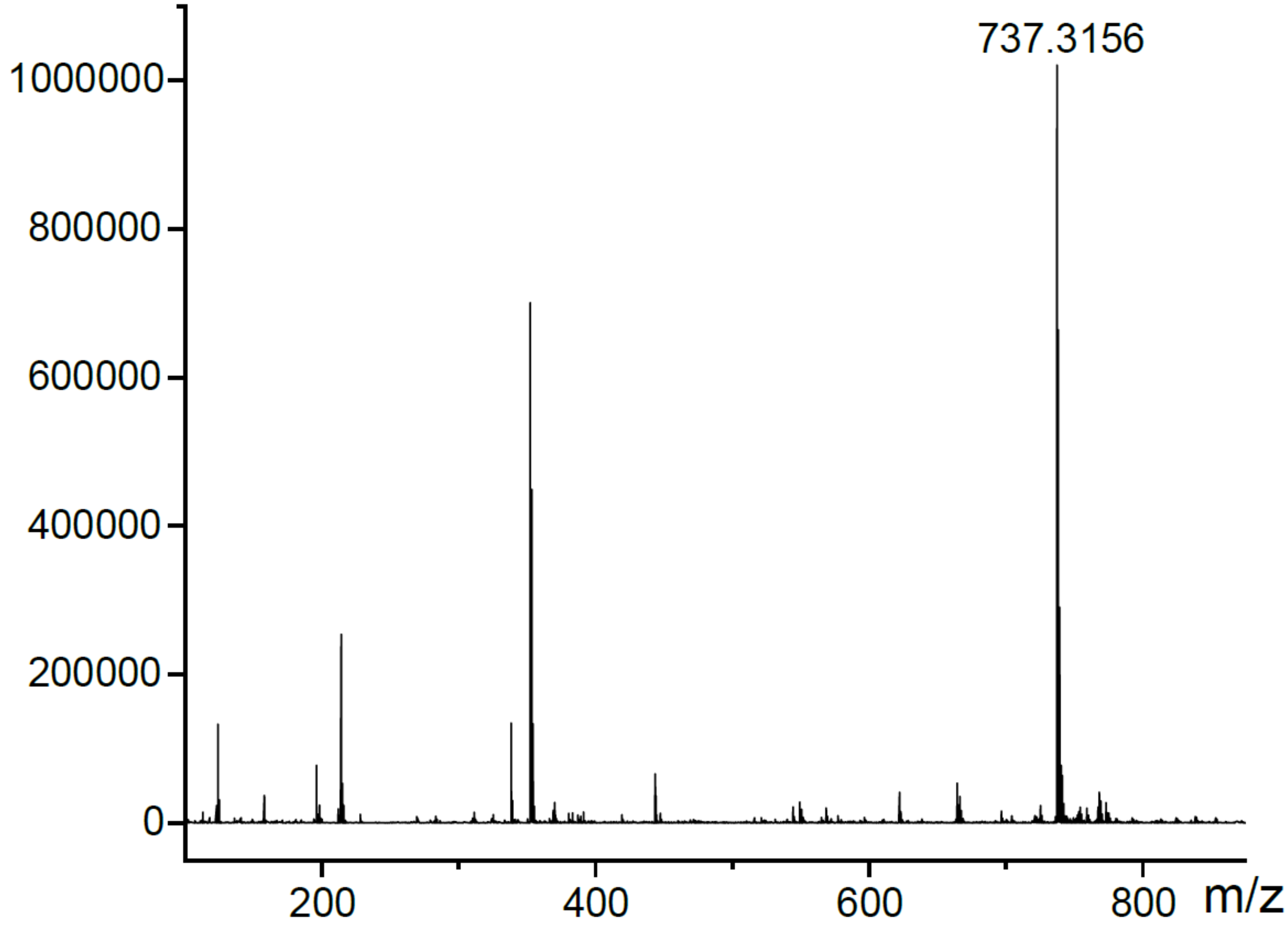

SUPPLEMENTARY REFERENCES

1. Yadav, J. S., Balanarsaiah, E., Raghavendra, S. & Satyanarayana, M. Chemoselective hydrolysis of tert-butyl esters in acetonitrile using molecular iodine as a mild and efficient catalyst. Tetrahedron Lett. 47, 4921–4924 (2006).
2. Wu, Y., Limburg, D. C., Wilkinson, D. E., Vaal, M. J. & Hamilton, G. S. A mild deprotection procedure for tert-butyl esters and tert-butyl ethers using ZnBr2 in methylene chloride. Tetrahedron Lett. 41, 2847–2849 (2000).
