# Supplementary material for: Protein mimetic 2D FAST rescues alpha synuclein aggregation mediated early and post disease Parkinson’s phenotypes
Source: Nat Commun. 2024 Apr 30;15:3658. doi: 10.1038/s41467-024-47980-4 (PMC11061149; doi:10.1038/s41467-024-47980-4)
Supplement: Supplementary file 3 — Description of Additional Supplementary Files [file 41467_2024_47980_MOESM3_ESM.pdf]

Description of Additional Supplementary Files

Supplementary Movie 1: Representative movie of N2 worms on day 3.

Supplementary Movie 2: Representative movie of NL5901 worms treated with 50 µM NS163 (on day 2 and 4) on day 3.

Supplementary Movie 3: Representative movie of NL5901 worms on day 3.

Supplementary Movie 4: Representative movie of N2 worms on day 8.

Supplementary Movie 5: Representative movie of NL5901 worms treated with 50 µM NS163 (on day 2 and 4) on day 8.

Supplementary Movie 6: Representative movie of NL5901 worms on day 8.

Supplementary Movie 7: Representative movie of the chemotaxis assay of N2 worms on day 3.

Supplementary Movie 8: Representative movie of the chemotaxis assay of UA196 worms on day 3.

Supplementary Movie 9: Representative movie of the chemotaxis assay of UA196 worms treated with 50 µM NS163 (on day 2 and 4) on day 3.

Supplementary Movie 10: Representative movie of the chemotaxis assay of UA196 worms on day 10.

Supplementary Movie 11: Representative movie of the chemotaxis assay of UA196 worms treated with 50 µM NS163 (on day 2 and 4) on day 10.

Supplementary Movie 12: Representative movie of the chemotaxis assay of N2 worms on day 10.

Supplementary Movie 13: Representative movie of the chemotaxis assay of UA196 worms treated with 50 µM NS132 (on day 2 and 4) on day 3.

Supplementary Movie 14: Representative movie of the chemotaxis assay of UA196 worms treated with 50 µM NS132 (on day 2 and 4) on day 10.
