# Supplementary material for: Protein mimetic 2D FAST rescues alpha synuclein aggregation mediated early and post disease Parkinson’s phenotypes
Source: Nat Commun. 2024 Apr 30;15:3658. doi: 10.1038/s41467-024-47980-4 (PMC11061149; doi:10.1038/s41467-024-47980-4)
Supplement: Supplementary file 18 — Reporting Summary [file 41467_2024_47980_MOESM18_ESM.pdf]

nature portfolio

Corresponding author(s): Sunil Kumar

Last updated by author(s): Jan 31, 2024

Reporting Summary

Nature Portfolio wishes to improve the reproducibility of the work that we publish. This form provides structure for consistency and transparency in reporting. For further information on Nature Portfolio policies, see our Editorial Policies and the Editorial Policy Checklist.

Statistics

For all statistical analyses, confirm that the following items are present in the figure legend, table legend, main text, or Methods section.

n/a	Confirmed	
☐	☒	The exact sample size (*n*) for each experimental group/condition, given as a discrete number and unit of measurement
☐	☒	A statement on whether measurements were taken from distinct samples or whether the same sample was measured repeatedly
☐	☒	The statistical test(s) used AND whether they are one- or two-sided<br>*Only common tests should be described solely by name; describe more complex techniques in the Methods section.*
☒	☐	A description of all covariates tested
☒	☐	A description of any assumptions or corrections, such as tests of normality and adjustment for multiple comparisons
☐	☒	A full description of the statistical parameters including central tendency (e.g. means) or other basic estimates (e.g. regression coefficient) AND variation (e.g. standard deviation) or associated estimates of uncertainty (e.g. confidence intervals)
☐	☒	For null hypothesis testing, the test statistic (e.g. *F*, *t*, *r*) with confidence intervals, effect sizes, degrees of freedom and *P* value noted<br>*Give P values as exact values whenever suitable.*
☒	☐	For Bayesian analysis, information on the choice of priors and Markov chain Monte Carlo settings
☒	☐	For hierarchical and complex designs, identification of the appropriate level for tests and full reporting of outcomes
☒	☐	Estimates of effect sizes (e.g. Cohen's *d*, Pearson's *r*), indicating how they were calculated

Our web collection on statistics for biologists contains articles on many of the points above.

Software and code

Policy information about availability of computer code

Data collection	The software programs and the instrument used for data collection in this manuscript (open source and commercial) are clearly indicated in the Methods section in the subheadings for various methods and techniques. The ThT aggregation curves were fit using OriginPro software (Version 9.1). The processing of the confocal images of HEK cells and C elegans was carried out using ImageJ software from Olympus. The SDS PAGE gel intensities were determined using ImageJ software from Olympus. The NMR data collected using MestReNova NMR (Version 12.0.4) software and Origin 2020b. The drawing of the chemical structures in synthetic schemes was carried out using ChemDraw Professional (Version 20.0). The data for ITC titration were processed using NanoAnalyze software (TA Instruments, New Castle, DE). The C. elegans based activity count and behavioral deficits curves were determined using WMicroTracker ARENA plate reader (Phylumtech, Santa Fe, Argentina). The videos for c elegans strains under various conditions were taken on Olympus EP50 Digital Microscope using the Olympus Software EPview software (EPview V3.7.7).
Data analysis	The data analysis for ThT aggregation assays, Fluorescence polarization titrations, MTT toxicity assays, Inclusion counts for HEK cells and C elegans, Activity counts for C. elegans, and behavioral deficits curves of C. elegans were carried out using OriginPro software (Version 9.1), Origin 2020b, and GraphPad Prism (Version 9.3.1) softwares. The NMR data analysis was carried out using MestReNova NMR (Version 12.0.4) software and Origin 2020b. The analysis of the confocal images of HEK cells and C. elegans was carried out using ImageJ software from Olympus. The SDS PAGE gel intensities were determined using ImageJ software from Olympus. The drawing of the chemical structures in synthetic schemes was carried out using ChemDraw Professional (Version 20.0). The date for ITC titrations were processed using NanoAnalyze software (TA Instruments, New Castle, DE).

For manuscripts utilizing custom algorithms or software that are central to the research but not yet described in published literature, software must be made available to editors and reviewers. We strongly encourage code deposition in a community repository (e.g. GitHub). See the Nature Portfolio guidelines for submitting code & software for further information.

Data

Policy information about availability of data

All manuscripts must include a data availability statement. This statement should provide the following information, where applicable:

- Accession codes, unique identifiers, or web links for publicly available datasets
- A description of any restrictions on data availability
- For clinical datasets or third party data, please ensure that the statement adheres to our policy

All the datasets generated and analyzed during the current study are also available from the corresponding author. Source data is available for Figs. 1–6 and Supplementary Figs. 4-10, 16–19, and 21–38 in the associated source data file. Source data are provided with this paper.

Human research participants

Policy information about studies involving human research participants and Sex and Gender in Research.

Reporting on sex and gender	*Use the terms sex (biological attribute) and gender (shaped by social and cultural circumstances) carefully in order to avoid confusing both terms. Indicate if findings apply to only one sex or gender; describe whether sex and gender were considered in study design whether sex and/or gender was determined based on self-reporting or assigned and methods used. Provide in the source data disaggregated sex and gender data where this information has been collected, and consent has been obtained for sharing of individual-level data; provide overall numbers in this Reporting Summary. Please state if this information has not been collected. Report sex- and gender-based analyses where performed, justify reasons for lack of sex- and gender-based analysis.*
Population characteristics	*Describe the covariate-relevant population characteristics of the human research participants (e.g. age, genotypic information, past and current diagnosis and treatment categories). If you filled out the behavioural & social sciences study design questions and have nothing to add here, write "See above."*
Recruitment	*Describe how participants were recruited. Outline any potential self-selection bias or other biases that may be present and how these are likely to impact results.*
Ethics oversight	*Identify the organization(s) that approved the study protocol.*

Note that full information on the approval of the study protocol must also be provided in the manuscript.

Field-specific reporting

Please select the one below that is the best fit for your research. If you are not sure, read the appropriate sections before making your selection.

☒ Life sciences ☐ Behavioural & social sciences ☐ Ecological, evolutionary & environmental sciences

For a reference copy of the document with all sections, see nature.com/documents/nr-reporting-summary-flat.pdf

Life sciences study design

All studies must disclose on these points even when the disclosure is negative.

Sample size	All in vitro experiments were carried out with three independent experiments (n = 3) and each independent experiments was repeated three times. For NMR experiments n=1, but at least one NMR experiment was repeated to confirm the reproducibility (data was not shown in the manuscript). Also, the NMR experiments between alpha-synuclein and NS132 were conducted under different conditions (without fibers and with fibers) and a consistent trend of the change in the chemical shift volumes was observed, which supports the reproducibility of the data. The cellular experiments (Proteostat, cell viability, confocal imaging, aggregate count etc) were carried out using four independent experiments (n = 4) and each experiment consisted of at least four technical replicates. For the in vivo experiments (C. elegans), the experiments were carried out using at least three independent experiments (n = 3) and each independent experiment consisted of at least two technical replicates.
Data exclusions	No data was excluded from the manuscript.
Replication	All attempts for the replication of data were successful.
Randomization	At least one randomization experiment was carried out to monitor the effect of molecules on synuclein aggregation in HEK cells and C. elegans. The allocation of the samples was carried out randomly without any prior selection. The randomly allocated samples then treated with different experiment conditions to pursue the experiments. The samples were prepared by one lab member and the data was collected by a second lab member. The identity of the samples was not revealed to the second lab member, who was collecting data to avoid biasness in the experiment. This experiment was carried out for both HEK cells and C. elegans models.
Blinding	At least one blinding experiment was carried out to monitor the effect of molecules on alpha-synuclein aggregation in HEK cells and C. elegans

Blinding	models. During the blinding experiment, a second member of the lab conducted the blinding part of the experiments and the first member of the lab conducted the experiment without knowing the identity of the samples to avoid any biasness in the experiments. At the end of the experiment, a third member of the lab matched the identity and results of the experiment, which were included in the final datasets of the experiments.

Reporting for specific materials, systems and methods

We require information from authors about some types of materials, experimental systems and methods used in many studies. Here, indicate whether each material, system or method listed is relevant to your study. If you are not sure if a list item applies to your research, read the appropriate section before selecting a response.

Materials & experimental systems

n/a	Involved in the study
☐	☒ Antibodies
☐	☒ Eukaryotic cell lines
☒	☐ Palaeontology and archaeology
☐	☒ Animals and other organisms
☒	☐ Clinical data
☒	☐ Dual use research of concern

Methods

n/a	Involved in the study
☒	☐ ChIP-seq
☒	☐ Flow cytometry
☒	☐ MRI-based neuroimaging

Antibodies

Antibodies used	Anti-pS129-a-syn/ 81a (monoclonal) Catalog # 825702, Lot # B318449<br>Anti-p62 (monoclonal) Catalog # NBP1-42821SS, Lot # B-4<br>Goat anti-mouse Alexa Fluor Plus 647 (red color) Catalog # A32787 TR, Lot # XB346098<br><br>We have specified the dilution used for secondary antibodies in the methods section of the manuscript.<br>The dilution factor for both the primary and secondary antibodies were 1:1000 (v/v) in TBST buffer containing 5% BSA
Validation	All antibodies (Primary and secondary) are commercially available and are well established. All of the antibodies have been tested, validated, and utilized in multiple study that are published and well documented.

Eukaryotic cell lines

Policy information about cell lines and Sex and Gender in Research

Cell line source(s)	HEK293 cells with transfection and over expression of A53T mutant alpha-synuclein-YFP were acquired via a generous Gift from Prof. Marc Diamond's lab (University of Texas Southwestern, Dallas, Texas) using a Materials Transfer Agreement.
Authentication	HEK293 cells (Overexpressing A53T mutant alpha-synuclein-YFP) were authenticated by Prof. Marc Diamond's lab (University of Texas Southwestern, Dallas, Texas). Our lab did not authenticate the cell lines.
Mycoplasma contamination	The testing of the contamination of cell lines were carried out by Prof. Marc Diamond's lab (University of Texas Southwestern, Dallas, Texas) (HEK293 modified cell lines). Our lab did not test these cell lines for mycoplasma contamination.
Commonly misidentified lines<br>(See ICLAC register)	No commonly misidentified cell lines were used in the current study.

Animals and other research organisms

Policy information about studies involving animals; ARRIVE guidelines recommended for reporting animal research, and Sex and Gender in Research

Laboratory animals	In this study, three strains of C. elegans were used, including N2, NL5901, and UA196. The maintenance of the C. elegans strains and their treatment with small molecules were carried out by using well established and published protocols. The C. elegans strain were mostly female (male population freequency was less than 0.002). The C. elegans strains (N2 and NL5901) were purchased from CGC (University of Minnesota) and the UA196 worm strain was a generous gift from Prof. Guy A Caldwell's lab at the University of Alabama (Tuscaloosa, AL, USA). The C. elegans strains used in the experiments when they were less than six month old from repetitive cultures.
Wild animals	No wild animals were used in the study.
Reporting on sex	The C. elegans strain were predominently female (male population freequency was less than 0.002).
Field-collected samples	*For laboratory work with field-collected samples, describe all relevant parameters such as housing, maintenance, temperature, photoperiod and end-of-experiment protocol OR state that the study did not involve samples collected from the field.*

Ethics oversight	No ethical approval required for the C. elegans strains. All Cell protocols and experiments were approved by the Institutional Committee (IBC).

Note that full information on the approval of the study protocol must also be provided in the manuscript.
